# CREB3L1 deficiency impairs odontoblastic differentiation and molar dentin deposition partially through the TMEM30B

**DOI:** 10.1038/s41368-024-00322-y

**Published:** 2024-10-10

**Authors:** Yuanyuan Li, Yuxiu Lin, Jinqiang Guo, Delan Huang, Huanyan Zuo, Hanshu Zhang, Guohua Yuan, Huan Liu, Zhi Chen

**Affiliations:** 1 State Key Laboratory of Oral & Maxillofacial Reconstruction and Regeneration, Key Laboratory of Oral Biomedicine Ministry of Education, Hubei Key Laboratory of Stomatology, School & Hospital of Stomatology, Wuhan University, Wuhan, China; 2 Department of Cariology and Endodontics, School of Stomatology, Wuhan University, Wuhan, China; 3 Frontier Science Center for Immunology and Metabolism, Wuhan University, Wuhan, China; 4 TaiKang Center for Life and Medical Sciences, Wuhan University, Wuhan, China

**Keywords:** Cell biology, Differentiation

## Abstract

Odontoblasts are primarily responsible for synthesizing and secreting extracellular matrix proteins, which are crucial for dentinogenesis. Our previous single-cell profile and RNAscope for odontoblast lineage revealed that cyclic adenosine monophosphate responsive element-binding protein 3 like 1 (*Creb3l1*) was specifically enriched in the terminal differentiated odontoblasts. In this study, deletion of *Creb3l1* in the *Wnt1+* lineage led to insufficient root elongation and dentin deposition. Assay for transposase-accessible chromatin with high-throughput sequencing (ATAC-seq) and RNA sequencing were performed to revealed that in CREB3L1-deficient mouse dental papilla cells (mDPCs), the genes near the closed chromatin regions were mainly associated with mesenchymal development and the downregulated genes were primarily related to biological processes including cell differentiation, protein biosynthesis and transport, all of which were evidenced by a diminished ability of odontoblastic differentiation, a significant reduction in intracellular proteins, and an even greater decline in extracellular supernatant proteins. Dentin matrix protein 1 (*Dmp1*), dentin sialophosphoprotein (*Dspp*), and transmembrane protein 30B (*Tmem30b*) were identified as direct transcriptional regulatory targets. TMEM30B was intensively expressed in the differentiated odontoblasts, and exhibited a significant decline in both CREB3L1-deficient odontoblasts in vivo and in vitro. Deletion of *Tmem30b* impaired the ability of odontoblastic differentiation, protein synthesis, and protein secretion in mDPCs. Moreover, overexpressing TMEM30B in CREB3L1-deficient mDPCs partially rescued the extracellular proteins secretion. Collectively, our findings suggest that CREB3L1 participates in dentinogenesis and facilitates odontoblastic differentiation by directly enhancing the transcription of *Dmp1, Dspp*, and other differentiation-related genes and indirectly promoting protein secretion partially *via* TMEM30B.

## Introduction

Dentin is the primary load-bearing tissue in teeth, and crucial for their durability.^1^ The formation of dentin, dentinogenesis, ensues from the deposition of an intricate extracellular matrix (ECM), which is synthesized and secreted by the odontoblasts.^2^ The ECM proteins form the initial organic dentinal matrix, representing the non-mineralized predentin.^3^ Dentin ECM contains abundant noncollagenous proteins (NCPs) considered to be responsible for initiating and controlling the mineralization process that transforms predentin into dentin.^4^ The small integrin-ligand N-linked glycoproteins (SIBLING) family, including dentin matrix protein 1 (DMP1) and dentin sialophosphoprotein (DSPP), are representatives of the NCPs in ECM proteins.^4,5^ DMP1 deficiency resulted in partial failure of maturation of predentin to dentin, hypomineralization, and expanded pulp and root canal cavities during postnatal tooth development.^6^ Mutations of *DSPP* led to dentinogenesis imperfecta characterized by enlarged or smaller dental pulp chambers.^7,8^ However, the transcription regulation of these ECM proteins during odontoblastic differentiation or lineage specification remained unclear. In order to obtain a global view for transcription regulation of the ECM proteins, we performed an integrated assay using single cell RNA-seq and ATAC-seq, and found a group of transcription factors (TFs) regulating the odontoblastic differentiation in a stage-dependent manner. We previously disclosed that *klf4* directly upregulate the transcription of *Dmp1*, which pre-determined the odontoblast lineage commitment.^9^ Besides, we noticed the regulons of basic leucine zipper (bZIP) family was mainly enriched in the mature odontoblasts, among which cyclic adenosine monophosphate (cAMP) responsive element-binding protein 3 like 1 (CREB3L1) was specifically upregulated.^10^

CREB3L1, also known as old astrocyte specifically induced substance (OASIS), was identified as a transcription factor.^11^ Ordinarily, the protein resides in the endoplasmic reticulum (ER) membrane. However, in response to ER stress, it undergoes translocation to the Golgi membrane for cleavage. The N-terminal segment of CREB3L1, comprising the bZIP domain, then migrates to the nucleus, where it initiates the transcriptional activation of target genes.^12,13^ Mutations in *CREB3L1* have been reported in individuals suffering from oligodontia, osteogenesis imperfecta, or fatal osteogenesis imperfecta.^14–18^ However, the specific reasons behind its heightened expression in mature odontoblasts and its potential contribution to dentinogenesis require further study.

In this study, we used *Wnt1-Cre* to ablate *Creb3l1* in the neural crest lineage. Shorter tooth roots and insufficient dentin deposition were observed in *Wnt1-Cre; Creb3l1*^*f/f*^ conditional knockout mice (cKO). We conducted assays for transposase-accessible chromatin with high-throughput sequencing (ATAC-seq)^19^ and RNA sequencing (RNA-seq). These analyses demonstrated that the downregulation of CREB3L1 in mDPCs, after 5 days of differentiation induction, affected biological processes associated with protein biosynthesis, transport, and epithelial-to-mesenchymal transition. Besides of the direct downregulation of *Dmp1 and Dspp*, slightly reduced intracellular and greatly decreased supernatant protein levels were also observed in the CREB3L1-deficient mDPCs. Combining the two sequencing results, *Tmem30b*, one of the highest-ranked possible target genes responsible for intracellular protein transport was acquired. Deficiency of TMEM30B impaired the odontoblastic differentiation ability of mDPCs by slightly decreasing the intracellular protein and severely reducing the supernatant protein levels. Overexpression of TMEM30B partially rescued the total amount of extracellular supernatant proteins in CREB3L1-deficient mDPCs. Our findings indicate that CREB3L1 deficiency results in inadequate root elongation and dentin deposition. Additionally, CREB3L1-regulated protein biosynthesis occurs independently of TMEM30B, and protein transport is partially mediated by TMEM30B, leading to a reduction in the total amount of both intracellular and ECM proteins.

## Results

### Deficiency of *Creb3l1* in *Wnt1+* lineage led to shorter roots and thinner dentin

**Fig. 1:**
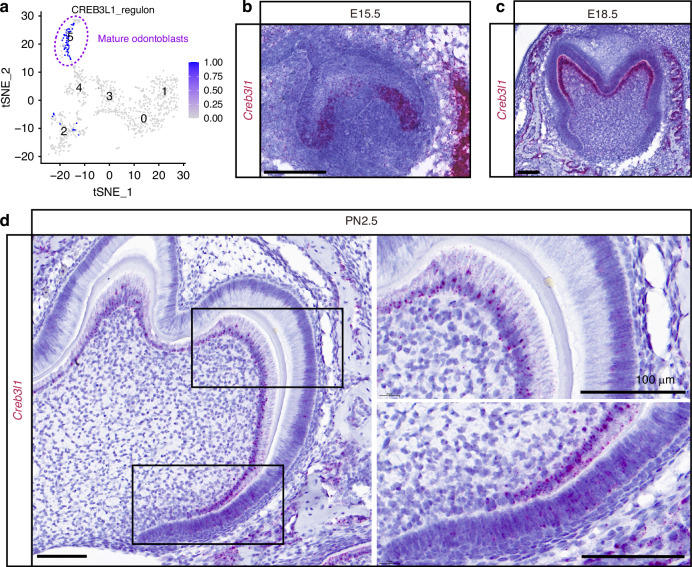
*Creb3l1* mRNA exhibited high expression levels in polarized and mature odontoblasts. **a** Regulon of CREB3L1 in scRNA-seq profile for dental mesenchyme isolated from PN0 mouse lower molar.^10^ The CREB3L1 regulons were notably enriched in mature odontoblasts population. **b**–**d** RNAscope in situ hybridization was performed on the dental germ at embryonic day 15.5, 18.5 (E15.5, E18.5), and postnatal day 2.5 (PN2.5) developmental stages. The *Creb3l1* mRNA was not expressed in dental mesenchymal cells but was specifically expressed in the epithelium and bone at E15.5 (**b**). *Creb3l1* mRNA exhibited a high level of expression in the polarized odontoblasts and bone at E18.5 (**c**). *Creb3l1* mRNA is localized in polarized and mature odontoblasts at PN2.5 (**d**). Scale bar = 100 μm

We firstly revisited the published scRNA-seq profile of the odontoblast lineage, and found regulon of CREB3L1 was specifically highly enriched at the terminal stage of odontoblast differentiation according to our previous single-cell RNA-seq results^10^ (Fig. 1a). RNAscope staining revealed that *Creb3l1* mRNA was not expressed in preodontoblasts at embryonic day 15.5 (E15.5) (Fig. 1b), whilst its expression was evident in polarized odontoblasts at E18.5 (Fig. 1c), and it was continuously expressed in mature odontoblasts at postnatal day 2.5 (PN2.5) (Fig. 1d). To uncover its necessity in dentinogenesis, we generated *Wnt1-Cre; Creb3l1*^*f/f*^ conditional knockout mice (cKO), which were used to specifically delete *Creb3l1* in the neural crest lineage. All conditional knockout mice survived and displayed no statistically significant differences in either body size or weight compared to wildtype (WT) mice (Supplementary Fig. S1a, b). To assess the efficiency of knockout and examine tooth morphology in cKO mice, hematoxylin and eosin (HE) staining and immunohistochemistry (IHC) staining of CREB3L1 were conducted on molars at postnatal day 0.5 (PN 0.5) (Supplementary Fig. S2a, b). As expected, the dental papilla showed no expression of CREB3L1 (Supplementary Fig. S2a). However, there were no significant differences in tooth crown morphology between WT and cKO (Supplementary Fig. S2b).

**Fig. 2:**
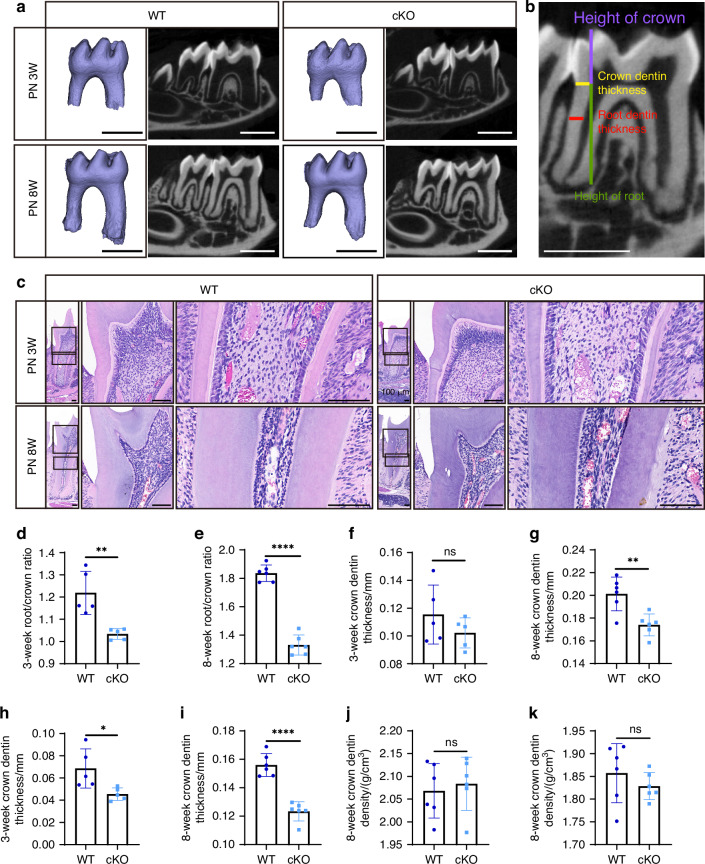
Conditional deletion of *Creb3l1* in neural crest lineage resulted in shorter root and thinner dentin. **a** Micro-CT scanning was performed to measure the mandibular first molar of PN 3 W and PN 8 W. Scale bar = 1 mm. **b** The measurement items, height of crown (violet line), crown dentin thickness (yellow line), root dentin thickness (red line), and height of root (green line) were included in the analysis. Scale bar = 1 mm. **c** HE staining was performed on wild-type (WT) and cKO mice at PN 3 W and PN 8 W. Scale bar = 100 μm. **d** The quantitative data of root-to-crown ratio from WT and cKO mice at PN 3 W. **e** The quantitative data of the root-to-crown ratio from PN 8 W mice. **f** The quantitative data of crown dentin thickness from WT and cKO mice at PN 3 W. **g** The quantitative data of crown dentin thickness from PN 8 W mice. **h** The quantitative data of root dentin thickness from WT and cKO mice at PN 3 W. **i** The quantitative data of root dentin thickness from PN 8 W mice. **j** The quantitative data of crown dentin density from PN 8 W mice. **k** The quantitative data of root dentin density from PN 8 W mice. *n* ≥ 5. ns not significant, *P* > 0.05; *P < 0.05; **P < 0.01; ****P < 0.000 1

Micro-CT scans were conducted on mice at two separate developmental periods: postnatal 3 weeks (PN 3 W), the time of tooth root development, and postnatal 8 weeks (PN 8 W), the time of completed body development (Fig. 2a). The conditional deletion of *Creb3l1* in the neural crest lineage demonstrated no effect on the number of teeth or crown morphology. The mandibular height below the root furcation of the first molar was analyzed (Supplementary Fig. S3a). It was found that this height was slightly decreased in cKO mice compared to WT mice at PN 3 W (Supplementary Fig. S3b), but significantly reduced at PN 8 W (Supplementary Fig. S3c). The mandibular alveolar bone (MAB) between the roots of the first molar (MAB1) and between the first and second molars (MAB2) were analyzed (Supplementary Figs. S4a, b, S5a, b). It was found that at PN 3 W, half of the cKO mice exhibited a significant decrease in bone mass. However, the other half did not exhibit any significant changes. Furthermore, there was no statistically significant difference in the bone volume (BV) to tissue volume (TV) ratio (BV/TV), trabecular thickness (Tb.Th), trabecular number (Tb.N), and trabecular separation (Tb.Sp) of cKO mice when compared with WT mice at PN 3 W (Supplementary Figs. S4c–f, S5c–f). A statistical analysis of all samples at PN 8 W revealed a statistically significant decrease in BV/TV in cKO mice in the mandible at both sites (Supplementary Figs. S4g, S5g). Additionally, a statistically significant reduction in trabecular number (Tb.N) was observed exclusively in the MAB1 in the PN 8 W cKO mice (Supplementary Fig. S4i). No statistically significant differences were observed for the remaining indicators (Supplementary Figs. S4h, j, S5h–j). Objectively, only a subset of the cKO mice exhibited a more pronounced reduction in bone mass when examined individually. The cementum of the first molar at PN 8 W exhibited a reduction in the amount of cementum in cKO mice compared to WT mice (Supplementary Fig. S6).

The crown and root length, the thickness of crown and root dentin were analyzed to investigate potential abnormalities in tooth development^20^ (Fig. 2a, b). Shorter roots were observed in PN 3 W and PN 8 W cKO mice (Fig. 2a). The histological examination (Fig. 2c) and Micro-CT analysis of PN 3 W (Fig. 2d) and PN 8 W (Fig. 2e) mice provided further evidence to support this observation. Although there was a reduction in crown dentin thickness in cKO mice at PN 3 W in comparison to WT mice, this difference was not statistically significant (Fig. 2f). However, a significant difference was observed in the reduction in crown dentin thickness in cKO mice at PN 8 W (Fig. 2g). Both PN 3 W and PN 8 W cKO mice displayed thinner root dentin in contrast to WT mice (Fig. 2h, i).

Although there were macroscopic changes, it is unclear if any alterations in density and microstructure occurred in dentin. The Micro-CT analysis was employed to assess the density of dentin in both the crown and root of PN 8 W mice. The density of the crown and root exhibited no significant alterations (Fig. 2j, k). Scanning electron microscopy (SEM) was used to examine the dentinal tubules at the crown, cementoenamel junction (CEJ), and root. No significant changes in the dentinal tubule structure and dentinal tubules were observed (Fig. S7). The findings indicate that CREB3L1 is associated with total dentin deposition, but not with the density or structure of dentinal tubules.

### Downregulation of *Creb3l1* attenuated the odontoblastic differentiation capability of mDPCs

**Fig. 3:**
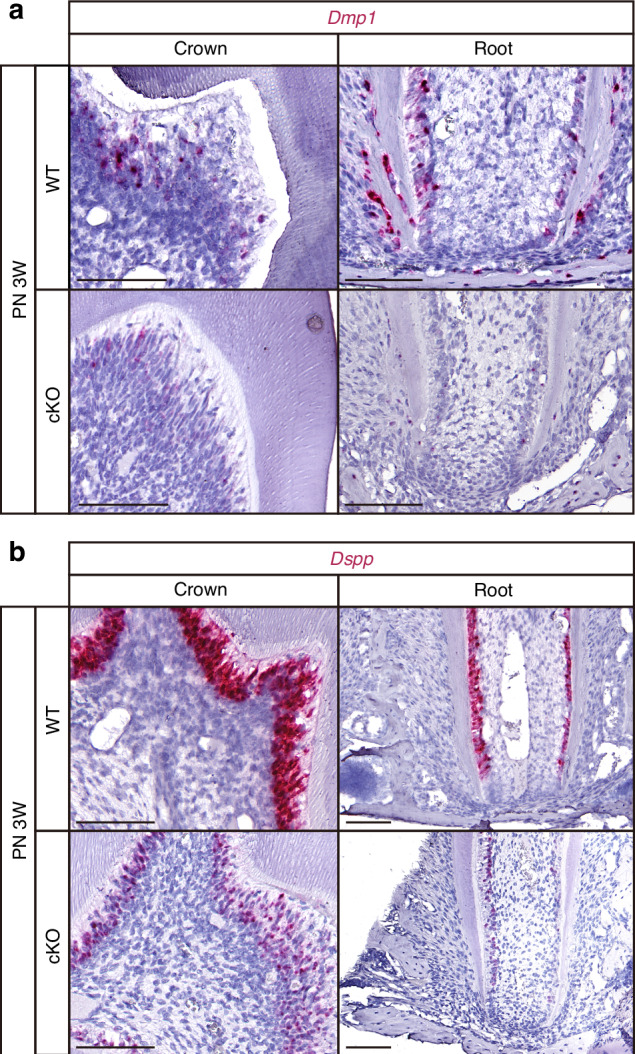
Deficiency of CREB3L1 resulted in a significant decrease in the level of *Dmp1* and *Dspp* in the first molar. **a** The RNAscope staining of *Dmp1* in odontoblasts of the first molar of WT and cKO mice at PN 3 W. Scale bar = 100 μm. **b** The RNAscope staining of *Dspp* in odontoblasts of the first molar of WT and cKO mice at PN 3 W. Scale bar = 100 μm

**Fig. 4:**
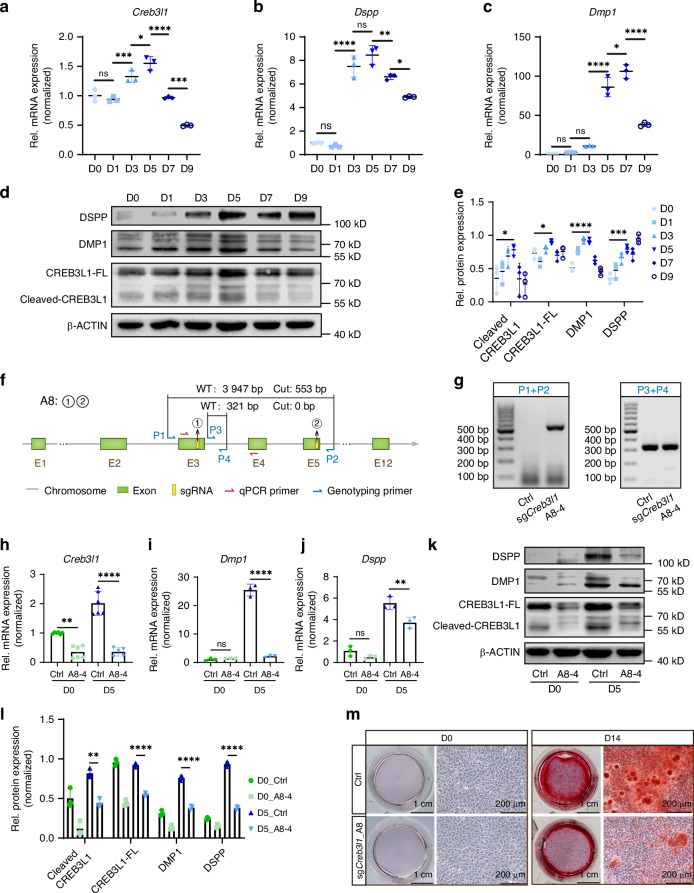
Downregulation of CREB3L1 inhibited the odontoblastic differentiation of mDPCs. **a**–**c** The mRNA expression level of *Creb3l1*, *Dmp1*, *Dspp* during differentiation induction. **d** The protein expression levels of the full length of CREB3L1 (CREB3L1-FL), Cleaved-CREB3L1, DMP1, and DSPP were detected during differentiation induction. **e** Quantification of the relative levels of protein expression in **d**. **f** Design of sgRNAs for *Creb3l1* knockout. **g** Genotyping results for a monoclonal heterozygous cell line with knockdown of *Creb3l1* (sg*Creb3l1*_A8-4). **h**–**j** The mRNA expression of *Creb3l1*, *Dmp1*, and *Dspp* decreased in sg*Creb3l1*_A8-4 cells after a 5-day induction of differentiation. **k** The expression of the CREB3L1-FL, Cleaved-CREB3L1, DMP1, and DSPP were decreased in sg*Creb3l1*_A8-4 during differentiation induction. **l** Quantification of the relative protein expression levels of **k**. **m** Alizarin red S staining was utilized to visualize calcium nodules in both the control (Ctrl) and sg*Creb3l1*_A8 groups after 14 days of differentiation induction. Scale bar = 1 cm, scale bar = 200 μm. *n* ≥ 3. ns, not significant, *P* > 0.05; *P < 0.05; **P < 0.01; ***P < 0.001; ****P < 0.000 1

The RNAscope technique was employed to ascertain the mRNA expression of genes encoding proteins associated with odontoblast terminal differentiation in order to evaluate the potential role of CREB3L1 in regulating odontoblast differentiation at the transcriptional level in vivo. The expression of *Dmp1* (Fig. 3a) and *Dspp* (Fig. 3b) in odontoblasts of cKO mice was observed to be diminished in comparison to WT mice in vivo, indicating that CREB3L1 may regulate the expression of *Dmp1* and *Dspp*. To determine the mechanism underlying the shorter root and thinner dentin in cKO mice, the mDPC6T-Cas9 cell line,^21,22^ which maintained the differentiation capacity of primary cultured mouse dental papilla cells and constitutively expressed the CAS9 protein, was used to knock down *Creb3l1* in vitro. The expression of CREB3L1 was initially verified during the differentiation induction of the mDPC6T-Cas9 cell line. The successful induction of differentiation was confirmed through the upregulation of differentiation-related proteins, including DMP1 and DSPP. Notably, the mRNA expression of *Creb3l1* peaked on the fifth day after differentiation induction (Fig. 4a). The mRNA expressions of *Dspp* and *Dmp1* increased during the differentiation induction (Fig. 4b, c) along with the increased protein levels (Fig. 4d, e). Since cleavage is required for CREB3L1 to function, the active form is the cleaved CREB3L1 fragment (Cleaved-CREB3L1). Western blot analysis detected two distinct bands, CREB3L1-FL (full-length CREB3L1) and Cleaved-CREB3L1 (Fig. 4d). Additionally, the highest expression of CREB3L1 was observed on the fifth day of differentiation induction as well (Fig. 4d, e). Furthermore, the role of CREB3L1 was explored by using CRISPR/CAS9 technology^23^ to knock out the *Creb3l1* gene with a pair of single guide RNAs (sgRNAs) designated A8 (Fig. 4f, Supplementary Table S1). The P1 and P2 primers (Supplementary Table S1) were used to identify the chromosome that was successfully excised if a 553 bp fragment appeared (Fig. 4f). P3 and P4 primers (Supplementary Table S1) were used to identify the WT chromosome when 321 bp appeared (Fig. 4f). If both 553 bp and 321 bp were observed, the chromosome was considered to be incompletely cleaved and was regarded as a heterozygous cell line.

The monoclonal cell line was obtained via limiting dilution of the mDPC6T-Cas9 cell line treated with A8 sgRNAs. Two heterozygous monoclones, sg*Creb3l1*_A8-4 and sg*Creb3l1*_A8-8, were identified from the A8 sgRNA-treated cell pool (Supplementary Figs. S8a, Fig. 4g). The groups were divided into day 0 (D0), without differentiation induction, and day 5 (D5), with differentiation induction for 5 days. The mRNA levels of *Creb3l1* (Fig. 4h)*, Dmp1* (Fig. 4i), and *Dspp* (Fig. 4j) were significantly decreased in sg*Creb3l1*_A8-4 cells on D5. These findings indicate that CREB3L1 was involved in the transcriptional regulation of *Dmp1* and *Dspp* expression.

Finally, for the subsequent experiments, sg*Creb3l1*_A8-4 was selected for further studies. After 5 days of differentiation induction, the protein levels of CREB3L1, DMP1, and DSPP were found to be reduced in the sg*Creb3l1*_A8-4 group (Fig. 4k, l). A similar outcome was observed in the sg*Creb3l1*_A8-8 group (Supplementary Fig. S8b, c). The mineralized nodule formation ability of mDPC6T-Cas9 cells treated with A8 sgRNAs (sg*Creb3l1*_A8) after 14 days of differentiation induction (D14) was also impaired (Fig. 4m). These observations implied that CREB3L1 deficiency diminished the synthesis of dentin matrix proteins.

### The deficiency of CREB3L1 altered chromatin accessibility and reduced gene expression associated with protein production and secretion

**Fig. 5:**
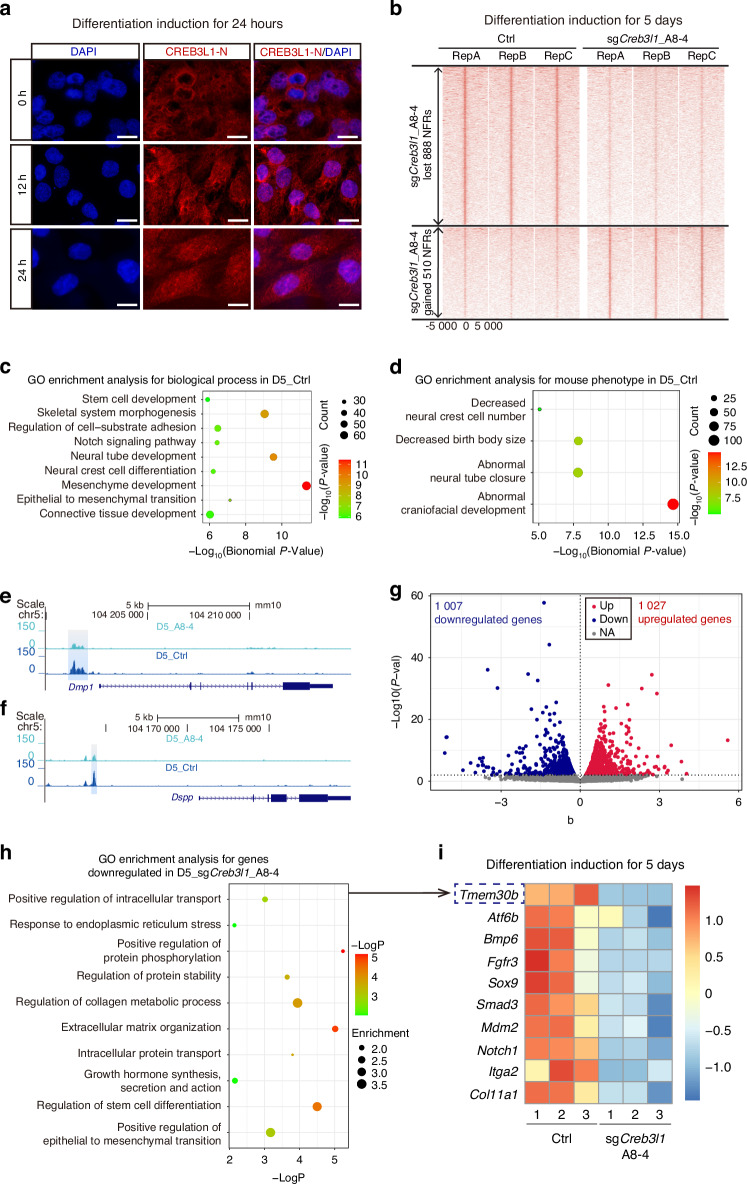
CREB3L1 deficiency led to substantial changes in chromatin accessibility and gene expression profile concerning protein production and secretion. **a** CREB3L1 acted as a transcriptional regulator by translocating to the nucleus during differentiation induction. Scale bar = 20 μm. **b** Heatmaps show the density of NFR summit-centered ATAC-seq signals in the Ctrl group and the sg*Creb3l1*_A8-4 group after 5 days of differentiation induction, respectively. The dot plot depicts the ATAC-seq outcomes, while the Gene Ontology (GO) enrichment analyses provided insight into the biological processes (**c**) and description of “Mouse Phenotype Single KO” (**d**) associated with the lost nucleosome-free regions (NFRs) in the sg*Creb3l1*_A8-4 group after 5 days of differentiation induction. **e**, **f** Visualization of ATAC-seq results for the Ctrl or sg*Creb3l1*_A8-4 cells after 5 days of differentiation induction from the UCSC Genome Browser. The blue box shows the closed NFRs in D5_sg*Creb3l1*_A8-4 near *Dmp1* and *Dspp*. **g** Scatter plots show the significantly up-regulated or down-regulated genes identified by bulk RNA-seq analysis in D5_sg*Creb3l1*_A8-4. **h** The dot plot displays the GO enrichment for the downregulated genes in D5_sg*Creb3l1*_A8-4 from the bulk RNA-seq results. **i** The heatmap visualizes the differential expression of genes in specific GO terms identified by the bulk RNA-seq analysis

The established function of CREB3L1 is acting as a transcription factor.^24^ To validate this, immunofluorescence staining of N-terminal forms of CREB3L1 (CREB3L1-N) was conducted during the differentiation induction of mDPCs to examine the nuclear translocation of CREB3L1. The CREB3L1-N were predominantly localized in the nucleus after 12 and 24 h of differentiation induction (Fig. 5a). To elucidate the mechanism behind the impaired differentiation capacity of CREB3L1-deficient mDPCs, ATAC-seq and RNA-seq analyses were performed in Ctrl and CREB3L1-deficient mDPCs on D5. A significant change in chromatin accessibility was observed, with 888 regions exhibiting decreased accessibility and 510 regions exhibiting increased accessibility in the CREB3L1-deficient group (Fig. 5b, Supplementary Table S2). Gene Ontology (GO) analysis for the genes near the closed chromatin regions revealed a significant impact on biological processes including stem cell development, skeletal morphogenesis, regulation of cell-substrate adhesion, Notch signaling pathway, neural tube development, neural crest cell differentiation, mesenchymal development, epithelial-to-mesenchymal transition, and connective tissue development (Fig. 5c). The “mouse phenotype single KO” descriptions of these genes were associated with decreased neural crest cell number, decreased birth size, abnormal neural tube closure and abnormal craniofacial development (Fig. 5d). Downregulation of CREB3L1 resulted in decreased chromatin accessibility at the regulatory regions near the *Dmp1* and *Dspp* genes (Fig. 5e, f).

Apart from the chromatin accessibility changes, the difference in gene expression was also investigated. RNA-seq revealed that 1 007 genes were downregulated and 1 027 genes were upregulated in the CREB3L1-deficient mDPCs (*P* < 0.01) (Fig. 5g, Supplementary Table S3). Furthermore, GO analysis of the downregulated genes in CREB3L1-deficient mDPCs revealed their association with several cellular processes, including positive regulation of intracellular transport, such as *Tmem30b*;^25–28^ response to ER stress, such as *Atf6b*,^29^ *Creb3l1*;^13^ positive regulation of protein phosphorylation, such as *Bmp6*,^30^ *Fgfr3*,^31,32^ *Sox9*;^33–35^ regulation of protein stability, such as *Smad3*,^36^ *Mdm2*;^37^ regulation of collagen metabolic process, such as *Notch1*,^38^ *Itga2*;^39,40^ extracellular matrix organization, such as *Col11a1*, an important component of collagen;^41^ intracellular protein transport, such as *Notch1*;^42^ growth hormone synthesis, secretion and action such as *Atf6b*,^29^ *Notch1*;^42^ regulation of stem cell differentiation, such as *Notch1*,^43^ *Sox9*;^33–35^ positive regulation of epithelial to mesenchymal transition, such as *Notch1*,^43^ *Smad3*^44^ (Fig. 5h, i). These findings are consistent with the reported role of CREB3L1 in ER stress, regulating transporter and extracellular matrix protein levels.^45,46^ These observations suggest that CREB3L1 altered chromatin accessibility and downregulated the expression of genes related to cell differentiation, protein biosynthesis, and protein secretion.

### The CREB3L1-deficient mDPCs exhibited a weakened capacity for protein biosynthesis and secretion

**Fig. 6:**
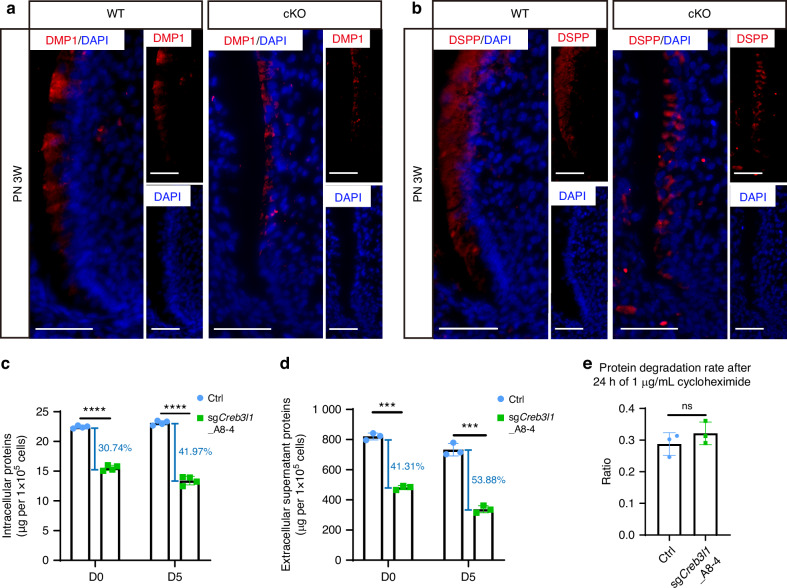
Lack of CREB3L1 reduced the protein biosynthesis and export. **a** The DMP1 expression in odontoblasts of the first molar significantly decreased in PN 3 W cKO mice. Scale bar = 50 μm. **b** The DSPP expression in odontoblasts of the first molar significantly decreased in PN 3 W cKO mice. Scale bar = 50 μm. **c** The total amount of intracellular proteins and the percentage of decline to the Ctrl group. **d** The total quantity of extracellular supernatant proteins and the percentage of decline to the Ctrl group. **e** The protein degradation rates were measured after 24 h of exposure to 1 μg/mL cycloheximide in both the Ctrl and sg*Creb3l1*_A8-4 groups. *n* ≥ 3. ns not significant, *P* > 0.05; ***P < 0.001; ****P < 0.000 1

Inspired by the top GO enriched term, we asked if loss of CREB3L1 leads to weakened capacity for protein biosynthesis and secretion. First, we chose to analyze marker protein for odontoblast terminal differentiation, DMP1 and DSPP. Consistent with the downregulation of RNA level for *Dmp1* and *Dspp* in vitro and in vivo, the protein levels of DMP1 (Fig. 6a) and DSPP (Fig. 6b) in the odontoblasts of cKO mice were significantly lower than that of WT mice. Since the downregulation of DMP1 and DSPP protein may be due to the inhibition of transcription, we further investigate protein production in the knockout clone. The quantities of intracellular protein and supernatant secreted protein were compared between the Ctrl and sg*Creb3l1*_A8-4 groups. A significant reduction (30.74% in D0 and 41.97% in D5) in the quantity of intracellular proteins was observed in CREB3L1-deficient mDPCs (Fig. 6c). Notably, a more severe decrease (41.31% in D0 and 53.88% in D5) in the amount of cellular supernatant proteins was detected in CREB3L1-deficient mDPCs (Fig. 6d).

The reduction in total protein levels could be due to a reduced ability to produce protein or an increase in degradation. To determine whether the rate of protein degradation varied after inhibition of protein synthesis, cycloheximide (CHX)^47^ was applied to inhibit protein synthesis in the Ctrl and sg*Creb3l1*_A8-4 groups. Through a gradient concentration screen, it was determined that the application of 1 ug/mL CHX affected cellular protein synthesis with less cell death (Supplementary Fig. S9). Both Ctrl and sg*Creb3l1*_A8-4 groups were treated with this concentration of CHX for 24 h and the protein degradation rates before and after treatment were compared between the two groups. No significant difference was observed in the degradation rate between the two groups (Fig. 6e). These observations support the sequencing results that downregulation of CREB3L1 leads to a diminution of protein production and secretion.

### CREB3L1 deficiency repressed TMEM30B expression in odontoblasts

**Fig. 7:**
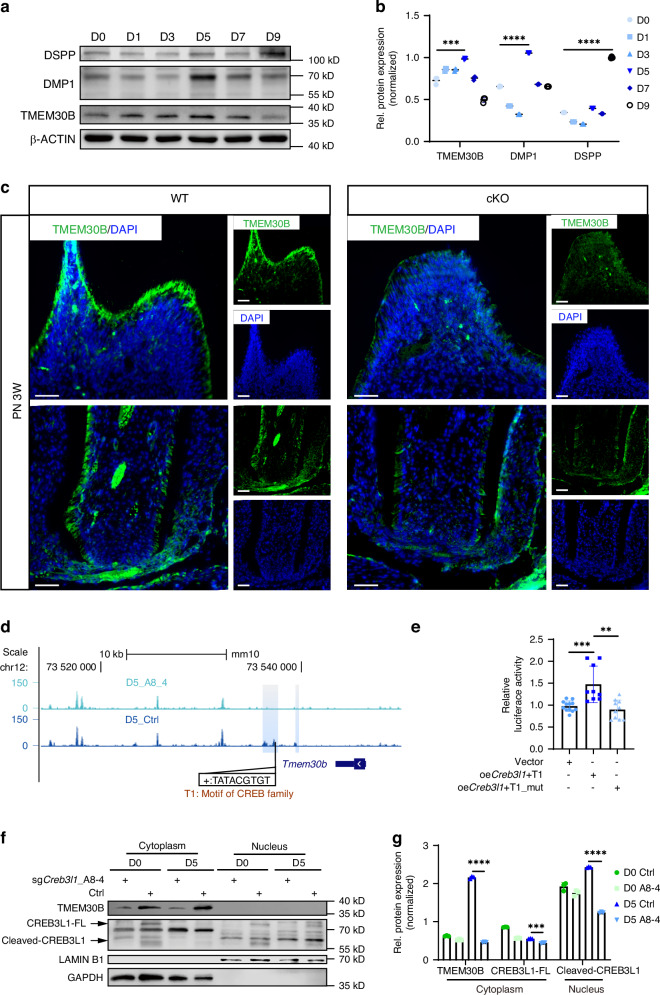
TMEM30B was identified to be regulated by CREB3L1 in odontoblasts. **a** The expression levels of TMEM30B protein significantly increased during the differentiation induction of mDPCs. **b** Quantification of the relative protein expression levels of **a**. **c** The expression of TMEM30B in odontoblasts of the first molar underwent a significant downregulation in PN 3 W cKO mice. **d** UCSC genome browser tracks demonstrate ATAC-seq peaks located at the *Tmem30b* locus. The blue box displays regions with closed chromatin in D5_sg*Creb3l1*_A8-4 near *Tmem30b*, while the black box exhibits the CREB family binding motif situated in the potential regulatory element region of *Tmem30b*. **e** Bar graph displays the results of the dual luciferase reporter assay. **f** The expression of the protein TMEM30B declined in the sg*Creb3l1*_A8-4 group after 5 days of differentiation induction. TMEM30B demonstrated predominant cytoplasmic expression. **g** Quantification of the relative protein expression levels of **f**. *n* ≥ 3. **P < 0.01; ***P < 0.001; ****P < 0.000 1

In addition to its role as a transcription factor regulating the synthesis of mineralization-associated proteins, we are particularly interested in understanding how CREB3L1 regulates the protein secretion pathway. The BETA tool^48^ was utilized to identify the downregulated genes whose potential regulatory elements were also closed in ATAC-seq (Supplementary Tables S4, 5). To understand the role of TMEM30B, we initially confirmed its expression during the differentiation induction process. Its expression pattern was parallel to that of CREB3L1, peaking at D5 in mDPCs (Fig. 7a, b). Similarly, its expression pattern in dental papilla is similar to that of CREB3L1 in vivo (Supplementary Fig. S10). Additionally, in vivo histological analysis of the first molar from PN 3 W cKO mice revealed a significant reduction in TMEM30B expression levels (Fig. 7c).

To determine if CREB3L1 can directly regulate *Tmem30b* transcription, a CREB family motif within *Tmem30b* located in the sg*Creb3l1*_A8-4 lost NFR was identified by JASPR website (Fig. 7d). Then, the corresponding region together with the regulatory element was cloned into the pGL3 promoter plasmid (T1) and the region without the motif (T1_mut). Dual-luciferase assays were conducted on the vector group, overexpressed *Creb3l1* (oe*Creb3l1*)+T1 group, and oe*Creb3l1* + T1_mut group. The results showed that overexpression of CREB3L1 could significantly upregulate the luciferase activity driven by the regulatory element of *Tmem30b*. However, the luciferase activity of the mutated *Tmem30b* regulatory element (oe*Creb3l1* + T1_mut) group was downregulated compared to wildtype (oe*Creb3l1* + T1) group. Thus, CREB3L1 is a positive regulator of this motif fragment (Fig. 7e).

The expression levels of TMEM30B protein were also detected during the 5-day differentiation induction of Ctrl and sg*Creb3l1*_A8-4 groups. As shown by the analysis of the isolated cytoplasm and nucleus, TMEM30B expression was exclusively observed in the cytoplasm and demonstrated a significant decrease in sg*Creb3l1*_A8-4 groups (Fig. 7f, g). Taken together, these observations suggest that TMEM30B is expressed in odontoblasts and regulated by CREB3L1.

### TMEM30B deprivation impaired the protein synthesis and secretion capability of mDPCs

**Fig. 8:**
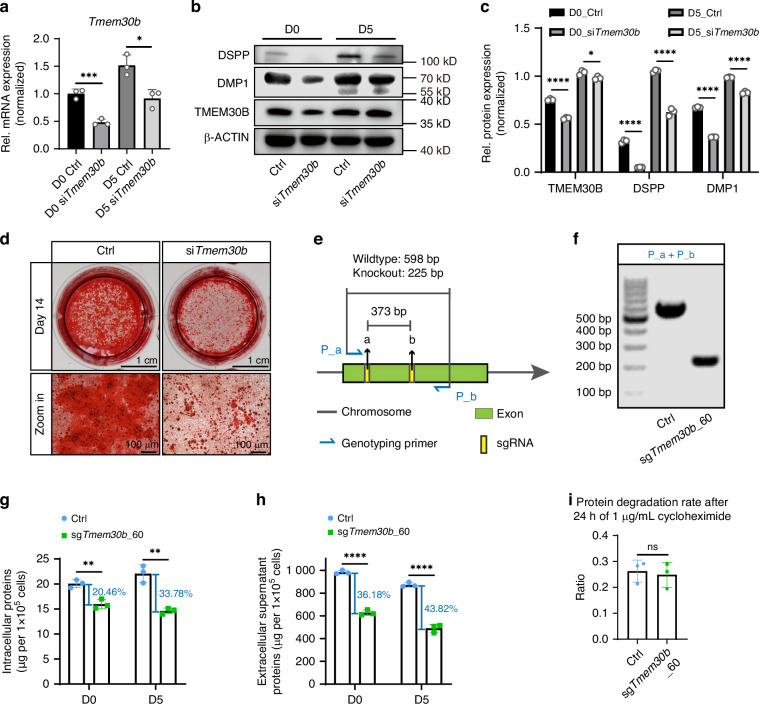
TMEM30B deficiency impaired the odontoblastic differentiation capacity of mDPCs and reduced their intracellular and supernatant protein levels. **a** The mRNA expression level of *Tmem30b* was down-regulated in the si*Tmem30b* group compared to the Ctrl group. **b** After 5 days of differentiation induction, the expression of TMEM30B, DMP1, and DSPP was reduced in mDPCs treated with si*Tmem30b* when compared to the Ctrl group. **c** Quantification of the relative protein expression levels of **b**. **d** Alizarin red S staining was performed to observe the calcium nodules in both Ctrl and si*Tmem30b* groups after 14 days of differentiation induction. Scale bar = 1 cm, scale bar = 100 μm. **e** Design of sgRNAs for *Tmem30b* knockout. **f** Genotyping results of the homozygous knockout cell line, sg*Tmem30b_60*. **g** The total amount of intracellular proteins and the percentage of decline to the Ctrl group. **h** The total quantity of extracellular supernatant proteins and the percentage of decline to the Ctrl group. **i** The protein degradation rates were measured after 24 h of treatment with 1 μg/mL cycloheximide in both the Ctrl and sg*Tmem30b*_60 groups. *n* = 3. ns, not significant, *P* > 0.05; *P < 0.05; **P < 0.01; ***P < 0.001; ****P < 0.000 1

To examine the impact of TMEM30B deficiency on mDPC differentiation, *Tmem30b* expression was inhibited by siRNA (Fig. 8a). The suppression of *Tmem30b* resulted in a significant reduction in TMEM30B, DMP1, and DSPP protein levels during the differentiation process (Fig. 8b, c). Moreover, inhibiting *Tmem30b* greatly decreased the formation of mineralized nodules (Fig. 8d).

Targeted knockout of *Tmem30b* was performed using a pair of sgRNAs (sgRNA_a and sgRNA_b) (Fig. 8e) to evaluate the impact of *Tmem30b* deficiency on intracellular and supernatant proteins during differentiation induction. The P_a + P_b primers (Supplementary Table S6) were utilized to identify the wild-type (598 bp) and knockout (225 bp) chromosome (Fig. 8e). Subsequently, a homozygous knockout monoclone, sg*Tmem30b*_60, was selected for further experiments (Fig. 8f). Initially, the knockout of *Tmem30b* significantly reduced the mRNA expression of *Tmem30b* and *Dmp1* (Supplementary Fig. S11a) and decreased the protein level of DMP1 and DSPP (Supplementary Fig. S11b, c) during differentiation induction. Moreover, sg*Tmem30b*_60 also hindered the formation of mineralized nodules after 14 days of differentiation induction, as evidenced by Alizarin red S staining (Supplementary Fig. S11d). The levels of intracellular and secreted proteins were examined in TMEM30B-deficient cells. The results indicated a marked reduction in intracellular proteins (20.46% in D0 and 33.78% in D5) and an even greater decrease in supernatant proteins (36.18% in D0 and 43.82% in D5) in the sg*Tmem30b*_60 group (Fig. 8g, h).

Similarly, the protein degradation rates before and after CHX treatment were compared between the Ctrl and sg*Tmem30b*_60 groups by treating both groups with 1 ug/mL CHX for 24 h. No significant difference in the rate of degradation was observed (Fig. 8i). The results suggest that the absence of *Tmem30b* results in impaired odontoblastic differentiation ability of mDPCs and reduced protein synthesis and secretion.

### Overexpression of TMEM30B in CREB3L1-deficient mDPCs partially rescued protein secretion ability

**Fig. 9:**
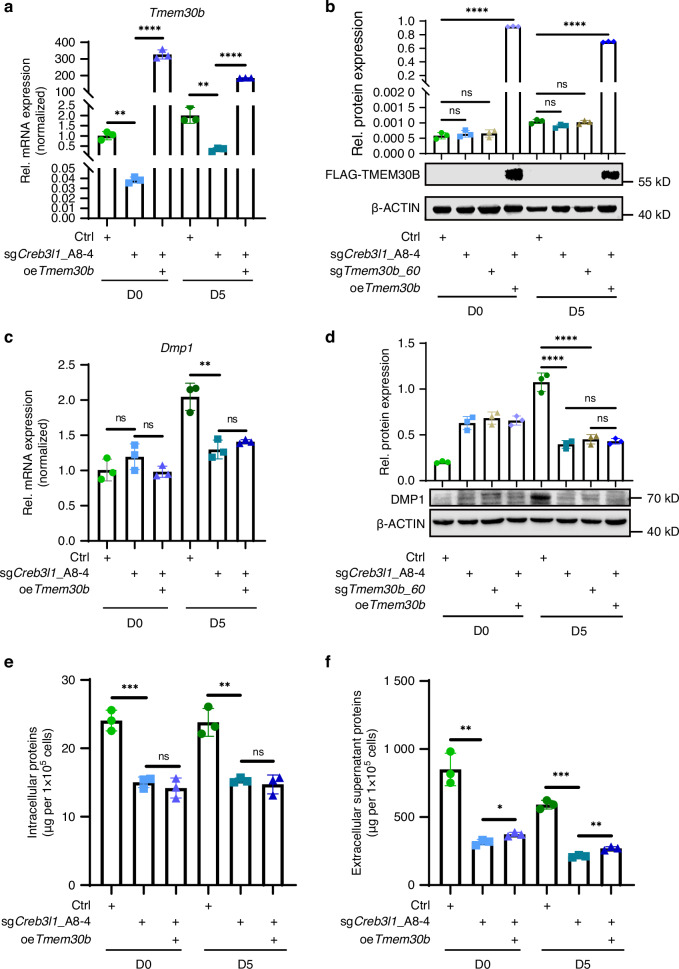
Overexpression of TMEM30B in CREB3L1-deficient mDPCs partially rescued the ability to export the protein. **a** The mRNA expression level of *Tmem30b* increased in the sg*Creb3l1*_A8-4 group treated with overexpression lentivirus *Flag-Tmem30b* (oe*Tmem30b*) during 5 days of differentiation induction. **b** FLAG-TMEM30B protein level was upregulated in sg*Creb3l1*_A8-4 treated with oe*Tmem30b* group during 5 days of differentiation induction. **c** The mRNA expression level of *Dmp1* remained unchanged between the sg*Creb3l1*_A8-4 group and the sg*Creb3l1*_A8-4 treated with oe*Tmem30b* group after 5 days of differentiation induction. **d** The protein level of DMP1 was unaltered between the sg*Creb3l1*_A8-4 group and the sg*Creb3l1*_A8-4 treated with oe*Tmem30b* group after 5 days of differentiation induction. **e** Quantification of intracellular protein was performed in three groups, Ctrl, sg*Creb3l1*_A8-4, and the sg*Creb3l1*_A8-4 treated with oe*Tmem30b*. **f** Quantification of culture supernatant protein was conducted in three groups, Ctrl, sg*Creb3l1*_A8-4, and the sg*Creb3l1*_A8-4 treated with oe*Tmem30b* group. *n* = 3. ns not significant, *P* > 0.05; *P < 0.05; **P < 0.01; ***P < 0.001; ****P < 0.000 1

TMEM30B was overexpressed in CREB3L1-deficient cells to investigate whether overexpressing TMEM30B rescues protein synthesis and secretion function. The mRNA level of *Tmem30b* increased upon the infection with *Flag-Tmem30b* lentivirus (oe*Tmem30b*) during differentiation induction (Fig. 9a). The levels of FLAG-TMEM30B were also elevated (Fig. 9b). *Dmp1* levels were not rescued by oe*Tmem30b* in the sg*Creb3l1*_A8-4 group compared to the sg*Creb3l1*_A8-4 group after a 5-day differentiation induction (Fig. 9c). Additionally, oe*Tmem30b* did not rescue the protein levels of DMP1 (Fig. 9d) and DSPP (Supplementary Fig. S12a, b) on D5. Likewise, the capacity to form mineralized nodules was also not restored (Supplementary Fig. S12c). Although oe*Tmem30b* cannot rescue the intracellular proteins (Fig. 9e), it can partially rescue the extracellular supernatant proteins in sg*Creb3l1*_A8-4 mDPCs during 5-day differentiation induction (Fig. 9f).

The reduction in mineralization-associated proteins (such as DMP1 and DSPP) in CREB3L1-deficient cells is not attributable to a shortage of TMEM30B. Thus, we propose that the upregulation of CREB3L1 in TMEM30B-deficient cells is likely to enhance the production of these proteins. CREB3L1 overexpression (oe*Creb3l1*) was performed on TMEM30B-deficient mDPCs (sg*Tmem30b*_60). *Creb3l1* mRNA levels showed a large increase in the oe*Creb3l1* treated group (Supplementary Fig. S13a). Consistent with the hypothesis, the overexpression group exhibited strongly upregulated expression of CREB3L1-FL, Cleaved-CREB3L1, DMP1, and DSPP (Supplementary Fig. S13b, c).

Overall, the deficiency of CREB3L1 in mDPCs leads to reduced protein levels both intracellularly and extracellularly. The reduction in intracellular protein is due to the transcriptional downregulation of *Dmp1*, *Dspp*, and other differentiation-related genes, while the decrease in extracellular protein is partially attributed to TMEM30B deficiency.

## Discussion

Tooth development is regulated by intricate and well-coordinated molecular networks.^2,49^ In this study, *Creb3l1* was deleted in the neural crest lineage by using *Wnt1-Cre*.^50–52^ *Wnt1-Cre; Creb3l1*^*f/f*^ conditional knockout mice exhibited deficiencies in both root elongation and dentin deposition. Subsequent investigations revealed that CREB3L1 primarily mediates transcriptional regulation of ECM proteins production and secretion. Deficiency of CREB3L1 in mDPCs caused decreased levels of intracellular and extracellular proteins, and the decline in extracellular proteins was partially attributed to the deprivation of TMEM30B.

Our RNAscope data were consistent with previous studies, indicating that CREB3L1 is primarily expressed in polarized, secretory, and mature odontoblasts,^53^ as well as in root odontoblasts,^54^ suggesting that CREB3L1 may participate in dentinogenesis. Previous findings reported that CREB3L1 was implicated in the terminal differentiation of osteoblasts, and CREB3L1 deficient mice exhibited severe bone loss.^55,56^ In this study, *Wnt1-Cre; Creb3l1*^*f/f*^ (cKO) mice exhibited a reduction in the height of mandibular bone below the first molar root furcation in comparison to the WT mice. A subset of cKO mice exhibited a reduction in mandibular bone mass compared to WT mice. However, the reduction was not as pronounced as that observed in global knockout mice. It is speculated that this may be due to the compensatory effect of cells from other subsequent lineage origins on the jawbone phenotype, but this requires further research to confirm this conjecture. It is also possible that the penetrance rate in conditional knockout mice is lower than in global knockout mice. A previous study reported that only 64% of *Wnt1-Cre; Fgf18*^*c/c*^ mice exhibited the cleft palate phenotype, which was lower than that of *Fgf18*^*−/−*^ mice.^57^

In addition to the observed phenotype in the mandible, the cKO mice exhibited shorter roots and thinner dentin, in comparison to the WT mice. The crown dentin thickness of PN 3 W cKO mice decreased compared to WT mice, but no statistically significant difference was observed. However, a statistically significant difference was identified in the decrease in root dentin thickness. With continued deposition of dentin in the crown and root until 8 weeks of age, cKO mice exhibited a more notable reduction in dentin thickness in both crown and root dentin thickness than WT mice. The length of the root has a significant impact upon the long-term viability of the dentition.^58^ Abnormal molecular signaling during root development can result in dentin dysplasia. The phenotype of dentin dysplasia type I encompasses thinner dentin and shorter root malformations. These abnormalities may be associated with early tooth loss.^59^ The phenotype resulting from CREB3L1 deficiency is evident in bone development, but the impact on tooth development is relatively mild. It may be due to the fact that tooth development is regulated by multiple transcription factor regulatory networks.^2^ In a similar vein, it was previously reported that the deletion of the Indian hedgehog (*Ihh*) in the *Wnt1*+ lineage resulted in skeletal malocclusion. Nevertheless, no notable alterations in the teeth or dentition were discerned.^60^ These phenomena may be attributed to the existence of a robust compensatory mechanism governing tooth development, which deserves further investigation.

CREB3L1 possesses a bZIP domain^61^ suggesting that CREB3L1 can repress or activate its target genes. Our results demonstrated that deficiency of CREB3L1 significantly decreased the expression of ECM proteins DMP1 and DSPP, and impaired the ability of mDPCs to differentiate into odontoblasts and form mineralized nodules. ATAC-seq and RNA-seq analyses were subsequently used to explore the function of CREB3L1 in epigenetic and transcriptional regulation. The closed regions in CREB3L1-deficient mDPCs were analyzed to be involved in the development of the connective tissue and skeletal system, consistent with previous studies in global knockout mice,^55,56^ It is noteworthy that deficiency of CREB3L1 was predicted to exhibit abnormal craniofacial development, which supports our in vivo observations of tooth and bone phenotypes.

In terms of transcriptional regulation, CREB3L1 deficiency downregulated genes associated with response to ER stress and protein production. Consistent with previous reports that CREB3L1 functioned as a transducer of endoplasmic reticulum stress and was involved in the proper synthesis of proteins.^24^ In addition to the direct transcriptional regulation of *Dmp1* and *Dspp*, sequencing data and in vitro studies have now provided new evidence that CREB3L1 downregulation significantly affects protein secretion. This perspective is a complement to previous studies that have focused primarily on transcriptional regulation or chromatin accessibility modification during odontoblastic differentiation.^9,62,63^

CREB3L1 functions primarily as a transcription factor, regulating downstream target gene expression at the transcriptional level to facilitate their functions. Therefore, its modulation of protein secretion may occur indirectly through specific proteins that participate in protein transport. Combining the ATAC-seq and RNA-seq data, TMEM30B was identified as a potential downstream target of CREB3L1. TMEM30B, also known as CDC50B belongs to the cell division control protein (CDC) family.^25^ The CDC50 family was predicted to be located primarily at the endoplasmic reticulum, Golgi, or plasma membrane in the cytoplasm and has been reported to be involved in the function of lipid transport activity.^26,64^ In our study, TMEM30B was also exclusively located in the cytoplasmic compartment in the odontoblasts. The absence of TMEM30B impaired the secretion of extracellular matrix proteins and suppressed the mineralization capacity of odontoblasts, implying that it may be responsible for the protein export. This was confirmed that overexpression of TMEM30B partially rescued the extracellular supernatant proteins in CREB3L1-deficient mDPCs. The reduction of intracellular protein levels attributed to TMEM30B deficiency is probably a consequence of decreased protein transportation. This hypothesis necessitates additional exploration. Overexpression of TMEM30B in CREB3L1-deficient cells did not rescue DMP1 and DSPP expression, whereas overexpression of CREB3L1 in TMEM30B-deficient cells rescued DMP1 and DSPP expression, indicating that CREB3L1-induced reduction of DMP1 and DSPP is not caused by TMEM30B. These findings demonstrate that TEME30B served as a novel molecule for protein secretion, and a downstream target of CREB3L1, which was also necessary for odontoblastic differentiation.

Several limitations are present in this study. The first is the lack of in vivo experiments to evaluate the efficiency of protein synthesis in WT and cKO mice. The provided information only described the changes in the amount of proteins present in either extracellular supernatant or intracellular proteins but does not specifically elucidate which proteins have been altered. Further studies are required to understand how TMEM30B mediates protein secretion in mDPCs.

In summary, CREB3L1 participates the dentinogenesis, and regulated the protein biosynthesis directly and protein secretion indirectly. The compromised ability of protein secretion caused by CREB3L1 deficiency is partially ascribed to the deprivation of TMEM30B.

## Materials and methods

### Animals husbandry

C57BL/6 wildtype (WT) mice were purchased from the Hubei Provincial Center for Disease Control and Prevention (Hubei CDC). E15.5, E18.5, PN0.5, PN2.5, PN 3 W, and PN 8 W) mice were collected for subsequent experiments.

*Creb3l1*^*f/f*^ mice bearing loxP sites flanking in introns 2 and 4 of the *Creb3l1* (Stock No: T019448) were generated by GemPharmatech Co. Ltd. using the CRISPR/Cas9 system. The *Wnt1-cre* mice (Stock NO. N000275) were obtained from the Jackson Laboratory. *Creb3l1*^*f/f*^ mice were crossed with *Wnt1-cre* mice to generate *Wnt1-cre; Creb3l1*^*f/w*^ mice. Then by mating *Wnt1-cre; Creb3l1*^*f/w*^ mice with *Creb3l1*^*f/f*^ mice, the obtained *Wnt1-cre; Creb3l1*^*f/f*^ (cKO) mice were used as the CREB3L1 deficient group, and the *Creb3l1*^*f/f*^ and *Creb3l1*^*f/w*^ littermates were used as the control group. The genotype of the transgenic mice was identified by conventional PCR analysis of genomic DNA extracted from mouse tails. For the floxed *Creb3l1* allele: F-primer, 5’-CTGTGCTCATGCCAACACACA-3’, and R-primer, 5’-TGCAAGAACAGCCAGCAGTCT-3’, the product size of wild type *Creb3l1* was 291 bp, and the mutant was 395 bp. For the *Wnt1-Cre* transgene: F-primer, 5’- GCCTGCATTACCGGTCGATGC-3’, and R-primer, 5’- CAGGGTGTTATAAGCAATCCC-3’, the product size of *Wnt1-Cre* transgene was 481 bp. All mice used in this study were C57BL/6 background strain and were bred and maintained under specific pathogen-free (SPF) conditions at the State Key Laboratory Breeding Base of Basic Science of Stomatology, Hubei Province & the Key Laboratory of Oral Biomedicine (Wuhan University), Ministry of Education (Hubei-MOST KLOS & KLOBM). Both female and male mice were included in the analysis. All mouse related experiments were performed in accordance with the guidelines and approved by the Institutional Animal Care and Use Committees at the School and Hospital of Stomatology of Wuhan University (protocol no. S07923090B).

### RNAscope in situ hybridization

RNAscope 2.5 HD Reagent Kit-RED (Cat No. 322350; Advanced Cell Diagnostics, Newark, CA, USA) was used to detect *Creb3l1* mRNA expression in situ in paraffin sections of E15.5, E18.5, and PN 2.5 mice molar according to the manufacturer’s instructions. The *Creb3l1* probe (Cat No. 585951; Advanced Cell Diagnostics, Newark, CA, USA)*, Dmp1* probe (Cat No. 441171), *Dspp* probe (Cat No. 448301), negative probe (Cat No. 310043), and positive probe (Cat No. 313911) were purchased from Advanced Cell Diagnostics.

### Micro-computed tomography and histomorphometric analyses

The mandibles harvested from the PN 8 W *Creb3l1*^*f/f*^ *and Wnt1-cre; Creb3l1*^*f/f*^ mice, were fixed immediately in the 4% paraformaldehyde (PFA; Biosharp, Hefei, China) at 4 °C for 24 h and then the residual paraformaldehyde was removed through running water. The mandibles of each genotype (*n* ≥ 5) were scanned via a Micro-CT system (SkyScan1276, Bruker, Belgium). Then we used the NRecon (Bruker) to reconstruct the mandibles. Mimics 19.0 was utilized to complete the virtual reconstruction. The Dataviewer software (Bruker) was utilized to adjust the three-dimensional orientations of the first molar assuring that each first molar presented a similar two-dimensional orientation, and to measure the thickness of dentin, the length of crown and root. To evaluate the bone density, the regions of interest (ROI) in the mandibles were limited to the crown dentin or root dentin, and the quantitative analysis was calculated by CTAn (Bruker).

### Immunofluorescence staining (IF) of tissues and cells

The mandibles collected from PN 0.5 or PN 3 W mice were fixed individually in 4% paraformaldehyde (PFA; Biosharp, Hefei, China) overnight at 4 °C and decalcified in 10% EDTA (pH 7.4) for 3 days or 4 weeks. Then the samples were dehydrated through a graded ethanol series and embedded in paraffin. Sagittal sections of mandibles were cut at 6 μm thickness using a Leica RM2265 paraffin microtome and then placed in microscope slides. To make an accurate comparison among groups, a uniform standard was developed. For molars, sections with the largest dental pulp and visible apical foramen were chosen. The sections were deparaffinized in the xylene, rehydrated using descending grades of ethanol solutions, and then rinsed in phosphate-buffered saline (PBS) for 3 × 5 min. For antigen retrieval, we immersed the sections in 10 mM citrate buffer (pH 6.0) and then used a microwave to heat the sections for 15 min followed by cooling to room temperature. Cells on coverslips were fixed with 4% paraformaldehyde (PFA; Biosharp, China) for 10 min at room temperature. The cells were treated with TritonX-100 to increase membrane permeability. Tissue slides or cells were blocked with 2.5% bovine serum albumin solution (BSA; Sigma-Aldrich, St Louis, MO, United States) at 37 °C for 60 min and incubated with primary antibodies at 4 °C overnight. The primary antibodies included anti-CREB3L1-N (F-8) (sc-514635, 1:50, Santa, CA, USA) or anti-TMEM30B (ab185944, 1:100, Abcam, Cambridge, UK). Then Alexa Fluor 594 or 488 -conjugated secondary antibody (ANT029, ANT024, 1:200, AntGene, Wuhan, China) was added to the sections for 1 h at 37 °C. Then slides were rinsed in PBS for 3 × 5 min. For single immunofluorescence staining, samples were counterstained with 4’,6-diamidino-2-phenylindole (DAPI; ZSGB-BIO, Beijing, China) for visualization of the nuclei.

The kit with tyramide signal amplification technology (RC0086-23RM, Record Biological Technology, Shanghai, China) was also utilized to perform single or double IF staining. The primary antibodies included anti-DMP1 (3844-100,1:100, BioVision, CA, USA), anti-DSPP (bs-8557R, 1:200, Bioss, Beijing, China), anti-CREB3L1 (11235-2-AP, 1:100, Proteintech, Wuhan, China), or anti-TMEM30B (ab185944, 1:100, Abcam, Cambridge, UK). Finally, the sections were observed and photographed using a fluorescence microscope (Carl Zeiss, Oberkochen, Germany).

### Quantification of intracellular and extracellular supernatant proteins

The intracellular proteins were collected, and the volume and the number of cells were recorded. The extracellular supernatant proteins were harvested from the cell culture supernatant and the volume was recorded. The intracellular and extracellular supernatant protein concentrations were measured and standardized using a BCA Protein Assay Kit (23225, Thermo Fisher Scientific, Rockford, Illinois, USA). The protein mass was obtained by multiplying the protein concentration by the volume.

### Treatment with protein synthesis inhibitor

Equal amount of mDPCs-Cas9, sg*Creb3l1*_A8-4, and sg*Tmem30b*_60 cells were cultured in 12-well plates. Cycloheximide (CHX) (Sigma-Aldrich, St Louis, MO, USA) was dissolved in DMSO. When CHX was applied, the culture medium was removed from the culture dish and complete culture medium with CHX solution was added to the dish (the final concentration, 1 μg/mL). The initial cells without CHX served as control. The cells treated with CHX for 24 h were used as the experimental group. Total protein from all groups was collected for cell counting and measuring the concentration to obtain the final total amount of protein.

### The detailed protocols of the following experiments are provided in the Supplementary materials and methods

- Hematoxylin and eosin staining (HE)
- Immunohistochemistry (IHC)
- Scanning electron microscope (SEM)
- Cell culture and differentiation induction
- Western blot analysis
- Total RNA isolation and quantitative reverse transcription PCR assay
- Alizarin Red S Staining
- Assay for transposase-accessible chromatin with high-throughput sequencing (ATAC-seq) library preparation
- Analysis of ATAC-seq library
- RNA-Seq Library Generation and Data Analysis
- Binding and Expression Target Analysis (BETA)
- siRNA transfection
- Overexpression of *Flag-Tmem30b* and *Flag-Creb3l1*
- Dual luciferase activity assay
- Statistical analysis

## Supplementary information

Supplementary information

## Data Availability

The datasets generated in the current study are available in the GSA repository, accession number: CRA013369. The authors are exclusively responsible for the content of this article and datasets collected to support the findings of this study. The data for this study are available from the corresponding author upon reasonable request.
