# Supplementary material for: CREB3L1 deficiency impairs odontoblastic differentiation and molar dentin deposition partially through the TMEM30B
Source: Int J Oral Sci. 2024 Oct 10;16:59. doi: 10.1038/s41368-024-00322-y (PMC11464721; doi:10.1038/s41368-024-00322-y)
Supplement: Supplementary file 1 — Supplementary information [file 41368_2024_322_MOESM1_ESM.pdf]

Supplementary figures

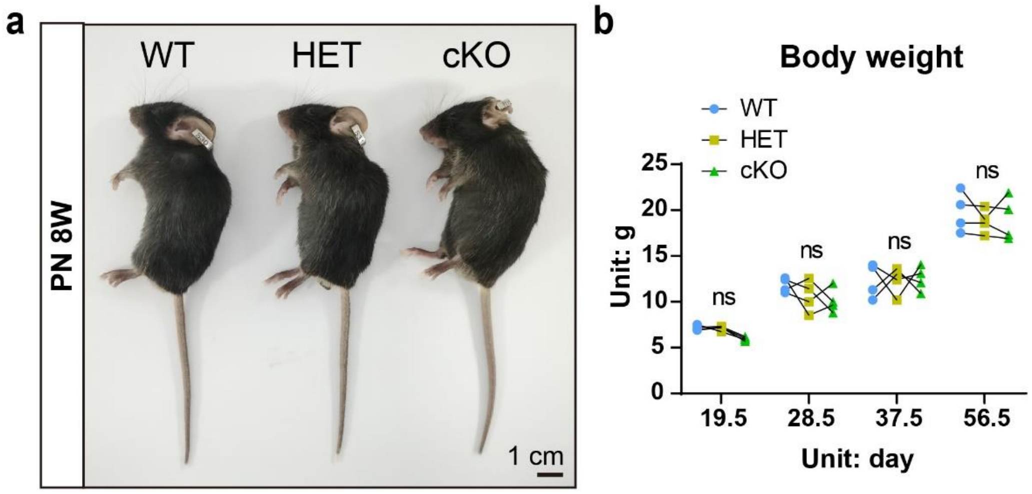

Fig. S1 No significant differences in body size or weight were observed among WT, heterozygous mice (HET), and cKO mice. n = 4. ns, not significant, $p > 0.05$.

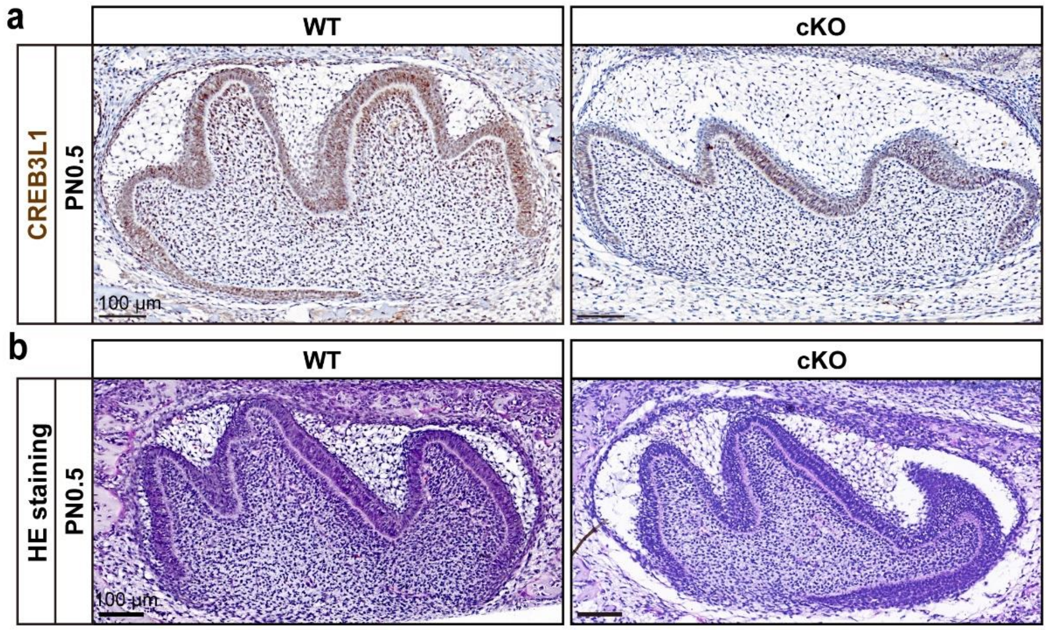

Fig. S2 Conditional deletion of *Creb3l1* in the neural crest lineage revealed no difference in the first molar of PN0.5 mice. **a** The absence of CREB3L1 in cKO

mice odontoblasts was validated by IHC staining in mandibular first molar sections of PN0.5 mice. Scale bar = 100 μm. **b** HE staining was performed on the first molar of PN0.5 WT and conditional knockout mice. Scale bar = 100 μm.

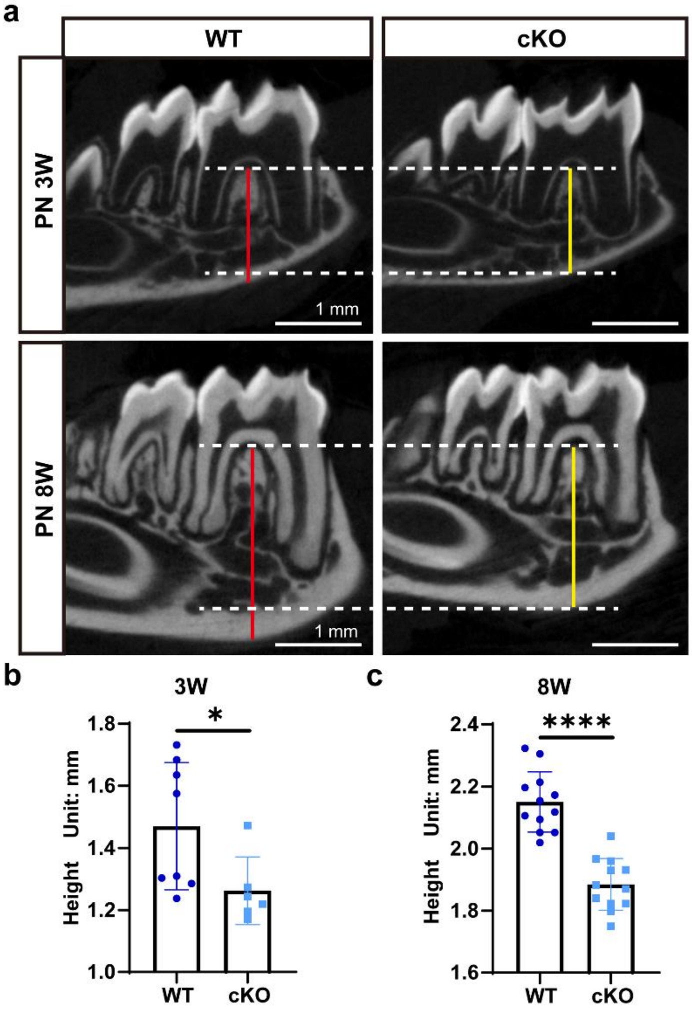

Fig. S3 The height of the mandible below the root furcation of the first molar was slightly decreased in cKO mice compared to WT mice. **a** The schematic diagram depicts the measurement site for the height of the mandible below the root furcation of the first molar. The vertical distance from the root furcation alveolar ridge to the outermost part of the mandibular bone cortex was quantified in the same section by adjusting the mandible to the same orientation. The red and yellow solid lines illustrate the height of the mandible below the

first molar root furcation in WT and cKO mice, respectively. Scale bar = 1 mm. **b** The quantitative data for the height of the mandible below the root furcation of the first molar in WT and cKO mice at PN 3W. **c** The quantitative data for the height of the mandible below the root furcation of the first molar in WT and cKO mice at PN 8W. n ≥ 6. *p < 0.05; ****p < 0.0001.

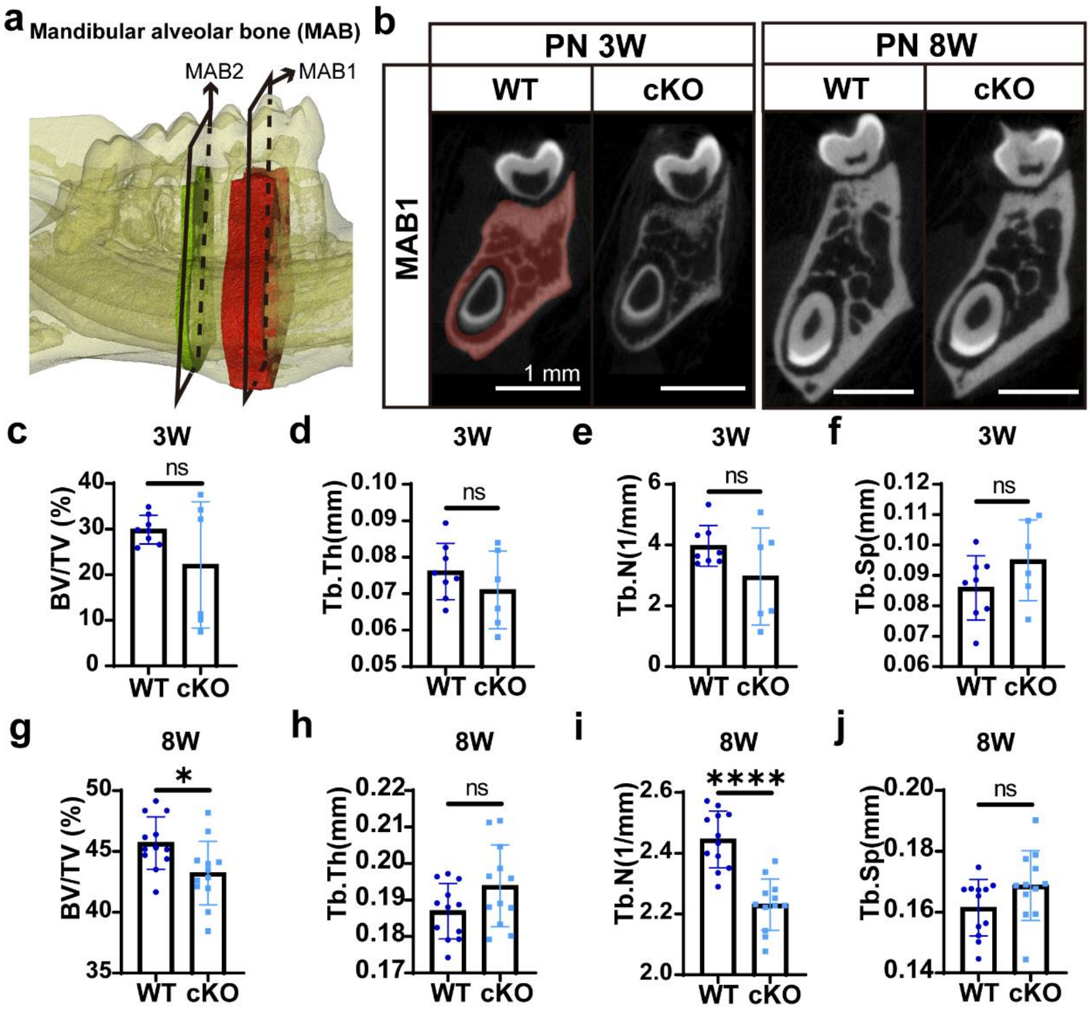

Fig. S4 Micro-CT analysis of mandibular alveolar bone (MAB) from PN 3W and PN 8W conditional knockout (cKO) and wild-type (WT) mice. **a** The schematic diagram of the measurement sites for MAB1. The red 3D area (MAB1) represents the mandibular alveolar bone in the region between the two roots of the first molar. The green 3D area (MAB2) represents the mandibular alveolar

bone between the first and second molars. **b** Micro-CT sections of MAB1 from WT and cKO mice at PN 3W and PN 8W. Scale bar = 1 mm**. c** The quantitative data of the bone volume (BV) to tissue volume (TV) from WT and cKO mice MAB1 at PN 3W. **d** The quantitative data of the trabecular thickness (Tb.Th) from WT and cKO mice MAB1 at PN 3W. **e** The quantitative data of the trabecular number (Tb.N) from WT and cKO mice MAB1 at PN 3W. **f** The quantitative data of the trabecular separation (Tb.Sp) from WT and cKO mice MAB1 at PN 3W. **g–j** The quantitative data of the BV/TV (**g**), Tb.Th (**h**), Tb.N (**i**), Tb.Sp (**j**) from WT and cKO mice MAB1 at PN 8W. n ≥ 6. ns, not significant, $p > 0.05$; *$p < 0.05$; ****$p < 0.0001$.

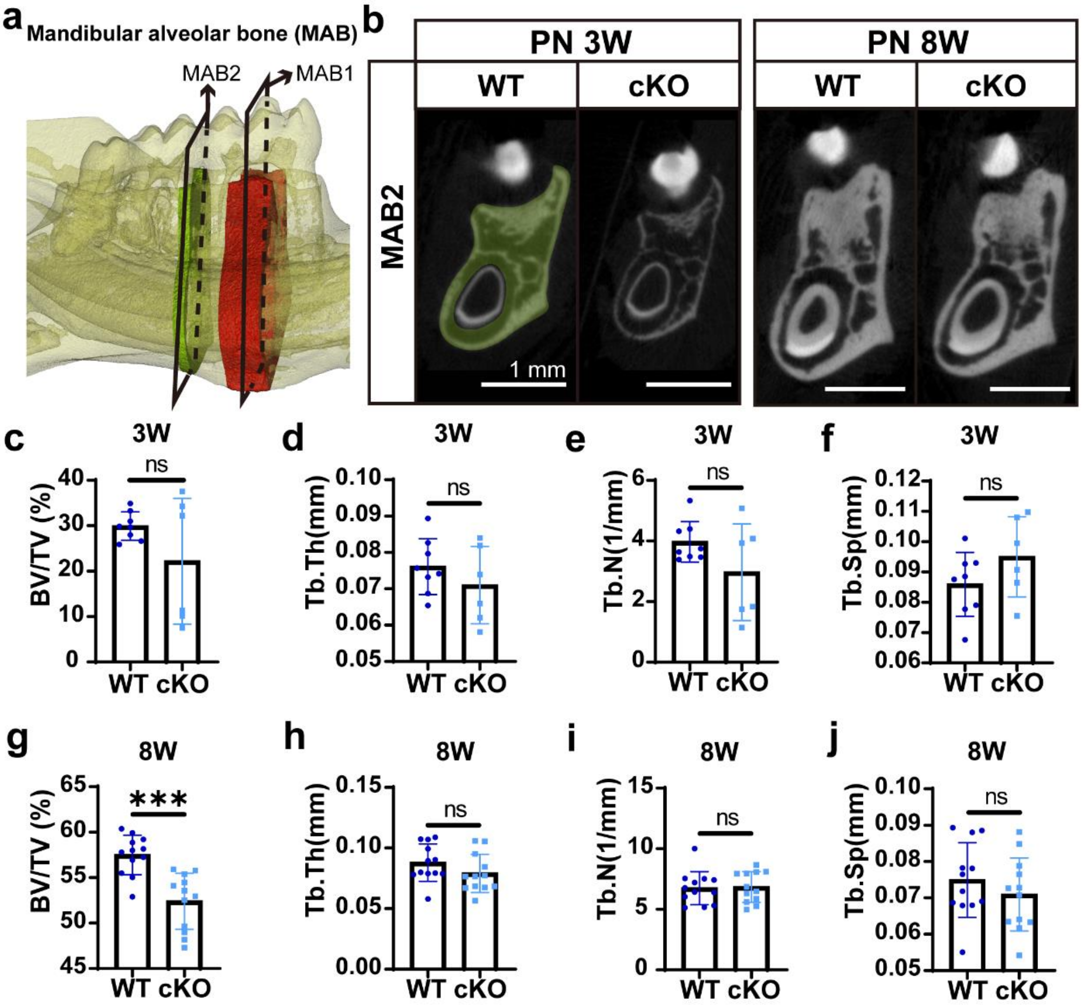

Fig. S5 Micro-CT analysis of MAB2 from WT and cKO mice at PN 3W and PN 8W. **a** The schematic diagram of the measurement sites for MAB. The red 3D area (MAB1) represents the mandibular alveolar bone in the region between the two roots of the first molar. The green 3D area (MAB2) represents the mandibular alveolar bone between the first and second molars. **b** Micro-CT sections of MAB2 from WT and cKO mice at PN 3W and PN 8W. Scale bar = 1 mm**.** **c** The quantitative data of the bone volume (BV) to tissue volume (TV) from WT and cKO mice MAB2 at PN 3W. **d** The quantitative data of the trabecular thickness (Tb.Th) from WT and cKO mice MAB2 at PN 3W. **e** The quantitative data of the trabecular number (Tb.N) from WT and cKO mice MAB2 at PN 3W. **f** The quantitative data of the trabecular separation (Tb.Sp) from WT and cKO mice MAB2 at PN 3W. **g–j** The quantitative data of the BV/TV (**g**), Tb.Th (**h**), Tb.N (**i**), Tb.Sp (**j**) from WT and cKO mice MAB2 at PN 8W. $n \geq 6$. ns, not significant, $p > 0.05$; ***$p < 0.001$.

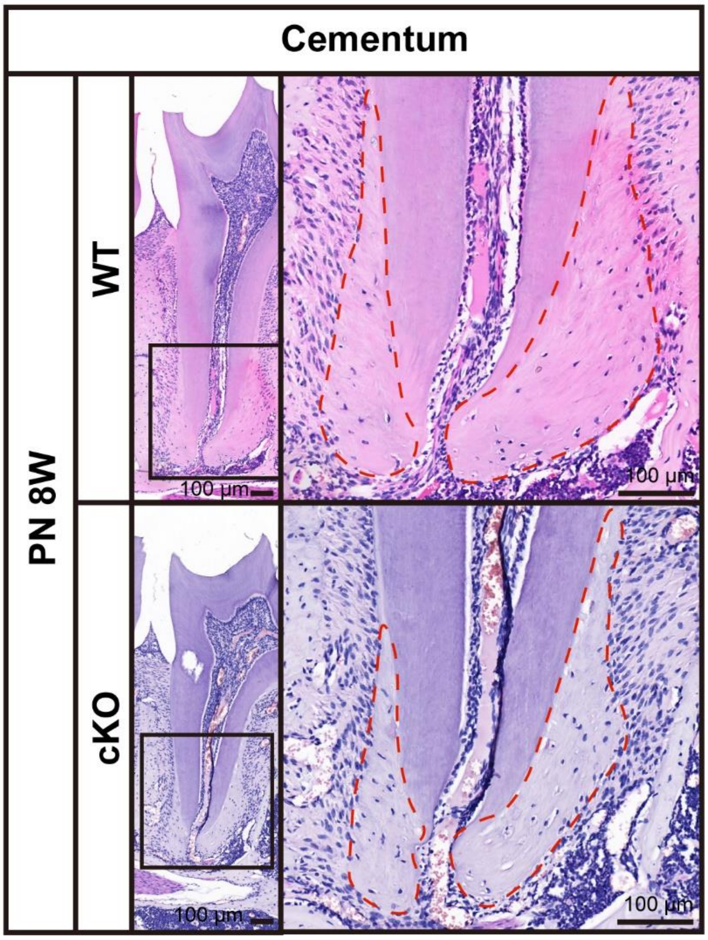

Fig. S6 The area of cementum in the first molar of cKO mice was slightly decreased compared to the WT mice at PN 8W. The HE staining of first molar roots in WT and cKO mice at PN 8W. The area delineated by the red dotted line represents the cementum. Scale bar = 100 μm**.**

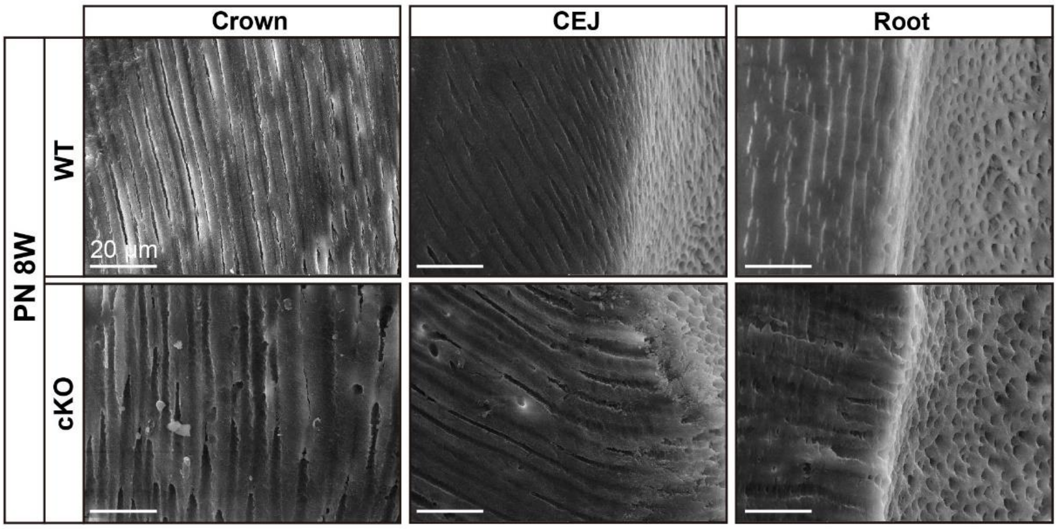

Fig. S7 No significant difference in dentinal tubule between WT and cKO. Scanning electron microscope (SEM) of dentin revealed no significant difference in dentinal tubule between WT and cKO. Scale bar = 20 μm. CEJ, cementoenamel junction.

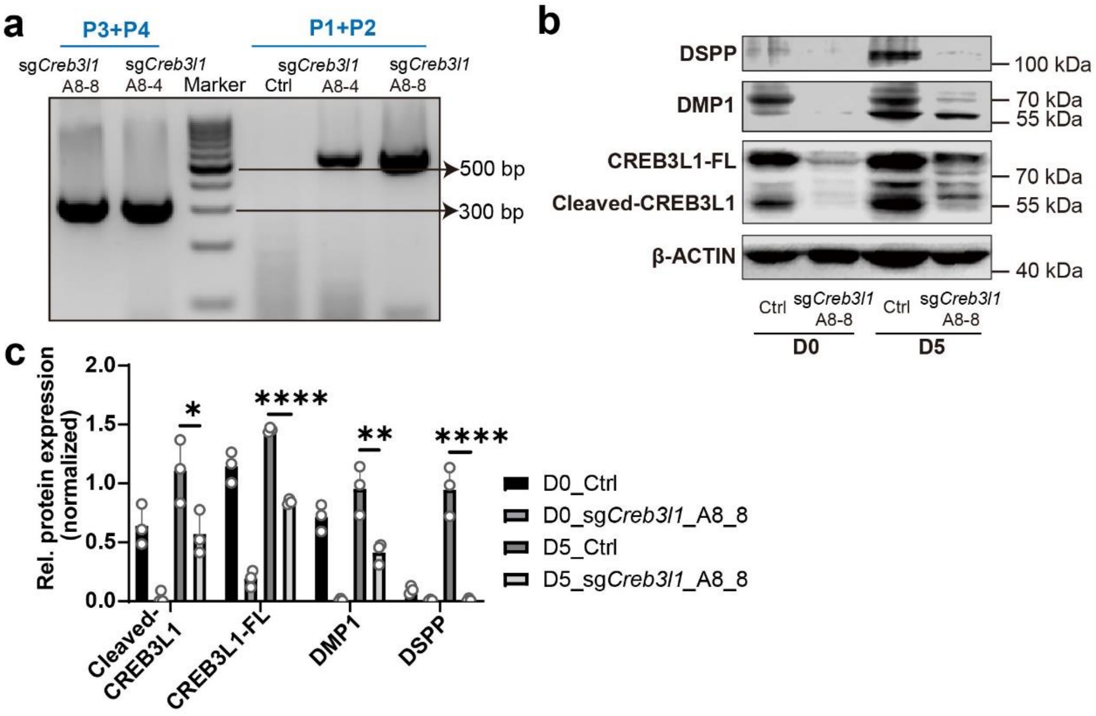

Fig. S8 Downregulation of *Creb3l1* impaired the odontoblastic differentiation ability of mDPCs. **a** Genotyping results for two heterozygote monoclonal cell lines with *Creb3l1* knockdown, sg*Creb3l1*_A8-4 and sg*Creb3l1*_A8_8. **b** The expression of the full length of CREB3L1 (CREB3L1-FL), Cleaved-CREB3L1, DMP1, and DSPP were decreased in sg*Creb3l1*_A8-8 during differentiation induction. **c** Quantification of the relative protein expression levels of (**b**). n = 3. *p < 0.05; **p < 0.01; ****p < 0.0001.

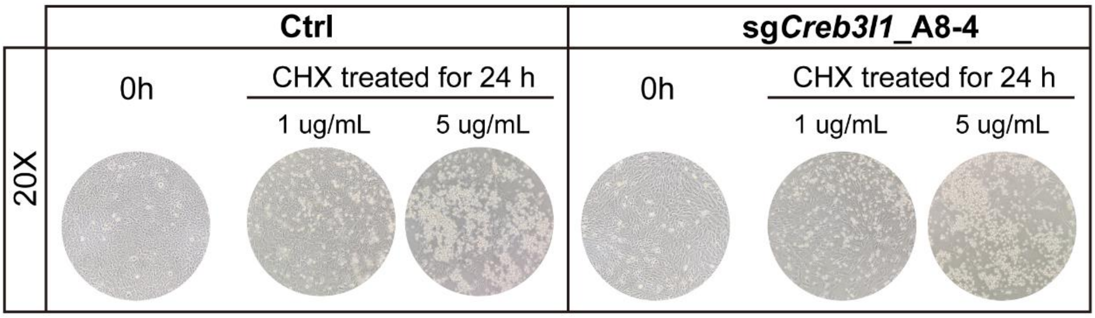

Fig. S9 Effects of two cycloheximide (CHX) concentrations on cell status after treatment for 24 h. Cells treated with CHX at a concentration of 1 ug/mL for 24 h exhibited less cell death than cells treated with CHX at a concentration of 5 ug/mL.

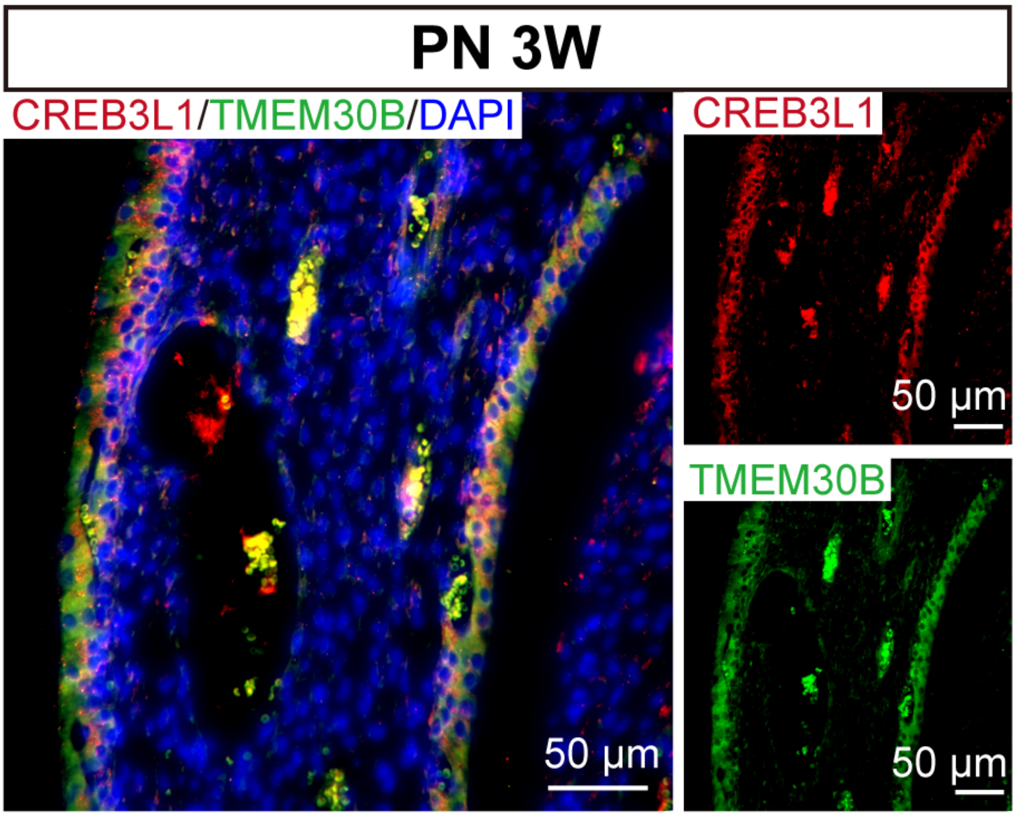

Fig. S10 The expression of TMEM30B was detected in cells expressing CREB3L1. The expression of TMEM30B and CREB3L1 in the first molar of PN 3W WT mice. Scale bar = 50 μm.

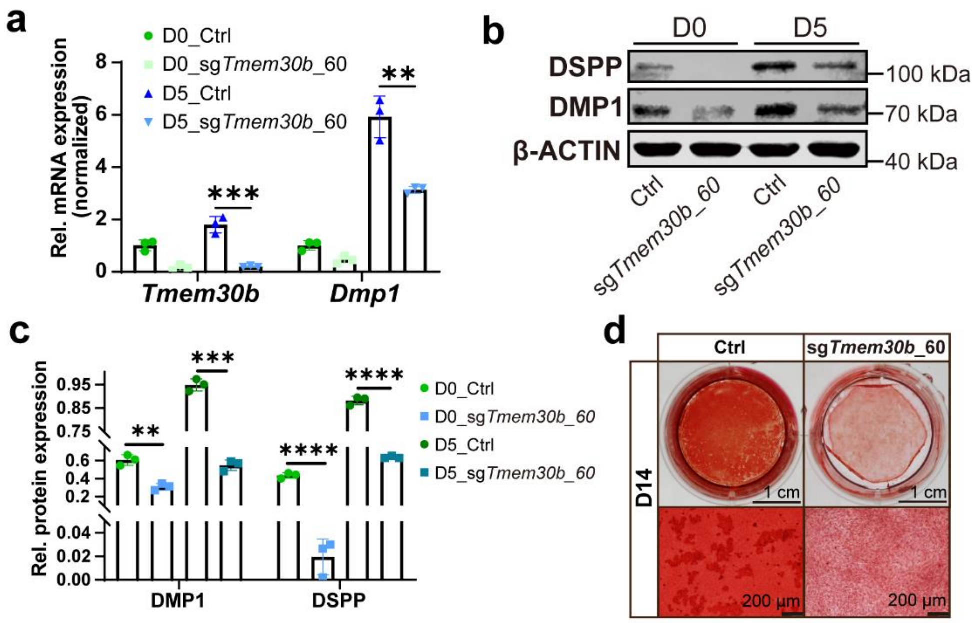

Fig. S11 Deficiency of *Tmem30b* impaired the odontoblastic differentiation capability of mDPCs. **a** During the induction of differentiation, the mRNA of *Tmem30b* and *Dspp* was decreased by *Tmem30b* deficiency (sg*Tmem30b*_60) compared to the Ctrl group. **b** During the induction of differentiation, the expression of DMP1 and DSPP was decreased in the sg*Tmem30b*_60 group compared to the Ctrl group. **c** Quantification of the relative levels of protein expression in (**b**). **d** Alizarin red S staining was performed to visualize the calcium nodules in Ctrl and sg*Tmem30b*_60 after 14 days of differentiation induction. Scale bar = 1 cm, scale bar = 200 μm. n = 3. ***p* < 0.01; ****p* < 0.001; *****p* < 0.0001.

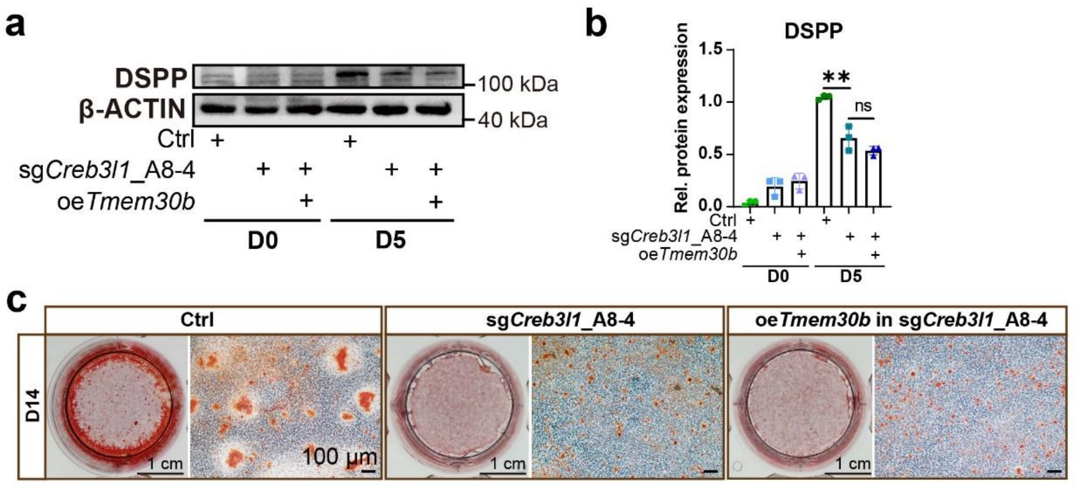

Fig. S12 The attenuated odontoblastic differentiation capability caused by CREB3L1 deficiency cannot be rescued by overexpression of TMEM30B. **a** The level of DSPP remained unchanged between the sg*Creb3l1*_A8-4 group and the sg*Creb3l1*_A8-4 group treated with oe*Tmem30b* after 5 days of differentiation induction. **b** Quantification of the relative levels of protein expression in (**a**). **c** Alizarin red S staining was performed to visualize the calcium nodules in Ctrl, sg*Creb3l1*_A8-4, and sg*Creb3l1*_A8-4 treated with oe*Tmem30b* group after 14 days of differentiation induction. Scale bar = 1 cm, scale bar = 100 μm. n = 3. ns, not significant, $p > 0.05$; **$p < 0.01$.

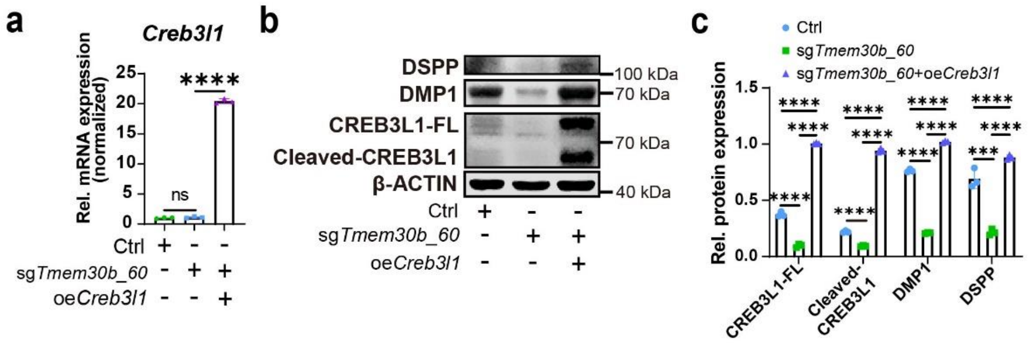

Fig. S13 Overexpression of CREB3L1 in TMEM30B-deficient mDPCs elevated the expression of DMP1 and DSPP. **a** The mRNA level of *Creb3l1* was increased in the sg*Tmem30b*_60 treated with *Creb3l1* overexpression lentivirus

(oe*Creb3l1*) group compared to sg*Tmem30b*_60 group. **b** The levels of CREB3L1-FL, Cleaved-CREB3L1, DMP1, and DSPP proteins were all upregulated in sg*Tmem30b*_60 treated with oe*Creb3l1* group compared to Ctrl and sg*Tmem30b*_60 groups. **c** Quantification of the relative levels of protein expression in (**b**). n = 3. ns, not significant, $p > 0.05$; ***$p < 0.001$; ****$p < 0.0001$.

Supplementary tables

Table S1. The sequences for the *Creb3l1* gRNAs and for the primers used in identification.	
gRNAs	
Name	Sequence
①	TGGTGGGGTGGCCATGGCGG
②	AGAGGTGGAAATGGCCGTGG
Primers	
Name	Sequence
P1	cagtgcctcaatacaaggatgc
P2	catcttgaggcagaactggaac
P3	ccaagagccaagaccttctcacgg
P4	tgtagctgttggatgaatgggaacaat

Table S1. The sequences for the Creb3l1 gRNAs and for the primers used in identification.

Table S2. ATAC-seq in D5 in mDPCs-CAS9 cells and sgCreb3l1_A8_4:
888 NFRs enriched and 510 NFRs lost at D5_Ctrl

	seqnames	start	end	width	strand	Conc	Conc_A8_4	Conc_Ctrl	Fold
85094	chr3	11213069	11213569	501	*	6.341268143	7.277271249	2.814767305	4.21167541
18494	chr11	8247451	8247951	501	*	6.169704638	7.078228435	3.144982127	3.740857371
124350	chr6	91236411	91236911	501	*	7.455387071	8.105563052	6.239942594	1.80063232
94308	chr4	55013761	55014261	501	*	7.099804138	5.719371618	7.792138822	-1.988402321
93800	chr4	45851617	45852117	501	*	6.017960631	6.828719933	3.993902349	2.626546869
50144	chr15	86645211	86645711	501	*	5.930334039	6.754812477	3.804416716	2.724463334
138240	chr8	31975653	31976153	501	*	6.267239343	7.032226872	4.533370729	2.328971713
139154	chr8	68157120	68157620	501	*	6.328954717	7.098336732	4.569991991	2.343312749
135513	chr7	123780510	123781010	501	*	5.554679575	6.501740708	1.759995844	4.304377076
6570	chr1	144086060	144086560	501	*	5.619503545	6.466706112	3.304704891	2.919852962
10463	chr10	10299872	10300372	501	*	6.037378874	6.809643595	4.261672127	2.375102109
142949	chr8	122227291	122227791	501	*	7.040193517	7.668030317	5.903350835	1.673756773
6571	chr1	144113459	144113959	501	*	5.42138897	6.316598472	2.586124295	3.409561281
142708	chr8	120962550	120963050	501	*	7.067706727	7.699953322	5.915778105	1.684355199
39559	chr14	25141859	25142359	501	*	5.809352914	3.488722884	6.657205519	-2.902633511
66037	chr18	54676110	54676610	501	*	6.093845912	6.825522698	4.535040481	2.127500148
129172	chr6	139909211	139909711	501	*	5.265938266	6.207618938	1.608228357	4.077990658
146863	chr9	62732505	62733005	501	*	5.670819746	3.084142778	6.545444623	-3.117288706
141586	chr8	106529815	106530315	501	*	6.327912271	7.002370594	5.020348667	1.866592003
129176	chr6	140147636	140148136	501	*	5.406489459	6.245315088	3.164580331	2.792176925
9898	chr1	193189267	193189767	501	*	7.156607934	6.1350583	7.748682704	-1.542295498
137627	chr8	13695029	13695529	501	*	6.82960468	7.477271804	5.623266352	1.74937612
127141	chr6	116786716	116787216	501	*	6.101970636	4.44850573	6.852254867	-2.226340504
30506	chr12	79574521	79575021	501	*	6.395627303	5.02045952	7.086707712	-1.937009404
3218	chr1	78821006	78821506	501	*	6.299573733	6.988085236	4.935115009	1.916435795
137738	chr8	15731243	15731743	501	*	5.496795214	6.472223246	0.608917475	4.912632029
41646	chr14	77484908	77485408	501	*	5.909245465	6.64056523	4.352185372	2.128705918
28044	chr12	29290898	29291398	501	*	5.891294678	6.620866574	4.342742747	2.101281442
120898	chr6	67323756	67324256	501	*	5.421319667	6.228754737	3.420758511	2.5543891
133431	chr7	83312148	83312648	501	*	5.942982823	6.659251765	4.457271184	2.054202354
126466	chr6	111733864	111734364	501	*	5.761488898	6.517166956	4.079140488	2.239238096
62104	chr17	56331249	56331749	501	*	6.613528593	5.325719595	7.282943138	-1.82495544
136439	chr7	139308975	139309475	501	*	6.874275695	7.473605863	5.830293691	1.549233633
119781	chr6	53859115	53859615	501	*	6.482792595	7.125197261	5.295319641	1.734481766
143649	chr8	128164916	128165416	501	*	6.107345162	6.786792908	4.779900546	1.869586882
89711	chr3	109936195	109936695	501	*	5.358306641	6.185498435	3.211242162	2.672090139
6565	chr1	143941415	143941915	501	*	5.062627929	5.971345166	2.034947091	3.526080291
143260	chr8	123999532	124000032	501	*	6.618005755	7.233544966	5.522183759	1.614569164
36209	chr13	60012368	60012868	501	*	5.833381042	6.559463335	4.30163658	2.069536271
151922	chrX	98452033	98452533	501	*	5.138021676	6.009851818	2.581773714	3.014979034
31765	chr12	102302795	102303295	501	*	6.167217266	4.734962099	6.87160128	-1.979233254
12057	chr10	41486733	41487233	501	*	5.840681705	6.558309532	4.348701145	2.015528524
34184	chr13	32704817	32705317	501	*	5.646060592	1.238159142	6.611673177	-4.512506665
120751	chr6	66947383	66947883	501	*	5.224411282	6.034786395	3.203091044	2.531883825
68638	chr19	9022947	9023447	501	*	6.710615704	5.617854993	7.325218788	-1.605765403
126967	chr6	115555419	115555919	501	*	6.546312023	7.161249854	5.452457609	1.594208014
63840	chr17	86277231	86277731	501	*	6.543215015	5.29149719	7.203187167	-1.792562511

42285	chr15	6001456	6001956	501	*	5.531732146	3.480742131	6.346226438	-2.569896306
118949	chr6	49987635	49988135	501	*	5.349500713	6.124266649	3.559079101	2.331129697
6568	chr1	144006501	144007001	501	*	4.928379993	5.813403285	2.221920584	3.110591453
125796	chr6	100059708	100060208	501	*	6.920745856	5.914864808	7.507663391	-1.501463507
96496	chr4	97601322	97601822	501	*	6.192916626	6.841778279	4.982251775	1.724184777
114068	chr5	141769033	141769533	501	*	5.823386549	4.276699708	6.552573627	-2.083298618
151204	chrX	51058687	51059187	501	*	6.218742257	6.851974155	5.063414878	1.668239752
121575	chr6	80018685	80019185	501	*	5.040332444	5.878279869	2.805832003	2.706726953
28332	chr12	33019979	33020479	501	*	6.466596593	7.082802771	5.368588321	1.592249531
131535	chr7	40536540	40537040	501	*	5.01117197	5.852807642	2.745266987	2.76934617
140791	chr8	91545489	91545989	501	*	5.480445661	6.221297254	3.875894586	2.130586647
43540	chr15	30569510	30570010	501	*	4.811104219	5.725708732	1.690154357	3.510630379
34531	chr13	38279073	38279573	501	*	5.588531442	3.852744319	6.353858123	-2.25812306
119536	chr6	52796038	52796538	501	*	6.499030136	5.367212873	7.125384655	-1.639570387
138346	chr8	34456493	34456993	501	*	4.784455747	5.698586086	1.671259803	3.395497991
96507	chr4	97695405	97695905	501	*	5.466619845	6.215727885	3.819363156	2.145999643
75978	chr2	50737437	50737937	501	*	5.509891529	3.597579512	6.304272911	-2.426754614
64664	chr18	14331385	14331885	501	*	5.483811486	6.216233351	3.921355425	2.077405217
5294	chr1	126656224	126656724	501	*	6.498653153	7.126353665	5.362273478	1.634530647
42613	chr15	9748674	9749174	501	*	5.756532676	6.448226278	4.378794025	1.882885481
40434	chr14	49493775	49494275	501	*	4.888173103	5.845475732	0.788401748	4.230982187
121760	chr6	82981121	82981621	501	*	5.73867762	4.16680572	6.47301078	-2.084278088
44301	chr15	38268714	38269214	501	*	6.28595917	5.024021376	6.948635462	-1.769304161
5131	chr1	120136146	120136646	501	*	6.238586607	6.880981582	5.051148104	1.679395529
144373	chr9	22937852	22938352	501	*	6.423644633	5.346627137	7.033392283	-1.576956543
140827	chr8	91937161	91937661	501	*	6.186238681	6.80323156	5.085646085	1.593041609
109182	chr5	103387366	103387866	501	*	6.411142436	5.32347272	7.024182961	-1.591369023
12497	chr10	53157645	53158145	501	*	4.913625061	5.779840292	2.41646898	2.856654496
120957	chr6	67962192	67962692	501	*	4.793943155	5.669182211	2.200484112	3.005026864
140629	chr8	88282637	88283137	501	*	5.820307267	6.482457884	4.560363379	1.776227191
141290	chr8	103534946	103535446	501	*	6.019148974	6.671693864	4.795038541	1.722184833
28102	chr12	30865611	30866111	501	*	5.905643695	6.572854568	4.626360089	1.781803706
1865	chr1	58056000	58056500	501	*	5.434460948	3.578940522	6.219942062	-2.347682516
127714	chr6	121017578	121018078	501	*	4.826040374	5.670406557	2.536384057	2.74915984
32981	chr13	3767075	3767575	501	*	5.493241621	3.794681193	6.25189157	-2.190297259
30186	chr12	76117617	76118117	501	*	5.175156699	2.94649853	6.012408031	-2.654073623
95107	chr4	63084592	63085092	501	*	5.637439218	4.069686309	6.370938122	-2.069616479
43254	chr15	25886480	25886980	501	*	6.171808471	4.992138741	6.812010553	-1.691292158
70398	chr19	41817092	41817592	501	*	5.433784538	3.669091525	6.204157516	-2.255442562
66667	chr18	63454757	63455257	501	*	6.099501488	6.713022318	5.010269382	1.57951193
4611	chr1	93211397	93211897	501	*	6.582822127	5.568154499	7.172640885	-1.50661864
71219	chr19	53852683	53853183	501	*	5.32006247	3.407135239	6.114538068	-2.380802838
140276	chr8	84501733	84502233	501	*	5.620555963	6.310368171	4.250687277	1.869506392
85804	chr3	38297459	38297959	501	*	5.263508957	3.307228035	6.064508724	-2.425774161
54822	chr16	33903862	33904362	501	*	5.646499959	6.322097464	4.334427242	1.811576982
84256	chr2	172758450	172758950	501	*	4.80137404	5.644804756	2.519904062	2.668263619
138875	chr8	54724237	54724737	501	*	5.846253408	6.505395986	4.597652586	1.733983212
62387	chr17	62984822	62985322	501	*	5.193405808	3.115406526	6.011567051	-2.527446852
89721	chr3	113705560	113706060	501	*	5.203454926	3.099569785	6.025058264	-2.534365608
98457	chr4	118539978	118540478	501	*	6.141254837	4.931549527	6.78985196	-1.713278207
6567	chr1	144004054	144004554	501	*	6.131329425	6.984729403	3.759840916	2.748824715

10052	chr10	3572922	3573422	501	*	4.915079845	5.744167781	2.753027122	2.57650208
22565	chr11	81369826	81370326	501	*	5.198768947	3.233467993	6.001098026	-2.409067162
27927	chr12	26329268	26329768	501	*	5.477673346	6.200297753	3.962353284	1.985700131
6574	chr1	144188883	144189383	501	*	4.798741932	5.624292495	2.664510507	2.534608331
37399	chr13	94158785	94159285	501	*	6.295778774	5.232985879	6.90107924	-1.554452756
24812	chr11	106845913	106846413	501	*	5.417729533	3.749117555	6.170858369	-2.13934717
144050	chr9	20682071	20682571	501	*	5.016944308	2.6175939	5.873292722	-2.772999916
135258	chr7	116993635	116994135	501	*	5.293632061	6.031015102	3.706580515	2.062386716
5271	chr1	125996536	125997036	501	*	6.004334025	6.619556067	4.909550174	1.576121313
71321	chr19	55560025	55560525	501	*	5.691499938	4.225312605	6.403490304	-1.972092305
63191	chr17	78521179	78521679	501	*	5.838399734	4.476735137	6.526237077	-1.862901565
25145	chr11	112239276	112239776	501	*	5.678660499	4.296027988	6.371517635	-1.875670669
81251	chr2	143018553	143019053	501	*	4.948618597	2.302791982	5.828495578	-2.975800886
30575	chr12	79859491	79859991	501	*	5.114759259	3.008814059	5.936633534	-2.500528718
58376	chr17	14647901	14648401	501	*	5.188350352	3.261860027	5.984891323	-2.382051931
142910	chr8	122064298	122064798	501	*	5.452271531	6.164524132	3.984896773	1.948134483
129170	chr6	139770100	139770600	501	*	4.625263206	5.495060582	2.090715535	2.828705317
46133	chr15	64622548	64623048	501	*	6.534057949	7.138137037	5.47513967	1.523656358
43009	chr15	13232512	13233012	501	*	4.519250673	5.425393102	1.530426866	3.255544933
94447	chr4	56411222	56411722	501	*	5.339904653	3.660383625	6.095060351	-2.146066786
107382	chr5	53493659	53494159	501	*	5.837643138	6.486989376	4.625218416	1.694641273
28787	chr12	41946507	41947007	501	*	6.34291988	5.312846055	6.937769271	-1.50800678
109084	chr5	101353879	101354379	501	*	5.166685412	5.905298038	3.573459426	2.055465753
12449	chr10	45893829	45894329	501	*	4.541572028	5.413932643	1.979601919	2.889405351
140000	chr8	79561928	79562428	501	*	5.282568575	6.026592278	3.661788088	2.08109481
43653	chr15	32002811	32003311	501	*	4.901377264	5.686429072	3.048531818	2.310003266
6385	chr1	136725191	136725691	501	*	6.025335665	4.868855891	6.658900964	-1.647829546
25261	chr11	113727026	113727526	501	*	5.061215335	2.983733772	5.87930702	-2.504146758
5741	chr1	133119896	133120396	501	*	5.238970171	3.238122058	6.046446212	-2.429672132
11677	chr10	35170253	35170753	501	*	4.518883655	5.409696523	1.740742754	3.127368548
127340	chr6	118336536	118337036	501	*	5.517272518	4.045672099	6.230456196	-1.957456702
104106	chr4	155606722	155607222	501	*	5.766406624	6.408159852	4.581247199	1.667490416
60365	chr17	31670065	31670565	501	*	5.25952487	3.525747124	6.024496323	-2.194171197
150751	chrX	14273863	14274363	501	*	5.996352218	6.64180076	4.798005775	1.662589734
90844	chr3	138063875	138064375	501	*	5.574618323	4.148221239	6.27766683	-1.913144538
81250	chr2	143012989	143013489	501	*	6.165531675	5.088004735	6.775437533	-1.553734341
51434	chr15	99431932	99432432	501	*	6.218206571	5.152074939	6.824556037	-1.539152204
36179	chr13	59718888	59719388	501	*	5.515634504	4.065616257	6.224027659	-1.928129039
112028	chr5	123980694	123981194	501	*	5.222124517	5.94230165	3.718293189	1.977063845
132048	chr7	49028964	49029464	501	*	4.986713373	2.832684074	5.814789038	-2.559001222
5276	chr1	126331992	126332492	501	*	5.061139607	5.805821175	3.436965814	2.076068452
71495	chr19	56939787	56940287	501	*	5.856756784	6.473959898	4.755472542	1.574396662
17024	chr10	116017733	116018233	501	*	4.864211349	5.638134491	3.078767604	2.2267677
9628	chr1	190952873	190953373	501	*	5.966891326	6.56464492	4.927823194	1.502897321
6652	chr1	150551385	150551885	501	*	5.025961198	5.782208574	3.340523742	2.113888702
34163	chr13	32495008	32495508	501	*	5.581267281	4.199903062	6.273823344	-1.855863339
90805	chr3	137525661	137526161	501	*	5.438228487	3.939510592	6.157308752	-1.980347497
37029	chr13	81401700	81402200	501	*	4.499336821	5.374820257	1.903169504	2.895562992
38065	chr13	102933644	102934144	501	*	5.206261695	3.472567919	5.97121829	-2.181648503
112564	chr5	130623130	130623630	501	*	5.839838333	6.45707919	4.738429873	1.564776531
22319	chr11	78505379	78505879	501	*	4.891751685	2.610161349	5.735196197	-2.622161503

135277	chr7	117494094	117494594	501	*	5.419039944	6.09983521	4.086166703	1.815969333
151994	chrX	99350319	99350819	501	*	5.144047215	3.347235349	5.919890103	-2.225560562
117159	chr6	34623233	34623733	501	*	5.01341294	2.97218415	5.826562674	-2.44288104
45931	chr15	62279471	62279971	501	*	4.83075166	2.320786192	5.698205515	-2.823563772
18756	chr11	17685625	17686125	501	*	5.286635184	5.989686877	3.86022416	1.888604769
102333	chr4	141592266	141592766	501	*	5.161750588	3.427960643	5.926724199	-2.180969539
37989	chr13	101906081	101906581	501	*	5.149567626	3.385971339	5.919751299	-2.216637776
17412	chr10	121837072	121837572	501	*	5.137318321	5.86766523	3.585001291	2.008428426
129372	chr6	143425497	143425997	501	*	4.452700481	5.32460851	1.895613908	2.851434709
107509	chr5	64062918	64063418	501	*	5.623728865	4.307311619	6.300419762	-1.795123897
95483	chr4	72105730	72106230	501	*	5.106363422	3.235884729	5.89422824	-2.289731487
117540	chr6	37254125	37254625	501	*	5.700776117	6.337589249	4.533007802	1.630726567
117493	chr6	36978022	36978522	501	*	5.800739841	4.552659483	6.459743679	-1.728675015
69034	chr19	16868581	16869081	501	*	5.466148088	3.996121024	6.178985484	-1.933509629
63482	chr17	81542225	81542725	501	*	5.027053844	3.027855288	5.834294238	-2.390780759
17009	chr10	115587254	115587754	501	*	5.387147724	6.066684224	4.059346336	1.791672925
76214	chr2	53370201	53370701	501	*	4.902296865	5.663456588	3.189875544	2.156531398
63202	chr17	78725694	78726194	501	*	5.702278663	4.452516212	6.361730668	-1.732060265
80209	chr2	127426190	127426690	501	*	4.940900019	2.884677492	5.756110815	-2.433844218
141273	chr8	103398134	103398634	501	*	5.666094088	6.307024331	4.483850643	1.646602023
27860	chr12	25189056	25189556	501	*	5.160837766	5.872197614	3.697500601	1.931423134
140628	chr8	88280874	88281374	501	*	5.809375455	6.430810581	4.694075725	1.572741056
26765	chr12	5668381	5668881	501	*	4.842305979	2.524058304	5.68989337	-2.645392891
55097	chr16	35681314	35681814	501	*	5.181490464	3.527720961	5.931832249	-2.100414999
63257	chr17	79315444	79315944	501	*	5.778510252	4.581669634	6.42353901	-1.661211517
143534	chr8	126683873	126684373	501	*	5.34367426	6.0255797	4.006309343	1.804486647
101711	chr4	139175302	139175802	501	*	6.169512639	5.131954372	6.766780378	-1.508565531
103390	chr4	151719067	151719567	501	*	5.620667082	4.329634932	6.290910499	-1.763987176
70258	chr19	40231036	40231536	501	*	6.021119332	4.871246224	6.652768576	-1.622252748
37121	chr13	90473829	90474329	501	*	5.841347658	4.656061642	6.483136565	-1.664064629
138965	chr8	61310415	61310915	501	*	5.53007315	6.183649392	4.302169021	1.697526092
99458	chr4	128698014	128698514	501	*	5.675375652	4.436177655	6.332001914	-1.704507858
107222	chr5	51568111	51568611	501	*	5.277732513	3.698540746	6.013541186	-2.041856876
139453	chr8	70710288	70710788	501	*	5.720397679	6.343391073	4.599892367	1.579268334
45420	chr15	57753039	57753539	501	*	5.837754097	6.464179998	4.705695343	1.578754637
59192	chr17	26451800	26452300	501	*	5.860123623	4.727377995	6.486752473	-1.595171395
64075	chr17	89450654	89451154	501	*	4.351053908	5.27211676	1.119847127	3.273679238
85248	chr3	21726793	21727293	501	*	5.545846308	4.24413693	6.218818947	-1.771816938
90638	chr3	132911671	132912171	501	*	5.995268777	4.859665834	6.622740528	-1.58936776
13368	chr10	65226963	65227463	501	*	5.265314304	3.651491168	6.007983385	-2.049272655
3223	chr1	78865992	78866492	501	*	4.39287231	5.336275839	0.692756116	3.585549645
91954	chr4	5264399	5264899	501	*	5.273150137	3.682941524	6.011162669	-2.044118851
115517	chr6	13816596	13817096	501	*	5.178305011	3.510294194	5.931321624	-2.094280446
127504	chr6	119460086	119460586	501	*	5.608367309	4.33624354	6.273714772	-1.746319269
36612	chr13	68572933	68573433	501	*	5.454357294	4.04822446	6.152734261	-1.876257418
17013	chr10	115627392	115627892	501	*	4.703272876	5.512354115	2.691130465	2.413036136
55576	chr16	45924086	45924586	501	*	5.05214824	3.200102173	5.837071576	-2.230644478
102432	chr4	142097771	142098271	501	*	5.833629652	6.433372556	4.788357574	1.510147729
138947	chr8	60859553	60860053	501	*	4.971992329	5.708783004	3.387904109	2.003739342
16605	chr10	99789465	99789965	501	*	4.905654031	2.875406946	5.717278659	-2.38859661
43991	chr15	36311501	36312001	501	*	5.301060353	5.984718352	3.956569047	1.791590931

140813	chr8	91777681	91778181	501	*	4.923235238	5.676904096	3.251726283	2.076383155
146204	chr9	57656884	57657384	501	*	5.916844355	4.816934506	6.533629575	-1.55597749
145444	chr9	45917435	45917935	501	*	5.786686662	4.505351852	6.454429235	-1.752286254
36281	chr13	60400804	60401304	501	*	5.602616475	4.354795331	6.261551191	-1.711975761
105640	chr5	31955122	31955622	501	*	5.524631296	4.182904621	6.207610682	-1.810768082
142454	chr8	120015926	120016426	501	*	5.478048568	6.151443523	4.174678118	1.759851789
72134	chr2	6483308	6483808	501	*	5.848331652	4.686544012	6.483428157	-1.639283643
152914	chrX	157390005	157390505	501	*	5.570259271	4.267365527	6.243533089	-1.767592925
16280	chr10	95139622	95140122	501	*	5.901057729	4.764784266	6.528726969	-1.615032819
129012	chr6	135700624	135701124	501	*	5.072744808	5.795487997	3.556864287	1.950805062
97145	chr4	106261198	106261698	501	*	4.884338213	2.794561946	5.704074144	-2.431480465
87220	chr3	79261999	79262499	501	*	5.482749949	4.153344112	6.162685249	-1.798475037
75807	chr2	45522831	45523331	501	*	4.81707065	2.628136182	5.649504316	-2.516670994
39035	chr14	13690429	13690929	501	*	5.413777628	6.090769602	4.096161192	1.758575554
55091	chr16	35630757	35631257	501	*	4.982702741	3.175644603	5.76026085	-2.207839166
139148	chr8	68117435	68117935	501	*	5.155123359	5.855810425	3.739013529	1.858048988
68123	chr19	5471783	5472283	501	*	5.403439383	4.033423046	6.093287006	-1.825850074
85787	chr3	38094445	38094945	501	*	5.279237056	3.794561946	5.995280622	-1.929194327
87027	chr3	66953533	66954033	501	*	5.599023968	4.320703384	6.265984884	-1.749212568
142216	chr8	116343275	116343775	501	*	5.06342788	3.28240411	5.836599828	-2.177801854
64469	chr18	11407901	11408401	501	*	4.972394619	5.705425982	3.406943937	1.993459777
89555	chr3	108174646	108175146	501	*	4.695815781	2.150804648	5.566597493	-2.762387024
62386	chr17	62961958	62962458	501	*	4.715807851	2.2845705	5.575454357	-2.683232255
31971	chr12	104801823	104802323	501	*	4.949908901	3.081457752	5.737452292	-2.240216016
96348	chr4	93440523	93441023	501	*	5.649864783	4.442392917	6.297845312	-1.670206365
151583	chrX	73567344	73567844	501	*	5.281578128	5.958100707	3.965830754	1.770034172
78903	chr2	110038681	110039181	501	*	5.731853999	4.518098299	6.381566225	-1.687203578
137894	chr8	23740555	23741055	501	*	4.761989824	5.546902473	2.910010234	2.201732171
120481	chr6	59434521	59435021	501	*	5.762343151	4.580773805	6.403082838	-1.649350328
122652	chr6	85949980	85950480	501	*	5.178476606	5.86766523	3.811204702	1.819311394
5991	chr1	134516514	134517014	501	*	5.655826828	4.434211329	6.307691564	-1.694443377
132332	chr7	64761103	64761603	501	*	4.326037465	5.203119636	1.712014602	2.786737301
39906	chr14	31079285	31079785	501	*	4.770708729	2.649783676	5.59454004	-2.464664803
39929	chr14	31226870	31227370	501	*	5.527498905	4.211890466	6.203986561	-1.75637561
109031	chr5	100604001	100604501	501	*	5.861340577	4.787452647	6.47011473	-1.530484865
54547	chr16	31538389	31538889	501	*	5.375288682	3.986420071	6.069621416	-1.827777865
130773	chr7	27081570	27082070	501	*	4.390506488	5.22584151	2.177796292	2.511555951
140711	chr8	89812059	89812559	501	*	4.557859688	5.358560518	2.603598316	2.293284655
28806	chr12	45047057	45047557	501	*	4.323730401	5.178651207	1.937944806	2.670254632
85146	chr3	16527349	16527849	501	*	5.177055229	5.865382496	3.81336076	1.79750845
70558	chr19	43845054	43845554	501	*	5.02827671	3.362315904	5.780910216	-2.079525552
80552	chr2	130657716	130658216	501	*	4.992548745	5.707540869	3.512697361	1.921954056
78472	chr2	102201455	102201955	501	*	5.439429282	4.102414031	6.121248452	-1.784907884
140472	chr8	86449804	86450304	501	*	5.169780255	5.848156383	3.846634628	1.770004604
120675	chr6	66224199	66224699	501	*	5.59353267	6.213912492	4.481744492	1.553865863
17446	chr10	122782697	122783197	501	*	4.824257407	2.851259267	5.627713333	-2.347675516
44787	chr15	48585862	48586362	501	*	4.899284862	3.054884398	5.682975259	-2.229421594
3577	chr1	86300081	86300581	501	*	5.286166582	3.788323387	6.005058744	-1.948565246
100164	chr4	132808278	132808778	501	*	5.494608178	4.247733853	6.15329028	-1.703032442
32934	chr12	119164065	119164565	501	*	5.178066522	3.691642803	5.89449018	-1.929616392
87316	chr3	81505222	81505722	501	*	4.71783122	2.500423441	5.553733171	-2.486725806

Table S2. ATAC-seq in D5 in mDPCs-CAS9 cells and sgCreb3l1_A8_4: 888 NFRs enriched and 510 NFRs lost at D5_Ctrl

138836	chr8	53859342	53859842	501	*	4.868176151	5.600545082	3.305979815	1.985662942
56070	chr16	61772345	61772845	501	*	4.404754266	5.230520202	2.268846128	2.436955654
147635	chr9	71032133	71032633	501	*	5.520904183	4.294869464	6.173972639	-1.672164489
111046	chr5	117780554	117781054	501	*	5.245057487	5.932477619	3.885118708	1.777583918
78214	chr2	93666100	93666600	501	*	4.882067516	3.041533635	5.665131488	-2.202189712
39449	chr14	23422606	23423106	501	*	5.443378272	4.152065098	6.113693848	-1.739068293
103361	chr4	151417804	151418304	501	*	4.593939752	2.030641853	5.466423132	-2.73690429
30081	chr12	74266900	74267400	501	*	4.316611047	5.158863894	2.045375585	2.50811874
143248	chr8	123866928	123867428	501	*	5.24718783	5.90865504	3.989830932	1.692041055
137259	chr8	10130777	10131277	501	*	5.187002272	5.861242464	3.880300069	1.735378746
139076	chr8	64726134	64726634	501	*	5.513684076	6.155764397	4.3273636	1.613604373
115488	chr6	12499765	12500265	501	*	4.492609922	5.297670995	2.508565548	2.313131609
151772	chrX	81732473	81732973	501	*	5.164070629	5.842674754	3.84001169	1.746404186
132208	chr7	58641849	58642349	501	*	4.816109578	2.960966426	5.601530199	-2.209453459
37522	chr13	95790841	95791341	501	*	5.581045738	4.401982438	6.221076005	-1.633274054
62172	chr17	56515988	56516488	501	*	5.68853248	4.523842519	6.324463179	-1.616514126
106762	chr5	37804367	37804867	501	*	5.63340395	4.478777134	6.266432998	-1.607319849
56419	chr16	78099781	78100281	501	*	5.260418587	5.925226463	3.990359751	1.689859087
40579	chr14	54736817	54737317	501	*	5.424987452	4.08947733	6.106434978	-1.783884381
79316	chr2	117820946	117821446	501	*	5.060542751	3.508411024	5.790851574	-1.982550181
142775	chr8	121444865	121445365	501	*	5.079284348	5.758087229	3.754428638	1.759198558
29415	chr12	59970749	59971249	501	*	5.227937365	5.891891057	3.961139786	1.720278504
52722	chr16	7558502	7559002	501	*	4.364750285	5.183576279	2.281792664	2.383697297
138732	chr8	46293645	46294145	501	*	5.069188947	5.752728632	3.72518012	1.774421098
22591	chr11	81845284	81845784	501	*	5.585066192	4.447463444	6.213126597	-1.584656914
97750	chr4	109484532	109485032	501	*	5.213258615	5.871499466	3.968036253	1.679975737
17221	chr10	119752237	119752737	501	*	4.276081737	5.123022772	1.963621878	2.526507345
105941	chr5	33955868	33956368	501	*	5.407510698	6.055038999	4.201674108	1.625377716
28804	chr12	44857988	44858488	501	*	4.254090654	5.093876655	2.004030658	2.510463514
30540	chr12	79707443	79707943	501	*	5.579967272	4.42901711	6.211929654	-1.598977823
99507	chr4	128906637	128907137	501	*	4.760510346	2.820088295	5.559149676	-2.278345798
143001	chr8	122423592	122424092	501	*	5.317858815	5.962040687	4.124049984	1.624130487
20005	chr11	50622149	50622649	501	*	5.009969195	5.698116101	3.64702242	1.797146854
129378	chr6	143571380	143571880	501	*	4.891151016	5.596012803	3.456793024	1.854723033
90807	chr3	137547944	137548444	501	*	5.556625384	4.361289942	6.201233977	-1.64221702
3222	chr1	78852362	78852862	501	*	4.666730613	5.441193862	2.878099099	2.122690703
109088	chr5	101426059	101426559	501	*	4.833290851	5.564794136	3.275333919	1.965761345
145629	chr9	50095112	50095612	501	*	5.302101055	3.985088595	5.97894143	-1.74161828
77432	chr2	80054021	80054521	501	*	5.699620381	4.59550818	6.317681721	-1.542915373
51576	chr15	99863181	99863681	501	*	4.765344749	2.857370398	5.559060531	-2.221538662
33493	chr13	20046344	20046844	501	*	4.510806606	1.311983775	5.430026425	-3.135808182
91584	chr3	152108853	152109353	501	*	5.338118547	4.025266603	6.013912464	-1.742966592
150756	chrX	16356835	16357335	501	*	4.15036673	5.096480095	0.380812222	3.408545179
88868	chr3	100411886	100412386	501	*	5.098108453	5.774007991	3.784837261	1.729671056
139102	chr8	66777325	66777825	501	*	5.453672881	6.098521782	4.257476821	1.610070007
89747	chr3	114065409	114065909	501	*	5.297255871	3.992045743	5.971117838	-1.734657521
86096	chr3	50044633	50045133	501	*	5.243961346	3.880162052	5.93231389	-1.785978608
142936	chr8	122174002	122174502	501	*	5.505398439	6.12844767	4.384706146	1.55556669
17218	chr10	119615196	119615696	501	*	4.150266923	5.012831476	1.690154357	2.659770776
139098	chr8	66696909	66697409	501	*	4.798922804	5.535035059	3.218219447	1.953595105
122203	chr6	84167463	84167963	501	*	5.493205475	4.277872909	6.143350745	-1.654462082

45277	chr15	56056437	56056937	501	*	5.061697015	3.59004286	5.774892512	-1.892340359
55762	chr16	54041857	54042357	501	*	5.340413121	5.973072356	4.187062372	1.585408269
59489	chr17	27591382	27591882	501	*	5.073467877	3.590180268	5.789209444	-1.901754582
64610	chr18	13390201	13390701	501	*	5.470795381	6.079523055	4.397056646	1.508233273
63342	chr17	79991172	79991672	501	*	4.773817879	2.943759526	5.555174868	-2.157078639
116333	chr6	29814566	29815066	501	*	4.621891661	2.496397501	5.446315344	-2.377586769
92541	chr4	14621662	14622162	501	*	5.616754404	4.487651099	6.242305636	-1.564359374
46428	chr15	68606024	68606524	501	*	5.123739472	5.788627877	3.853372701	1.701980086
144000	chr9	14842327	14842827	501	*	5.254627641	3.866733207	5.948730383	-1.820933609
58065	chr17	8864490	8864990	501	*	4.101873701	4.998402279	1.248985095	2.827161908
136375	chr7	138867217	138867717	501	*	5.495752026	6.103684595	4.424562433	1.506178551
19790	chr11	46127553	46128053	501	*	4.644788743	5.390294279	3.016351388	2.000071049
67168	chr18	74721014	74721514	501	*	4.728129922	5.469714678	3.119847127	1.965179983
143273	chr8	124182083	124182583	501	*	5.31429268	5.973475996	4.065538988	1.66534898
92249	chr4	10189824	10190324	501	*	5.22345167	3.872529453	5.908681964	-1.763515738
34596	chr13	40137063	40137563	501	*	4.152842612	5.004608993	1.796538578	2.565635793
120712	chr6	66848844	66849344	501	*	5.203251926	5.86426818	3.947598885	1.697318906
30593	chr12	79917736	79918236	501	*	5.212415278	3.871088552	5.895296357	-1.753546077
45690	chr15	59237768	59238268	501	*	4.71706989	5.45711686	3.116601011	1.953759363
50148	chr15	86696773	86697273	501	*	4.207241333	5.059737663	1.844174202	2.532114893
139892	chr8	77535023	77535523	501	*	5.116274034	5.787040711	3.823202467	1.718468634
119282	chr6	51489664	51490164	501	*	5.458986619	4.153363776	6.132953253	-1.722210128
43547	chr15	30762879	30763379	501	*	5.223009578	5.871139118	4.014998364	1.631195767
84778	chr2	181657406	181657906	501	*	4.905883734	3.270837476	5.652660767	-1.990390951
74617	chr2	32074392	32074892	501	*	5.157205333	3.789754976	5.846436855	-1.778513361
25282	chr11	113927232	113927732	501	*	5.058162231	5.721903893	3.792172736	1.695037385
142764	chr8	121379377	121379877	501	*	4.390176213	5.184257248	2.479823398	2.248153881
19721	chr11	45229560	45230060	501	*	5.226246029	3.930695373	5.897647525	-1.7212758
72724	chr2	20419963	20420463	501	*	5.52135517	4.357098036	6.157161601	-1.596935646
33755	chr13	24655104	24655604	501	*	5.717750974	4.589974151	6.342908877	-1.571959123
86454	chr3	55248012	55248512	501	*	5.509425899	4.38388959	6.133918378	-1.555582923
71853	chr2	3579239	3579739	501	*	5.634435878	4.55258897	6.245680368	-1.518844477
69494	chr19	27563608	27564108	501	*	5.038417968	3.550775171	5.755106285	-1.900117044
32046	chr12	105521347	105521847	501	*	5.483546366	4.33624354	6.114447115	-1.584156787
32372	chr12	110278862	110279362	501	*	4.589664607	2.492654347	5.41036059	-2.3303344
106787	chr5	37897242	37897742	501	*	4.515662934	2.185134605	5.364612265	-2.510551102
9456	chr1	186658817	186659317	501	*	5.623840274	4.558323378	6.229996835	-1.503734643
39481	chr14	23902482	23902982	501	*	5.296616594	4.027956731	5.961058352	-1.689695204
142210	chr8	116144141	116144641	501	*	4.665762732	5.41133177	3.036996163	1.973150258
80130	chr2	126425140	126425640	501	*	4.969900983	3.381487294	5.707555825	-1.94827791
15994	chr10	90903573	90904073	501	*	4.991854934	3.494642908	5.710611278	-1.904216472
46501	chr15	71853160	71853660	501	*	5.190354066	5.834522926	3.996591754	1.627205375
105197	chr5	25666043	25666543	501	*	4.861108953	3.214521869	5.61008977	-1.994797285
140660	chr8	88526265	88526765	501	*	4.759166758	5.484274686	3.232073314	1.92015841
42593	chr15	9184645	9185145	501	*	5.223359131	5.870693841	4.018221846	1.612761135
91677	chr3	153276028	153276528	501	*	4.117771103	4.97099776	1.747902047	2.520765982
99621	chr4	129270186	129270686	501	*	5.266478746	3.942257667	5.945123328	-1.750346485
78164	chr2	93451505	93452005	501	*	5.473049863	4.326236751	6.103807807	-1.57032167
90435	chr3	129445819	129446319	501	*	4.230208303	5.047647406	2.157573607	2.316459691
105241	chr5	28047944	28048444	501	*	4.916386224	3.373174556	5.644854679	-1.932159201
81569	chr2	148364501	148365001	501	*	4.660614499	2.813866597	5.444684255	-2.167421888

134579	chr7	104507675	104508175	501	*	4.309024804	5.126792189	2.233954192	2.303627213
107070	chr5	45475834	45476334	501	*	4.823312748	3.183177924	5.57106406	-2.00594993
32731	chr12	112861830	112862330	501	*	5.311036395	4.072196573	5.967566393	-1.662643069
87370	chr3	83541163	83541663	501	*	4.684407306	2.878728758	5.461735299	-2.124959483
28820	chr12	45733620	45734120	501	*	4.617257178	5.352642383	3.040170761	1.95103235
39002	chr14	11618057	11618557	501	*	5.380403776	5.991071654	4.300419762	1.504758121
63487	chr17	81669098	81669598	501	*	5.003049969	3.524058304	5.717854249	-1.872875797
19709	chr11	45080123	45080623	501	*	5.028185012	3.58274288	5.735551111	-1.850145852
64603	chr18	13328282	13328782	501	*	4.378797152	5.157636127	2.564031698	2.129776241
92073	chr4	7215131	7215631	501	*	4.705645025	2.872755393	5.487464793	-2.139583798
55211	chr16	38073894	38074394	501	*	4.492796843	2.266115931	5.329811874	-2.406078273
55567	chr16	45889567	45890067	501	*	5.197133863	3.920433485	5.863673772	-1.690706662
32271	chr12	108915880	108916380	501	*	5.31182066	4.061633793	5.97138564	-1.656999901
148771	chr9	98668350	98668850	501	*	4.560982215	5.315787909	2.883351995	2.028231716
133807	chr7	92696300	92696800	501	*	5.115283203	3.745606477	5.805049362	-1.766438909
140955	chr8	93871240	93871740	501	*	5.309383137	5.929955026	4.196956679	1.525892201
113707	chr5	139681784	139682284	501	*	5.502151987	4.322006835	6.142488702	-1.611620282
5363	chr1	127961066	127961566	501	*	4.936199194	3.385846511	5.666142064	-1.908839888
77147	chr2	74243696	74244196	501	*	4.594076922	5.332860907	2.999987929	1.938278723
74917	chr2	33223656	33224156	501	*	4.772769978	3.128093796	5.521387347	-2.00424498
104302	chr5	4355870	4356370	501	*	5.043188392	5.713465036	3.752026846	1.680071091
54140	chr16	25016363	25016863	501	*	5.068689422	3.701940698	5.757752244	-1.762624447
117840	chr6	38710423	38710923	501	*	4.824955811	5.520492996	3.430972192	1.79953655
149665	chr9	111460400	111460900	501	*	4.361282369	1.51214424	5.257531688	-2.797305867
33980	chr13	30198233	30198733	501	*	4.422162002	2.185498435	5.260366359	-2.417173731
91940	chr4	5088190	5088690	501	*	4.829854283	3.150990955	5.584888298	-2.006055805
92736	chr4	21969041	21969541	501	*	4.943757489	3.432181877	5.665586866	-1.898880282
34893	chr13	44293721	44294221	501	*	4.440097498	2.170416654	5.28217061	-2.426552302
146691	chr9	61580986	61581486	501	*	5.234149205	3.982295115	5.894157572	-1.67579305
60337	chr17	31456675	31457175	501	*	4.926141544	3.407135239	5.649546181	-1.891136049
18673	chr11	12513590	12514090	501	*	4.558133585	2.646154185	5.35246401	-2.199013967
67205	chr18	75157549	75158049	501	*	4.755172427	3.115215566	5.502889735	-1.989406375
115012	chr5	151046793	151047293	501	*	5.243957087	5.874929985	4.096406762	1.55796193
87319	chr3	81667406	81667906	501	*	5.256141981	4.013025643	5.913819369	-1.665183864
89581	chr3	108379234	108379734	501	*	4.900101712	3.392203136	5.621147934	-1.870400718
75950	chr2	50347530	50348030	501	*	4.343824359	1.674214858	5.225747969	-2.661278437
3484	chr1	84518253	84518753	501	*	5.054948868	3.644231651	5.754389758	-1.789738466
28624	chr12	37769778	37770278	501	*	4.064542488	4.927860314	1.596871554	2.528698154
42222	chr15	4642754	4643254	501	*	3.935940258	4.82406326	1.191582213	2.730148882
73751	chr2	28225828	28226328	501	*	5.230006481	3.992045743	5.886300087	-1.640415867
99789	chr4	130060932	130061432	501	*	4.883497917	3.375527859	5.604559386	-1.887070355
78767	chr2	106425558	106426058	501	*	4.236685921	5.041483389	2.254462274	2.22546138
41427	chr14	72884454	72884954	501	*	4.403762851	2.150804648	5.243888873	-2.39745843
20343	chr11	54884895	54885395	501	*	4.813672478	3.245336901	5.547289568	-1.92858706
94166	chr4	49682916	49683416	501	*	4.619313612	2.829487225	5.393978951	-2.110218254
24866	chr11	107512879	107513379	501	*	5.374498753	4.187834419	6.016675882	-1.593215488
70411	chr19	41935884	41936384	501	*	5.165283122	3.875632435	5.835171564	-1.693168063
70427	chr19	42022326	42022826	501	*	5.001163736	3.573357148	5.704534185	-1.816103219
66114	chr18	56038240	56038740	501	*	5.122448638	3.807263374	5.798829899	-1.719883442
80925	chr2	135537933	135538433	501	*	5.369333021	4.221747965	6.000316042	-1.569295539
89192	chr3	103944798	103945298	501	*	4.597772737	2.742555286	5.383205273	-2.158050346

Table S2. ATAC-seq in D5 in mDPCs-CAS9 cells and sgCreb3l1_A8_4: 888 NFRs enriched and 510 NFRs lost at D5_Ctrl

98942	chr4	123239512	123240012	501	*	5.542324335	4.450376994	6.156678259	-1.502178157
65823	chr18	47562485	47562985	501	*	4.293416463	5.101175266	2.290586129	2.213494301
33050	chr13	5714257	5714757	501	*	4.764443652	3.175644603	5.502175333	-1.941582562
42919	chr15	12243909	12244409	501	*	4.930410406	5.598770914	3.646687094	1.680081578
4099	chr1	90538807	90539307	501	*	5.151022352	3.886216808	5.814453093	-1.666965316
148220	chr9	84050820	84051320	501	*	4.34886432	2.014250844	5.198263853	-2.444278238
34540	chr13	38347657	38348157	501	*	4.980285528	3.506556181	5.693937033	-1.868528692
10628	chr10	13916932	13917432	501	*	5.33735468	4.133025534	5.984465523	-1.611224104
107661	chr5	65162908	65163408	501	*	4.388968092	2.1305445	5.229733247	-2.385608672
46808	chr15	73886702	73887202	501	*	4.426779932	2.261723985	5.256245142	-2.326494506
58703	chr17	24047984	24048484	501	*	4.423095027	2.289232131	5.248598215	-2.321596443
58064	chr17	8856281	8856781	501	*	4.070910535	4.90490248	1.869261125	2.370619743
38735	chr13	114267765	114268265	501	*	5.148715717	3.772248114	5.840106323	-1.773490216
101692	chr4	139087360	139087860	501	*	5.050598755	3.70530583	5.734453217	-1.735373113
120214	chr6	55618026	55618526	501	*	4.753946736	3.110338536	5.502360728	-1.982388495
125463	chr6	98845948	98846448	501	*	4.482068946	5.234204453	2.818767779	1.99338641
81543	chr2	148162156	148162656	501	*	4.763725147	3.052543268	5.524661603	-2.038413941
31070	chr12	85157692	85158192	501	*	4.286855006	1.573466762	5.172451605	-2.710670121
12447	chr10	45888740	45889240	501	*	3.886023699	4.786568702	0.978003162	2.79164151
27245	chr12	15274020	15274520	501	*	3.87910416	4.793088056	0.768294007	2.908972294
29984	chr12	73507147	73507647	501	*	5.534387162	4.4606542	6.143113038	-1.501274252
84808	chr3	3388309	3388809	501	*	4.529166733	5.275440106	2.896742688	1.950396247
29954	chr12	73393938	73394438	501	*	5.513763918	4.402823429	6.13388849	-1.534778929
35068	chr13	45931157	45931657	501	*	5.350368356	4.196275004	5.983242854	-1.559499082
69294	chr19	23919992	23920492	501	*	4.985455558	3.59004286	5.681328312	-1.785384385
41268	chr14	69692418	69692918	501	*	5.455627974	4.355906737	6.072355806	-1.514882889
75727	chr2	44093862	44094362	501	*	4.59647394	2.826516576	5.367753245	-2.078222304
847	chr1	36965783	36966283	501	*	5.277359227	5.897056211	4.167837305	1.505952323
77739	chr2	91027374	91027874	501	*	4.621914428	2.838642919	5.395468726	-2.053729774
22650	chr11	82943290	82943790	501	*	4.992803961	5.643473168	3.775560617	1.615216614
92004	chr4	6259321	6259821	501	*	4.653118834	2.935078499	5.415287904	-2.010903706
151921	chrX	98380419	98380919	501	*	4.053004643	4.896735839	1.768910772	2.362173665
7743	chr1	163607704	163608204	501	*	5.17424072	3.89213481	5.842182927	-1.680294061
16981	chr10	113718053	113718553	501	*	4.512465657	5.25238561	2.912639926	1.925217787
13512	chr10	67877264	67877764	501	*	4.425280443	2.338558728	5.244609352	-2.297963415
152493	chrX	135574189	135574689	501	*	4.227063087	5.032184842	2.242599214	2.203737112
16090	chr10	93130390	93130890	501	*	4.36793305	2.209574229	5.196555734	-2.341209418
88002	chr3	89210562	89211062	501	*	5.085508629	3.812248073	5.751152729	-1.675896968
15517	chr10	83343733	83344233	501	*	4.95166361	5.604724547	3.72565655	1.626216059
41887	chr14	103260816	103261316	501	*	4.563516694	2.691669884	5.351598104	-2.120041445
99457	chr4	128694330	128694830	501	*	5.469149013	4.382862089	6.081763876	-1.511795381
96146	chr4	88427572	88428072	501	*	4.735653937	3.180488672	5.466585514	-1.901596694
105201	chr5	25700784	25701284	501	*	5.030671069	3.708789694	5.708731294	-1.703789454
2004	chr1	59566693	59567193	501	*	5.08430394	5.717274015	3.929880713	1.571962737
110290	chr5	112958126	112958626	501	*	4.468137381	2.539222947	5.265045143	-2.179204266
134583	chr7	104861308	104861808	501	*	4.779194209	5.461657393	3.439566104	1.712411209
62593	chr17	66602257	66602757	501	*	4.675641363	2.955324685	5.438218017	-2.01256747
115696	chr6	17906264	17906764	501	*	4.97263626	3.601196775	5.662825	-1.767718467
95822	chr4	84109721	84110221	501	*	5.528753205	4.44029873	6.142035062	-1.500210398
115823	chr6	22821014	22821514	501	*	4.691832924	5.400866813	3.238950816	1.802231644
89566	chr3	108206880	108207380	501	*	4.564325075	2.816863382	5.331704173	-2.048197444

100973	chr4	135879034	135879534	501	*	5.385855394	4.189172843	6.030840054	-1.587312937
117903	chr6	38893542	38894042	501	*	5.046144402	3.758988792	5.715390636	-1.676240857
135916	chr7	129918451	129918951	501	*	4.306708901	5.080862825	2.519904062	2.040359716
95667	chr4	82572950	82573450	501	*	5.324897714	4.140690301	5.966382487	-1.590211819
147318	chr9	66251029	66251529	501	*	5.005313653	3.679652903	5.684317215	-1.712005119
71611	chr19	57803183	57803683	501	*	4.942968754	3.58274288	5.630458334	-1.744092583
94893	chr4	59585044	59585544	501	*	5.193181743	3.97239501	5.844820215	-1.616497789
107914	chr5	72442723	72443223	501	*	5.156928302	5.792267281	3.994297823	1.541938272
87018	chr3	66739716	66740216	501	*	4.812275945	3.323061107	5.529305145	-1.853129872
137273	chr8	10499788	10500288	501	*	4.699619232	5.398250554	3.292392026	1.785710122
68949	chr19	14240162	14240662	501	*	4.935241359	3.520211607	5.635679289	-1.807501
143064	chr8	122658183	122658683	501	*	5.152998336	5.769579344	4.053759496	1.505237836
89916	chr3	118443698	118444198	501	*	4.406811007	2.432181877	5.210504821	-2.193613663
86350	chr3	53564407	53564907	501	*	4.523734711	2.698440235	5.304310647	-2.083337567
108926	chr5	99190780	99191280	501	*	4.618665833	2.960966426	5.369748875	-1.962503892
138067	chr8	26255046	26255546	501	*	4.948719333	3.500684941	5.656667725	-1.824870244
12527	chr10	53765458	53765958	501	*	4.290526421	5.06638373	2.493628762	2.059136465
81234	chr2	142838135	142838635	501	*	5.251199359	4.085462808	5.887430343	-1.574430895
4717	chr1	97656029	97656529	501	*	4.889128679	3.456531513	5.593590455	-1.789575417
146256	chr9	58036748	58037248	501	*	4.392536046	2.402683508	5.198435487	-2.182624273
81643	chr2	150257213	150257713	501	*	4.775300684	5.452516212	3.456793024	1.691088012
4764	chr1	105431810	105432310	501	*	4.446591615	5.184873165	2.855031269	1.899524169
7324	chr1	159054953	159055453	501	*	4.408246568	2.427960643	5.212763074	-2.186463186
41946	chr14	106075115	106075615	501	*	4.702332422	3.160643977	5.430485234	-1.867735323
75691	chr2	38990124	38990624	501	*	4.835688907	3.373174556	5.546866447	-1.82051952
35573	chr13	52116403	52116903	501	*	5.345624178	4.234746177	5.965729926	-1.526428659
143284	chr8	124259126	124259626	501	*	4.390245288	5.131907179	2.781569217	1.931182386
63439	chr17	80834657	80835157	501	*	5.162654795	3.978075531	5.804244452	-1.588683232
138190	chr8	28148533	28149033	501	*	3.769676442	4.677760851	0.751649674	2.772041219
138411	chr8	35615798	35616298	501	*	4.759969366	5.45006775	3.388907019	1.737978117
125418	chr6	98342463	98342963	501	*	4.860266273	3.38802119	5.573591703	-1.802933112
34390	chr13	35879000	35879500	501	*	4.831977615	3.430072804	5.52936966	-1.77084977
137606	chr8	13620199	13620699	501	*	5.014363644	5.655937308	3.829841089	1.582342026
28504	chr12	34990406	34990906	501	*	4.314093569	5.091334798	2.508934792	2.028956198
140058	chr8	81776912	81777412	501	*	4.332272564	5.088102036	2.649103367	1.951830627
13632	chr10	69253261	69253761	501	*	4.754322975	3.286903198	5.466585514	-1.804279372
41787	chr14	99456715	99457215	501	*	3.973704604	4.795381417	1.869261125	2.251048592
92852	chr4	30450398	30450898	501	*	4.827122738	3.357884894	5.539786259	-1.811521574
4743	chr1	98280425	98280925	501	*	3.765973669	4.661152143	0.931120779	2.647508299
1030	chr1	39312518	39313018	501	*	4.975938898	3.667302046	5.650668771	-1.685921361
93781	chr4	45755840	45756340	501	*	4.61468587	5.310148466	3.221019663	1.755458072
67699	chr18	84700369	84700869	501	*	4.907089554	3.582604762	5.585799926	-1.700392116
142897	chr8	122021688	122022188	501	*	4.624940472	5.319614714	3.234623835	1.734156797
140935	chr8	93626709	93627209	501	*	4.390179924	5.128009161	2.800891437	1.892137803
50182	chr15	88502221	88502721	501	*	3.999562231	4.827038961	1.850256623	2.261416311
90453	chr3	129680242	129680742	501	*	4.180903417	1.701940698	5.045338539	-2.459724515
38783	chr13	115199134	115199634	501	*	4.253373918	2.041332642	5.088628043	-2.311503832
107757	chr5	66219155	66220089	935	*	5.289144488	4.163278086	5.913735088	-1.514844283
43541	chr15	30573563	30574063	501	*	3.829256713	4.710578314	1.166687173	2.555534427
36967	chr13	76937731	76938231	501	*	4.277793503	2.06327156	5.113347422	-2.317992423
103226	chr4	150784971	150785471	501	*	4.84695336	3.423727022	5.549276227	-1.781957908

Table S2. ATAC-seq in D5 in mDPCs-CAS9 cells and sgCreb3l1_A8_4: 888 NFRs enriched and 510 NFRs lost at D5_Ctrl

126487	chr6	112281833	112282333	501	*	5.09797879	5.736180857	3.92534802	1.535302018
10621	chr10	13793394	13793894	501	*	5.158407265	3.930501277	5.811984012	-1.592515168
9386	chr1	185937527	185938027	501	*	4.939298722	5.578437214	3.763377734	1.55273599
109196	chr5	103576743	103577243	501	*	4.838945244	3.450452683	5.53318922	-1.73135842
72039	chr2	5515049	5515549	501	*	4.356866415	2.419480941	5.155050369	-2.137224173
82279	chr2	155806494	155806994	501	*	4.578135911	5.273217161	3.18609117	1.750276345
34178	chr13	32643283	32643783	501	*	4.638644796	3.073724131	5.371568395	-1.900027795
98608	chr4	119866226	119866726	501	*	5.133184127	3.945129678	5.775752319	-1.563439632
86650	chr3	58317723	58318223	501	*	4.459725132	2.698440235	5.229509286	-2.010373621
51304	chr15	99010887	99011387	501	*	4.792741262	3.386129624	5.491229543	-1.772647068
95086	chr4	62998431	62998931	501	*	5.312122949	4.191555155	5.935135002	-1.522963785
73328	chr2	26556176	26556676	501	*	4.926464169	3.633778564	5.59713189	-1.670656139
94336	chr4	55415781	55416281	501	*	5.09613617	3.829487225	5.760050874	-1.657039565
136045	chr7	130972671	130973171	501	*	4.821231142	5.478747872	3.578714572	1.611140259
56108	chr16	63161531	63162031	501	*	4.794244672	3.371137304	5.496540274	-1.779082097
137622	chr8	13673115	13673615	501	*	4.830139654	5.479183737	3.618812592	1.601922739
120821	chr6	67105340	67105840	501	*	5.019088008	5.664281834	3.82165551	1.575138918
9973	chr1	194456249	194456749	501	*	3.740530409	4.630108533	0.978003162	2.59272901
34101	chr13	31747409	31747909	501	*	5.067728654	3.82788614	5.724528062	-1.63269031
28876	chr12	51117804	51118304	501	*	4.038229043	4.833390645	2.12081317	2.134338935
46338	chr15	67354784	67355284	501	*	4.421508738	2.628403825	5.196727571	-2.036117751
138621	chr8	42069299	42069799	501	*	4.646957152	5.336284501	3.279108064	1.70772558
29410	chr12	59582553	59583053	501	*	3.911341718	4.748981469	1.679426837	2.318786553
89788	chr3	115848968	115849468	501	*	4.92137144	3.595562153	5.600412014	-1.699896516
95807	chr4	84000712	84001212	501	*	4.459150494	2.729143731	5.223453719	-1.992437587
43549	chr15	30785896	30786396	501	*	3.973919164	4.794608963	1.876955632	2.21955988
46448	chr15	69107890	69108390	501	*	5.0011765	5.622480131	3.886314933	1.507398783
75760	chr2	44976461	44976961	501	*	4.958188813	3.578802039	5.650274808	-1.72727992
70465	chr19	42193305	42193805	501	*	4.820973509	3.377274125	5.527947419	-1.762057034
60528	chr17	33030877	33031377	501	*	4.806914181	3.366572918	5.513130737	-1.785123028
129333	chr6	142548318	142548818	501	*	3.989619053	4.791398918	2.02805263	2.160166742
152393	chrX	113724062	113724562	501	*	3.781544584	4.719052153	0.221470052	2.957638744
46813	chr15	73897628	73898128	501	*	4.884300811	3.554541337	5.564323937	-1.680792619
140337	chr8	84737508	84738008	501	*	4.937250364	5.566312484	3.796236268	1.530040503
56513	chr16	84919911	84920411	501	*	4.923374712	3.575128116	5.607951902	-1.702213914
36245	chr13	60222575	60223075	501	*	4.365708418	2.547029084	5.145194787	-2.052057952
120654	chr6	65888739	65889239	501	*	4.917199129	3.589797067	5.596636287	-1.666609218
76910	chr2	71499440	71499940	501	*	5.068414765	3.866506323	5.714854074	-1.58868252
11682	chr10	36506635	36507135	501	*	3.971719026	4.77771458	1.981198907	2.148814434
90444	chr3	129611561	129612061	501	*	4.16453987	1.688401121	5.028696468	-2.418398814
91195	chr3	145099283	145099783	501	*	4.721257728	3.286903198	5.42611873	-1.762509078
87013	chr3	66688857	66689357	501	*	4.845230142	3.454759642	5.539940647	-1.727588181
76867	chr2	71031519	71032019	501	*	4.200748106	2.025063331	5.031541463	-2.252060275
80319	chr2	128338984	128339484	501	*	5.252984467	4.093383774	5.887450964	-1.551660553
80503	chr2	130353843	130354343	501	*	4.668898149	3.158283109	5.390523172	-1.814339694
104387	chr5	5359854	5360354	501	*	4.190813841	1.991963735	5.02446404	-2.246657022
87592	chr3	85846170	85846670	501	*	4.11214885	0.694931258	5.042993321	-2.849380421
98865	chr4	121216137	121216637	501	*	4.200797129	1.98623333	5.036356101	-2.250539465
115821	chr6	22802269	22802769	501	*	3.874402637	4.706177585	1.690805441	2.230140877
55735	chr16	51758317	51758817	501	*	3.693800252	4.580773805	0.963621878	2.538702689
30738	chr12	80691518	80692018	501	*	4.816512315	3.466556425	5.501506908	-1.695885575

32982	chr13	3769942	3770442	501	*	4.393831435	2.54341518	5.178492582	-2.06794364
105278	chr5	28418916	28419416	501	*	4.819208215	3.456650372	5.507261255	-1.730127583
77546	chr2	84410133	84410633	501	*	4.610133932	3.055083512	5.341041962	-1.859461142
21707	chr11	72503358	72503858	501	*	4.297813323	2.32533244	5.101193739	-2.145297607
36236	chr13	60130923	60131423	501	*	4.772339752	3.368937006	5.47008115	-1.746905306
94751	chr4	58332394	58332894	501	*	4.366007197	2.55837876	5.143660356	-2.045371975
79492	chr2	118775321	118775821	501	*	4.324375566	2.415532526	5.11822483	-2.086537241
111047	chr5	117790012	117790512	501	*	3.914030079	4.728969738	1.859790166	2.17316838
72017	chr2	5241457	5241957	501	*	4.106236588	1.729143731	4.960234706	-2.365535389
120685	chr6	66397286	66397786	501	*	4.926107448	5.556398433	3.780894149	1.534395844
44720	chr15	44188626	44189126	501	*	4.318126743	2.419790227	5.11035534	-2.080576381
64871	chr18	25025754	25026254	501	*	5.003369557	3.735740883	5.66754116	-1.64340342
92078	chr4	7379980	7380480	501	*	5.033044573	3.843380893	5.676064866	-1.561001829
101078	chr4	136383726	136384226	501	*	4.644110107	3.091973942	5.374419844	-1.838492911
23256	chr11	92889998	92890498	501	*	4.168535912	4.928510296	2.462680545	1.937769079
140520	chr8	86967876	86968376	501	*	4.460645655	2.778556423	5.21627588	-1.927312862
81931	chr2	152907539	152908039	501	*	4.767196813	3.409278088	5.454127816	-1.698969551
140966	chr8	93946360	93946860	501	*	4.478322467	5.174385216	3.082099873	1.730354738
34887	chr13	44178150	44178650	501	*	4.749049614	3.364526316	5.442354986	-1.728173876
68332	chr19	6384816	6385316	501	*	4.183292086	2.019463154	5.012603261	-2.225804569
120324	chr6	56842168	56842668	501	*	4.818380544	3.496659732	5.496400624	-1.682312408
129269	chr6	140906477	140906977	501	*	4.253384559	2.293878747	5.054860685	-2.111432875
18757	chr11	17727402	17727902	501	*	3.920583236	4.753328069	1.729119805	2.232387817
39522	chr14	24510647	24511147	501	*	4.246691865	2.219377464	5.057906969	-2.177355717
96498	chr4	97618504	97619004	501	*	4.058840042	4.837996775	2.242155095	2.053470309
21905	chr11	75289913	75290413	501	*	4.07617212	1.472897548	4.952294503	-2.443070053
3226	chr1	78939822	78940322	501	*	3.845251366	4.676162602	1.668618952	2.249139826
3352	chr1	82116384	82116884	501	*	4.12738998	1.86340767	4.968802415	-2.276715694
102415	chr4	141928737	141929237	501	*	4.901457441	5.536335459	3.740428719	1.527726901
145713	chr9	51105672	51106172	501	*	4.094304369	1.653196641	4.95495559	-2.351350446
102840	chr4	149373753	149374253	501	*	4.802963365	3.470666386	5.483615888	-1.671935268
129302	chr6	142188476	142188976	501	*	4.354755735	2.528007894	5.135570274	-2.028419457
121584	chr6	80830345	80830845	501	*	3.916075714	4.726568249	1.893919258	2.145367736
76215	chr2	53411761	53412261	501	*	3.917335737	4.731429531	1.869261125	2.15950561
98968	chr4	123402215	123402715	501	*	4.996306262	3.807381576	5.639119021	-1.554343253
37127	chr13	90546534	90547034	501	*	4.364708744	2.624720077	5.130776317	-1.970276309
91120	chr3	144261667	144262167	501	*	4.32392201	2.492654347	5.105476793	-2.01266507
41909	chr14	105255568	105256068	501	*	4.592425493	5.284053717	3.214961218	1.702440219
78766	chr2	106424869	106425369	501	*	4.48774296	5.178696896	3.113104719	1.697202157
47123	chr15	75609081	75609581	501	*	4.92949476	3.700191528	5.583450492	-1.580475759
36145	chr13	58560735	58561235	501	*	4.760394442	3.392485004	5.449736291	-1.72029272
41533	chr14	75834409	75834909	501	*	3.589865733	0	4.589865733	-3.176396794
33932	chr13	29550545	29551045	501	*	4.121053999	1.899553778	4.957448166	-2.251873461
145935	chr9	55067509	55068009	501	*	4.267883567	2.402996416	5.054860685	-2.035418794
36714	chr13	71050568	71051068	501	*	4.248164901	2.316225571	5.045529364	-2.087818729
143524	chr8	126644116	126644616	501	*	4.450408278	5.155815724	3.01364407	1.742996702
137431	chr8	11600327	11600827	501	*	4.600453008	5.270281939	3.311033761	1.643087251
84809	chr3	3389164	3389664	501	*	3.759335143	4.602948315	1.476272532	2.267200881
27913	chr12	25991663	25992163	501	*	3.828367189	4.647984029	1.739485414	2.160220649
89072	chr3	102384544	102385044	501	*	4.002531423	1.293541251	4.887764461	-2.48126525
86387	chr3	54508939	54509439	501	*	4.13012921	1.722265534	4.987366045	-2.34816184

Table S2. ATAC-seq in D5 in mDPCs-CAS9 cells and sgCreb3l1_A8_4: 888 NFRs enriched and 510 NFRs lost at D5_Ctrl

43141	chr15	25482561	25483061	501	*	4.980328263	3.767123028	5.629889176	-1.570537866
62354	chr17	62323319	62323819	501	*	4.946848688	3.660383625	5.615916935	-1.63463896
84200	chr2	170772012	170772512	501	*	4.904648858	3.640958095	5.567786547	-1.627889156
75839	chr2	46465283	46465783	501	*	4.584670907	3.081611899	5.304682674	-1.817941418
17028	chr10	116072611	116073111	501	*	4.402543573	5.134490056	2.842418001	1.816542247
58543	chr17	18558154	18558654	501	*	3.754053014	4.597320544	1.474005799	2.26082195
143822	chr9	9196004	9196504	501	*	4.56193628	3.06559536	5.280505057	-1.805552844
80029	chr2	125641818	125642318	501	*	4.530601547	3.003313071	5.25575041	-1.833191444
53087	chr16	12032436	12032936	501	*	4.063296928	4.851066308	2.1934206	2.011831173
41094	chr14	65746896	65747396	501	*	4.681659184	3.282368146	5.378440566	-1.740606216
24989	chr11	109329247	109329747	501	*	4.945667619	3.760546056	5.587410175	-1.547282967
24026	chr11	99127736	99128236	501	*	4.703160611	5.354754892	3.482535665	1.585981317
126439	chr6	109276395	109276895	501	*	4.103883915	4.859596786	2.421346842	1.91753764
76841	chr2	70399279	70399779	501	*	4.311746539	5.026612294	2.832473581	1.784794889
11676	chr10	34904731	34905231	501	*	4.092042784	4.865839791	2.307342323	1.993949782
91121	chr3	144264387	144264887	501	*	4.136643139	2.009019634	4.961342163	-2.169561434
79321	chr2	117866706	117867206	501	*	4.40458784	2.728894141	5.159034503	-1.914099053
61953	chr17	52616802	52617302	501	*	4.319966125	5.041483389	2.809857296	1.791339344
38343	chr13	108526325	108526825	501	*	4.529258942	2.992127746	5.256464991	-1.832476416
129265	chr6	140852352	140852852	501	*	4.393328148	2.745703755	5.142506019	-1.906776187
70192	chr19	38298214	38298714	501	*	4.810908101	3.537407323	5.476614848	-1.626800611
139999	chr8	79559465	79559965	501	*	4.331775029	5.049559482	2.839069776	1.793513334
138195	chr8	31031980	31032480	501	*	4.615048686	5.276070044	3.359376378	1.60610649
11680	chr10	35586181	35586681	501	*	3.640034786	4.511219514	1.090715535	2.360672107
33375	chr13	16275015	16275515	501	*	5.020446394	3.832476333	5.662990859	-1.543241633
89999	chr3	121402064	121402564	501	*	4.573719334	5.24413693	3.282008723	1.618328949
31390	chr12	91730822	91731322	501	*	4.565923283	3.076227385	5.283056706	-1.800935379
46094	chr15	64355927	64356427	501	*	4.853796685	3.597334999	5.515027046	-1.589013293
79236	chr2	117010155	117010655	501	*	4.730266393	3.407135239	5.408638907	-1.651905319
139080	chr8	64841942	64842442	501	*	4.843898371	5.478266906	3.684637351	1.509996333
115797	chr6	22378420	22378920	501	*	4.474086499	5.160183176	3.119605363	1.672735842
150063	chr9	120555880	120556380	501	*	4.205132501	2.320786192	4.995181789	-2.041508633
105617	chr5	31876524	31877024	501	*	4.737506827	5.378034868	3.55668571	1.546367589
97509	chr4	107641529	107642029	501	*	4.210236223	2.338558728	4.998290841	-2.043181494
32184	chr12	108294591	108295091	501	*	4.44675049	2.881818449	5.179676403	-1.827528845
143118	chr8	122905276	122905776	501	*	4.327816127	5.04021243	2.859790166	1.758554869
18806	chr11	19423740	19424240	501	*	3.811579958	4.647156387	1.596871554	2.206235056
44456	chr15	40038386	40038886	501	*	4.196340198	4.932089444	2.617444099	1.834306475
76002	chr2	51117934	51118434	501	*	4.950960811	3.762223601	5.593720898	-1.543873732
125125	chr6	94574554	94575054	501	*	4.629779411	3.231047202	5.326430055	-1.70624927
23147	chr11	88901381	88901881	501	*	4.194273027	2.2845705	4.988254268	-2.041771488
29413	chr12	59836490	59836990	501	*	4.34558848	5.070451621	2.81966066	1.797533629
29993	chr12	73536085	73536585	501	*	4.365779996	2.729143731	5.112861899	-1.881450609
85613	chr3	33082836	33083336	501	*	3.92651962	4.722453593	2.004030658	2.059029534
31955	chr12	104593610	104594110	501	*	4.614464386	3.150618317	5.325936926	-1.747241186
120830	chr6	67125533	67126033	501	*	4.677812989	5.337287463	3.42796614	1.575533579
111002	chr5	117182220	117182720	501	*	4.797471238	3.500423441	5.469255442	-1.619254212
134581	chr7	104588099	104588599	501	*	3.664857372	4.513213313	1.339693943	2.265634467
13879	chr10	73028697	73029197	501	*	3.635089526	4.513197989	1.009476321	2.413084465
58589	chr17	22884225	22884725	501	*	3.995367987	4.77444111	2.179188391	1.984227102
3493	chr1	84668372	84668872	501	*	4.49189339	2.969704778	5.215969655	-1.797283703

91453	chr3	148682336	148682836	501	*	3.952332103	1.100108026	4.848811256	-2.521679934
25130	chr11	111961826	111962326	501	*	4.365445831	2.705432688	5.116964169	-1.886114009
92769	chr4	25200003	25200503	501	*	4.811904514	3.577035578	5.467365253	-1.578485212
19602	chr11	43751975	43752475	501	*	4.994144161	3.780092719	5.643937651	-1.552756573
50728	chr15	95489135	95489635	501	*	4.48608726	2.969493524	5.208981525	-1.791545748
27182	chr12	11196206	11196706	501	*	4.185370112	4.919627398	2.613873409	1.820433762
92782	chr4	26323774	26324274	501	*	4.550735028	3.115215566	5.255860342	-1.735491178
105592	chr5	31673404	31673904	501	*	4.937858105	3.757429846	5.578274965	-1.532211184
90491	chr3	130524673	130525173	501	*	3.564942883	4.455629903	0.788401748	2.471299968
109356	chr5	105110291	105110791	501	*	4.022042792	4.788156387	2.281792664	1.94159373
71561	chr19	57243433	57243933	501	*	4.705447521	3.351699137	5.391366034	-1.66334629
81979	chr2	153179929	153180429	501	*	4.962551923	3.737225586	5.615427744	-1.563463722
1083	chr1	39846060	39846560	501	*	4.927002738	3.770460936	5.560585973	-1.515691738
9731	chr1	191529401	191529901	501	*	4.704448647	5.353764005	3.492136156	1.540577089
13448	chr10	67297464	67297964	501	*	4.435059612	2.869419783	5.168129421	-1.815481667
70016	chr19	36194958	36195458	501	*	4.969753369	3.824912189	5.599935642	-1.509370782
29770	chr12	71354788	71355288	501	*	4.765857085	3.46078861	5.439683119	-1.651101807
39460	chr14	23522683	23523183	501	*	4.754171489	3.472866033	5.42190646	-1.613477532
30318	chr12	77126353	77126853	501	*	4.324861468	2.624720077	5.08379912	-1.902917985
27157	chr12	10411388	10411888	501	*	4.657221047	5.302215546	3.460503231	1.542003322
103299	chr4	151132443	151132943	501	*	4.755997822	3.480593893	5.422200422	-1.612269435
103526	chr4	152221553	152222053	501	*	4.619564319	3.250126829	5.309273077	-1.683640388
61955	chr17	52712269	52712769	501	*	3.791871414	4.601143012	1.778382909	2.0943802
65821	chr18	47330197	47330697	501	*	3.936853858	4.712202038	2.142981519	1.987054539
4900	chr1	111833763	111834263	501	*	3.943235889	0.694931258	4.865254325	-2.616956445
92282	chr4	10909600	10910100	501	*	4.018038873	1.775427809	4.856947446	-2.225993416
109029	chr5	100587576	100588076	501	*	4.576456254	3.170600445	5.274768796	-1.704747942
138114	chr8	26813117	26813617	501	*	4.713410734	5.376971787	3.448109127	1.57105971
66736	chr18	64816601	64817101	501	*	3.931021168	1.100108026	4.8259023	-2.488781072
15980	chr10	89911796	89912296	501	*	3.688617713	4.519953623	1.508565548	2.17617296
95520	chr4	76766794	76767294	501	*	4.088487234	2.057818028	4.900170729	-2.100537887
85234	chr3	21500764	21501264	501	*	4.355378567	2.695186793	5.106930478	-1.870170855
129589	chr6	146254401	146254901	501	*	4.225484986	2.432181877	5.000737231	-1.965152587
139823	chr8	76601489	76601989	501	*	4.519408431	5.187870218	3.235293168	1.599383137
61529	chr17	46115430	46115930	501	*	3.710668727	4.542028161	1.530426866	2.183536739
19476	chr11	35307908	35308408	501	*	4.434675664	2.857370398	5.170104922	-1.812901047
98139	chr4	117052651	117053151	501	*	4.093283868	2.089456776	4.90118469	-2.099410012
13871	chr10	72484304	72484804	501	*	4.470143885	5.147077781	3.152758872	1.643732912
99700	chr4	129699769	129700269	501	*	4.531352894	3.073527574	5.241489079	-1.752874261
10748	chr10	17231409	17231909	501	*	4.810669542	3.580842954	5.464767107	-1.572507184
142191	chr8	115926259	115926759	501	*	4.65070231	5.306203949	3.415681745	1.547412891
63477	chr17	81513502	81514002	501	*	3.979349453	1.722265534	4.819958195	-2.204466483
76598	chr2	65010053	65010553	501	*	4.029968852	1.893888847	4.855756848	-2.141135163
34500	chr13	37918783	37919283	501	*	4.701383663	3.407135239	5.372451868	-1.6092219
108692	chr5	92708109	92708609	501	*	4.928151229	3.723764162	5.575278122	-1.525572793
109443	chr5	106378026	106378526	501	*	4.094272385	4.84355381	2.446102523	1.841539299
45276	chr15	56054758	56055258	501	*	4.37164816	2.81062773	5.103777289	-1.809567791
19528	chr11	35976531	35977031	501	*	4.078449961	2.052343803	4.889496068	-2.086797385
58071	chr17	9817297	9817797	501	*	3.555987479	4.431127726	0.963621878	2.335214839
63438	chr17	80825393	80825893	501	*	4.201156002	2.398688789	4.97794735	-1.942034206
89484	chr3	107801389	107801889	501	*	4.035323576	1.875406946	4.864142632	-2.1385908

104857	chr5	23035574	23036074	501	*	3.705858253	4.523914451	1.628640729	2.098730466
147195	chr9	65203353	65203853	501	*	4.152463969	2.135353813	4.962246718	-2.0592724
72561	chr2	16818201	16818701	501	*	4.097235043	2.150804648	4.896772165	-2.02322167
66966	chr18	67991025	67991525	501	*	3.961218959	1.708916246	4.801268145	-2.183515432
38680	chr13	113534799	113535299	501	*	4.358838819	2.768575799	5.096862185	-1.803877568
22507	chr11	80327308	80327808	501	*	4.623045683	3.259313641	5.31138201	-1.672544618
148543	chr9	92617322	92617822	501	*	4.84498546	3.672663838	5.483099399	-1.513060679
67602	chr18	82725165	82725665	501	*	4.145746844	2.298174031	4.929949739	-1.976400639
141262	chr8	103244094	103244594	501	*	4.40876013	5.093345552	3.060479851	1.640311667
120787	chr6	67014519	67015019	501	*	4.641872658	5.292667351	3.424171194	1.530142094
151254	chrX	51905296	51905796	501	*	3.967800778	1.742308005	4.80467364	-2.18126848
140264	chr8	84343756	84344256	501	*	4.017522962	4.768805782	2.358762218	1.866107114
118181	chr6	40431160	40431660	501	*	3.843797006	4.63823417	1.931120779	1.988164381
61194	chr17	43784485	43784985	501	*	4.805974022	3.624720077	5.446624511	-1.519803207
145974	chr9	55560917	55561417	501	*	4.777258129	3.556335539	5.428933703	-1.54429672
19103	chr11	28692815	28693315	501	*	3.750223048	4.565692551	1.692105912	2.102788917
144028	chr9	17099332	17099832	501	*	4.33072462	5.047747467	2.841539099	1.734624438
260	chr1	14716345	14716845	501	*	4.725528817	3.468612869	5.386879387	-1.58056227
137897	chr8	24212626	24213126	501	*	3.564690436	4.433235259	1.043338389	2.309390974
23903	chr11	98361115	98361615	501	*	4.642979066	3.320620572	5.32115855	-1.633249187
58083	chr17	10390709	10391209	501	*	4.679963237	5.326351764	3.478237638	1.521525151
62641	chr17	67340660	67341160	501	*	4.03631271	1.888201584	4.863637636	-2.120564654
86742	chr3	60129756	60130256	501	*	3.994830688	1.86964621	4.819214288	-2.104135825
103817	chr4	154496935	154497435	501	*	4.288954762	2.646154185	5.037214838	-1.859142983
87071	chr3	67719823	67720323	501	*	4.212278384	2.4240354	4.986675879	-1.935901919
86900	chr3	65455944	65456444	501	*	3.942337397	1.687887596	4.782638118	-2.161097041
98796	chr4	120725878	120726378	501	*	4.486821127	2.977804889	5.208105651	-1.730392797
20756	chr11	60153909	60154409	501	*	4.437169272	5.103641556	3.160728929	1.583874626
139347	chr8	70389597	70390097	501	*	4.372211763	5.066334342	2.984233335	1.648336017
11914	chr10	39629876	39630376	501	*	4.467106983	2.963885095	5.187153634	-1.757905562
2546	chr1	71916940	71917440	501	*	4.892895345	3.708663132	5.534387114	-1.512078084
77329	chr2	77502146	77502646	501	*	4.533159688	5.186712335	3.305342494	1.552391344
58377	chr17	14651838	14652338	501	*	4.748281317	3.464497046	5.416657579	-1.597012295
109089	chr5	101441701	101442201	501	*	4.133762542	4.854215747	2.628640729	1.751558965
147500	chr9	69181354	69181854	501	*	4.55806983	3.180488672	5.249725906	-1.663946214
127702	chr6	120978110	120978610	501	*	4.67325917	3.394368261	5.340368142	-1.580248695
118203	chr6	40781035	40781535	501	*	3.862595888	4.635424437	2.083587394	1.922003721
104398	chr5	5483192	5483692	501	*	4.352736266	2.797837041	5.083613291	-1.775507041
33193	chr13	9757892	9758392	501	*	4.089420887	2.185134605	4.882568928	-1.990925383
21725	chr11	72734837	72735337	501	*	4.250108319	2.591685246	5.001327598	-1.832658079
56282	chr16	73044159	73044659	501	*	3.925538786	1.701940698	4.762184667	-2.137788643
10710	chr10	16337532	16338032	501	*	4.115762654	2.316225571	4.89206313	-1.935250732
35770	chr13	54122187	54122687	501	*	4.305165341	2.725708732	5.041027231	-1.804958949
12404	chr10	44702076	44702576	501	*	4.496830624	3.084142778	5.196727571	-1.674905859
148539	chr9	92560663	92561163	501	*	4.471064819	3.038768048	5.175458294	-1.697183199
65488	chr18	38812396	38812896	501	*	3.86141385	1.257318629	4.73760979	-2.290399348
443	chr1	21836308	21836808	501	*	4.310910408	5.012218403	2.892104329	1.669956358
76216	chr2	53518302	53518802	501	*	3.422641076	4.322913232	0.518438177	2.416871953
93055	chr4	33701902	33702402	501	*	3.918325275	0.321117374	4.857454789	-2.599504813
136854	chr7	143305786	143306286	501	*	4.511875506	5.173124728	3.255342557	1.548076319
79942	chr2	124903487	124903987	501	*	4.249319754	2.580595588	5.002469527	-1.83352432

Table S2. ATAC-seq in D5 in mDPCs-CAS9 cells and sgCreb3l1_A8_4: 888 NFRs enriched and 510 NFRs lost at D5_Ctrl

28809	chr12	45074155	45074655	501	*	3.483657098	4.353764005	0.945828057	2.263325164
75679	chr2	38943187	38943687	501	*	4.420816246	2.937834018	5.136491296	-1.721425702
144716	chr9	34029419	34029919	501	*	4.464252863	3.025266603	5.170163214	-1.695027257
81044	chr2	137536547	137537047	501	*	3.973675324	1.929288469	4.787261198	-2.071181994
4744	chr1	98350102	98350602	501	*	4.386135524	5.057629798	3.090222102	1.600404262
145975	chr9	55586445	55586945	501	*	3.836671622	1.311983775	4.705534271	-2.268428443
44576	chr15	42252675	42253175	501	*	4.074633133	2.195104747	4.86392624	-1.97285698
132336	chr7	64839805	64840305	501	*	4.411297298	5.090107025	3.086414136	1.600140251
33944	chr13	29679738	29680238	501	*	4.674565002	3.404832974	5.339287391	-1.573048893
38592	chr13	112608391	112608891	501	*	4.367073695	2.857370398	5.088504599	-1.735451767
29575	chr12	69760008	69760508	501	*	4.544663365	3.187996808	5.231290744	-1.650750504
36081	chr13	56648003	56648503	501	*	4.865521656	3.681298151	5.507010969	-1.51361864
143558	chr8	126861645	126862145	501	*	4.220247503	4.919682072	2.809557576	1.697579632
36962	chr13	76197405	76197905	501	*	4.207208468	2.539507602	4.960167199	-1.845616712
42702	chr15	10691401	10691901	501	*	4.530753179	3.158283109	5.221188681	-1.646300171
60330	chr17	31309201	31309701	501	*	4.625708726	5.285178519	3.375879463	1.535185956
149676	chr9	111755176	111755676	501	*	4.199490023	4.907814781	2.749777077	1.696869391
44687	chr15	43735607	43736107	501	*	4.662682045	3.381612499	5.330355912	-1.595250366
8785	chr1	180284089	180284589	501	*	4.454575283	3.041533635	5.154554028	-1.682952643
150574	chrX	11528475	11528975	501	*	4.182208105	4.909373042	2.64527441	1.75685507
56479	chr16	79186416	79186916	501	*	3.670623361	4.496659732	1.532606728	2.092410501
67589	chr18	82517913	82518413	501	*	4.515771304	5.16884234	3.289727096	1.534024756
18406	chr11	6406321	6406821	501	*	4.714592081	3.440314847	5.380501228	-1.559921312
36077	chr13	56634185	56634685	501	*	4.420190153	2.946713178	5.133786244	-1.712068424
140171	chr8	83853261	83853761	501	*	4.61832109	5.286429651	3.33557223	1.558035383
149801	chr9	115988370	115988870	501	*	4.528423637	3.138128447	5.223092824	-1.640982576
69437	chr19	26704639	26705139	501	*	4.274271019	2.688144381	5.011469328	-1.771665398
135225	chr7	116191022	116191522	501	*	4.523855847	5.166897849	3.334114838	1.505429467
104638	chr5	16967601	16968101	501	*	3.791576449	1.100108026	4.675349099	-2.320982321
44809	chr15	50248316	50248816	501	*	3.99349941	2.052343803	4.792248581	-1.989731904
139101	chr8	66772600	66773100	501	*	3.451768933	4.325102243	0.87924166	2.225349335
44632	chr15	43044890	43045390	501	*	4.551762227	3.252342197	5.224151817	-1.599054688
23236	chr11	90851153	90851653	501	*	4.168766714	2.468730737	4.927685194	-1.875050641
137261	chr8	10143924	10144424	501	*	3.475280269	4.338412433	1.009476321	2.218314513
67178	chr18	74917659	74918159	501	*	4.334755318	2.829487225	5.055239799	-1.744211668
84463	chr2	179669048	179669548	501	*	3.857890802	4.626496135	2.103400678	1.886253434
92654	chr4	18461603	18462103	501	*	4.036908325	2.135730414	4.829576561	-1.942330401
91678	chr3	153292785	153293285	501	*	3.768458837	4.559358784	1.878670313	1.950276101
30448	chr12	79317090	79317590	501	*	4.193934404	2.55837876	4.940809152	-1.82541283
151916	chrX	98205733	98206233	501	*	3.890570979	4.660369788	2.129201375	1.861860852
96722	chr4	100307395	100307895	501	*	4.126684078	2.394368261	4.891396717	-1.859228681
74323	chr2	30912303	30912803	501	*	4.576885488	3.307395199	5.241544627	-1.569129482
62941	chr17	72965924	72966424	501	*	4.260375856	2.674419276	4.997540184	-1.798056688
69262	chr19	23351785	23352285	501	*	4.608348351	3.364526316	5.266214663	-1.547691638
142133	chr8	114928670	114929170	501	*	4.519835669	5.172622018	3.294838198	1.52252591
92559	chr4	14870941	14871441	501	*	4.125486995	2.419790227	4.885432668	-1.848719229
147656	chr9	71408852	71409352	501	*	4.2216913	2.591685246	4.96749927	-1.79491341
45930	chr15	62278489	62278989	501	*	4.009126444	1.991963735	4.818916599	-1.989873382
108529	chr5	90855824	90856324	501	*	4.627554429	3.35166486	5.293883428	-1.591850331
97501	chr4	107617504	107618004	501	*	4.63806021	3.402839971	5.293615698	-1.534617298
138353	chr8	34622616	34623116	501	*	3.673947535	4.471692049	1.739485414	1.960072806

100473	chr4	134136260	134136760	501	*	4.641402919	3.427960643	5.291028999	-1.524451428
107847	chr5	66901070	66901570	501	*	4.294977392	2.784931936	5.016481162	-1.718875704
64449	chr18	10840639	10841139	501	*	3.958855045	1.905638281	4.773654614	-2.03410174
90780	chr3	136911330	136911830	501	*	4.438296361	3.052343803	5.131940074	-1.654133327
66862	chr18	66258873	66259373	501	*	4.617357675	3.347072738	5.282224681	-1.561749135
76426	chr2	60445056	60445556	501	*	3.91915241	1.875857889	4.732587551	-2.013238372
140964	chr8	93938125	93938625	501	*	3.999838762	4.755795013	2.315981891	1.816576006
145613	chr9	49922626	49923126	501	*	4.268017391	2.719065439	4.997671744	-1.754174822
93550	chr4	43703842	43704342	501	*	4.13778061	2.444467998	4.895472881	-1.850311338
86108	chr3	51064117	51064617	501	*	3.868934051	1.735989292	4.694319151	-2.062260217
84336	chr2	173343619	173344119	501	*	4.174492325	2.535589411	4.922008145	-1.805224657
145460	chr9	45975615	45976115	501	*	4.235044652	2.635743221	4.974861009	-1.788554405
140798	chr8	91607010	91607510	501	*	3.990733854	4.734094421	2.373364248	1.795032294
92393	chr4	11975729	11976229	501	*	4.513938812	3.175644603	5.1960734	-1.60754977
104324	chr5	4747600	4748100	501	*	4.204525848	2.635476937	4.938287551	-1.764739773
131436	chr7	36328131	36328631	501	*	3.597393769	4.421544654	1.474005799	2.043376673
38773	chr13	115037265	115037765	501	*	4.110028273	2.415532526	4.867936815	-1.83533796
93151	chr4	37844364	37844864	501	*	3.811099715	0.302791982	4.746272156	-2.464506471
129921	chr6	149332878	149333378	501	*	3.913701662	1.911697231	4.721342704	-2.002049871
93078	chr4	34243696	34244196	501	*	4.060974244	2.2845705	4.833358115	-1.874149445
92544	chr4	14745499	14745999	501	*	4.071934839	2.293878747	4.844600905	-1.871134603
21897	chr11	75240479	75240979	501	*	4.230833178	2.61361206	4.974164834	-1.781139484
95103	chr4	63064817	63065317	501	*	4.186802181	2.516415785	4.940261937	-1.81824386
72010	chr2	5200839	5201339	501	*	4.312475829	2.823306016	5.029495273	-1.713373596
38165	chr13	104393881	104394381	501	*	3.962115918	1.98623333	4.765992347	-1.954836287
69052	chr19	17035268	17035768	501	*	4.42809688	3.033625144	5.123748728	-1.643163681
109156	chr5	102794531	102795031	501	*	4.701345567	3.494642908	5.349113457	-1.524135798
115489	chr6	12748576	12749076	501	*	3.899075698	4.656780737	2.205693274	1.810960997
137737	chr8	15729002	15729502	501	*	3.34387008	4.234676867	0.565806526	2.299635025
147098	chr9	64383371	64383871	501	*	4.211097591	2.649783676	4.943286593	-1.761226682
76780	chr2	69349025	69349525	501	*	4.106624608	2.32533244	4.879842249	-1.889119601
138944	chr8	60664231	60664731	501	*	3.849859769	4.607364241	2.157573607	1.82422571
34137	chr13	32183106	32183606	501	*	3.85056302	1.722265534	4.675349099	-2.046442904
95616	chr4	81981796	81982296	501	*	4.000631352	2.180488672	4.780359247	-1.896921362
142257	chr8	117093967	117094467	501	*	4.369959785	5.041483389	3.073931657	1.569405347
69518	chr19	28225578	28226078	501	*	4.676925161	3.466855783	5.325622679	-1.51124708
56643	chr16	86797052	86797552	501	*	4.442463518	3.110146905	5.123120906	-1.600632601
9381	chr1	185754418	185754918	501	*	4.203916975	4.903525474	2.792475899	1.6608752
12466	chr10	51416647	51417646	1000	*	4.215576129	4.902721579	2.856772192	1.610292131
60543	chr17	33526448	33526948	501	*	4.553176795	3.296027988	5.214588967	-1.544297832
86111	chr3	51082877	51083377	501	*	4.099516815	2.419790227	4.854710532	-1.819566248
78603	chr2	103461543	103462043	501	*	4.249082696	2.742555286	4.96983626	-1.725326187
24609	chr11	104316905	104317405	501	*	4.428669093	3.049798727	5.1206322	-1.639757975
130248	chr7	16324601	16325101	501	*	4.455792293	3.041533635	5.156052219	-1.652308381
133079	chr7	79907347	79907847	501	*	4.600872628	3.327305368	5.266596711	-1.542604782
139097	chr8	66681236	66681736	501	*	4.086810515	4.792279909	2.649772818	1.675557808
132335	chr7	64808778	64809278	501	*	3.414616368	4.277720329	0.949095793	2.155634965
64466	chr18	11387416	11387916	501	*	3.463194657	4.300568831	1.233507403	2.089065828
58482	chr17	16051673	16052173	501	*	4.599047433	3.360263249	5.255562475	-1.532646196
78900	chr2	110005109	110005609	501	*	3.897999035	1.899553778	4.705131714	-1.978833385
43095	chr15	25157675	25158175	501	*	4.471529369	3.148240973	5.149941149	-1.577865115

Table S2. ATAC-seq in D5 in mDPCs-CAS9 cells and sgCreb3l1_A8_4: 888 NFRs enriched and 510 NFRs lost at D5_Ctrl

144374	chr9	22945562	22946062	501	*	4.327801633	2.875632435	5.036676167	-1.672858492
114923	chr5	149381729	149382229	501	*	4.06252509	2.293878747	4.833579048	-1.85102295
37358	chr13	93996528	93997028	501	*	4.474979197	3.155732701	5.152379923	-1.579734412
58052	chr17	8552597	8553097	501	*	3.637408091	4.43407442	1.710089811	1.921270084
132240	chr7	60244117	60244617	501	*	3.391367994	4.328485678	0	2.421680971
6033	chr1	134765139	134765639	501	*	4.191317916	2.61361206	4.926827791	-1.745339956
151174	chrX	48904430	48904930	501	*	3.754311748	1.364653002	4.609641692	-2.146537134
94174	chr4	52481674	52482174	501	*	3.883378008	1.893888847	4.689225111	-1.9682197
85887	chr3	39431797	39432297	501	*	4.134016058	2.500423441	4.880514019	-1.775259357
107913	chr5	72441661	72442161	501	*	4.053385694	4.778651504	2.525539846	1.724090399
58060	chr17	8757538	8758038	501	*	3.513567162	4.33624354	1.401503636	2.030327734
150385	chrX	7282575	7283075	501	*	3.981518905	2.175461453	4.758910125	-1.873857056
69598	chr19	29014449	29014949	501	*	4.123228922	2.411262223	4.884306778	-1.824794036
89749	chr3	114082032	114082532	501	*	4.326897428	2.866530312	5.037598617	-1.672685206
61699	chr17	46918923	46919423	501	*	4.609540908	3.392327415	5.260201938	-1.521551878
19096	chr11	25937921	25938421	501	*	3.303748803	4.19512385	0.518438177	2.251227161
142310	chr8	117406446	117406946	501	*	3.587983417	4.392422873	1.608228357	1.944441459
149049	chr9	104073207	104073707	501	*	4.074590323	2.27554362	4.850808535	-1.853582535
29930	chr12	73303208	73303708	501	*	4.270614377	2.755405065	4.993215304	-1.687725246
12589	chr10	56968832	56969332	501	*	3.846836183	1.701940698	4.673751737	-2.01573675
38882	chr13	119768245	119768745	501	*	4.245861062	2.708916246	4.973028314	-1.718331654
26641	chr12	4672659	4673159	501	*	4.003297629	2.233606726	4.774531153	-1.872407228
55635	chr16	49332305	49332805	501	*	4.212607123	2.688144381	4.937162248	-1.701098982
54177	chr16	25693049	25693549	501	*	4.218955443	2.719065439	4.938287551	-1.698578845
37140	chr13	90738383	90738883	501	*	4.583383693	3.375368406	5.231514359	-1.506875573
77053	chr2	73048941	73049441	501	*	4.180857131	2.598940231	4.917212833	-1.737298184
140690	chr8	88649970	88650470	501	*	4.764417832	5.560232935	2.842710973	1.907003819
149246	chr9	107279766	107280266	501	*	4.073384719	2.381646072	4.830788953	-1.792494966
41800	chr14	99908180	99908680	501	*	4.263766322	2.820088295	4.970735422	-1.669296286
25508	chr11	116155453	116155953	501	*	3.923159307	2.025063331	4.71535064	-1.904409397
38101	chr13	103400145	103400645	501	*	3.989466743	2.20500646	4.763222975	-1.863485882
12448	chr10	45892393	45892893	501	*	3.516888924	4.352633819	1.300784237	2.029957749
31723	chr12	101789208	101789708	501	*	4.476200857	3.185316532	5.14640631	-1.56443708
94724	chr4	58182326	58182826	501	*	4.164792046	2.610161349	4.895613997	-1.725275937
92204	chr4	9336672	9337172	501	*	4.083681677	2.390034756	4.84143508	-1.793005092
53290	chr16	16060355	16060855	501	*	4.398743211	3.000516889	5.095275449	-1.616879645
28875	chr12	50999574	51000074	501	*	3.901995536	4.632742634	2.347729706	1.730092589
64754	chr18	22621815	22622315	501	*	3.739563704	4.521863366	1.903731233	1.850448206
29925	chr12	73259233	73259733	501	*	4.121037008	2.51214424	4.86274139	-1.751213016
150755	chrX	16275791	16276291	501	*	3.601761821	4.408190581	1.608228357	1.937335341
77037	chr2	72888158	72888658	501	*	4.373260045	2.958254779	5.073692325	-1.635137504
5204	chr1	121116815	121117315	501	*	4.569971887	3.336015077	5.225186486	-1.503974324
43638	chr15	31588954	31589454	501	*	4.529129582	3.245162389	5.197553112	-1.549558248
44659	chr15	43460516	43461016	501	*	3.974999599	2.150804648	4.755394837	-1.86483518
85174	chr3	19444872	19445372	501	*	4.209124817	2.701940698	4.930018589	-1.688973821
19062	chr11	23118788	23119288	501	*	3.912523941	2.008608456	4.705614809	-1.89891216
63967	chr17	87375870	87376370	501	*	4.579468489	3.340677015	5.235985492	-1.510515339
78382	chr2	101600599	101601099	501	*	3.978377731	2.195466071	4.751870865	-1.854461675
152782	chrX	145515703	145516203	501	*	3.923006566	2.079117978	4.706614146	-1.891238902
61958	chr17	52832131	52832631	501	*	3.72471575	4.501355111	1.92315653	1.873674219
128861	chr6	134609008	134609508	501	*	4.165985469	2.639133355	4.891042737	-1.710348001

75997	chr2	51001515	51002015	501	*	4.476264111	3.178160083	5.148318016	-1.555102295
27912	chr12	25978311	25978811	501	*	3.504746328	4.319582039	1.451265682	1.953704497
61260	chr17	44378404	44378904	501	*	4.212532306	2.631811228	4.948648117	-1.737764777
123465	chr6	88384597	88385097	501	*	4.494690134	3.24293596	5.154671948	-1.527992096
34743	chr13	42524798	42525298	501	*	4.165107177	2.642780529	4.889212535	-1.708925658
41649	chr14	77617275	77617775	501	*	3.937410612	4.6637817	2.404284125	1.700686854
20949	chr11	62034409	62034909	501	*	4.367624766	3.033423046	5.048748831	-1.578943843
13568	chr10	68353601	68354101	501	*	4.384960672	3.030641853	5.071017891	-1.586488225
12454	chr10	48322480	48322980	501	*	3.307210679	4.179324848	0.747902047	2.139104907
53934	chr16	22877636	22878136	501	*	4.248210483	2.813866597	4.953069068	-1.651501564
72865	chr2	24752439	24752939	501	*	4.000306097	2.289232131	4.761223103	-1.800199162
65705	chr18	43967757	43968257	501	*	3.643073806	0.667590058	4.548315076	-2.240175883
69066	chr19	17271019	17271519	501	*	4.465477998	3.173308175	5.136013426	-1.54724372
95559	chr4	81082504	81083004	501	*	4.211563741	2.722265534	4.928611006	-1.685464384
53822	chr16	21440970	21441470	501	*	4.545465521	3.282063919	5.208527148	-1.523187892
28878	chr12	51185752	51186252	501	*	3.271797605	4.160643977	0.518438177	2.195667532
39523	chr14	24530262	24530762	501	*	4.340354426	2.958041841	5.033135648	-1.603273019
144962	chr9	40717576	40718076	501	*	4.245134159	2.791279399	4.954385307	-1.650925893
64597	chr18	13281745	13282245	501	*	3.692418471	4.482630439	1.80703323	1.860339425
43113	chr15	25358540	25359040	501	*	4.380722177	3.044093321	5.062445985	-1.58388039
142494	chr8	120180612	120181112	501	*	3.692300256	4.482587723	1.806432553	1.90325142
147909	chr9	75679521	75680021	501	*	3.75525162	1.480476991	4.597912836	-2.0313551
5233	chr1	121653826	121654326	501	*	3.507259459	4.316160068	1.496393036	1.93890563
42738	chr15	10916233	10916733	501	*	3.942834998	2.180488672	4.712802711	-1.826610431
79753	chr2	120986434	120986934	501	*	3.652039502	1.068704555	4.526360569	-2.144935088
24973	chr11	108963816	108964316	501	*	3.573495031	4.377479313	1.596871554	1.906379778
18805	chr11	19204019	19204519	501	*	4.044575737	4.749888738	2.608228357	1.628123505
41644	chr14	77375570	77376070	501	*	4.276209626	4.964708624	2.91180279	1.582987085
137387	chr8	11372010	11372510	501	*	4.224808522	4.888522707	2.958923711	1.537763312
139747	chr8	72905424	72905924	501	*	3.386677549	4.229923472	1.106818607	2.019441808
137507	chr8	12894840	12895340	501	*	4.030213236	4.764627391	2.457940678	1.686458319
75477	chr2	37370586	37371086	501	*	4.198601713	2.732570572	4.910557565	-1.666411271
86619	chr3	58137008	58137508	501	*	3.828213455	1.844989234	4.633155833	-1.907524004
81556	chr2	148233896	148234396	501	*	4.097343825	2.539222947	4.828880643	-1.718376747
70949	chr19	47031940	47032440	501	*	4.518501356	3.279893759	5.174968926	-1.500641936
85788	chr3	38128332	38128832	501	*	4.292368121	2.869419783	4.994627274	-1.626284418
33824	chr13	26126494	26126994	501	*	3.640823546	1.078726303	4.513195922	-2.129371061
90145	chr3	122381531	122382031	501	*	3.736023296	4.487651099	2.07542762	1.764749713
129376	chr6	143535136	143535636	501	*	3.923639224	4.660318186	2.340109033	1.696183437
132637	chr7	68337233	68337733	501	*	4.317004638	2.960966426	5.003479534	-1.584148606
140016	chr8	80314758	80315258	501	*	3.713263574	4.470741018	2.021125063	1.793600784
36982	chr13	77927837	77928337	501	*	3.776079074	1.701940698	4.593720898	-1.948271994
107836	chr5	66780961	66781461	501	*	4.410993033	3.128093796	5.079140488	-1.543536272
56485	chr16	84378346	84378846	501	*	3.446628618	4.280131284	1.248985095	1.970038138
45191	chr15	55358707	55359207	501	*	3.62201621	0.694931258	4.523912232	-2.197794409
35612	chr13	52647955	52648455	501	*	3.998054288	2.32533244	4.751948857	-1.779021337
67427	chr18	78298832	78299332	501	*	4.158701589	2.635476937	4.882996124	-1.690372015
80713	chr2	131706465	131706965	501	*	4.254148274	2.832684074	4.95606702	-1.632795983
115494	chr6	13030909	13031409	501	*	3.63350323	4.417601891	1.786576282	1.863927133
126942	chr6	115356481	115356981	501	*	3.708163706	1.51214424	4.541467575	-1.991735642
139209	chr8	69328568	69329068	501	*	4.068214603	4.758866558	2.694839786	1.592036225

96505	chr4	97664684	97665184	501	*	3.686217544	4.468538126	1.850256623	1.836751727
53988	chr16	23513412	23513912	501	*	4.26953383	4.929895	3.016351388	1.504106664
19543	chr11	40552069	40552569	501	*	3.715134439	4.516240586	1.758134056	1.890143893
3339	chr1	81978613	81979113	501	*	4.213794621	2.71560633	4.932761012	-1.668891784
84099	chr2	168725953	168726453	501	*	4.379241088	3.057818028	5.057186707	-1.569718566
1791	chr1	57040026	57040526	501	*	4.200996881	2.708663132	4.918701054	-1.6597076
93058	chr4	33747454	33747954	501	*	3.940033128	2.150804648	4.714597366	-1.81301367
16980	chr10	113538206	113538706	501	*	3.545501215	4.34057807	1.628640729	1.873652961
63880	chr17	86593078	86593578	501	*	4.10418731	2.550633958	4.834788175	-1.712151438
143451	chr8	125624197	125624697	501	*	3.619612168	4.406046116	1.758134056	1.848705474
30093	chr12	74951023	74951523	501	*	4.084161325	2.539222947	4.812987103	-1.697563995
108010	chr5	73755622	73756122	501	*	4.291948643	2.926516563	4.980694614	-1.598120346
76961	chr2	71958300	71958800	501	*	3.783458645	4.5296921	2.151242183	1.727191204
96844	chr4	101332982	101333482	501	*	3.859492303	1.997671468	4.645980925	-1.832716029
31358	chr12	90740439	90740939	501	*	3.648584315	4.432105223	1.805231202	1.813020414
143735	chr9	6373243	6373743	501	*	4.09364754	2.524058304	4.827518725	-1.706366753
139783	chr8	74639239	74639739	501	*	3.202226756	4.106189566	0.24544515	2.18202119
94098	chr4	47780991	47781491	501	*	4.132895234	4.812248073	2.805832003	1.548419282
76767	chr2	69086807	69087307	501	*	4.28811123	2.899775525	4.982318174	-1.596790619
12778	chr10	59543281	59543781	501	*	4.181277566	2.749091532	4.885645858	-1.634295476
58086	chr17	10745051	10745551	501	*	3.37772293	4.218085585	1.122743923	1.979909604
76861	chr2	70904504	70905004	501	*	4.436965368	3.202537395	5.092307136	-1.502771543
77703	chr2	90670301	90670801	501	*	4.168444128	2.712138908	4.878241834	-1.63055903
82270	chr2	155735116	155735616	501	*	3.602938299	0.76256364	4.498532046	-2.149682158
18453	chr11	6773368	6773868	501	*	3.983571085	2.329535227	4.733963185	-1.75377044
142056	chr8	113523484	113523984	501	*	3.241715828	4.10760588	0.747902047	2.051044767
29994	chr12	73536986	73537486	501	*	3.672810809	1.488606638	4.504660807	-1.950527284
108466	chr5	90077007	90077507	501	*	3.989510938	4.695859651	2.548584379	1.613683629
135259	chr7	117033982	117034482	501	*	3.246961808	4.111367871	0.768294007	2.049848453
6562	chr1	143914275	143914775	501	*	3.164611796	4.069686309	0.191582213	2.153414544
17188	chr10	118781861	118782361	501	*	3.438289799	4.255918745	1.364246997	1.920162732
147496	chr9	68943960	68944460	501	*	3.518613394	4.309542059	1.628640729	1.846566649
85757	chr3	37769143	37769643	501	*	3.86055517	2.019871251	4.643643486	-1.814404138
15584	chr10	84256542	84257042	501	*	4.381572619	3.107509886	5.047425866	-1.513583418
31944	chr12	104450251	104450751	501	*	3.949309154	2.298174031	4.699152696	-1.733094419
35484	chr13	51408581	51409081	501	*	4.366654975	3.00619095	5.05420215	-1.557303901
24444	chr11	102716071	102716571	501	*	3.976382081	2.316557803	4.727864918	-1.742393106
142456	chr8	120028229	120028729	501	*	3.419708819	4.2381406	1.339693943	1.917883918
18588	chr11	9216250	9216750	501	*	4.059301502	2.51214424	4.788585698	-1.676919355
71128	chr19	53107236	53107736	501	*	3.949869377	2.307395199	4.698067219	-1.729223678
80921	chr2	135499643	135500143	501	*	4.036532915	2.444467998	4.774914806	-1.708285189
64456	chr18	11015751	11016251	501	*	3.934524944	4.654124479	2.433389033	1.626372729
90202	chr3	122921650	122922150	501	*	4.098397928	2.610161349	4.815215054	-1.637009625
26157	chr11	119945219	119945719	501	*	4.156284786	2.735740883	4.857992234	-1.610429923
77040	chr2	72967042	72967542	501	*	4.154330483	2.719065439	4.859398094	-1.611472919
34914	chr13	44580159	44580659	501	*	4.142928867	2.698440235	4.850080462	-1.610525957
116332	chr6	29812559	29813059	501	*	3.869374937	2.073724131	4.645022565	-1.804606508
16509	chr10	97687115	97687615	501	*	3.779627472	1.857142036	4.575560909	-1.842113953
62634	chr17	67257757	67258257	501	*	3.733905525	0	4.688050615	-2.166796187
142064	chr8	113721286	113721786	501	*	4.146115254	4.810463292	2.877813227	1.507812858
69839	chr19	32740985	32741485	501	*	3.846002461	2.062875559	4.619532182	-1.792910412

137664	chr8	14331220	14331720	501	*	3.94137531	4.655864726	2.463823288	1.618033038
86328	chr3	53221497	53221997	501	*	4.080131563	2.55837876	4.804115957	-1.670987892
115745	chr6	22010705	22011205	501	*	4.283651082	2.966586191	4.960504621	-1.537926005
6430	chr1	137635753	137636253	501	*	3.719207117	1.729143731	4.52513692	-1.873109592
86395	chr3	54578168	54579130	963	*	3.853695954	2.089456776	4.623990617	-1.786786182
95418	chr4	65473051	65473551	501	*	4.060009527	2.520097871	4.787793643	-1.664952824
45998	chr15	63607938	63608438	501	*	4.27446429	2.966797871	4.948948638	-1.526886119
5347	chr1	127625900	127626400	501	*	3.8379493	4.578955163	2.232613972	1.689160472
126261	chr6	108237664	108238164	501	*	3.874297778	2.160643977	4.635679289	-1.743909669
83758	chr2	167546840	167547340	501	*	4.148690123	2.694931258	4.857919859	-1.609698239
76606	chr2	65126406	65126906	501	*	4.18540553	2.80436501	4.8778847	-1.571700454
6566	chr1	143946787	143947287	501	*	3.307023819	4.168236366	0.860369038	1.975625252
65664	chr18	43360037	43360537	501	*	4.099594267	2.591959736	4.820584158	-1.634312291
81424	chr2	145527110	145527610	501	*	3.956635959	2.320786192	4.703567108	-1.717691092
146860	chr9	62725237	62725737	501	*	4.091384426	2.6175939	4.805049362	-1.622183807
65388	chr18	37948779	37949279	501	*	4.099229084	2.635476937	4.810680829	-1.619490914
44881	chr15	51197764	51198264	501	*	3.539401742	0.749091532	4.431167027	-2.076002092
81567	chr2	148346017	148346517	501	*	3.514199783	0.722766969	4.406052492	-2.071811116
86974	chr3	66180798	66181298	501	*	4.323535863	3.055083512	4.987923275	-1.501493923
7330	chr1	159093055	159093555	501	*	3.52416879	0.653196641	4.422032967	-2.072053995
71328	chr19	55599077	55599577	501	*	3.647524506	1.488606638	4.476217689	-1.908711154
33015	chr13	4660179	4660679	501	*	4.317059624	3.025266603	4.987498369	-1.506265749
138042	chr8	25781267	25781767	501	*	4.058245658	4.75476887	2.660057888	1.559887285
95721	chr4	83154267	83154767	501	*	4.006584537	2.411262223	4.745613197	-1.687918596
72665	chr2	18853232	18853732	501	*	5.169298881	3.660355951	5.890567781	-1.646936494
95352	chr4	64535843	64536343	501	*	3.650522361	1.55091637	4.471561507	-1.896506042
76576	chr2	62602675	62603175	501	*	4.331957187	3.002943913	5.011794964	-1.522318747
143121	chr8	122923470	122923970	501	*	3.485366188	4.274283007	1.608228357	1.806263722
19818	chr11	46617146	46617646	501	*	3.472208214	4.255763825	1.628640729	1.79622583
16629	chr10	102553870	102554370	501	*	4.187807169	2.800867273	4.881684369	-1.569433437
69497	chr19	27581707	27582207	501	*	3.644972251	1.496690731	4.472318841	-1.89936723
82833	chr2	160353568	160354068	501	*	3.869211711	4.602933916	2.300357676	1.673329844
70736	chr19	45591638	45592138	501	*	4.327155007	3.030844341	4.998750843	-1.511033987
70315	chr19	40650711	40651211	501	*	3.811707473	1.997671468	4.590425564	-1.770572904
28807	chr12	45067409	45067909	501	*	3.303203207	4.136910039	1.103889625	1.901098999
40401	chr14	48224145	48224645	501	*	3.922810195	2.316225571	4.664061597	-1.691405539
109132	chr5	102566624	102567124	501	*	3.988327327	2.390034756	4.727944219	-1.674325117
61952	chr17	52569561	52570061	501	*	3.532753778	4.324049462	1.640425358	1.777013389
8151	chr1	170067996	170068496	501	*	4.038393356	2.496397501	4.766609902	-1.642794805
11675	chr10	34904062	34904562	501	*	3.634475081	4.398610334	1.905414403	1.720120715
76566	chr2	62505258	62505758	501	*	3.685457052	1.729143731	4.486461611	-1.835388078
13371	chr10	66187034	66187534	501	*	3.237174097	4.093286829	0.840072824	1.973504309
98131	chr4	117007043	117007543	501	*	4.076514032	2.613882411	4.787717564	-1.603043976
55540	chr16	45765982	45766482	501	*	3.854653595	2.185498435	4.607883783	-1.713026904
137898	chr8	24227501	24228001	501	*	3.174274533	4.033423046	0.747902047	1.963640211
26739	chr12	5354702	5355202	501	*	3.861984742	2.165723004	4.620215708	-1.72055601
92454	chr4	13484566	13485066	501	*	3.661492586	1.588123434	4.479030736	-1.87946265
38095	chr13	103315294	103315794	501	*	4.318897845	3.005778964	4.994759016	-1.50394649
39809	chr14	28532849	28533349	501	*	4.017636347	2.492654347	4.742300683	-1.626714183
86534	chr3	56122868	56123368	501	*	3.503737937	1.046848728	4.365979925	-1.970988645
78317	chr2	94919347	94919847	501	*	3.560253747	1.311983775	4.399829359	-1.919898783

Table S2. ATAC-seq in D5 in mDPCs-CAS9 cells and sgCreb3l1_A8_4: 888 NFRs enriched and 510 NFRs lost at D5_Ctrl

75829	chr2	46107016	46107516	501	*	4.290933102	2.97260584	4.968103393	-1.524149948
144540	chr9	27770902	27771402	501	*	3.47302414	4.268632196	1.552678373	1.788131492
92634	chr4	17189429	17189929	501	*	3.704596026	1.768767276	4.5025461	-1.830977876
32966	chr13	3675842	3676342	501	*	4.047268992	2.520097871	4.772393224	-1.639460179
109670	chr5	108667051	108667551	501	*	3.611577387	1.528007894	4.430485234	-1.863861328
139852	chr8	77330218	77330718	501	*	3.913122368	2.256626228	4.663978705	-1.681432704
140461	chr8	86275650	86276150	501	*	3.674013446	4.409122322	2.09829886	1.663783081
108347	chr5	81474400	81474900	501	*	3.953354521	2.411262223	4.681591059	-1.640361631
75739	chr2	44334486	44334986	501	*	3.52422706	1.046848728	4.388505989	-1.967764467
28591	chr12	35853114	35853614	501	*	3.614709405	4.375305534	1.905414403	1.699018447
86388	chr3	54530239	54530739	501	*	3.868669402	2.224254214	4.617237086	-1.703456151
56651	chr16	86897903	86898403	501	*	3.726615978	1.86964621	4.512329311	-1.775738176
109573	chr5	107541599	107542099	501	*	3.998241155	2.419480941	4.733963086	-1.653860512
70046	chr19	36669561	36670061	501	*	4.211772422	2.896724093	4.888119247	-1.519337635
33720	chr13	24268293	24268793	501	*	3.747185666	1.929288469	4.526542825	-1.773926277
13587	chr10	68589480	68589980	501	*	4.068931499	2.624720077	4.776020699	-1.586907063
46174	chr15	66500658	66501158	501	*	3.992707643	2.394682978	4.732271537	-1.661776494
134584	chr7	105022477	105022977	501	*	3.604004674	4.350422251	1.97083044	1.678260821
129375	chr6	143530754	143531254	501	*	3.242052627	4.081437083	0.995406894	1.902662197
47186	chr15	75818514	75819014	501	*	3.986641636	4.667315816	2.654257245	1.531997715
120850	chr6	67170158	67170658	501	*	3.661252074	4.398688789	2.073931657	1.657444875
90739	chr3	135966265	135966765	501	*	3.983782496	2.411262223	4.718246715	-1.644051542
41963	chr14	117267821	117268321	501	*	4.070914058	2.635476937	4.776020699	-1.58378044
141212	chr8	95672703	95673203	501	*	3.502947124	4.2845705	1.671259803	1.740925986
46071	chr15	64240544	64241044	501	*	3.953382415	2.415532526	4.680738012	-1.632475935
10055	chr10	3807884	3808384	501	*	3.341112448	4.148314562	1.342181649	1.819179799
95962	chr4	86266422	86266922	501	*	3.670252712	1.645945722	4.48104687	-1.811965635
142473	chr8	120096462	120096962	501	*	3.972947981	4.671641736	2.565452002	1.537091879
127132	chr6	116673592	116674092	501	*	4.209637495	2.835459261	4.900481519	-1.521571029
41602	chr14	76618616	76619116	501	*	3.515019062	1.025469847	4.380493005	-1.944598362
125206	chr6	94843379	94843879	501	*	3.766426617	4.483721599	2.275984632	1.605819597
116380	chr6	30112718	30113218	501	*	4.149679157	2.81062773	4.832000327	-1.521688746
56582	chr16	85938279	85938779	501	*	3.53135838	1.256626228	4.374014702	-1.889284845
75705	chr2	39157742	39158242	501	*	3.794848163	2.041332642	4.563283878	-1.72458662
118267	chr6	41826646	41827146	501	*	3.273600413	4.095970412	1.163877366	1.849936383
92531	chr4	14558126	14558626	501	*	3.832311267	2.185498435	4.581334984	-1.683970125
63475	chr17	81480294	81480794	501	*	4.152295219	2.778556423	4.843034205	-1.529368819
41792	chr14	99641963	99642463	501	*	3.455791514	0.749091532	4.340834761	-1.98410722
54025	chr16	24056340	24056840	501	*	3.903541177	2.302791982	4.643643486	-1.65479186
135888	chr7	129642672	129643172	501	*	3.897447012	4.589181292	2.519537615	1.549597623
149671	chr9	111587442	111587942	501	*	3.582918879	4.367818649	1.73101939	1.748520348
144542	chr9	27813340	27813840	501	*	3.335101228	4.134321532	1.390794377	1.805640001
126807	chr6	114478635	114479135	501	*	4.093948144	2.688144381	4.792248581	-1.545587165
46379	chr15	67981998	67982498	501	*	3.469047466	0.969326919	4.335511639	-1.928357832
85368	chr3	27604595	27605095	501	*	3.831850965	2.175461453	4.582687202	-1.678733877
10746	chr10	17213183	17213683	501	*	3.658974084	1.638658176	4.469208064	-1.799820339
102481	chr4	144879238	144879738	501	*	3.586835135	1.543131295	4.400326773	-1.828085966
79110	chr2	114812717	114813217	501	*	3.463021845	1.014250844	4.324447914	-1.923118141
57354	chr16	95415889	95416389	501	*	3.775352045	4.487651099	2.307766824	1.576208042
38036	chr13	102591233	102591733	501	*	3.785585265	2.0310468	4.554199008	-1.717655166
52613	chr16	4730804	4731304	501	*	4.084806415	2.621295865	4.796204663	-1.582358687

152320	chrX	106656379	106656879	501	*	4.112230013	2.715354387	4.80844586	-1.544288775
86517	chr3	55779977	55780477	501	*	3.461899988	0.801291926	4.343053288	-1.966412716
69059	chr19	17223730	17224230	501	*	3.695833676	1.86340767	4.477577735	-1.738815675
22071	chr11	76395271	76395771	501	*	3.760984911	2.002943913	4.530207096	-1.705769114
54134	chr16	24978197	24978697	501	*	3.989142821	2.496397501	4.706935931	-1.588880509
32863	chr12	116208215	116208715	501	*	3.994214849	2.51980936	4.708014758	-1.586586204
81926	chr2	152898950	152899450	501	*	3.515264039	1.266115931	4.354942899	-1.862206712
93979	chr4	46868136	46868636	501	*	3.793864149	4.498616018	2.359990275	1.568217459
86912	chr3	65587446	65587946	501	*	3.874985392	2.302791982	4.609383549	-1.636293228
76336	chr2	59554275	59554775	501	*	4.137974921	2.784931936	4.823721814	-1.511857691
11505	chr10	29177950	29178450	501	*	3.670011298	4.413460189	2.052187951	1.639571464
151368	chrX	53236571	53237556	986	*	4.134241332	2.78162738	4.819883793	-1.50711615
91986	chr4	5744824	5745324	501	*	3.507490054	1.311983775	4.340731052	-1.855785221
79312	chr2	117796034	117796534	501	*	3.948472324	2.368490283	4.68443976	-1.616099777
17561	chr10	126520961	126521461	501	*	3.145325568	3.989287747	0.859211062	1.88750988
143972	chr9	14599699	14600199	501	*	4.086518776	2.698440235	4.780664998	-1.53041293
120479	chr6	59431701	59432201	501	*	3.932569073	2.411262223	4.656459422	-1.599247525
57082	chr16	92890667	92891167	501	*	3.875741085	2.334054032	4.603893609	-1.623269335
91085	chr3	143713199	143713699	501	*	3.56319719	1.504146517	4.378794025	-1.803603038
152877	chrX	153359278	153359778	501	*	3.902391964	4.596380444	2.514981205	1.512167946
35006	chr13	45622493	45622993	501	*	3.564704204	0	4.516947524	-1.920902751
88788	chr3	99112925	99113425	501	*	3.834207714	2.209574229	4.57897826	-1.656884436
45068	chr15	54291020	54291520	501	*	3.925333507	2.411262223	4.647693048	-1.593710614
81075	chr2	137801226	137801726	501	*	3.626997071	1.742308005	4.417100044	-1.751271807
89273	chr3	104856732	104857232	501	*	3.48504943	1.302791982	4.316658598	-1.831224574
65043	chr18	33413210	33413710	501	*	3.682509921	1.911697231	4.453936117	-1.70997531
143385	chr8	125126476	125126976	501	*	3.476783273	1.025469847	4.338465412	-1.883998639
10762	chr10	17512538	17513038	501	*	3.615716032	1.66706907	4.415583279	-1.753536814
80850	chr2	133521030	133521530	501	*	3.385276736	0.694931258	4.268955135	-1.916419794
16951	chr10	112051892	112052392	501	*	3.76376629	4.464721891	2.346491163	1.543241731
95913	chr4	85202516	85203016	501	*	3.831737144	4.536513731	2.397754418	1.544845406
3812	chr1	88478663	88479163	501	*	3.667409633	4.389169576	2.156160484	1.582100908
90128	chr3	122210074	122210574	501	*	3.545567815	1.520097871	4.356524869	-1.785297874
8461	chr1	174632204	174632704	501	*	3.044964649	3.920433485	0.448959284	1.895954537
152912	chrX	157365475	157365975	501	*	3.327191368	0	4.294686386	-1.793997681
100223	chr4	133070416	133070916	501	*	3.906050262	2.377274125	4.631511144	-1.583422763
38828	chr13	118459078	118459578	501	*	3.689442951	1.929288469	4.459031441	-1.703652611
6592	chr1	145957657	145958157	501	*	3.624089204	4.364526316	2.0216427	1.60829741
9971	chr1	194345552	194346052	501	*	3.377637719	4.1656308	1.506349028	1.732553102
122396	chr6	84725904	84726404	501	*	3.855187983	2.316225571	4.582774898	-1.603713337
94668	chr4	57775510	57776010	501	*	3.474443038	1.24777069	4.311457045	-1.818136473
92203	chr4	9323881	9324381	501	*	3.488232512	1.27554362	4.323564958	-1.818926832
90232	chr3	123185010	123185510	501	*	3.750033055	2.09479223	4.500652641	-1.663377624
124652	chr6	91886316	91886816	501	*	3.531958078	1.51214424	4.342121465	-1.771314817
4905	chr1	114039321	114039821	501	*	3.12654826	3.976736328	0.784748503	1.84998902
13880	chr10	73515691	73516191	501	*	3.452098772	4.221659273	1.692105912	1.698446915
63131	chr17	75335774	75336274	501	*	4.106882763	2.752471371	4.792962487	-1.517859214
63340	chr17	79977041	79977541	501	*	3.596368646	1.722265534	4.384806742	-1.728010081
16604	chr10	99787422	99787922	501	*	3.476967314	1.293541251	4.308721096	-1.810943352
122418	chr6	84959249	84959749	501	*	3.457018873	4.215727885	1.758134056	1.657151608
118270	chr6	41914805	41915305	501	*	3.345826745	4.155991571	1.326002668	1.760431723

Table S2. ATAC-seq in D5 in mDPCs-CAS9 cells and sgCreb3l1_A8_4: 888 NFRs enriched and 510 NFRs lost at D5_Ctrl

77956	chr2	92426348	92426848	501	*	3.596494318	1.66706907	4.39347928	-1.727891076
29519	chr12	69434259	69434759	501	*	3.672162361	1.887754483	4.445909694	-1.691652626
132347	chr7	65082243	65082743	501	*	3.243558684	4.052343803	1.233507403	1.771733318
90436	chr3	129509652	129510152	501	*	3.461791608	4.222878079	1.749777077	1.657707539
107115	chr5	47807999	47808499	501	*	3.840630299	2.2845705	4.571745214	-1.595539777
9936	chr1	193476947	193477447	501	*	3.839699338	2.266115931	4.574378303	-1.597198074
76102	chr2	52321793	52322293	501	*	3.779623764	2.1305445	4.529077728	-1.625742697
3340	chr1	82004933	82005433	501	*	3.931627176	2.436085049	4.650023835	-1.571735143
86420	chr3	54832474	54832974	501	*	4.017781119	2.646154185	4.708014758	-1.516174336
7998	chr1	168002504	168003004	501	*	3.716915854	2.014250844	4.476311984	-1.650348622
125491	chr6	98990864	98991364	501	*	4.002371791	2.591685246	4.701805576	-1.516009592
134580	chr7	104533067	104533567	501	*	3.501989814	4.253535365	1.84183188	1.656971389
80308	chr2	128293648	128294148	501	*	3.897438429	2.419790227	4.611948893	-1.561275121
86636	chr3	58233326	58233826	501	*	3.654448624	1.875406946	4.427282832	-1.681200656
69539	chr19	28416036	28416536	501	*	3.587053385	1.722265534	4.374014702	-1.713775905
138192	chr8	28299493	28299993	501	*	2.879797525	3.851054184	0	1.71189872
69401	chr19	25805330	25805830	501	*	3.600173196	4.325102243	2.073931657	1.575572785
15942	chr10	88989941	88990441	501	*	3.294443183	4.085462808	1.403887097	1.726341242
77195	chr2	75400453	75400953	501	*	3.592336529	4.318442283	2.060479851	1.575169111
135256	chr7	116701411	116701911	501	*	3.089952243	3.937919162	0.768294007	1.819479273
115224	chr6	5448334	5448834	501	*	3.742227447	2.041332642	4.501302075	-1.651486145
114834	chr5	148662358	148662858	501	*	3.586827131	1.729143731	4.372654704	-1.706172289
131397	chr7	35365381	35365881	501	*	3.957985479	2.520097871	4.663647298	-1.536139133
35303	chr13	48995596	48996096	501	*	3.448995616	1.238159142	4.284103929	-1.783742616
79711	chr2	120366442	120366942	501	*	3.747860255	1.997671468	4.515715977	-1.656866838
65927	chr18	52832336	52832836	501	*	3.334601638	0.694931258	4.213942846	-1.855186717
137287	chr8	10695162	10695662	501	*	3.591864605	4.329717229	2.002458759	1.605361436
132341	chr7	64906921	64907421	501	*	3.71085941	4.421699084	2.249868448	1.542564408
142360	chr8	119496757	119497257	501	*	3.738445076	4.441270035	2.313025722	1.538400017
12326	chr10	43634288	43634788	501	*	3.531234605	1.527433971	4.339131658	-1.738145364
146541	chr9	60373117	60373617	501	*	3.807960206	2.165723004	4.556112854	-1.621035734
129377	chr6	143559256	143559756	501	*	3.49555411	4.24406807	1.851421074	1.645445531
80100	chr2	126083087	126083587	501	*	3.787374701	2.20500646	4.523820883	-1.602814845
150789	chrX	17505864	17506364	501	*	3.847086837	2.275201807	4.581422652	-1.579000682
145536	chr9	48354743	48355243	501	*	3.665740749	4.380537528	2.186783397	1.552027408
36082	chr13	56652425	56652925	501	*	3.67264502	1.844989234	4.453608476	-1.676946029
25146	chr11	112351988	112352488	501	*	3.594669639	1.742308005	4.379643701	-1.695906666
97089	chr4	105895346	105895846	501	*	3.956499694	2.53166055	4.659191905	-1.519376189
138234	chr8	31862749	31863249	501	*	3.602438011	4.322995913	2.096826438	1.562462601
302	chr1	16257466	16257966	501	*	3.634443901	1.851307755	4.407975193	-1.658864133
66196	chr18	57141210	57141710	501	*	3.695431896	1.997671468	4.453936117	-1.625454491
41466	chr14	74220179	74220679	501	*	3.974645603	2.566082208	4.673587162	-1.522074592
92816	chr4	29174586	29175086	501	*	3.579033345	1.722265534	4.364714335	-1.694153925
34288	chr13	34240008	34240508	501	*	3.654244448	1.923303102	4.418713475	-1.65563734
37586	chr13	96947344	96947844	501	*	3.89748029	2.456650372	4.603807186	-1.536436659
23229	chr11	90653913	90654413	501	*	3.88095675	2.411262223	4.593720898	-1.536137464
133698	chr7	89913410	89913910	501	*	3.655334	4.363438533	2.206603528	1.540637352
151892	chrX	96100305	96100805	501	*	3.957316168	2.54341518	4.657493487	-1.518076382
19473	chr11	35295286	35295786	501	*	3.699188468	2.036198888	4.451265561	-1.614998608
11679	chr10	35411055	35411555	501	*	3.536084414	4.260432976	2.012603179	1.569720844
128691	chr6	129212517	129213017	501	*	3.423902073	1.256626228	4.253645585	-1.754617586

21313	chr11	68666042	68666542	501	*	3.835784613	2.307395199	4.561164423	-1.560277523
67123	chr18	73541901	73542401	501	*	3.238361919	4.030641853	1.339693943	1.693536517
91996	chr4	5989937	5990437	501	*	3.953028403	2.569639845	4.646064857	-1.509126738
38224	chr13	106941512	106942012	501	*	3.500257999	1.472301272	4.31156288	-1.72058155
136284	chr7	134805694	134806194	501	*	3.745999873	2.165723004	4.482026512	-1.580623241
132486	chr7	66702751	66703251	501	*	3.536010776	1.456412644	4.354386655	-1.728551845
39721	chr14	27048477	27048977	501	*	3.693809428	2.057818028	4.440767732	-1.608417574
38795	chr13	116949484	116949984	501	*	3.828412735	2.298510445	4.554109557	-1.55734528
75850	chr2	48416870	48417370	501	*	3.65651879	1.875406946	4.429705734	-1.654942462
81008	chr2	137019969	137020469	501	*	3.421956095	1.238159142	4.253755747	-1.750881488
70356	chr19	41417982	41418482	501	*	3.753825019	2.145672734	4.495384173	-1.584919483
81405	chr2	145312167	145312667	501	*	3.683404947	2.008608456	4.437685074	-1.611517043
33832	chr13	26323982	26324482	501	*	3.361115357	1.014250844	4.211856556	-1.785307526
96055	chr4	87141954	87142454	501	*	3.931345587	2.539222947	4.626445171	-1.502300948
85095	chr3	13640705	13641205	501	*	2.993849478	3.93802713	0	1.765579562
153186	chrX	167103694	167104194	501	*	3.759858093	2.180488672	4.495702464	-1.579715977
5348	chr1	127627188	127627688	501	*	3.364708563	4.128113807	1.639751131	1.636890966
55110	chr16	35905224	35905724	501	*	3.467671001	4.207311094	1.869261125	1.584216016
138193	chr8	28487820	28488320	501	*	3.002184972	3.946690485	0	1.761448403
149661	chr9	111429316	111429816	501	*	3.955609929	2.508441772	4.663363883	-1.512991487
8421	chr1	172552967	172553467	501	*	3.624481246	4.331813775	2.179188391	1.518039712
94100	chr4	47845229	47845729	501	*	3.305497671	4.094445756	1.428161381	1.667130162
63476	chr17	81497851	81498351	501	*	3.858908769	2.377274125	4.574290095	-1.522021677
82908	chr2	160994181	160994681	501	*	3.932209575	2.527720961	4.630203943	-1.501785472
80855	chr2	134214660	134215160	501	*	3.635598827	4.35810992	2.12081317	1.551740721
133942	chr7	96797840	96798340	501	*	3.871968065	2.4240354	4.57989363	-1.520229163
58073	chr17	10054359	10054859	501	*	3.456364956	4.202466521	1.824834098	1.581527597
95342	chr4	64306627	64307127	501	*	3.355194295	1.046848728	4.201674108	-1.770714949
7200	chr1	156801214	156801714	501	*	3.108863268	3.933567571	0.981198907	1.733340134
151190	chrX	49763856	49764356	501	*	3.701852753	2.073724131	4.447298721	-1.601598133
81633	chr2	149931094	149931594	501	*	2.98503183	3.848242558	0.518438177	1.785459188
125268	chr6	95841472	95841972	501	*	3.298060247	4.072294946	1.510778668	1.646452279
71657	chr19	58502751	58503251	501	*	3.473952348	1.51214424	4.275767806	-1.690374511
69469	chr19	27179306	27179806	501	*	3.754869842	2.195104747	4.486742711	-1.567799144
78588	chr2	103285493	103285993	501	*	3.539528042	1.694931258	4.323250193	-1.653337605
9366	chr1	185573112	185573612	501	*	3.275819323	4.056273526	1.451265682	1.645140318
142063	chr8	113672817	113673317	501	*	3.759048788	4.471825435	2.289297522	1.500859709
88848	chr3	100081226	100081726	501	*	3.399211941	1.238159142	4.228174068	-1.722731694
80841	chr2	132993079	132993579	501	*	2.936517666	3.815365768	0.302488152	1.793237768
6569	chr1	144078899	144079399	501	*	2.902074471	3.784931936	0.221470052	1.78519011
36494	chr13	63993852	63994352	501	*	3.847996093	2.377274125	4.560986489	-1.503803609
30771	chr12	80925727	80926227	501	*	3.625944679	1.887754483	4.391695424	-1.616413406
71244	chr19	54399534	54400034	501	*	3.542771022	1.729143731	4.32142135	-1.642069837
77238	chr2	75837193	75837693	501	*	3.613022287	1.875857889	4.378592113	-1.612429285
82792	chr2	158734357	158734857	501	*	3.793928575	2.307060851	4.510448657	-1.517141342
37379	chr13	94100421	94100921	501	*	3.526055649	1.66706907	4.312091672	-1.634958608
91110	chr3	144185217	144185717	501	*	3.785185507	2.270837476	4.507603769	-1.514946508
115646	chr6	17559775	17560275	501	*	3.724506639	2.165723004	4.456178969	-1.541939541
2714	chr1	73429561	73430061	501	*	3.475432413	1.603177482	4.263578389	-1.660725529
28803	chr12	44848801	44849301	501	*	3.274913567	4.045571894	1.508565548	1.614556181
129131	chr6	137487371	137487871	501	*	3.448838069	1.480476991	4.251615967	-1.661303632

79283	chr2	117539201	117539701	501	*	3.785747853	2.311983775	4.499406982	-1.510151192
41648	chr14	77608173	77608673	501	*	3.327670056	4.088063673	1.619496459	1.593064167
32330	chr12	109897805	109898305	501	*	3.673353403	2.019871251	4.423640894	-1.570175854
142172	chr8	115755988	115756488	501	*	3.498992698	4.23950226	1.896178585	1.534781032
149675	chr9	111731764	111732264	501	*	2.923697274	3.789754976	0.428161381	1.735381277
41451	chr14	73439811	73440311	501	*	3.577633339	1.881593924	4.33582374	-1.586268894
135314	chr7	118425845	118426345	501	*	3.518434295	4.240531017	2.005600847	1.524746655
137822	chr8	22693093	22693593	501	*	3.21672405	3.993474713	1.414499962	1.632152972
37981	chr13	101829788	101830288	501	*	3.392986837	1.27554362	4.216365313	-1.689229071
11678	chr10	35328303	35328803	501	*	2.853352457	3.770339669	0	1.735479425
76172	chr2	52910914	52911414	501	*	3.239674284	0.708916246	4.109113224	-1.736946691
146590	chr9	60826743	60827243	501	*	3.390184426	1.373460167	4.19991278	-1.679565627
138073	chr8	26301955	26302455	501	*	3.273902758	4.052343803	1.461536895	1.609959329
69543	chr19	28493760	28494260	501	*	3.797582873	2.32533244	4.510909479	-1.510730411
90044	chr3	121609090	121609590	501	*	3.653249163	2.008608456	4.40185978	-1.562302686
2690	chr1	73105716	73106216	501	*	3.443100503	1.535303982	4.236788948	-1.645824713
153014	chrX	161782772	161783272	501	*	3.249718964	0.801291926	4.111110352	-1.730423201
29653	chr12	70502628	70503128	501	*	3.445523568	1.565802749	4.234846934	-1.641751267
71633	chr19	58193632	58194132	501	*	3.642024755	2.014250844	4.387402266	-1.550888474
22826	chr11	85621984	85622484	501	*	3.140849111	3.946713178	1.151242183	1.656663692
63741	chr17	84747210	84747710	501	*	3.773157994	2.209574229	4.50580956	-1.544984664
81045	chr2	137551340	137551840	501	*	3.386620648	1.338558728	4.200712696	-1.673948284
107086	chr5	46950555	46951055	501	*	3.43415222	1.51214424	4.230013097	-1.639193207
107880	chr5	68327143	68327643	501	*	2.880402629	3.758866558	0.250751261	1.730323366
133342	chr7	82338133	82338633	501	*	3.375395775	1.329535227	4.1891848	-1.668692771
22562	chr11	81100821	81101321	501	*	3.703286643	2.165723004	4.430582687	-1.521484731
55633	chr16	49317028	49317528	501	*	3.292240295	1.014250844	4.135271489	-1.698193782
41446	chr14	73399803	73400303	501	*	3.035704187	0	3.999987929	-1.546313632
75813	chr2	45600283	45600783	501	*	3.646819891	2.073724131	4.381400379	-1.54087379
76416	chr2	60289414	60289914	501	*	3.385730706	1.398936221	4.19118922	-1.662650354
132114	chr7	49579231	49579731	501	*	3.366802767	1.266115931	4.187984598	-1.662730472
138218	chr8	31436387	31436887	501	*	3.310573495	4.068232257	1.617444099	1.567416357
46464	chr15	70882505	70883005	501	*	3.506200719	4.231204799	1.979601919	1.505050885
149685	chr9	113670135	113670635	501	*	3.519803472	1.749091532	4.291212371	-1.596092515
86817	chr3	61419725	61420225	501	*	3.242149927	0.749091532	4.107966372	-1.707689154
145905	chr9	54782364	54782864	501	*	3.722518824	2.219377464	4.442548229	-1.513915955
30459	chr12	79366651	79367151	501	*	3.647566041	2.041332642	4.388748438	-1.537064202
137263	chr8	10218313	10218813	501	*	3.193688835	3.976736328	1.353256508	1.604740281
91929	chr4	4856415	4856915	501	*	3.178760915	0.24777069	4.080931336	-1.697081958
41774	chr14	98626423	98626923	501	*	3.313651458	4.060270794	1.679426837	1.552835875
66724	chr18	64660750	64661250	501	*	3.291863019	0.945899805	4.142505944	-1.67410093
27183	chr12	11202409	11202909	501	*	3.177886635	3.966797871	1.300784237	1.600129021
12496	chr10	53114995	53115495	501	*	3.039574349	3.857460425	0.963621878	1.640081665
67734	chr18	86025478	86025978	501	*	2.819099492	3.734032242	0	1.671783208
80474	chr2	129817496	129817996	501	*	3.638674828	1.980480073	4.389851133	-1.531974378
18866	chr11	20258373	20258873	501	*	3.645088966	2.019463154	4.390051475	-1.526373764
78715	chr2	104994299	104994799	501	*	3.620984388	2.073724131	4.350289842	-1.509957275
71612	chr19	57808297	57808797	501	*	3.635430819	1.98623333	4.384907204	-1.520210957
139824	chr8	76748360	76748860	501	*	2.846767907	3.744156493	0	1.672664192
145845	chr9	54153629	54154129	501	*	3.245831346	4.017042172	1.476272532	1.563220177
38164	chr13	104374661	104375161	501	*	3.329460074	1.338558728	4.135510492	-1.621178598

56314	chr16	74161969	74162469	501	*	3.616114527	2.052343803	4.348804153	-1.504937669
75875	chr2	49019312	49019812	501	*	3.017575819	3.832476333	0.963621878	1.619351875
37260	chr13	92490809	92491309	501	*	3.913450013	1.035795384	4.811803209	-1.654015165
36624	chr13	69236339	69236839	501	*	3.223363286	0	4.161315988	-1.582443279
72579	chr2	17297083	17297583	501	*	3.173126611	0.708916246	4.036100093	-1.654044604
6401	chr1	137125568	137126068	501	*	3.386916255	1.480476991	4.18039593	-1.581567617
67799	chr19	3720613	3721113	501	*	4.1048009	2.062875559	4.918046923	-1.615784173
42385	chr15	7026006	7026506	501	*	3.306925754	1.27554362	4.118708627	-1.607958407
45359	chr15	57349481	57349981	501	*	3.547117179	1.875857889	4.300739547	-1.5272804
5295	chr1	126658687	126659187	501	*	3.072221368	3.872642428	1.119847127	1.589700597
139923	chr8	78544465	78544965	501	*	3.340691835	4.078844939	1.749777077	1.501043363
76694	chr2	67989897	67990397	501	*	3.223991758	0	4.163242338	-1.566128282
75093	chr2	33908328	33908828	501	*	3.237599572	1.057818028	4.068901917	-1.628958515
38992	chr14	9525553	9526053	501	*	3.472610226	1.735492432	4.238171829	-1.549006442
151479	chrX	71108031	71108531	501	*	3.526462926	1.881593924	4.275116994	-1.518320709
76681	chr2	67591781	67592281	501	*	3.22541389	0.788178285	4.085671867	-1.645630284
37984	chr13	101866883	101867383	501	*	3.554271492	1.911697231	4.302488419	-1.522664202
44438	chr15	39690319	39690819	501	*	3.160884309	3.932186389	1.390794377	1.559101896
138915	chr8	57430836	57431336	501	*	2.86110105	3.752348583	0.077420035	1.636175246
85612	chr3	33074142	33074642	501	*	2.875733629	3.722390909	0.565806526	1.629337619
136280	chr7	134730605	134731105	501	*	3.439709859	1.674214858	4.210221161	-1.533676754
91675	chr3	153250548	153251048	501	*	2.886179681	3.739178311	0.518438177	1.624360663
34306	chr13	34421573	34422073	501	*	3.522685396	1.899553778	4.267165112	-1.503710326
139940	chr8	78797891	78798391	501	*	3.169048831	3.948057858	1.353256508	1.547897762
140978	chr8	94117202	94117702	501	*	2.915426403	3.747301474	0.73101939	1.603487546
66156	chr18	56742549	56743049	501	*	3.533056362	1.887754483	4.2817928	-1.508238147
90789	chr3	137124821	137125321	501	*	3.453678704	1.755843311	4.212196585	-1.526469508
89043	chr3	102184682	102185182	501	*	3.142602339	0.653196641	4.008062254	-1.613818661
72721	chr2	20376978	20377478	501	*	3.292936602	1.266115931	4.104082667	-1.577096127
151678	chrX	75021459	75021959	501	*	2.976788382	3.793040989	0.912918979	1.577589611
97116	chr4	106100328	106100828	501	*	3.362020548	1.504146517	4.147878627	-1.548575038
40144	chr14	36751103	36751603	501	*	3.279318482	1.293541251	4.084630085	-1.570729018
96346	chr4	93437310	93437810	501	*	3.283174836	1.228483129	4.098176318	-1.572218415
139946	chr8	78940013	78940513	501	*	3.328459317	4.098725175	1.564387016	1.517146852
58556	chr17	21512531	21513530	1000	*	3.375892547	1.543131295	4.157691354	-1.54565167
65647	chr18	42980572	42981072	501	*	3.286401709	1.27554362	4.095301137	-1.567653612
9234	chr1	184244226	184244726	501	*	3.110507667	0.355791741	3.999462628	-1.591317491
33933	chr13	29555007	29555507	501	*	3.109970256	0.355791741	3.99888221	-1.591140712
4319	chr1	91547501	91548001	501	*	3.198429555	1.046848728	4.026195044	-1.584604554
53113	chr16	13305881	13306381	501	*	3.075076009	0.302791982	3.965427398	-1.577647452
36628	chr13	69318269	69318769	501	*	3.088077673	0.355791741	3.975222846	-1.573973306
129177	chr6	140152512	140153012	501	*	2.905165393	3.735767149	0.73101939	1.570091761
112568	chr5	130901652	130902152	501	*	2.998034545	3.796319535	1.059975966	1.552073328
56240	chr16	67613890	67614390	501	*	2.831115259	3.682968769	0.474005799	1.576079229
61991	chr17	55721079	55721579	501	*	3.265520967	1.302791982	4.067471978	-1.54060768
99337	chr4	126496169	126496669	501	*	3.065184958	0.302791982	3.9547524	-1.55754448
63872	chr17	86499626	86500126	501	*	3.121511179	0.76256364	3.973563328	-1.568415691
22592	chr11	81860545	81861045	501	*	3.173650103	1.014250844	4.002403967	-1.557086835
39962	chr14	31569225	31569725	501	*	3.34594501	1.543131295	4.122794329	-1.514332221
63878	chr17	86580642	86581142	501	*	3.125854413	0.62397171	3.992528097	-1.566662011
75130	chr2	34331755	34332255	501	*	3.353872468	1.565802749	4.128240633	-1.511332387

Table S2. ATAC-seq in D5 in mDPCs-CAS9 cells and sgCreb3l1_A8_4: 888 NFRs enriched and 510 NFRs lost at D5_Ctrl

38386	chr13	109583383	109583883	501	*	3.176802642	1.057818028	4.000381738	-1.551050059
9505	chr1	187448087	187448587	501	*	2.909949961	3.729143731	0.824240788	1.549592002
78345	chr2	97099549	97100049	501	*	3.102280649	0.694931258	3.95946398	-1.559806818
112625	chr5	134297566	134298066	501	*	3.337544057	1.543131295	4.112983276	-1.505303773
33097	chr13	6395461	6395961	501	*	3.340358383	1.573466762	4.111110352	-1.505577702
70056	chr19	36696422	36696922	501	*	3.095926093	0.708916246	3.950976354	-1.557102551
87278	chr3	80501039	80501539	501	*	3.251598138	1.247073691	4.059598253	-1.518365732
11683	chr10	36791697	36792197	501	*	2.856524373	3.703660893	0.54231675	1.54233335
4909	chr1	115742552	115743052	501	*	2.89428054	3.713999767	0.804629774	1.530545992
84886	chr3	7694501	7695001	501	*	3.179603936	1.014250844	4.009106425	-1.530553152
42221	chr15	4561374	4561874	501	*	2.936339803	3.742334152	0.945828057	1.513745447
56229	chr16	66768958	66769458	501	*	3.152493601	1.002943913	3.980001332	-1.527410921
43993	chr15	36328811	36329311	501	*	3.156984318	1.025469847	3.98218529	-1.525214387
25089	chr11	110710710	110711210	501	*	3.091873686	0.638658176	3.953747075	-1.531046529
81198	chr2	141997941	141998441	501	*	3.255571959	1.355791741	4.04802406	-1.507245466
39830	chr14	29362409	29362909	501	*	3.037747466	0.373460167	3.919216252	-1.513884714
32090	chr12	107018633	107019133	501	*	2.91484443	3.749214597	0.710089811	1.522856283
37744	chr13	98437004	98437504	501	*	3.104877768	0.839007697	3.946509389	-1.527088483
74793	chr2	32769553	32770053	501	*	3.222698163	1.27554362	4.022343201	-1.501491931
36776	chr13	72409724	72410224	501	*	3.227668702	1.27554362	4.028052792	-1.501871258
79993	chr2	125415443	125415943	501	*	3.159659123	1.078726303	3.978213971	-1.513028392
78343	chr2	96755001	96755501	501	*	3.166825024	0.991547665	3.997567673	-1.511122071
41928	chr14	105698577	105699077	501	*	3.065658335	0.749091532	3.913058311	-1.513880704
64710	chr18	16889066	16889566	501	*	2.864548987	3.690009317	0.73101939	1.501931958
5505	chr1	131489228	131489728	501	*	3.137833862	1.035795384	3.959193955	-1.500677402

Table S2. ATAC-seq in D5 in mDPCs-CAS9 cells and sgCreb3l1_A8_4: 888 NFRs enriched and 510 NFRs lost at D5_Ctrl

p.value	FDR
3.74E-38	4.29E-33
9.09E-37	5.21E-32
1.65E-31	6.31E-27
2.78E-29	7.97E-25
1.88E-26	3.60E-22
3.05E-26	5.00E-22
5.49E-26	7.87E-22
6.80E-25	8.67E-21
8.03E-25	9.22E-21
3.72E-24	3.88E-20
9.27E-24	8.87E-20
5.09E-23	4.49E-19
1.01E-22	7.73E-19
8.89E-22	6.38E-18
2.35E-21	1.59E-17
3.33E-21	2.12E-17
4.80E-21	2.90E-17
6.34E-21	3.63E-17
9.72E-21	5.31E-17
1.22E-20	6.39E-17
1.50E-20	7.17E-17
1.65E-20	7.56E-17
4.17E-20	1.84E-16
4.66E-20	1.93E-16
4.70E-20	1.93E-16
4.90E-20	1.94E-16
1.40E-19	5.19E-16
1.70E-19	5.90E-16
2.27E-19	7.65E-16
2.33E-19	7.65E-16
2.76E-19	8.79E-16
3.87E-19	1.20E-15
3.99E-19	1.20E-15
6.64E-19	1.95E-15
9.27E-19	2.59E-15
1.00E-18	2.74E-15
1.03E-18	2.74E-15
1.59E-18	4.05E-15
1.69E-18	4.22E-15
1.74E-18	4.25E-15
5.94E-18	1.42E-14
9.41E-18	2.20E-14
1.24E-17	2.85E-14
1.63E-17	3.60E-14
2.01E-17	4.36E-14
2.72E-17	5.79E-14
3.30E-17	6.88E-14

3.80E-17	7.66E-14
7.11E-17	1.38E-13
8.92E-17	1.70E-13
1.07E-16	2.00E-13
1.08E-16	2.00E-13
1.16E-16	2.08E-13
1.16E-16	2.08E-13
1.38E-16	2.44E-13
2.17E-16	3.71E-13
2.41E-16	4.07E-13
2.64E-16	4.39E-13
3.62E-16	5.83E-13
3.66E-16	5.83E-13
5.08E-16	7.77E-13
6.14E-16	9.04E-13
6.37E-16	9.26E-13
6.62E-16	9.50E-13
7.30E-16	1.02E-12
9.26E-16	1.28E-12
1.07E-15	1.47E-12
1.20E-15	1.63E-12
1.41E-15	1.86E-12
1.82E-15	2.34E-12
1.83E-15	2.34E-12
2.16E-15	2.67E-12
2.16E-15	2.67E-12
2.37E-15	2.86E-12
2.75E-15	3.29E-12
2.79E-15	3.30E-12
4.00E-15	4.64E-12
4.16E-15	4.78E-12
4.53E-15	5.15E-12
4.95E-15	5.51E-12
5.34E-15	5.89E-12
5.60E-15	6.11E-12
6.43E-15	6.96E-12
6.56E-15	6.97E-12
7.39E-15	7.77E-12
7.65E-15	7.98E-12
8.20E-15	8.45E-12
9.11E-15	9.25E-12
1.08E-14	1.08E-11
1.22E-14	1.20E-11
1.38E-14	1.34E-11
1.54E-14	1.49E-11
1.65E-14	1.58E-11
1.68E-14	1.60E-11
1.92E-14	1.81E-11
2.25E-14	2.08E-11
2.44E-14	2.24E-11
2.70E-14	2.46E-11

3.25E-14	2.91E-11
3.97E-14	3.53E-11
4.11E-14	3.63E-11
5.08E-14	4.35E-11
5.80E-14	4.89E-11
6.60E-14	5.49E-11
6.68E-14	5.50E-11
6.71E-14	5.50E-11
7.38E-14	5.98E-11
7.40E-14	5.98E-11
7.60E-14	6.10E-11
8.24E-14	6.57E-11
8.74E-14	6.92E-11
1.00E-13	7.82E-11
1.06E-13	8.10E-11
1.08E-13	8.19E-11
1.09E-13	8.19E-11
1.10E-13	8.19E-11
1.31E-13	9.67E-11
1.40E-13	1.02E-10
1.40E-13	1.02E-10
1.47E-13	1.06E-10
1.49E-13	1.07E-10
1.63E-13	1.15E-10
1.71E-13	1.20E-10
1.80E-13	1.26E-10
1.86E-13	1.30E-10
1.88E-13	1.30E-10
1.99E-13	1.37E-10
2.15E-13	1.47E-10
2.27E-13	1.52E-10
2.29E-13	1.53E-10
2.69E-13	1.78E-10
2.91E-13	1.89E-10
2.91E-13	1.89E-10
3.19E-13	2.05E-10
3.39E-13	2.16E-10
3.73E-13	2.37E-10
4.29E-13	2.68E-10
4.72E-13	2.93E-10
5.39E-13	3.31E-10
5.49E-13	3.35E-10
6.11E-13	3.71E-10
6.30E-13	3.81E-10
6.61E-13	3.97E-10
7.05E-13	4.19E-10
7.40E-13	4.38E-10
7.47E-13	4.40E-10
8.09E-13	4.74E-10
8.16E-13	4.75E-10
8.32E-13	4.82E-10

8.65E-13	4.99E-10
8.97E-13	5.15E-10
9.27E-13	5.29E-10
9.40E-13	5.31E-10
1.01E-12	5.66E-10
1.04E-12	5.83E-10
1.08E-12	6.02E-10
1.11E-12	6.17E-10
1.18E-12	6.43E-10
1.30E-12	7.04E-10
1.39E-12	7.51E-10
1.47E-12	7.86E-10
1.47E-12	7.86E-10
1.49E-12	7.90E-10
1.50E-12	7.93E-10
1.59E-12	8.36E-10
1.74E-12	9.11E-10
1.81E-12	9.38E-10
1.83E-12	9.43E-10
1.90E-12	9.70E-10
1.92E-12	9.76E-10
1.97E-12	9.97E-10
2.16E-12	1.09E-09
2.22E-12	1.11E-09
2.29E-12	1.14E-09
2.32E-12	1.15E-09
2.58E-12	1.27E-09
2.73E-12	1.34E-09
2.74E-12	1.34E-09
2.77E-12	1.35E-09
2.88E-12	1.39E-09
2.96E-12	1.43E-09
3.19E-12	1.52E-09
3.28E-12	1.56E-09
3.46E-12	1.64E-09
3.47E-12	1.64E-09
3.68E-12	1.73E-09
3.84E-12	1.79E-09
3.93E-12	1.83E-09
4.07E-12	1.88E-09
4.11E-12	1.89E-09
4.20E-12	1.92E-09
4.21E-12	1.92E-09
4.29E-12	1.95E-09
4.61E-12	2.07E-09
4.63E-12	2.07E-09
4.96E-12	2.20E-09
5.73E-12	2.52E-09
5.86E-12	2.56E-09
5.96E-12	2.59E-09
6.01E-12	2.60E-09

6.04E-12	2.61E-09
6.41E-12	2.76E-09
6.63E-12	2.82E-09
6.70E-12	2.83E-09
6.73E-12	2.83E-09
6.75E-12	2.83E-09
7.12E-12	2.95E-09
7.12E-12	2.95E-09
7.20E-12	2.97E-09
7.75E-12	3.18E-09
8.12E-12	3.29E-09
8.16E-12	3.30E-09
8.28E-12	3.32E-09
8.33E-12	3.33E-09
8.42E-12	3.35E-09
8.47E-12	3.36E-09
8.62E-12	3.41E-09
9.07E-12	3.57E-09
9.09E-12	3.57E-09
9.41E-12	3.68E-09
9.44E-12	3.68E-09
9.86E-12	3.82E-09
1.02E-11	3.93E-09
1.06E-11	4.04E-09
1.08E-11	4.12E-09
1.09E-11	4.13E-09
1.09E-11	4.13E-09
1.21E-11	4.54E-09
1.25E-11	4.69E-09
1.27E-11	4.73E-09
1.33E-11	4.95E-09
1.43E-11	5.25E-09
1.45E-11	5.32E-09
1.63E-11	5.90E-09
1.86E-11	6.69E-09
1.87E-11	6.70E-09
1.87E-11	6.70E-09
2.15E-11	7.63E-09
2.19E-11	7.77E-09
2.21E-11	7.81E-09
2.32E-11	8.16E-09
2.56E-11	8.97E-09
2.59E-11	9.01E-09
2.70E-11	9.29E-09
2.95E-11	1.01E-08
3.08E-11	1.05E-08
3.12E-11	1.06E-08
3.19E-11	1.08E-08
3.40E-11	1.14E-08
3.48E-11	1.17E-08
3.62E-11	1.20E-08

3.71E-11	1.23E-08
3.93E-11	1.29E-08
3.97E-11	1.30E-08
4.03E-11	1.31E-08
4.81E-11	1.54E-08
5.00E-11	1.59E-08
5.05E-11	1.60E-08
5.43E-11	1.71E-08
5.45E-11	1.71E-08
5.52E-11	1.73E-08
5.59E-11	1.75E-08
6.16E-11	1.90E-08
6.27E-11	1.93E-08
6.48E-11	1.98E-08
6.72E-11	2.04E-08
6.83E-11	2.06E-08
7.14E-11	2.15E-08
7.72E-11	2.31E-08
7.81E-11	2.33E-08
8.26E-11	2.45E-08
8.42E-11	2.49E-08
8.53E-11	2.50E-08
9.09E-11	2.65E-08
9.11E-11	2.65E-08
9.32E-11	2.70E-08
9.33E-11	2.70E-08
9.86E-11	2.82E-08
1.00E-10	2.86E-08
1.03E-10	2.92E-08
1.08E-10	3.07E-08
1.10E-10	3.12E-08
1.12E-10	3.14E-08
1.13E-10	3.19E-08
1.14E-10	3.20E-08
1.16E-10	3.24E-08
1.17E-10	3.25E-08
1.22E-10	3.39E-08
1.24E-10	3.42E-08
1.31E-10	3.58E-08
1.31E-10	3.58E-08
1.31E-10	3.58E-08
1.33E-10	3.62E-08
1.35E-10	3.67E-08
1.47E-10	3.98E-08
1.48E-10	3.98E-08
1.51E-10	4.05E-08
1.54E-10	4.12E-08
1.57E-10	4.17E-08
1.63E-10	4.33E-08
1.65E-10	4.36E-08
1.69E-10	4.45E-08

1.70E-10	4.48E-08
1.74E-10	4.56E-08
1.75E-10	4.58E-08
1.80E-10	4.70E-08
1.81E-10	4.72E-08
1.88E-10	4.87E-08
1.90E-10	4.93E-08
1.97E-10	5.07E-08
1.99E-10	5.11E-08
2.10E-10	5.36E-08
2.10E-10	5.36E-08
2.18E-10	5.50E-08
2.22E-10	5.59E-08
2.45E-10	6.12E-08
2.48E-10	6.17E-08
2.51E-10	6.23E-08
2.52E-10	6.23E-08
2.59E-10	6.38E-08
2.71E-10	6.63E-08
2.76E-10	6.68E-08
2.77E-10	6.68E-08
2.77E-10	6.68E-08
2.99E-10	7.17E-08
3.06E-10	7.33E-08
3.17E-10	7.55E-08
3.19E-10	7.60E-08
3.29E-10	7.77E-08
3.40E-10	7.98E-08
3.71E-10	8.60E-08
3.73E-10	8.64E-08
3.78E-10	8.70E-08
3.90E-10	8.95E-08
3.91E-10	8.96E-08
3.93E-10	8.98E-08
4.00E-10	9.10E-08
4.07E-10	9.25E-08
4.10E-10	9.30E-08
4.17E-10	9.45E-08
4.25E-10	9.59E-08
4.35E-10	9.80E-08
4.42E-10	9.92E-08
4.65E-10	1.04E-07
4.69E-10	1.04E-07
4.69E-10	1.04E-07
4.77E-10	1.05E-07
4.83E-10	1.06E-07
5.18E-10	1.13E-07
5.22E-10	1.13E-07
5.27E-10	1.14E-07
5.32E-10	1.15E-07
5.37E-10	1.16E-07

5.59E-10	1.20E-07
6.02E-10	1.28E-07
6.08E-10	1.29E-07
6.09E-10	1.29E-07
6.11E-10	1.29E-07
6.15E-10	1.30E-07
6.23E-10	1.31E-07
6.35E-10	1.33E-07
6.57E-10	1.38E-07
6.75E-10	1.41E-07
6.91E-10	1.43E-07
7.25E-10	1.50E-07
7.61E-10	1.57E-07
7.79E-10	1.60E-07
8.19E-10	1.68E-07
8.72E-10	1.78E-07
8.96E-10	1.82E-07
9.33E-10	1.89E-07
9.43E-10	1.90E-07
9.58E-10	1.93E-07
9.64E-10	1.94E-07
9.78E-10	1.96E-07
9.91E-10	1.98E-07
1.06E-09	2.10E-07
1.06E-09	2.11E-07
1.07E-09	2.12E-07
1.10E-09	2.17E-07
1.14E-09	2.24E-07
1.14E-09	2.25E-07
1.15E-09	2.26E-07
1.17E-09	2.29E-07
1.20E-09	2.33E-07
1.20E-09	2.34E-07
1.21E-09	2.35E-07
1.24E-09	2.40E-07
1.28E-09	2.47E-07
1.34E-09	2.57E-07
1.38E-09	2.64E-07
1.40E-09	2.66E-07
1.42E-09	2.70E-07
1.43E-09	2.72E-07
1.46E-09	2.77E-07
1.47E-09	2.78E-07
1.49E-09	2.81E-07
1.50E-09	2.82E-07
1.51E-09	2.85E-07
1.52E-09	2.85E-07
1.53E-09	2.87E-07
1.54E-09	2.88E-07
1.55E-09	2.88E-07
1.57E-09	2.93E-07

1.64E-09	3.02E-07
1.68E-09	3.08E-07
1.69E-09	3.10E-07
1.77E-09	3.23E-07
1.83E-09	3.34E-07
1.87E-09	3.40E-07
1.90E-09	3.45E-07
1.90E-09	3.45E-07
1.95E-09	3.53E-07
1.96E-09	3.54E-07
1.96E-09	3.54E-07
1.97E-09	3.55E-07
1.99E-09	3.58E-07
2.01E-09	3.60E-07
2.02E-09	3.62E-07
2.10E-09	3.74E-07
2.10E-09	3.74E-07
2.11E-09	3.75E-07
2.13E-09	3.78E-07
2.16E-09	3.83E-07
2.21E-09	3.92E-07
2.28E-09	4.01E-07
2.33E-09	4.08E-07
2.34E-09	4.09E-07
2.35E-09	4.11E-07
2.48E-09	4.31E-07
2.52E-09	4.36E-07
2.56E-09	4.42E-07
2.59E-09	4.45E-07
2.60E-09	4.45E-07
2.62E-09	4.49E-07
2.67E-09	4.56E-07
2.68E-09	4.57E-07
2.87E-09	4.87E-07
2.93E-09	4.94E-07
2.96E-09	4.98E-07
3.17E-09	5.29E-07
3.20E-09	5.34E-07
3.28E-09	5.46E-07
3.31E-09	5.49E-07
3.32E-09	5.51E-07
3.39E-09	5.59E-07
3.45E-09	5.65E-07
3.46E-09	5.66E-07
3.49E-09	5.69E-07
3.60E-09	5.83E-07
3.62E-09	5.84E-07
3.70E-09	5.96E-07
3.74E-09	5.98E-07
3.78E-09	6.02E-07
3.82E-09	6.08E-07

4.08E-09	6.43E-07
4.11E-09	6.46E-07
4.25E-09	6.66E-07
4.25E-09	6.66E-07
4.40E-09	6.86E-07
4.43E-09	6.87E-07
4.70E-09	7.27E-07
4.71E-09	7.27E-07
4.90E-09	7.55E-07
4.94E-09	7.60E-07
5.01E-09	7.67E-07
5.02E-09	7.67E-07
5.03E-09	7.67E-07
5.03E-09	7.67E-07
5.32E-09	8.02E-07
5.65E-09	8.48E-07
5.83E-09	8.66E-07
6.18E-09	9.09E-07
6.22E-09	9.13E-07
6.59E-09	9.59E-07
6.67E-09	9.69E-07
6.73E-09	9.75E-07
6.86E-09	9.92E-07
6.88E-09	9.95E-07
6.92E-09	9.97E-07
7.03E-09	1.01E-06
7.09E-09	1.02E-06
7.45E-09	1.06E-06
7.50E-09	1.06E-06
7.55E-09	1.07E-06
7.69E-09	1.09E-06
7.74E-09	1.09E-06
7.87E-09	1.10E-06
8.08E-09	1.13E-06
8.19E-09	1.14E-06
8.53E-09	1.19E-06
8.56E-09	1.19E-06
8.58E-09	1.19E-06
8.66E-09	1.20E-06
8.71E-09	1.20E-06
8.83E-09	1.22E-06
9.19E-09	1.26E-06
9.56E-09	1.30E-06
9.78E-09	1.33E-06
9.95E-09	1.35E-06
1.01E-08	1.37E-06
1.02E-08	1.38E-06
1.03E-08	1.40E-06
1.08E-08	1.45E-06
1.09E-08	1.46E-06
1.11E-08	1.49E-06

1.11E-08	1.49E-06
1.13E-08	1.51E-06
1.16E-08	1.54E-06
1.16E-08	1.54E-06
1.17E-08	1.55E-06
1.18E-08	1.57E-06
1.19E-08	1.58E-06
1.20E-08	1.59E-06
1.20E-08	1.59E-06
1.21E-08	1.60E-06
1.24E-08	1.63E-06
1.26E-08	1.65E-06
1.27E-08	1.66E-06
1.28E-08	1.67E-06
1.30E-08	1.70E-06
1.35E-08	1.75E-06
1.37E-08	1.77E-06
1.44E-08	1.85E-06
1.48E-08	1.89E-06
1.48E-08	1.89E-06
1.50E-08	1.92E-06
1.56E-08	1.98E-06
1.58E-08	2.00E-06
1.58E-08	2.00E-06
1.59E-08	2.00E-06
1.60E-08	2.02E-06
1.62E-08	2.03E-06
1.63E-08	2.04E-06
1.64E-08	2.05E-06
1.64E-08	2.05E-06
1.69E-08	2.11E-06
1.70E-08	2.12E-06
1.79E-08	2.23E-06
1.82E-08	2.26E-06
1.93E-08	2.39E-06
1.97E-08	2.43E-06
1.99E-08	2.45E-06
1.99E-08	2.46E-06
2.04E-08	2.51E-06
2.06E-08	2.52E-06
2.08E-08	2.55E-06
2.11E-08	2.58E-06
2.13E-08	2.60E-06
2.14E-08	2.61E-06
2.22E-08	2.70E-06
2.26E-08	2.75E-06
2.28E-08	2.76E-06
2.29E-08	2.76E-06
2.32E-08	2.80E-06
2.33E-08	2.80E-06
2.36E-08	2.83E-06

2.39E-08	2.86E-06
2.41E-08	2.88E-06
2.41E-08	2.88E-06
2.41E-08	2.88E-06
2.43E-08	2.89E-06
2.44E-08	2.91E-06
2.45E-08	2.91E-06
2.54E-08	2.99E-06
2.58E-08	3.04E-06
2.59E-08	3.05E-06
2.64E-08	3.09E-06
2.66E-08	3.12E-06
2.72E-08	3.17E-06
2.83E-08	3.28E-06
2.88E-08	3.33E-06
2.92E-08	3.38E-06
2.92E-08	3.38E-06
2.93E-08	3.38E-06
2.97E-08	3.42E-06
3.02E-08	3.47E-06
3.03E-08	3.48E-06
3.09E-08	3.54E-06
3.09E-08	3.54E-06
3.14E-08	3.59E-06
3.16E-08	3.60E-06
3.16E-08	3.60E-06
3.29E-08	3.73E-06
3.44E-08	3.88E-06
3.46E-08	3.89E-06
3.61E-08	4.05E-06
3.75E-08	4.17E-06
3.87E-08	4.29E-06
3.90E-08	4.31E-06
3.92E-08	4.33E-06
3.93E-08	4.33E-06
3.96E-08	4.36E-06
4.11E-08	4.52E-06
4.13E-08	4.53E-06
4.16E-08	4.55E-06
4.26E-08	4.65E-06
4.32E-08	4.71E-06
4.33E-08	4.71E-06
4.39E-08	4.76E-06
4.61E-08	4.97E-06
4.64E-08	5.01E-06
4.67E-08	5.03E-06
4.67E-08	5.03E-06
4.75E-08	5.10E-06
4.87E-08	5.21E-06
4.90E-08	5.24E-06
4.93E-08	5.26E-06

4.93E-08	5.26E-06
4.99E-08	5.32E-06
5.03E-08	5.34E-06
5.08E-08	5.39E-06
5.16E-08	5.47E-06
5.19E-08	5.49E-06
5.32E-08	5.60E-06
5.44E-08	5.73E-06
5.48E-08	5.75E-06
5.54E-08	5.80E-06
5.61E-08	5.86E-06
5.68E-08	5.92E-06
5.69E-08	5.93E-06
5.82E-08	6.04E-06
5.86E-08	6.08E-06
5.87E-08	6.08E-06
5.96E-08	6.16E-06
6.15E-08	6.33E-06
6.24E-08	6.40E-06
6.38E-08	6.54E-06
6.38E-08	6.54E-06
6.40E-08	6.55E-06
6.43E-08	6.57E-06
6.56E-08	6.69E-06
6.58E-08	6.70E-06
6.63E-08	6.73E-06
6.64E-08	6.74E-06
6.76E-08	6.84E-06
6.77E-08	6.84E-06
6.93E-08	6.98E-06
7.11E-08	7.13E-06
7.19E-08	7.21E-06
7.21E-08	7.22E-06
7.26E-08	7.24E-06
7.45E-08	7.39E-06
7.47E-08	7.41E-06
7.49E-08	7.41E-06
7.56E-08	7.46E-06
7.57E-08	7.46E-06
7.60E-08	7.49E-06
7.65E-08	7.53E-06
7.80E-08	7.65E-06
7.93E-08	7.77E-06
7.97E-08	7.81E-06
8.01E-08	7.84E-06
8.14E-08	7.94E-06
8.18E-08	7.97E-06
8.37E-08	8.11E-06
8.44E-08	8.16E-06
8.61E-08	8.29E-06
8.69E-08	8.37E-06

8.77E-08	8.42E-06
8.82E-08	8.46E-06
8.96E-08	8.58E-06
8.99E-08	8.60E-06
9.00E-08	8.60E-06
9.15E-08	8.73E-06
9.25E-08	8.80E-06
9.37E-08	8.89E-06
9.41E-08	8.92E-06
9.56E-08	9.04E-06
9.57E-08	9.04E-06
9.74E-08	9.19E-06
9.89E-08	9.32E-06
1.01E-07	9.49E-06
1.01E-07	9.51E-06
1.02E-07	9.54E-06
1.03E-07	9.67E-06
1.07E-07	9.94E-06
1.08E-07	1.00E-05
1.08E-07	1.00E-05
1.08E-07	1.00E-05
1.09E-07	1.01E-05
1.12E-07	1.03E-05
1.13E-07	1.04E-05
1.15E-07	1.05E-05
1.16E-07	1.06E-05
1.16E-07	1.06E-05
1.17E-07	1.07E-05
1.18E-07	1.08E-05
1.21E-07	1.10E-05
1.21E-07	1.10E-05
1.22E-07	1.11E-05
1.23E-07	1.11E-05
1.23E-07	1.11E-05
1.24E-07	1.12E-05
1.25E-07	1.12E-05
1.26E-07	1.13E-05
1.28E-07	1.14E-05
1.33E-07	1.18E-05
1.33E-07	1.18E-05
1.38E-07	1.22E-05
1.41E-07	1.24E-05
1.41E-07	1.24E-05
1.43E-07	1.26E-05
1.47E-07	1.29E-05
1.49E-07	1.30E-05
1.50E-07	1.31E-05
1.57E-07	1.36E-05
1.59E-07	1.38E-05
1.61E-07	1.39E-05
1.64E-07	1.41E-05

1.67E-07	1.44E-05
1.70E-07	1.46E-05
1.71E-07	1.46E-05
1.71E-07	1.46E-05
1.75E-07	1.49E-05
1.76E-07	1.50E-05
1.78E-07	1.51E-05
1.79E-07	1.52E-05
1.80E-07	1.52E-05
1.86E-07	1.56E-05
1.88E-07	1.57E-05
1.98E-07	1.65E-05
1.98E-07	1.65E-05
2.01E-07	1.67E-05
2.01E-07	1.67E-05
2.04E-07	1.69E-05
2.07E-07	1.71E-05
2.07E-07	1.71E-05
2.10E-07	1.72E-05
2.10E-07	1.73E-05
2.11E-07	1.73E-05
2.13E-07	1.74E-05
2.15E-07	1.76E-05
2.18E-07	1.77E-05
2.21E-07	1.80E-05
2.22E-07	1.80E-05
2.32E-07	1.87E-05
2.35E-07	1.89E-05
2.36E-07	1.89E-05
2.37E-07	1.90E-05
2.39E-07	1.92E-05
2.40E-07	1.92E-05
2.41E-07	1.92E-05
2.48E-07	1.97E-05
2.49E-07	1.98E-05
2.56E-07	2.03E-05
2.58E-07	2.03E-05
2.61E-07	2.06E-05
2.64E-07	2.07E-05
2.68E-07	2.09E-05
2.71E-07	2.11E-05
2.71E-07	2.11E-05
2.72E-07	2.12E-05
2.73E-07	2.12E-05
2.78E-07	2.16E-05
2.80E-07	2.17E-05
2.81E-07	2.17E-05
2.82E-07	2.17E-05
2.87E-07	2.20E-05
2.87E-07	2.21E-05
2.89E-07	2.22E-05

2.90E-07	2.22E-05
2.90E-07	2.22E-05
2.91E-07	2.23E-05
2.97E-07	2.27E-05
2.98E-07	2.27E-05
3.04E-07	2.32E-05
3.05E-07	2.32E-05
3.12E-07	2.38E-05
3.16E-07	2.40E-05
3.17E-07	2.41E-05
3.26E-07	2.47E-05
3.29E-07	2.48E-05
3.43E-07	2.58E-05
3.50E-07	2.62E-05
3.51E-07	2.63E-05
3.55E-07	2.65E-05
3.60E-07	2.68E-05
3.61E-07	2.68E-05
3.67E-07	2.72E-05
3.67E-07	2.72E-05
3.68E-07	2.72E-05
3.78E-07	2.79E-05
3.86E-07	2.83E-05
3.88E-07	2.84E-05
3.91E-07	2.86E-05
4.00E-07	2.92E-05
4.06E-07	2.95E-05
4.06E-07	2.95E-05
4.07E-07	2.96E-05
4.11E-07	2.98E-05
4.24E-07	3.06E-05
4.27E-07	3.08E-05
4.36E-07	3.13E-05
4.38E-07	3.14E-05
4.39E-07	3.15E-05
4.49E-07	3.21E-05
4.52E-07	3.23E-05
4.59E-07	3.26E-05
4.71E-07	3.34E-05
4.72E-07	3.35E-05
4.77E-07	3.37E-05
4.79E-07	3.39E-05
4.84E-07	3.41E-05
4.89E-07	3.45E-05
4.91E-07	3.46E-05
4.98E-07	3.50E-05
5.00E-07	3.51E-05
5.03E-07	3.52E-05
5.04E-07	3.53E-05
5.12E-07	3.58E-05
5.15E-07	3.60E-05

5.35E-07	3.73E-05
5.37E-07	3.73E-05
5.39E-07	3.75E-05
5.43E-07	3.76E-05
5.50E-07	3.81E-05
5.52E-07	3.81E-05
5.57E-07	3.84E-05
5.62E-07	3.87E-05
5.73E-07	3.93E-05
5.80E-07	3.98E-05
5.84E-07	4.00E-05
5.87E-07	4.01E-05
5.99E-07	4.07E-05
5.99E-07	4.08E-05
6.01E-07	4.08E-05
6.02E-07	4.08E-05
6.02E-07	4.08E-05
6.05E-07	4.10E-05
6.13E-07	4.15E-05
6.16E-07	4.16E-05
6.24E-07	4.21E-05
6.24E-07	4.21E-05
6.29E-07	4.24E-05
6.32E-07	4.25E-05
6.39E-07	4.29E-05
6.44E-07	4.31E-05
6.48E-07	4.33E-05
6.48E-07	4.33E-05
6.51E-07	4.35E-05
6.60E-07	4.40E-05
6.61E-07	4.40E-05
6.64E-07	4.42E-05
6.70E-07	4.45E-05
6.83E-07	4.53E-05
6.91E-07	4.57E-05
6.91E-07	4.57E-05
6.98E-07	4.60E-05
7.17E-07	4.70E-05
7.26E-07	4.74E-05
7.29E-07	4.76E-05
7.32E-07	4.77E-05
7.38E-07	4.81E-05
7.45E-07	4.84E-05
7.58E-07	4.91E-05
7.62E-07	4.94E-05
7.79E-07	5.02E-05
7.93E-07	5.09E-05
7.98E-07	5.11E-05
8.06E-07	5.15E-05
8.12E-07	5.19E-05
8.18E-07	5.21E-05

Table S2. ATAC-seq in D5 in mDPCs-CAS9 cells and sgCreb3l1_A8_4: 888 NFRs enriched and 510 NFRs lost at D5_Ctrl

8.27E-07	5.26E-05
8.36E-07	5.31E-05
8.37E-07	5.31E-05
8.40E-07	5.33E-05
8.53E-07	5.39E-05
8.68E-07	5.46E-05
8.68E-07	5.46E-05
8.73E-07	5.49E-05
8.76E-07	5.50E-05
9.02E-07	5.65E-05
9.03E-07	5.65E-05
9.08E-07	5.67E-05
9.10E-07	5.68E-05
9.13E-07	5.69E-05
9.31E-07	5.79E-05
9.33E-07	5.80E-05
9.38E-07	5.83E-05
9.43E-07	5.86E-05
9.55E-07	5.92E-05
9.55E-07	5.92E-05
9.75E-07	6.01E-05
9.76E-07	6.01E-05
9.85E-07	6.05E-05
9.86E-07	6.05E-05
9.91E-07	6.07E-05
1.01E-06	6.17E-05
1.01E-06	6.20E-05
1.02E-06	6.23E-05
1.02E-06	6.23E-05
1.03E-06	6.29E-05
1.04E-06	6.32E-05
1.04E-06	6.34E-05
1.05E-06	6.40E-05
1.06E-06	6.42E-05
1.06E-06	6.45E-05
1.08E-06	6.57E-05
1.10E-06	6.65E-05
1.10E-06	6.67E-05
1.10E-06	6.67E-05
1.11E-06	6.70E-05
1.11E-06	6.70E-05
1.12E-06	6.73E-05
1.12E-06	6.77E-05
1.13E-06	6.78E-05
1.14E-06	6.86E-05
1.15E-06	6.87E-05
1.16E-06	6.96E-05
1.16E-06	6.96E-05
1.17E-06	7.02E-05
1.21E-06	7.22E-05
1.21E-06	7.23E-05

1.27E-06	7.53E-05
1.28E-06	7.53E-05
1.28E-06	7.53E-05
1.29E-06	7.59E-05
1.30E-06	7.64E-05
1.30E-06	7.65E-05
1.32E-06	7.71E-05
1.32E-06	7.73E-05
1.33E-06	7.75E-05
1.33E-06	7.75E-05
1.33E-06	7.76E-05
1.33E-06	7.76E-05
1.33E-06	7.78E-05
1.33E-06	7.78E-05
1.36E-06	7.87E-05
1.40E-06	8.12E-05
1.42E-06	8.17E-05
1.43E-06	8.26E-05
1.44E-06	8.31E-05
1.45E-06	8.35E-05
1.47E-06	8.41E-05
1.47E-06	8.44E-05
1.49E-06	8.54E-05
1.49E-06	8.54E-05
1.51E-06	8.62E-05
1.52E-06	8.65E-05
1.52E-06	8.68E-05
1.55E-06	8.82E-05
1.56E-06	8.89E-05
1.58E-06	8.95E-05
1.58E-06	8.98E-05
1.60E-06	9.05E-05
1.63E-06	9.17E-05
1.64E-06	9.27E-05
1.65E-06	9.31E-05
1.66E-06	9.32E-05
1.66E-06	9.33E-05
1.68E-06	9.40E-05
1.69E-06	9.45E-05
1.69E-06	9.47E-05
1.70E-06	9.50E-05
1.72E-06	9.58E-05
1.74E-06	9.64E-05
1.76E-06	9.75E-05
1.77E-06	9.80E-05
1.80E-06	9.92E-05
1.81E-06	9.97E-05
1.82E-06	0.00010009
1.83E-06	0.000100504
1.86E-06	0.0001016
1.86E-06	0.000101701

1.89E-06	0.000103373
1.89E-06	0.000103476
1.90E-06	0.000103627
1.92E-06	0.000104377
1.93E-06	0.000105215
1.96E-06	0.000106323
1.96E-06	0.000106445
1.98E-06	0.000107118
1.99E-06	0.000107349
1.99E-06	0.000107384
2.08E-06	0.000111638
2.17E-06	0.000115512
2.18E-06	0.000116039
2.21E-06	0.000117319
2.22E-06	0.000118187
2.24E-06	0.000118857
2.27E-06	0.000120407
2.27E-06	0.000120545
2.28E-06	0.000120705
2.30E-06	0.000121435
2.31E-06	0.000121998
2.34E-06	0.000123249
2.41E-06	0.000126521
2.44E-06	0.000127768
2.48E-06	0.000129784
2.60E-06	0.000134711
2.61E-06	0.000135194
2.62E-06	0.000135378
2.63E-06	0.000135758
2.68E-06	0.00013838
2.70E-06	0.000138816
2.71E-06	0.000139679
2.75E-06	0.000141454
2.79E-06	0.000142973
2.81E-06	0.000143608
2.86E-06	0.000145705
2.87E-06	0.00014617
2.88E-06	0.000146361
2.88E-06	0.000146361
2.95E-06	0.000149539
2.96E-06	0.000149607
2.98E-06	0.000150551
3.00E-06	0.000151379
3.00E-06	0.000151379
3.04E-06	0.000153491
3.06E-06	0.000154027
3.07E-06	0.000154315
3.13E-06	0.00015653
3.20E-06	0.000159578
3.26E-06	0.000161963
3.27E-06	0.000162367

3.33E-06	0.000165125
3.39E-06	0.000167174
3.42E-06	0.000168066
3.45E-06	0.000169164
3.55E-06	0.000173632
3.56E-06	0.00017387
3.62E-06	0.000176392
3.64E-06	0.00017716
3.65E-06	0.000177619
3.68E-06	0.000178883
3.69E-06	0.000179278
3.72E-06	0.000180524
3.76E-06	0.000181801
3.80E-06	0.000183464
3.80E-06	0.000183478
3.82E-06	0.000184344
3.85E-06	0.000185675
3.86E-06	0.000185833
3.93E-06	0.000188831
3.95E-06	0.000189343
3.97E-06	0.000190279
4.00E-06	0.000191593
4.02E-06	0.000192001
4.06E-06	0.000193852
4.12E-06	0.000195981
4.12E-06	0.00019601
4.17E-06	0.000197633
4.17E-06	0.00019769
4.17E-06	0.000197718
4.25E-06	0.000200539
4.26E-06	0.000201185
4.27E-06	0.000201355
4.32E-06	0.000203226
4.36E-06	0.00020517
4.42E-06	0.000206973
4.47E-06	0.000208963
4.52E-06	0.000210989
4.58E-06	0.000213271
4.62E-06	0.000215075
4.64E-06	0.000215511
4.77E-06	0.000220167
4.84E-06	0.000222945
4.86E-06	0.000223683
4.86E-06	0.000223812
4.96E-06	0.000227394
4.96E-06	0.000227426
4.99E-06	0.000228379
5.06E-06	0.000231376
5.08E-06	0.000231834
5.14E-06	0.000234012
5.15E-06	0.000234195

5.18E-06	0.000235278
5.18E-06	0.000235329
5.23E-06	0.000237153
5.29E-06	0.00023928
5.40E-06	0.000243307
5.44E-06	0.00024459
5.48E-06	0.000246083
5.49E-06	0.000246492
5.52E-06	0.000247841
5.55E-06	0.000248856
5.63E-06	0.000251863
5.65E-06	0.000252455
5.68E-06	0.000253624
5.75E-06	0.000256641
5.78E-06	0.000257473
5.87E-06	0.000260722
5.89E-06	0.000261554
5.93E-06	0.000262812
5.93E-06	0.000262812
6.00E-06	0.000265831
6.10E-06	0.000269117
6.10E-06	0.000269117
6.13E-06	0.000270272
6.20E-06	0.000272806
6.24E-06	0.000274502
6.33E-06	0.000277898
6.36E-06	0.000278579
6.43E-06	0.000281157
6.49E-06	0.00028261
6.52E-06	0.000283854
6.52E-06	0.000283854
6.55E-06	0.000284374
6.55E-06	0.000284374
6.59E-06	0.000285827
6.67E-06	0.000288556
6.70E-06	0.000289755
6.71E-06	0.000289847
6.78E-06	0.000292045
6.79E-06	0.000292594
6.85E-06	0.000294364
6.97E-06	0.000298763
6.98E-06	0.000299037
7.02E-06	0.000300385
7.04E-06	0.000300867
7.09E-06	0.000302673
7.17E-06	0.000305474
7.19E-06	0.000306141
7.23E-06	0.000307462
7.28E-06	0.000309133
7.32E-06	0.000310667
7.46E-06	0.000315809

7.55E-06	0.000319078
7.58E-06	0.000319939
7.69E-06	0.000323684
7.73E-06	0.000325181
7.82E-06	0.000327768
7.94E-06	0.000331941
7.97E-06	0.000332857
7.97E-06	0.000332857
8.04E-06	0.000335347
8.11E-06	0.000337584
8.20E-06	0.000341022
8.30E-06	0.000343906
8.35E-06	0.000345417
8.57E-06	0.000353008
8.64E-06	0.00035531
8.78E-06	0.000359826
8.80E-06	0.000360635
9.03E-06	0.000368394
9.11E-06	0.000371239
9.62E-06	0.000389417
9.70E-06	0.000391841
9.71E-06	0.00039223
9.72E-06	0.000392444
9.80E-06	0.000394855
9.89E-06	0.000397752
9.91E-06	0.000398435
1.00E-05	0.000400756
1.01E-05	0.000403236
1.01E-05	0.000404881
1.02E-05	0.000407421
1.03E-05	0.000410199
1.04E-05	0.000412358
1.04E-05	0.000412358
1.05E-05	0.000417493
1.07E-05	0.000421925
1.08E-05	0.000425975
1.10E-05	0.00043248
1.11E-05	0.000434359
1.11E-05	0.000434359
1.11E-05	0.000435708
1.12E-05	0.000437951
1.12E-05	0.000437951
1.15E-05	0.000446333
1.15E-05	0.000446966
1.16E-05	0.000448429
1.16E-05	0.000449327
1.18E-05	0.000455292
1.18E-05	0.000456861
1.20E-05	0.000462586
1.22E-05	0.00046999
1.23E-05	0.000472345

1.23E-05	0.000473476
1.24E-05	0.000473925
1.24E-05	0.000474636
1.25E-05	0.00047671
1.25E-05	0.000479371
1.26E-05	0.000480038
1.26E-05	0.00048102
1.26E-05	0.000481718
1.27E-05	0.000483502
1.27E-05	0.000484333
1.28E-05	0.000486861
1.31E-05	0.000494801
1.31E-05	0.00049681
1.32E-05	0.000499569
1.33E-05	0.000500657
1.35E-05	0.000508385
1.39E-05	0.000518867
1.40E-05	0.000521196
1.40E-05	0.000522953
1.41E-05	0.000525137
1.43E-05	0.000531906
1.44E-05	0.00053277
1.44E-05	0.000533153
1.46E-05	0.000539958
1.47E-05	0.000540148
1.47E-05	0.000541596
1.49E-05	0.000546066
1.49E-05	0.000548132
1.51E-05	0.000552108
1.54E-05	0.000560963
1.54E-05	0.000562861
1.55E-05	0.000565682
1.56E-05	0.000567573
1.56E-05	0.000568895
1.57E-05	0.000570786
1.57E-05	0.00057079
1.59E-05	0.00057575
1.63E-05	0.000586853
1.63E-05	0.000588908
1.64E-05	0.000590148
1.64E-05	0.000590148
1.65E-05	0.000592698
1.66E-05	0.000595235
1.68E-05	0.000601284
1.68E-05	0.000602912
1.71E-05	0.000609816
1.72E-05	0.000614366
1.76E-05	0.000626059
1.76E-05	0.000626437
1.77E-05	0.000630371
1.78E-05	0.000631466

1.80E-05	0.000637659
1.81E-05	0.000640422
1.81E-05	0.000641861
1.82E-05	0.000642048
1.82E-05	0.000642752
1.84E-05	0.000649611
1.84E-05	0.000649691
1.85E-05	0.000652443
1.86E-05	0.000656297
1.87E-05	0.000657906
1.87E-05	0.000657961
1.87E-05	0.000658306
1.87E-05	0.000658306
1.88E-05	0.00066071
1.89E-05	0.000663853
1.90E-05	0.000665897
1.91E-05	0.000668914
1.92E-05	0.000670944
1.92E-05	0.000671526
1.96E-05	0.00068531
1.97E-05	0.000687088
1.97E-05	0.000687877
1.98E-05	0.000691017
2.03E-05	0.000702233
2.04E-05	0.000706785
2.05E-05	0.000710598
2.06E-05	0.000711603
2.07E-05	0.000713594
2.10E-05	0.000723722
2.13E-05	0.000733085
2.13E-05	0.000734459
2.17E-05	0.000745023
2.18E-05	0.000746917
2.21E-05	0.000755462
2.22E-05	0.000758656
2.22E-05	0.00075869
2.23E-05	0.000759756
2.26E-05	0.000767672
2.27E-05	0.000768771
2.37E-05	0.000797623
2.41E-05	0.000808488
2.48E-05	0.000828418
2.56E-05	0.000849978
2.60E-05	0.000860399
2.67E-05	0.000877213
2.68E-05	0.000878502
2.72E-05	0.000890708
2.76E-05	0.000903246
2.77E-05	0.000904304
2.80E-05	0.00091461
2.81E-05	0.000915841

2.83E-05	0.000922429
2.85E-05	0.000926328
2.91E-05	0.000940991
2.91E-05	0.000941739
2.92E-05	0.000945153
2.94E-05	0.000948839
2.96E-05	0.000954854
2.97E-05	0.000958363
2.97E-05	0.000958363
2.99E-05	0.000964587
3.00E-05	0.000966629
3.02E-05	0.000970478
3.05E-05	0.000976914
3.05E-05	0.000977047
3.07E-05	0.000981467
3.09E-05	0.000986436
3.12E-05	0.000993922
3.13E-05	0.000995072
3.14E-05	0.00099969
3.15E-05	0.001001342
3.21E-05	0.001018616
3.22E-05	0.001020082
3.23E-05	0.001021745
3.24E-05	0.001025537
3.27E-05	0.001031176
3.28E-05	0.001034163
3.33E-05	0.001047135
3.33E-05	0.001047328
3.38E-05	0.001059327
3.43E-05	0.001070686
3.46E-05	0.001078222
3.49E-05	0.00108519
3.60E-05	0.001114423
3.60E-05	0.001114478
3.60E-05	0.001114516
3.67E-05	0.001131392
3.67E-05	0.001131728
3.76E-05	0.001155451
3.83E-05	0.001170098
3.89E-05	0.001184115
3.94E-05	0.001197174
3.98E-05	0.001207063
4.10E-05	0.001236766
4.12E-05	0.001243009
4.22E-05	0.001268249
4.31E-05	0.001289898
4.45E-05	0.001324718
4.45E-05	0.001325687
4.47E-05	0.001329694
4.48E-05	0.001331716
4.55E-05	0.001348626

4.63E-05	0.001368792
4.75E-05	0.001396099
4.78E-05	0.001402451
4.85E-05	0.001419989
4.91E-05	0.001433098
4.96E-05	0.001445576
4.97E-05	0.00144631
4.99E-05	0.001451651
5.03E-05	0.001459627
5.04E-05	0.001463762
5.07E-05	0.001469416
5.11E-05	0.001478463
5.13E-05	0.001482583
5.16E-05	0.001488821
5.18E-05	0.001495498
5.18E-05	0.001495498
5.21E-05	0.00150014
5.24E-05	0.001505579
5.45E-05	0.001554147
5.56E-05	0.001576088
5.77E-05	0.001621823
5.79E-05	0.001624289
5.92E-05	0.001658616
5.97E-05	0.001670608
6.00E-05	0.001676648
6.03E-05	0.001682922
6.14E-05	0.001706173
6.22E-05	0.001722314
6.24E-05	0.001726215
6.36E-05	0.001750974
6.38E-05	0.001755895
6.45E-05	0.001771788
6.54E-05	0.001790056
6.56E-05	0.001794741
6.61E-05	0.001806404
6.67E-05	0.001818451
6.70E-05	0.001824472
6.71E-05	0.001825881
6.79E-05	0.001844503
7.23E-05	0.001941126
7.36E-05	0.001967627
7.51E-05	0.001999122
7.51E-05	0.001999122
7.69E-05	0.002040277
7.89E-05	0.002078125
8.01E-05	0.002105129
8.09E-05	0.00212273
8.09E-05	0.00212273
8.16E-05	0.002135778
8.17E-05	0.00213816
8.27E-05	0.002155648

8.44E-05	0.002189439
8.44E-05	0.002190956
8.50E-05	0.002198258
8.52E-05	0.002203634
8.54E-05	0.002206459
8.63E-05	0.002223932
9.34E-05	0.002362292
9.38E-05	0.002371944
9.56E-05	0.002405323
9.72E-05	0.002433019
9.75E-05	0.002438327
9.76E-05	0.002440127
9.89E-05	0.002464828
0.000100169	0.002487282
0.000100191	0.00248729
0.00010079	0.002500517
0.000101966	0.002524804
0.000102562	0.002538268
0.000102621	0.002538812
0.000103411	0.002554823
0.000106789	0.00262003
0.000109015	0.002659724
0.000110644	0.002694324
0.000114457	0.002762541
0.000115441	0.002782792

Table S3. RNAseq in D5 in mDPCs-CAS9 cells and sg*Creb3l1* _A8_4: 1007 downregulated genes and 1027 upregulated genes

ens_gene	ext_gene	target_id	pval	b	D5_A8_4_1_tpm
ENSMUSG00000087651	*1500009L16Rik*	ENSMUST00000150459.2	0.001460584	-1.515405599	0.721732
ENSMUSG00000026051	*1500015O10Rik*	ENSMUST00000027217.8	6.62E-06	-3.705058795	0
ENSMUSG00000026831	*1700007K13Rik*	ENSMUST00000086370.10	5.94E-05	-1.910682547	1.23863
ENSMUSG00000052794	*1700030K09Rik*	ENSMUST00000064853.12	3.85E-06	0.689860469	10.1813
ENSMUSG00000071653	*1810009A15Rik*	ENSMUST00000096251.9	0.000163063	-0.43827419	64.0883
ENSMUSG00000021290	*2010107E04Rik*	ENSMUST00000021719.6	0.001282743	-0.338447888	473.745
ENSMUSG00000071252	*2210408I21Rik*	ENSMUST00000168779.2	3.48E-09	-1.006512326	1.52485
ENSMUSG00000031983	*2310022B05Rik*	ENSMUST00000034464.7	4.92E-05	0.658235272	130.607
ENSMUSG00000031242	*2610002M06Rik*	ENSMUST00000120722.1	0.000128212	0.381051258	16.5013
ENSMUSG00000071793	*2610005L07Rik*	ENSMUST00000139264.1	0.003669618	0.379713632	53.9533
ENSMUSG00000059482	*2610301B20Rik*	ENSMUST00000080517.13	0.000410698	-0.403523299	21.4185
ENSMUSG00000049916	*2610318N02Rik*	ENSMUST00000207116.1	1.42E-06	-0.946150954	5.61491
ENSMUSG00000092486	*2610524H06Rik*	ENSMUST00000100850.4	3.89E-05	-0.487185532	47.3142
ENSMUSG00000031984	*2810004N23Rik*	ENSMUST00000034465.8	3.64E-07	0.581359952	27.9512
ENSMUSG00000062822	*4833420G17Rik*	ENSMUST00000026519.9	0.00341885	-0.411064996	11.0664
ENSMUSG00000032057	*4833427G06Rik*	ENSMUST00000170947.2	2.47E-06	-1.677668604	1.42723
ENSMUSG00000036934	*4921524J17Rik*	ENSMUST00000047749.6	0.000107941	0.496263863	28.0936
ENSMUSG00000027309	*4930402H24Rik*	ENSMUST00000044766.14	0.000904466	-0.326229685	10.9842
ENSMUSG00000008129	*4930432K21Rik*	ENSMUST00000118856.7	0.006897977	0.53881225	5.38089
ENSMUSG00000071796	*6820431F20Rik*	ENSMUST00000153748.1	4.40E-10	0.537904939	85.9743
ENSMUSG00000039901	*9130011E15Rik*	ENSMUST00000045396.7	0.001128728	-0.390839298	11.3259
ENSMUSG00000054293	*A630033H20Rik*	ENSMUST00000167673.1	1.77E-06	0.681095374	54.8753
ENSMUSG00000031960	*Aars*	ENSMUST00000034441.7	0.001367367	0.583870011	190.862
ENSMUSG00000023938	*Aars2*	ENSMUST00000024733.7	3.99E-05	-0.573226608	5.64948
ENSMUSG00000057880	*Abat*	ENSMUST00000065987.13	0.003348812	-1.213004059	0.20845
ENSMUSG00000026944	*Abca2*	ENSMUST00000102919.3	9.45E-08	0.520693474	13.5395
ENSMUSG00000018800	*Abca5*	ENSMUST00000043961.11	0.008074814	-0.254802217	10.2342
ENSMUSG00000020620	*Abca8b*	ENSMUST00000020948.14	5.45E-05	0.877294237	1.91901
ENSMUSG00000041797	*Abca9*	ENSMUST00000044850.3	6.66E-06	-1.00609092	6.46465
ENSMUSG00000031974	*Abcb10*	ENSMUST00000075578.6	4.31E-11	0.786170536	13.3473
ENSMUSG00000022822	*Abcc5*	ENSMUST00000079158.12	0.000194211	0.39096946	16.8548
ENSMUSG00000030834	*Abcc6*	ENSMUST00000002850.7	0.006160891	1.024938687	0.87929
ENSMUSG00000021240	*Abcd4*	ENSMUST00000021666.5	0.00603011	-0.430657698	8.15479
ENSMUSG00000058355	*Abce1*	ENSMUST00000080536.7	6.00E-09	0.499556685	70.8598
ENSMUSG00000040396	*Abhd13*	ENSMUST00000048216.5	3.35E-09	0.792675557	14.3479
ENSMUSG00000042073	*Abhd14b*	ENSMUST00000048527.14	2.52E-05	0.61822605	19.5253
ENSMUSG00000032540	*Abhd5*	ENSMUST00000156520.7	2.33E-06	0.52569464	24.7668
ENSMUSG00000035258	*Abi3bp*	ENSMUST00000096013.9	9.35E-25	-1.287898084	7.58183
ENSMUSG00000110900	*AC162446.2*	ENSMUST00000215536.1	0.009509193	0.422557699	57.4978
ENSMUSG00000098411	*AC165954.1*	ENSMUST00000183925.7	5.84E-06	-3.683254498	0.0670103
ENSMUSG00000026003	*Acadl*	ENSMUST00000027153.5	0.005829868	0.268293625	60.8213
ENSMUSG00000062908	*Acadm*	ENSMUST00000072697.12	0.000186545	-0.334784889	71.8098
ENSMUSG00000026499	*Acbd3*	ENSMUST00000027780.5	1.73E-05	0.377472247	62.4394
ENSMUSG00000038000	*Acd*	ENSMUST00000212642.1	0.000113369	0.794868586	20.9362
ENSMUSG00000028405	*Aco1*	ENSMUST00000102973.3	0.002537552	-0.264432155	43.4397
ENSMUSG00000052392	*Acot4*	ENSMUST00000021652.4	0.004318638	1.02744419	0.450112
ENSMUSG00000018796	*Acsl1*	ENSMUST00000034046.12	0.006764811	0.55666334	19.0985
ENSMUSG00000006457	*Actn3*	ENSMUST00000006626.3	2.91E-07	-0.749909247	6.18977
ENSMUSG00000025228	*Actr1a*	ENSMUST00000040270.4	3.53E-05	-0.35046452	86.7181
ENSMUSG00000031555	*Adam9*	ENSMUST00000208247.2	0.002589533	0.714282442	73.8841
ENSMUSG00000022893	*Adamts1*	ENSMUST00000023610.14	0.006872345	-0.238313182	195.086
ENSMUSG00000036545	*Adamts2*	ENSMUST00000040523.8	7.49E-09	-0.753355331	22.0913
ENSMUSG00000043635	*Adamts3*	ENSMUST00000061427.9	9.51E-09	2.937202233	1.51067
ENSMUSG00000020262	*Adarb1*	ENSMUST00000020496.13	0.009035617	0.526338145	3.10366
ENSMUSG00000020654	*Adcy3*	ENSMUST00000020984.8	1.50E-05	-0.746055393	3.2983
ENSMUSG00000031659	*Adcy7*	ENSMUST00000171456.8	6.32E-18	1.829261363	9.11727
ENSMUSG00000025026	*Add3*	ENSMUST00000050096.13	3.51E-05	-0.386581086	25.313
ENSMUSG00000031486	*Adgra2*	ENSMUST00000033876.13	0.004538112	0.323269533	91.0628
ENSMUSG00000031785	*Adgrg1*	ENSMUST00000093271.7	6.96E-06	1.002637423	3.60158
ENSMUSG00000037605	*Adgrl3*	ENSMUST00000124117.1	0.000197223	-0.950200617	1.26777
ENSMUSG00000028138	*Adh5*	ENSMUST00000005964.6	0.005164771	-0.256157914	82.4457
ENSMUSG00000026457	*Adipor1*	ENSMUST00000027727.14	0.00289276	0.262597482	83.4033
ENSMUSG00000054136	*Adm2*	ENSMUST00000066991.5	0.000118826	0.886316578	58.8827
ENSMUSG00000039041	*Adrm1*	ENSMUST00000061437.4	0.008524496	-0.276307425	102.761
ENSMUSG00000015961	*Adss*	ENSMUST00000016105.8	0.008476895	0.235278676	61.7289
ENSMUSG00000030609	*Aen*	ENSMUST00000107421.7	1.03E-05	-0.589123479	20.4694
ENSMUSG00000031967	*Afg3l1*	ENSMUST00000001520.12	2.43E-12	0.776898612	21.0407
ENSMUSG00000049659	*Aftph*	ENSMUST00000035350.11	0.001428025	0.339901221	16.9666

ENSMUSG00000031521	*Aga*	ENSMUST00000033920.5	2.71E-06	0.436153247	164.251
ENSMUSG00000041530	*Ago1*	ENSMUST00000097888.9	1.58E-09	-0.523415374	22.8857
ENSMUSG00000041936	*Agrn*	ENSMUST00000180572.1	0.001216865	-0.629243928	59.1214
ENSMUSG00000049115	*Agtr1a*	ENSMUST00000066412.7	6.87E-05	-3.307419342	0.0944533
ENSMUSG00000068122	*Agtr2*	ENSMUST00000089188.8	4.63E-24	-1.853544667	1.43697
ENSMUSG00000027893	*Ahcyl1*	ENSMUST00000029490.14	2.60E-10	-0.529942418	83.7875
ENSMUSG00000019986	*Ahi1*	ENSMUST00000105525.11	0.004845338	0.317793301	11.8605
ENSMUSG00000069833	*Ahnak*	ENSMUST00000092956.2	3.37E-05	-0.393838972	18.046
ENSMUSG00000019256	*Ahr*	ENSMUST00000116436.8	0.000253383	0.617900801	49.4698
ENSMUSG00000021575	*Ahrr*	ENSMUST00000022059.13	0.000532324	0.812008297	9.94616
ENSMUSG00000074384	*AI429214*	ENSMUST00000098825.4	0.000358238	0.762310002	4.90045
ENSMUSG00000047423	*AI837181*	ENSMUST00000159759.1	0.009785744	-0.274646493	53.3245
ENSMUSG00000024847	*Aip*	ENSMUST00000025767.13	0.002880541	-0.370676345	37.8732
ENSMUSG00000026817	*Ak1*	ENSMUST00000068271.4	6.63E-19	-0.97453974	30.3353
ENSMUSG00000028792	*Ak2*	ENSMUST00000102604.10	0.000955975	-0.29189662	98.2315
ENSMUSG00000024782	*Ak3*	ENSMUST00000025696.4	1.25E-05	-0.406065866	36.6796
ENSMUSG00000078941	*Ak6*	ENSMUST00000084721.7	2.64E-06	-0.432108945	107.627
ENSMUSG00000038729	*Akap2*	ENSMUST00000102903.7	0.00012246	-0.341171899	40.2798
ENSMUSG00000039166	*Akap7*	ENSMUST00000100012.2	0.002816379	0.525490319	9.24838
ENSMUSG00000002625	*Akap8l*	ENSMUST00000050214.7	0.001422621	-0.37113023	19.2606
ENSMUSG00000040407	*Akap9*	ENSMUST00000044492.9	0.000227427	0.390287855	5.12056
ENSMUSG00000028291	*Akirin2*	ENSMUST00000084299.5	0.000711532	-0.360770612	40.6087
ENSMUSG00000028692	*Akr1a1*	ENSMUST00000030455.14	0.005289444	-0.238827059	611.367
ENSMUSG00000021211	*Akr1c12*	ENSMUST00000021632.4	0.006474232	-2.833575272	0
ENSMUSG00000021213	*Akr1c13*	ENSMUST00000021634.3	2.67E-13	1.76084735	12.8025
ENSMUSG00000001729	*Akt1*	ENSMUST00000001780.9	0.007116422	-0.347391757	167.433
ENSMUSG00000031667	*Aktip*	ENSMUST00000125257.2	9.41E-05	0.58572168	21.3005
ENSMUSG00000020256	*Aldh1l2*	ENSMUST00000020497.13	0.009199757	0.75902807	109.514
ENSMUSG00000028737	*Aldh4a1*	ENSMUST00000039818.9	6.26E-14	-0.925934724	7.41458
ENSMUSG00000021238	*Aldh6a1*	ENSMUST00000085192.6	0.008314493	-0.30258198	12.7635
ENSMUSG00000063362	*Alg11*	ENSMUST00000072572.12	0.000102662	0.489982674	13.6475
ENSMUSG00000039740	*Alg2*	ENSMUST00000044148.2	0.000973052	-0.337822454	19.7222
ENSMUSG00000087247	*Alkal1*	ENSMUST00000133144.3	0.003203547	1.019362141	5.7254
ENSMUSG00000042650	*Alkbh5*	ENSMUST00000044250.3	5.76E-05	0.335553094	76.4065
ENSMUSG00000002661	*Alkbh7*	ENSMUST00000002737.6	1.33E-05	-0.857613433	9.38998
ENSMUSG00000031751	*Amfr*	ENSMUST00000053766.13	2.97E-19	0.778240138	85.068
ENSMUSG00000050947	*Amigo1*	ENSMUST00000050909.6	0.001193427	-0.504725952	2.87131
ENSMUSG00000048218	*Amigo2*	ENSMUST00000053106.5	1.08E-05	-1.411116188	1.30596
ENSMUSG00000042225	*Ammecr1*	ENSMUST00000041317.2	0.001241273	-0.354699721	15.2557
ENSMUSG00000068250	*Amn1*	ENSMUST00000111535.7	0.003639346	0.598760494	9.56072
ENSMUSG00000013076	*Amotl1*	ENSMUST00000013220.7	0.000242358	-0.357180814	28.396
ENSMUSG00000027889	*Ampd2*	ENSMUST00000078912.6	0.000107133	-0.784051171	11.4826
ENSMUSG00000026634	*Angel2*	ENSMUST00000066632.13	0.002425687	0.330201329	22.5664
ENSMUSG00000033544	*Angptl1*	ENSMUST00000027885.7	0.000271974	1.992771447	1.04343
ENSMUSG00000031508	*Ankrd10*	ENSMUST00000211690.1	0.000749076	0.567694076	8.96011
ENSMUSG00000035569	*Ankrd11*	ENSMUST00000098334.12	4.37E-08	0.672308613	29.8874
ENSMUSG00000034647	*Ankrd12*	ENSMUST00000038116.11	0.005035219	-0.418915504	5.91206
ENSMUSG00000032231	*Anxa2*	ENSMUST00000034756.14	0.004451591	-0.248720202	690.895
ENSMUSG00000029994	*Anxa4*	ENSMUST00000001187.14	1.51E-09	0.506536183	226.845
ENSMUSG00000018340	*Anxa6*	ENSMUST00000102727.2	7.30E-06	-0.400049665	102.476
ENSMUSG00000021814	*Anxa7*	ENSMUST00000065504.16	0.000669083	0.45317852	43.8349
ENSMUSG00000019326	*Aoc3*	ENSMUST00000103105.9	2.45E-11	-1.166318095	1.64408
ENSMUSG00000031731	*Ap1g1*	ENSMUST00000093157.12	9.15E-11	0.625472641	19.9487
ENSMUSG00000003033	*Ap1m1*	ENSMUST00000003117.14	3.73E-08	0.555560944	55.0803
ENSMUSG00000031367	*Ap1s2*	ENSMUST00000033734.13	0.005034994	-0.611516887	5.09338
ENSMUSG00000054702	*Ap1s3*	ENSMUST00000162342.7	1.17E-07	1.154910371	3.4252
ENSMUSG00000021686	*Ap3b1*	ENSMUST00000022196.4	0.00192715	-0.271251727	40.8409
ENSMUSG00000037032	*Apbb1*	ENSMUST00000189378.6	0.006892777	0.331306128	23.1826
ENSMUSG00000020135	*Apc2*	ENSMUST00000105359.7	0.007514692	-0.352542063	4.66882
ENSMUSG00000035960	*Apex1*	ENSMUST00000049411.11	0.000258749	-0.383421353	55.7233
ENSMUSG00000010911	*Apip*	ENSMUST00000011055.6	0.004087127	-0.335616466	55.3375
ENSMUSG00000042759	*Apobr*	ENSMUST00000039522.7	1.75E-06	0.622080483	12.3519
ENSMUSG00000057346	*Apol9a*	ENSMUST00000081776.9	7.67E-07	-0.591156872	95.0389
ENSMUSG00000006589	*Aprt*	ENSMUST00000006764.8	3.93E-06	0.483378775	149.428
ENSMUSG00000004655	*Aqp1*	ENSMUST00000004774.3	0.003511477	0.741691375	25.7388
ENSMUSG00000044217	*Aqp5*	ENSMUST00000169082.1	9.52E-09	2.106455162	17.6528
ENSMUSG00000040383	*Aqr*	ENSMUST00000043160.12	0.000265109	-0.323569045	28.0308
ENSMUSG00000046532	*Ar*	ENSMUST00000052837.8	3.34E-35	2.711351685	3.00891
ENSMUSG00000037999	*Arap2*	ENSMUST00000076623.7	7.49E-06	0.715652792	5.52247
ENSMUSG00000062421	*Arf2*	ENSMUST00000057921.9	8.80E-08	-0.545285651	30.4255
ENSMUSG00000051853	*Arf3*	ENSMUST00000053183.10	0.007469624	0.223319253	119.547
ENSMUSG00000040459	*Arglu1*	ENSMUST00000208252.1	0.000130799	0.662089539	24.7722

Table S3. RNAseq in D5 in mDPCs-CAS9 cells and sgCreb3l1 _A8_4: 1007 downregulated genes and 1027 upregulated genes

ENSMUSG00000037148	*Arhgap10*	ENSMUST00000076316.5	0.000553088	0.619080482	43.5481
ENSMUSG00000039031	*Arhgap18*	ENSMUST00000039557.8	0.000980812	0.348244822	20.7144
ENSMUSG00000049807	*Arhgap23*	ENSMUST00000121799.7	0.005419998	-0.472520949	11.219
ENSMUSG00000057315	*Arhgap24*	ENSMUST00000112853.7	3.29E-06	-1.142398619	3.14279
ENSMUSG00000024043	*Arhgap28*	ENSMUST00000024840.11	1.21E-14	-0.688131791	23.3659
ENSMUSG00000022799	*Arhgap31*	ENSMUST00000023487.4	0.002440136	-0.295477423	19.5952
ENSMUSG00000050730	*Arhgap42*	ENSMUST00000093893.11	1.62E-06	-0.543593787	6.64755
ENSMUSG00000031355	*Arhgap6*	ENSMUST00000112131.8	0.00603675	-0.670233219	2.07089
ENSMUSG00000071176	*Arhgef10*	ENSMUST00000084207.11	0.000444934	0.704357376	24.2116
ENSMUSG00000052921	*Arhgef15*	ENSMUST00000108671.1	2.65E-05	0.716252637	3.95207
ENSMUSG00000004568	*Arhgef18*	ENSMUST00000004684.12	2.80E-05	0.436981195	19.7427
ENSMUSG00000033542	*Arhgef5*	ENSMUST00000031750.13	1.46E-05	0.404896921	25.3073
ENSMUSG00000031133	*Arhgef6*	ENSMUST00000175900.1	3.22E-05	0.930980084	23.7746
ENSMUSG00000031511	*Arhgef7*	ENSMUST00000074856.12	4.97E-06	0.536336433	27.766
ENSMUSG00000007880	*Arid1a*	ENSMUST00000145664.8	0.004219686	-0.336341636	15.1271
ENSMUSG00000060904	*Arl1*	ENSMUST00000116234.8	0.007407942	0.251612527	243.344
ENSMUSG00000031776	*Arl2bp*	ENSMUST00000034228.15	2.49E-09	0.617180141	111.1
ENSMUSG00000025035	*Arl3*	ENSMUST00000026009.8	0.000299837	-0.378561768	100.458
ENSMUSG00000049866	*Arl4c*	ENSMUST00000159814.1	0.003348326	0.836938956	27.9137
ENSMUSG00000035199	*Arl6ip5*	ENSMUST00000044681.6	1.37E-05	0.557903835	283.291
ENSMUSG00000030105	*Arl8b*	ENSMUST00000032196.8	0.001979136	0.25831364	101.017
ENSMUSG00000050394	*Armcx6*	ENSMUST00000052431.11	0.000111106	-0.572756188	12.5428
ENSMUSG00000036412	*Arsi*	ENSMUST00000040359.5	0.00287633	-0.478132388	10.6878
ENSMUSG00000048040	*Arxes2*	ENSMUST00000058119.8	0.00584434	-0.312585973	72.26
ENSMUSG00000003559	*As3mt*	ENSMUST00000003655.7	2.49E-05	-0.905299374	3.14072
ENSMUSG00000031591	*Asah1*	ENSMUST00000034000.14	1.99E-06	0.397479133	167.555
ENSMUSG00000036995	*Asap3*	ENSMUST00000047526.7	0.000780307	-0.486048023	8.87161
ENSMUSG00000044475	*Ascc1*	ENSMUST00000050516.13	0.001523083	0.475554791	25.2968
ENSMUSG00000005470	*Asf1b*	ENSMUST00000005607.8	6.10E-08	0.61364738	41.9627
ENSMUSG00000028053	*Ash1l*	ENSMUST00000186583.6	0.003822548	-0.515686797	6.58799
ENSMUSG00000031575	*Ash2l*	ENSMUST00000166078.8	8.69E-07	0.667604071	33.4296
ENSMUSG00000023017	*Asic1*	ENSMUST00000023758.7	0.000109322	0.573395708	7.82035
ENSMUSG00000038276	*Asic3*	ENSMUST00000049346.9	0.000565937	-3.653220574	0
ENSMUSG00000052456	*Asna1*	ENSMUST00000064314.9	1.05E-05	0.419082219	188.431
ENSMUSG00000028207	*Asph*	ENSMUST00000078139.12	0.001065286	-0.356469636	30.3946
ENSMUSG00000029348	*Asphd2*	ENSMUST00000031291.8	0.009046131	-1.424557753	2.02421
ENSMUSG00000042548	*Asxl1*	ENSMUST00000109790.1	0.006471272	-0.23876894	21.8202
ENSMUSG00000022360	*Atad2*	ENSMUST00000038194.3	0.001849752	0.264311251	139.819
ENSMUSG00000023027	*Atf1*	ENSMUST00000023769.10	0.006297196	0.247529938	81.0751
ENSMUSG00000026628	*Atf3*	ENSMUST00000027941.13	8.19E-05	1.083754344	6.70636
ENSMUSG00000042406	*Atf4*	ENSMUST00000109605.3	0.004360731	0.261977511	407.638
ENSMUSG00000026663	*Atf6*	ENSMUST00000027974.6	2.94E-05	0.38945258	17.5989
ENSMUSG00000015461	*Atf6b*	ENSMUST00000015605.14	0.006473204	-0.260997437	42.6146
ENSMUSG00000024773	*Atg2a*	ENSMUST00000045351.12	0.000999789	-0.466921276	3.34559
ENSMUSG00000079418	*Atg4a*	ENSMUST00000112971.1	0.005835458	-0.34544588	11.9643
ENSMUSG00000037621	*Atoh8*	ENSMUST00000042646.7	0.004999336	-0.754456365	10.2604
ENSMUSG00000031441	*Atp11a*	ENSMUST00000033818.9	5.02E-12	0.678524965	26.0682
ENSMUSG00000031862	*Atp13a1*	ENSMUST00000034326.6	4.16E-06	0.46588957	38.5345
ENSMUSG00000019943	*Atp2b1*	ENSMUST00000020107.7	0.008710972	0.334242106	37.3565
ENSMUSG00000016252	*Atp5e*	ENSMUST00000016396.7	0.000146854	-0.381134086	459.769
ENSMUSG00000006057	*Atp5g1*	ENSMUST00000107684.1	0.001110635	0.392799737	157.705
ENSMUSG00000018770	*Atp5g3*	ENSMUST00000018914.2	7.76E-06	-0.400114207	312.97
ENSMUSG00000031007	*Atp6ap2*	ENSMUST00000033313.2	0.002226065	0.255378907	263.344
ENSMUSG00000013160	*Atp6v0d1*	ENSMUST00000013304.7	0.000649654	0.3716503	164.39
ENSMUSG00000039347	*Atp6v0e2*	ENSMUST00000040361.7	4.40E-09	0.86150589	20.3356
ENSMUSG00000006273	*Atp6v1b2*	ENSMUST00000006435.7	1.06E-11	0.626084323	66.6258
ENSMUSG00000021114	*Atp6v1d*	ENSMUST00000219316.1	0.00070977	-0.490443684	40.1213
ENSMUSG00000039529	*Atp8b1*	ENSMUST00000025482.8	7.26E-10	-2.290553108	0.0670073
ENSMUSG00000054428	*Atpif1*	ENSMUST00000067496.6	0.00449359	-0.248200975	779.967
ENSMUSG00000046876	*Atxn1*	ENSMUST00000091628.10	0.002039633	-0.338913548	4.54722
ENSMUSG00000027496	*Aurka*	ENSMUST00000109140.9	0.002878776	-0.276935773	71.051
ENSMUSG00000029787	*Avl9*	ENSMUST00000031805.10	2.09E-05	0.385879624	22.3027
ENSMUSG00000078349	*AW011738*	ENSMUST00000105140.2	1.27E-15	-1.338257833	4.05156
ENSMUSG00000002602	*Axl*	ENSMUST00000002677.10	0.001619074	0.269357224	142.324
ENSMUSG00000028789	*Azin2*	ENSMUST00000030581.9	0.004677695	-0.618933895	7.74662
ENSMUSG00000034780	*B3galt1*	ENSMUST00000112346.2	5.16E-06	-0.790771128	1.99679
ENSMUSG00000013418	*B4galnt2*	ENSMUST00000038343.6	0.001288092	1.195958459	5.20623
ENSMUSG00000055629	*B4galnt4*	ENSMUST00000048002.6	3.00E-11	1.387275682	4.94784
ENSMUSG00000047379	*B4gat1*	ENSMUST00000053705.6	0.000167632	-0.459936962	15.75
ENSMUSG00000031820	*Babam1*	ENSMUST00000002473.9	0.00242456	0.300892996	83.0473
ENSMUSG00000032086	*Bace1*	ENSMUST00000034591.10	6.07E-06	-0.478118525	13.6763
ENSMUSG00000024959	*Bad*	ENSMUST00000113423.9	0.000581611	-0.402459098	52.5323

Table S3. RNAseq in D5 in mDPCs-CAS9 cells and sgCreb3l1 _A8_4: 1007 downregulated genes and 1027 upregulated genes

ENSMUSG00000042215	*Bag2*	ENSMUST00000044691.8	0.007518532	0.261482081	66.8081
ENSMUSG00000037316	*Bag4*	ENSMUST00000038498.9	8.18E-05	0.447355046	12.7977
ENSMUSG00000021381	*Barx1*	ENSMUST00000021813.4	3.21E-06	-2.587081513	1.91639
ENSMUSG00000006464	*Bbs1*	ENSMUST00000053506.6	0.005431228	-0.370428876	3.96391
ENSMUSG00000031755	*Bbs2*	ENSMUST00000034206.5	2.48E-05	0.620905997	9.42302
ENSMUSG00000025235	*Bbs4*	ENSMUST00000026265.7	0.006886982	-0.426247943	5.2361
ENSMUSG00000022641	*Bbx*	ENSMUST00000138166.7	0.001210714	0.29658229	16.3562
ENSMUSG00000081137	*BC022960*	ENSMUST00000119987.1	0.001740319	-0.686812624	2.14992
ENSMUSG00000053684	*BC048403*	ENSMUST00000065600.7	0.003438394	0.520440417	6.42133
ENSMUSG00000005687	*Bcas2*	ENSMUST00000005830.14	0.001338363	-0.30584954	76.3442
ENSMUSG00000030268	*Bcat1*	ENSMUST00000111742.7	8.15E-09	0.572155757	48.9366
ENSMUSG00000030826	*Bcat2*	ENSMUST00000033098.15	8.03E-05	-0.468116887	22.6033
ENSMUSG00000030802	*Bckdk*	ENSMUST00000124533.2	0.004489306	0.316366127	50.0783
ENSMUSG00000007659	*Bcl2l1*	ENSMUST00000007803.11	0.001414009	-0.429362352	18.2744
ENSMUSG00000027381	*Bcl2l11*	ENSMUST00000110341.8	0.009166005	-0.285905076	11.139
ENSMUSG00000037608	*Bclaf1*	ENSMUST00000185800.6	0.00013928	-0.484129721	19.0255
ENSMUSG00000048482	*Bdnf*	ENSMUST00000111045.8	3.11E-06	0.749283455	8.01361
ENSMUSG00000030103	*Bhlhe40*	ENSMUST00000032194.10	0.000128493	0.606398624	75.2239
ENSMUSG00000067787	*Blcap*	ENSMUST00000109528.8	0.001204976	-0.469613146	33.9611
ENSMUSG00000057506	*Bloc1s2*	ENSMUST00000079033.4	0.003664645	-0.354722355	50.1559
ENSMUSG00000026577	*Blzf1*	ENSMUST00000120447.7	0.003489781	0.423299647	12.9576
ENSMUSG00000039004	*Bmp6*	ENSMUST00000171970.2	0.000129564	-2.368854943	0.358002
ENSMUSG00000031963	*Bmper*	ENSMUST00000071982.6	3.27E-07	1.059615694	24.6047
ENSMUSG00000022687	*Boc*	ENSMUST00000114634.2	3.31E-06	-0.523681549	14.7635
ENSMUSG00000022070	*Bora*	ENSMUST00000022656.6	0.00098447	-0.4766388	6.69043
ENSMUSG00000062376	*Borcs7*	ENSMUST00000074912.7	0.006499582	-0.549634054	2.65478
ENSMUSG00000002413	*Braf*	ENSMUST00000002487.14	9.15E-05	0.406267234	13.2016
ENSMUSG00000041147	*Brca2*	ENSMUST00000202313.1	9.44E-08	0.705707496	4.90792
ENSMUSG00000031201	*Brcc3*	ENSMUST00000033544.13	0.001310803	0.458600308	7.90023
ENSMUSG00000022387	*Brd1*	ENSMUST00000109381.7	0.008042271	0.239914795	35.4926
ENSMUSG00000031660	*Brd7*	ENSMUST00000034085.7	6.08E-10	0.619566511	32.5028
ENSMUSG00000035131	*Brinp3*	ENSMUST00000166814.7	5.46E-14	5.587011688	12.1128
ENSMUSG00000071657	*Bscl2*	ENSMUST00000086058.12	7.77E-12	-0.880163738	18.44
ENSMUSG00000032589	*Bsn*	ENSMUST00000124763.1	0.003464582	1.321223579	0.914337
ENSMUSG00000040565	*Btaf1*	ENSMUST00000099494.3	0.005792092	-0.271078858	10.0034
ENSMUSG00000041702	*Btbd7*	ENSMUST00000223554.1	0.00137706	-0.284109731	18.0909
ENSMUSG00000021660	*Btf3*	ENSMUST00000152704.7	0.003736367	-0.244712235	486.044
ENSMUSG00000036478	*Btg1*	ENSMUST00000038377.8	0.000276277	0.343290988	80.2442
ENSMUSG00000020423	*Btg2*	ENSMUST00000020692.6	4.89E-08	-0.632780222	12.2636
ENSMUSG00000040084	*Bub1b*	ENSMUST00000038341.7	0.008271252	-0.225487293	64.219
ENSMUSG00000023988	*Bysl*	ENSMUST00000024783.8	0.003177904	-0.332785667	11.912
ENSMUSG00000045912	*C2cd4c*	ENSMUST00000178228.2	0.002140916	0.714483411	2.46305
ENSMUSG00000046679	*C87436*	ENSMUST00000050497.13	0.005038711	0.454594331	10.7529
ENSMUSG00000036707	*Cab39*	ENSMUST00000113360.7	0.003886288	0.256801341	57.6649
ENSMUSG00000024112	*Cacna1h*	ENSMUST00000078496.11	0.002918284	-1.938190027	2.87783
ENSMUSG00000057914	*Cacnb2*	ENSMUST00000114723.8	4.01E-05	1.251066167	4.09179
ENSMUSG00000032076	*Cadm1*	ENSMUST00000114547.7	5.24E-06	-1.306775156	0.320589
ENSMUSG00000064115	*Cadm2*	ENSMUST00000120594.7	0.002376069	2.504436143	1.45751
ENSMUSG00000029767	*Calu*	ENSMUST00000031779.16	0.003467217	0.306576027	1091.92
ENSMUSG00000039145	*Camk1d*	ENSMUST00000044009.13	0.001039409	-0.342230279	8.75259
ENSMUSG00000046447	*Camk2n1*	ENSMUST00000050918.3	0.003525689	0.401259461	6.04616
ENSMUSG00000056737	*Capg*	ENSMUST00000114071.7	3.75E-06	0.655811209	65.2981
ENSMUSG00000027184	*Caprin1*	ENSMUST00000111147.7	5.16E-05	-0.351180053	180.176
ENSMUSG00000028745	*Capzb*	ENSMUST00000102508.9	3.85E-06	-0.387484353	237.571
ENSMUSG00000032185	*Carm1*	ENSMUST00000034703.14	0.001805313	-0.395871858	25.335
ENSMUSG00000031012	*Cask*	ENSMUST00000033321.10	0.005919653	0.246058483	45.9975
ENSMUSG00000031628	*Casp3*	ENSMUST00000211115.1	9.67E-09	0.545848059	69.0881
ENSMUSG00000027187	*Cat*	ENSMUST00000028610.9	0.001150908	-0.310883077	37.6725
ENSMUSG00000007655	*Cav1*	ENSMUST00000007799.12	0.008948405	-0.357561845	190.083
ENSMUSG00000045954	*Cavin2*	ENSMUST00000051572.7	0.008344089	-0.225303616	73.5805
ENSMUSG00000028348	*Cavin4*	ENSMUST00000030033.4	2.97E-05	1.040208892	10.7907
ENSMUSG00000031885	*Cbfb*	ENSMUST00000109392.8	4.95E-17	0.755580692	128.25
ENSMUSG00000053411	*Cbx7*	ENSMUST00000089293.10	0.00256237	0.467410495	8.14653
ENSMUSG00000036686	*Cc2d1a*	ENSMUST00000040383.8	0.00560186	0.39684138	40.8398
ENSMUSG00000033712	*Ccar2*	ENSMUST00000035612.5	0.006833492	-0.280197839	15.6384
ENSMUSG00000026578	*Ccdc181*	ENSMUST00000027867.6	0.000203708	0.533801454	15.8024
ENSMUSG00000028795	*Ccdc28b*	ENSMUST00000030586.14	0.001961399	-0.731618412	7.00654
ENSMUSG00000026676	*Ccdc3*	ENSMUST00000027988.7	0.003550602	-2.68455719	0.31077
ENSMUSG00000027160	*Ccdc34*	ENSMUST00000028580.11	0.008459511	-0.253374088	43.0272
ENSMUSG00000038127	*Ccdc50*	ENSMUST00000100026.9	0.000964739	0.387731639	33.4325
ENSMUSG00000090946	*Ccdc71l*	ENSMUST00000172332.3	0.006513441	0.285966309	18.2388
ENSMUSG00000022665	*Ccdc80*	ENSMUST00000099498.8	1.86E-09	0.860648462	125.608

ENSMUSG00000024732	*Ccdc86*	ENSMUST00000025639.6	0.008753609	-0.362569948	8.20925
ENSMUSG00000030301	*Ccdc91*	ENSMUST00000032441.13	0.0005002	0.340434105	50.0612
ENSMUSG00000037979	*Ccdc92*	ENSMUST00000036206.13	0.00113495	0.62582905	4.58873
ENSMUSG00000041431	*Ccnb1*	ENSMUST00000072119.14	0.000180512	-0.319315993	93.5031
ENSMUSG00000032218	*Ccnb2*	ENSMUST00000034742.7	0.007036686	0.248630539	109.456
ENSMUSG00000070348	*Ccnd1*	ENSMUST00000093962.4	2.69E-06	-0.929178829	19.6853
ENSMUSG00000000184	*Ccnd2*	ENSMUST00000000188.11	0.003361421	-0.337201287	38.9405
ENSMUSG00000020326	*Ccng1*	ENSMUST00000020576.7	8.01E-20	-0.772530588	57.3833
ENSMUSG00000021258	*Ccnk*	ENSMUST00000221167.1	1.22E-07	-0.543696511	19.9437
ENSMUSG00000031971	*Ccsap*	ENSMUST00000034452.11	0.002925934	0.577437274	5.93008
ENSMUSG00000039578	*Ccser1*	ENSMUST00000134710.2	0.003936498	1.405714856	3.3411
ENSMUSG00000001416	*Cct3*	ENSMUST00000001452.13	0.000211751	-0.313054177	123.133
ENSMUSG00000047139	*Cd24a*	ENSMUST00000058714.9	2.42E-12	1.225963615	13.106
ENSMUSG00000016494	*Cd34*	ENSMUST00000110815.8	2.01E-10	1.166917096	26.9633
ENSMUSG00000024610	*Cd74*	ENSMUST00000050487.14	3.81E-08	0.634035208	92.05
ENSMUSG00000075122	*Cd80*	ENSMUST00000099816.2	2.99E-11	-2.162067378	0.696673
ENSMUSG00000027215	*Cd82*	ENSMUST00000028644.10	0.001468405	-0.293827896	89.5518
ENSMUSG00000030342	*Cd9*	ENSMUST00000032492.8	4.18E-07	0.508206966	511.819
ENSMUSG00000038416	*Cdc16*	ENSMUST00000043962.8	1.33E-09	0.530402125	117.305
ENSMUSG00000024769	*Cdc42bpg*	ENSMUST00000025681.7	1.52E-07	-0.731621253	2.71561
ENSMUSG00000045664	*Cdc42ep2*	ENSMUST00000055458.4	7.78E-07	-0.668030726	12.1242
ENSMUSG00000047832	*Cdca4*	ENSMUST00000220899.1	0.00837557	-0.259724604	51.9946
ENSMUSG00000040420	*Cdh18*	ENSMUST00000164787.7	0.002534763	-1.613043072	2.08969
ENSMUSG00000028926	*Cdk14*	ENSMUST00000030763.12	0.000183795	-0.317443481	45.5039
ENSMUSG00000026023	*Cdk15*	ENSMUST00000114248.2	0.000284463	0.776005104	8.73007
ENSMUSG00000069089	*Cdk7*	ENSMUST00000091299.7	0.000153467	-0.383484543	38.8134
ENSMUSG00000023067	*Cdkn1a*	ENSMUST00000023829.6	0.007051726	-0.864648173	88.1013
ENSMUSG00000038069	*Cdkn2aip*	ENSMUST00000212175.1	0.007813011	0.542945184	5.57024
ENSMUSG00000033022	*Cdo1*	ENSMUST00000035804.7	1.06E-05	-0.68255541	98.4575
ENSMUSG00000030878	*Cdr2*	ENSMUST00000033169.8	9.93E-06	0.552968788	27.3811
ENSMUSG00000056216	*Cebpg*	ENSMUST00000130491.2	0.005536793	0.426893724	26.9438
ENSMUSG00000023031	*Cela1*	ENSMUST00000023775.7	0.000139076	1.005023979	15.2269
ENSMUSG00000052353	*Cemip*	ENSMUST00000064174.11	3.26E-05	0.420535066	12.2347
ENSMUSG00000060240	*Cend1*	ENSMUST00000124444.1	0.000217078	0.952224665	5.3486
ENSMUSG00000045328	*Cenpe*	ENSMUST00000062893.11	0.000191981	-0.323994596	19.2259
ENSMUSG00000031262	*Cenpi*	ENSMUST00000081064.11	0.000436173	-0.466409661	11.5895
ENSMUSG00000031756	*Cenpn*	ENSMUST00000034205.4	0.00012808	0.476506534	27.3819
ENSMUSG00000039523	*Cep104*	ENSMUST00000047497.14	0.000395879	-0.333442219	19.5712
ENSMUSG00000072825	*Cep170b*	ENSMUST00000220627.1	0.002810483	-0.378682832	11.6102
ENSMUSG00000038594	*Cep85l*	ENSMUST00000220443.1	0.004819759	0.420333603	3.1458
ENSMUSG00000027035	*Cers6*	ENSMUST00000028426.8	0.000431693	-0.320115522	16.9642
ENSMUSG00000031796	*Cfap20*	ENSMUST00000034249.7	1.45E-06	0.511193938	82.9371
ENSMUSG00000031631	*Cfap97*	ENSMUST00000034048.12	5.59E-07	0.603641774	17.8157
ENSMUSG00000031954	*Cfdp1*	ENSMUST00000034432.6	3.00E-09	0.52187318	241.124
ENSMUSG00000056201	*Cfl1*	ENSMUST00000116560.2	2.25E-06	-0.403146467	614.529
ENSMUSG00000032232	*Cgnl1*	ENSMUST00000072899.8	0.006790593	0.474860469	21.1137
ENSMUSG00000029161	*Cgref1*	ENSMUST00000031051.7	3.93E-05	0.683104645	17.8876
ENSMUSG00000002835	*Chaf1a*	ENSMUST00000002914.8	0.001354374	-0.306031761	63.1239
ENSMUSG00000057133	*Chd6*	ENSMUST00000039782.13	0.002150968	-0.286948643	8.87395
ENSMUSG00000052488	*Cherp*	ENSMUST00000212991.1	0.004712362	0.373074536	49.2968
ENSMUSG00000000743	*Chmp1a*	ENSMUST00000000759.8	1.51E-06	0.450403726	74.7812
ENSMUSG00000004843	*Chmp2b*	ENSMUST00000004965.6	0.005934147	0.247689061	105.434
ENSMUSG00000053119	*Chmp3*	ENSMUST00000059462.11	0.001314683	0.276663717	105.54
ENSMUSG00000027536	*Chmp4c*	ENSMUST00000029049.6	0.000122495	0.801904396	1.35538
ENSMUSG00000056486	*Chn1*	ENSMUST00000102677.10	0.003551298	-0.7151046	1.12195
ENSMUSG00000014077	*Chp1*	ENSMUST00000014221.12	0.00054583	-0.291592235	94.7504
ENSMUSG00000032997	*Chpf*	ENSMUST00000079205.13	0.000485084	-0.378552709	46.1283
ENSMUSG00000068391	*Chrac1*	ENSMUST00000089765.8	0.004121568	0.327550125	90.1796
ENSMUSG00000006958	*Chrd*	ENSMUST00000007171.12	9.65E-10	1.17595795	8.1851
ENSMUSG00000031283	*Chrdl1*	ENSMUST00000074660.11	2.04E-16	-1.236616742	10.418
ENSMUSG00000041189	*Chrnb1*	ENSMUST00000045971.8	0.000206615	1.370218161	2.60812
ENSMUSG00000034612	*Chst11*	ENSMUST00000040110.7	0.002756857	0.679662237	11.8468
ENSMUSG00000037347	*Chst7*	ENSMUST00000044138.7	4.27E-12	-2.061246434	1.67089
ENSMUSG00000058152	*Chsy3*	ENSMUST00000080721.4	3.91E-05	-2.076459673	0.15566
ENSMUSG00000046691	*Chtf8*	ENSMUST00000177068.7	8.74E-07	0.434871949	91.2177
ENSMUSG00000025199	*Chuk*	ENSMUST00000026217.10	0.00697189	-0.302964056	17.2164
ENSMUSG00000031781	*Ciapin1*	ENSMUST00000034233.14	1.03E-05	0.648310539	58.2433
ENSMUSG00000037725	*Ckap2*	ENSMUST00000046916.8	4.08E-05	0.381070513	57.4675
ENSMUSG00000048327	*Ckap2l*	ENSMUST00000052708.6	0.004988935	-0.243089354	56.8521
ENSMUSG00000040549	*Ckap5*	ENSMUST00000111338.9	0.000733651	-0.28299113	53.9327
ENSMUSG00000028044	*Cks1b*	ENSMUST00000029679.3	0.000823518	-0.31516194	173.765
ENSMUSG00000062248	*Cks2*	ENSMUST00000075853.5	0.009051721	-0.299299393	181.484

Table S3. RNAseq in D5 in mDPCs-CAS9 cells and sgCreb3l1 _A8_4: 1007 downregulated genes and 1027 upregulated genes

ENSMUSG00000002660	*Clpp*	ENSMUST00000002735.7	0.00071349	-0.394269752	41.4521
ENSMUSG00000041216	*Clvs1*	ENSMUST00000141734.1	0.000247168	-1.227671522	18.0776
ENSMUSG00000034390	*Cmip*	ENSMUST00000166750.8	6.46E-05	0.413482577	39.4629
ENSMUSG00000031875	*Cmtm3*	ENSMUST00000034343.4	0.002160913	0.270495545	158.503
ENSMUSG00000096188	*Cmtm4*	ENSMUST00000179802.1	8.19E-12	0.592670648	32.6945
ENSMUSG00000046441	*Cmtr2*	ENSMUST00000056972.5	0.000452143	0.519820083	7.52629
ENSMUSG00000030057	*Cnbp*	ENSMUST00000113619.7	0.007049791	0.228198127	295.358
ENSMUSG00000036810	*Cnep1r1*	ENSMUST00000095214.9	1.58E-05	0.456645821	52.0025
ENSMUSG00000036550	*Cnot1*	ENSMUST00000098473.10	1.36E-07	0.522011597	31.7429
ENSMUSG00000031601	*Cnot7*	ENSMUST00000034012.9	9.10E-13	0.717217029	49.3027
ENSMUSG00000036968	*Cnpy4*	ENSMUST00000110934.8	0.000655009	-0.307054141	74.6491
ENSMUSG00000017188	*Coa3*	ENSMUST00000017332.3	0.00154215	0.38653256	80.0896
ENSMUSG00000031979	*Cog2*	ENSMUST00000034460.10	1.52E-06	0.53459392	26.0545
ENSMUSG00000031753	*Cog4*	ENSMUST00000034203.16	2.74E-08	0.548732135	43.2958
ENSMUSG00000031916	*Cog8*	ENSMUST00000034391.3	0.00151499	0.470140974	11.1555
ENSMUSG00000027966	*Col11a1*	ENSMUST00000092155.11	0.0001204	-1.298737775	71.4991
ENSMUSG00000032332	*Col12a1*	ENSMUST00000071750.12	1.51E-06	0.608290198	388.763
ENSMUSG00000022371	*Col14a1*	ENSMUST00000110221.8	1.84E-06	1.861232921	7.44662
ENSMUSG00000001435	*Col18a1*	ENSMUST00000081654.12	0.003856482	0.504224979	15.7159
ENSMUSG00000016356	*Col20a1*	ENSMUST00000108856.7	0.005184778	1.178236926	4.70676
ENSMUSG00000031502	*Col4a1*	ENSMUST00000033898.9	0.000381534	-0.835731796	84.8911
ENSMUSG00000031273	*Col4a6*	ENSMUST00000101205.2	5.00E-15	-5.057725796	0.116674
ENSMUSG00000091345	*Col6a5*	ENSMUST00000165165.2	0.002877205	-2.812209344	0
ENSMUSG00000056174	*Col8a2*	ENSMUST00000070132.6	0.000326466	-2.632802893	6.86511
ENSMUSG00000034807	*Colgalt1*	ENSMUST00000047903.9	0.001621244	0.405072006	110.219
ENSMUSG00000055681	*Cope*	ENSMUST00000066469.13	5.68E-09	0.548708325	148.912
ENSMUSG00000030058	*Copg1*	ENSMUST00000113607.9	0.001685207	0.271756045	241.65
ENSMUSG00000031458	*Coprs*	ENSMUST00000033839.8	0.000229115	0.518161998	65.8431
ENSMUSG00000026798	*Coq4*	ENSMUST00000028137.9	0.003637891	-0.564282529	3.98645
ENSMUSG00000031782	*Coq9*	ENSMUST00000034234.14	0.000102095	0.445773455	44.693
ENSMUSG00000041729	*Coro2b*	ENSMUST00000048043.11	0.008144807	0.374874708	8.36249
ENSMUSG00000031818	*Cox4i1*	ENSMUST00000034276.12	9.57E-08	0.520242931	1041.12
ENSMUSG00000009876	*Cox4i2*	ENSMUST00000010020.11	0.006900581	1.144257581	14.7301
ENSMUSG00000051811	*Cox6b2*	ENSMUST00000063324.13	0.002782202	-0.929593502	21.0365
ENSMUSG00000031231	*Cox7b*	ENSMUST00000033582.4	0.004934642	0.248248902	230.353
ENSMUSG00000035885	*Cox8a*	ENSMUST00000039758.4	7.35E-05	-0.486423235	484.634
ENSMUSG00000037852	*Cpe*	ENSMUST00000048967.8	0.008680263	1.080086003	438.248
ENSMUSG00000025586	*Cpeb1*	ENSMUST00000130310.7	1.99E-07	0.738048023	13.3279
ENSMUSG00000062980	*Cped1*	ENSMUST00000115383.8	0.00016825	0.332998009	36.9414
ENSMUSG00000034361	*Cpne2*	ENSMUST00000048653.9	0.000480294	0.515057994	36.511
ENSMUSG00000022742	*Cpox*	ENSMUST00000060077.5	2.46E-06	0.487925923	27.4571
ENSMUSG00000065979	*Cpped1*	ENSMUST00000096272.10	0.002432863	-0.299177769	28.5907
ENSMUSG00000039007	*Cpq*	ENSMUST00000042167.9	4.57E-05	0.377948558	88.8348
ENSMUSG00000041781	*Cpsf2*	ENSMUST00000047357.9	0.001891067	-0.285829371	17.7608
ENSMUSG00000055531	*Cpsf6*	ENSMUST00000176686.7	2.41E-06	-0.588858226	19.3028
ENSMUSG00000078937	*Cpt1b*	ENSMUST00000109313.8	8.11E-05	0.780260989	4.65459
ENSMUSG00000027408	*Cpxm1*	ENSMUST00000028897.7	1.38E-08	-1.338784381	25.3375
ENSMUSG00000036596	*Cpz*	ENSMUST00000038676.6	4.92E-09	-2.721856356	0.251962
ENSMUSG00000032291	*Crabp1*	ENSMUST00000034830.8	4.83E-08	-1.484116419	137.275
ENSMUSG00000027230	*Creb3l1*	ENSMUST00000028663.4	1.96E-11	-1.361076117	22.4521
ENSMUSG00000053007	*Creb5*	ENSMUST00000205120.2	0.003889153	0.619011308	3.43492
ENSMUSG00000032652	*Crebl2*	ENSMUST00000046303.11	0.004557512	0.352833235	10.8112
ENSMUSG00000051451	*Crebzf*	ENSMUST00000061767.4	0.007515665	0.332384309	31.4624
ENSMUSG00000030284	*Creld1*	ENSMUST00000032422.5	0.002738843	0.412398795	29.9743
ENSMUSG00000006356	*Crip2*	ENSMUST00000084882.8	1.32E-12	-1.527647411	6.57086
ENSMUSG00000068742	*Cry2*	ENSMUST00000090559.11	0.000383167	-0.47888368	6.22677
ENSMUSG00000042109	*Csdc2*	ENSMUST00000038757.7	0.002599711	-0.752560038	2.16133
ENSMUSG00000014599	*Csf1*	ENSMUST00000014743.9	3.03E-26	-0.912883406	69.6327
ENSMUSG00000042042	*Csgalnact2*	ENSMUST00000049344.14	0.000180744	0.335733558	47.2128
ENSMUSG00000032312	*Csk*	ENSMUST00000217314.1	0.001438398	-0.384237728	26.4408
ENSMUSG00000032384	*Csnk1g1*	ENSMUST00000034949.9	0.000198348	-0.392974244	7.55509
ENSMUSG00000026421	*Csrp1*	ENSMUST00000027677.7	0.009006508	-0.258929671	302.373
ENSMUSG00000053536	*Cstf2t*	ENSMUST00000066039.6	0.003416545	-0.263282113	34.8238
ENSMUSG00000005698	*Ctcf*	ENSMUST00000005841.15	0.002227753	0.277920733	42.0483
ENSMUSG00000038816	*Ctnnal1*	ENSMUST00000045142.14	1.04E-06	-0.555288176	11.4925
ENSMUSG00000034101	*Ctnnd1*	ENSMUST00000111691.1	7.13E-05	-0.459616563	41.4404
ENSMUSG00000017760	*Ctsa*	ENSMUST00000103093.9	2.61E-07	-0.444051559	296.428
ENSMUSG00000083282	*Ctsf*	ENSMUST00000119694.1	9.12E-06	-0.886119577	24.5337
ENSMUSG00000016256	*Ctsz*	ENSMUST00000016400.8	4.11E-08	-0.45761876	308.116
ENSMUSG00000031446	*Cul4a*	ENSMUST00000016680.13	2.05E-12	0.609975447	79.3676
ENSMUSG00000032030	*Cul5*	ENSMUST00000034529.13	0.00308978	-0.334454285	12.9631
ENSMUSG00000038545	*Cul7*	ENSMUST00000043464.13	0.001512562	-0.369443546	39.1766

Table S3. RNAseq in D5 in mDPCs-CAS9 cells and sgCreb3l1 _A8_4: 1007 downregulated genes and 1027 upregulated genes

ENSMUSG00000029705	*Cux1*	ENSMUST00000176745.7	0.007299114	0.407194509	12.9722
ENSMUSG00000027014	*Cwc22*	ENSMUST00000144727.7	0.000467925	0.3319222	60.629
ENSMUSG00000025200	*Cwf19l1*	ENSMUST00000026218.5	0.007830478	-0.331468869	8.09349
ENSMUSG00000022865	*Cxadr*	ENSMUST00000023572.13	5.13E-11	0.860485379	7.85955
ENSMUSG00000021508	*Cxcl14*	ENSMUST00000021970.10	2.04E-11	2.21187226	12.4795
ENSMUSG00000029417	*Cxcl9*	ENSMUST00000113093.4	0.006376756	-1.359867087	0.165022
ENSMUSG00000031924	*Cyb5b*	ENSMUST00000034400.4	5.22E-12	0.648948904	80.1104
ENSMUSG00000006519	*Cyba*	ENSMUST00000017604.9	2.66E-16	1.257259582	82.38
ENSMUSG00000027015	*Cybrd1*	ENSMUST00000028403.2	0.000286138	-2.000329858	0.685634
ENSMUSG00000026170	*Cyp27a1*	ENSMUST00000027356.6	2.01E-05	1.25997183	4.35714
ENSMUSG00000015224	*Cyp2j9*	ENSMUST00000055693.8	2.78E-07	0.933071923	9.70983
ENSMUSG00000079057	*Cyp4v3*	ENSMUST00000095328.5	2.79E-05	1.082274144	5.87183
ENSMUSG00000039519	*Cyp7b1*	ENSMUST00000035625.6	0.005159405	1.761548925	0.918782
ENSMUSG00000041134	*Cyyr1*	ENSMUST00000114174.2	0.001303228	-1.361748384	0.079927
ENSMUSG00000031889	*D230025D16Rik*	ENSMUST00000034361.9	0.000307555	0.511768637	12.0681
ENSMUSG00000019362	*D8Ertd738e*	ENSMUST00000019506.8	0.000865035	0.455458918	410.374
ENSMUSG00000021559	*Dapk1*	ENSMUST00000077453.12	3.29E-06	-1.311104805	1.30662
ENSMUSG00000000346	*Dazap2*	ENSMUST00000000356.8	0.004396876	0.314948141	175.2
ENSMUSG00000000340	*Dbt*	ENSMUST00000000349.10	0.001140898	-0.449619296	5.34136
ENSMUSG00000028436	*Dcaf12*	ENSMUST00000030145.8	0.002474323	-0.273715232	37.1705
ENSMUSG00000037103	*Dcaf15*	ENSMUST00000210279.1	0.00013518	0.498067377	34.9245
ENSMUSG00000021222	*Dcaf4*	ENSMUST00000021645.8	7.87E-05	-0.530025545	12.5019
ENSMUSG00000049106	*Dcaf5*	ENSMUST00000054145.7	1.17E-05	-0.394151872	20.6498
ENSMUSG00000027797	*Dclk1*	ENSMUST00000167204.7	2.10E-08	-0.509379996	18.3621
ENSMUSG00000032500	*Dclk3*	ENSMUST00000111879.4	2.91E-05	-3.482613943	0.090861
ENSMUSG00000031562	*Dctd*	ENSMUST00000170263.8	1.92E-07	0.921834324	12.9292
ENSMUSG00000031516	*Dctn6*	ENSMUST00000033913.10	0.004864577	0.311442367	68.8118
ENSMUSG00000051674	*Dcun1d4*	ENSMUST00000063882.11	0.001444717	-0.334962751	17.4352
ENSMUSG00000061313	*Ddhd2*	ENSMUST00000033975.7	1.15E-09	0.668659874	18.6463
ENSMUSG00000030641	*Ddias*	ENSMUST00000032877.10	3.38E-05	-0.516898048	8.17125
ENSMUSG00000046818	*Ddit4l*	ENSMUST00000053855.7	5.99E-23	-1.420719971	4.63245
ENSMUSG00000015023	*Ddx19a*	ENSMUST00000040416.7	4.88E-07	0.560489811	70.5985
ENSMUSG00000033658	*Ddx19b*	ENSMUST00000040241.14	3.41E-08	0.945708375	3.69539
ENSMUSG00000027905	*Ddx20*	ENSMUST00000090680.10	0.001326914	-0.342852011	19.5685
ENSMUSG00000041645	*Ddx24*	ENSMUST00000044923.14	0.001252758	-0.283040147	68.4222
ENSMUSG00000045538	*Ddx28*	ENSMUST00000058579.6	0.000222074	0.641812123	8.11857
ENSMUSG00000069045	*Ddx3y*	ENSMUST00000091190.11	1.17E-11	0.601360841	56.6559
ENSMUSG00000070291	*Ddx43*	ENSMUST00000113367.1	0.001484419	1.346630835	2.35517
ENSMUSG00000057788	*Ddx49*	ENSMUST00000008004.9	0.005657932	0.333765085	37.6213
ENSMUSG00000040296	*Ddx58*	ENSMUST00000037907.12	0.004216797	-0.245891854	53.2428
ENSMUSG00000026404	*Ddx59*	ENSMUST00000027655.7	0.004440957	0.346639789	21.2784
ENSMUSG00000001482	*Def8*	ENSMUST00000065534.9	0.008085189	0.332258328	22.9897
ENSMUSG00000038456	*Dennd2a*	ENSMUST00000036877.9	0.004365102	0.402528538	46.106
ENSMUSG00000036661	*Dennd3*	ENSMUST00000043414.11	0.000183459	0.588571373	5.5316
ENSMUSG00000035901	*Dennd5a*	ENSMUST00000080437.12	0.000349008	0.325313207	74.1212
ENSMUSG00000022419	*Deptor*	ENSMUST00000096433.9	1.95E-10	0.826339221	19.8373
ENSMUSG00000022472	*Desi1*	ENSMUST00000152227.7	0.000362269	0.362028739	36.4658
ENSMUSG00000025357	*Dgka*	ENSMUST00000026414.8	3.37E-08	0.741091482	15.4248
ENSMUSG00000021185	*Dglucy*	ENSMUST00000069782.10	0.002422858	-0.718842687	6.36239
ENSMUSG00000014554	*Dguok*	ENSMUST00000014698.9	0.008914912	0.337118025	54.283
ENSMUSG00000027068	*Dhrs9*	ENSMUST00000063690.3	0.000943181	1.565072112	6.3543
ENSMUSG00000042426	*Dhx29*	ENSMUST00000038574.6	0.004916281	-0.354079781	19.0676
ENSMUSG00000030986	*Dhx32*	ENSMUST00000033290.11	0.003123464	0.387769221	18.8003
ENSMUSG00000037993	*Dhx38*	ENSMUST00000042601.8	8.39E-06	0.410860379	35.8952
ENSMUSG00000041415	*Dicer1*	ENSMUST00000222528.1	0.001095276	-0.472536337	4.46337
ENSMUSG00000007682	*Dio2*	ENSMUST00000082432.4	0.0037071	4.028927662	0.268459
ENSMUSG00000075707	*Dio3*	ENSMUST00000173014.1	0.002387323	-2.083769724	0.284232
ENSMUSG00000061689	*Dlgap4*	ENSMUST00000109566.8	0.000531882	-0.557011082	11.8332
ENSMUSG00000040856	*Dlk1*	ENSMUST00000109844.10	5.84E-06	-3.683254498	0.0670103
ENSMUSG00000045103	*Dmd*	ENSMUST00000113991.7	1.20E-05	2.214582657	2.05502
ENSMUSG00000030409	*Dmpk*	ENSMUST00000032568.13	4.35E-07	-0.789331843	7.59647
ENSMUSG00000055809	*Dnaaf3*	ENSMUST00000094897.4	0.005324644	1.044651403	1.31586
ENSMUSG00000031701	*Dnaja2*	ENSMUST00000034138.6	1.33E-11	0.574325903	117.53
ENSMUSG00000005483	*Dnajb1*	ENSMUST00000005620.9	0.006218759	0.365117949	46.1637
ENSMUSG00000074212	*Dnajb14*	ENSMUST00000090178.9	0.008532507	-0.281687375	5.29419
ENSMUSG00000026203	*Dnajb2*	ENSMUST00000188931.6	0.003134944	0.326415874	26.0821
ENSMUSG00000028035	*Dnajb4*	ENSMUST00000050073.12	0.008051569	-0.259714458	36.2329
ENSMUSG00000036052	*Dnajb5*	ENSMUST00000107973.2	1.09E-05	-0.860936757	5.85439
ENSMUSG00000044224	*Dnajc21*	ENSMUST00000136591.7	0.006022479	0.272668874	58.0779
ENSMUSG00000020657	*Dnajc27*	ENSMUST00000020986.14	0.003924063	0.386496409	7.99428
ENSMUSG00000022136	*Dnajc3*	ENSMUST00000022734.7	1.11E-05	-0.440349679	12.3235
ENSMUSG00000075467	*Dnlz*	ENSMUST00000028295.8	4.13E-05	-0.546605634	17.7662

ENSMUSG00000004099	*Dnmt1*	ENSMUST00000004202.16	0.001176422	-0.319066324	83.1117
ENSMUSG00000031093	*Dock11*	ENSMUST00000033419.12	0.00060038	0.473124797	20.4621
ENSMUSG00000027560	*Dok5*	ENSMUST00000029075.4	1.44E-05	-0.819038638	3.60823
ENSMUSG00000075419	*Dolk*	ENSMUST00000100219.4	0.000417811	-0.420248497	18.8189
ENSMUSG00000026856	*Dolpp1*	ENSMUST00000028209.14	5.53E-05	-0.840136961	5.02523
ENSMUSG00000024826	*Dpf2*	ENSMUST00000123029.7	0.000137429	-0.574011926	37.7145
ENSMUSG00000026958	*Dpp7*	ENSMUST00000028332.7	2.19E-07	-0.588492989	76.7729
ENSMUSG00000043671	*Dpy19l3*	ENSMUST00000051377.14	0.003626655	-0.29990946	7.31889
ENSMUSG00000033308	*Dpyd*	ENSMUST00000039177.11	3.78E-10	-2.423374783	0.498444
ENSMUSG00000009145	*Dqx1*	ENSMUST00000077502.4	4.44E-05	0.918565219	2.32075
ENSMUSG00000020457	*Drg1*	ENSMUST00000020741.11	0.005877125	0.251385665	109.417
ENSMUSG00000015932	*Dstn*	ENSMUST00000103172.3	0.004039562	-0.242255528	321.226
ENSMUSG00000024302	*Dtna*	ENSMUST00000115832.3	0.004377925	0.347155322	7.55678
ENSMUSG00000033268	*Duox1*	ENSMUST00000099461.3	5.86E-08	1.628373107	1.62187
ENSMUSG00000027224	*Duoxa1*	ENSMUST00000148417.7	1.81E-05	3.199220629	9.88346
ENSMUSG00000031901	*Dus2*	ENSMUST00000034375.10	0.003248457	1.222546507	5.79203
ENSMUSG00000007603	*Dus3l*	ENSMUST00000007747.8	0.000432353	-0.462038159	17.0912
ENSMUSG00000020648	*Dus4l*	ENSMUST00000020977.3	0.002068331	0.554672168	8.10309
ENSMUSG00000030002	*Dusp11*	ENSMUST00000032071.12	1.39E-06	0.496415242	45.7056
ENSMUSG00000018648	*Dusp14*	ENSMUST00000100705.10	0.005321248	0.584297633	17.0093
ENSMUSG00000030203	*Dusp16*	ENSMUST00000100857.9	0.000261278	0.404683577	13.7656
ENSMUSG00000003518	*Dusp3*	ENSMUST00000003612.12	0.0012162	0.371568807	14.8458
ENSMUSG00000037887	*Dusp8*	ENSMUST00000039926.9	0.002781518	1.068088041	4.14427
ENSMUSG00000018707	*Dync1h1*	ENSMUST00000018851.13	0.00033584	-0.299791207	26.7855
ENSMUSG00000027012	*Dync1i2*	ENSMUST00000112138.7	0.001748945	-0.284466915	71.6356
ENSMUSG00000035770	*Dync1li2*	ENSMUST00000041769.7	6.39E-05	0.356116335	45.8433
ENSMUSG00000092074	*Dynlt1a*	ENSMUST00000169415.1	0.001158182	-0.428129426	48.9909
ENSMUSG00000031176	*Dynlt3*	ENSMUST00000033519.2	0.001922059	0.321915013	119.835
ENSMUSG00000018983	*E2f2*	ENSMUST00000061721.5	0.001626633	0.607932109	2.36563
ENSMUSG00000014859	*E2f4*	ENSMUST00000015003.9	1.05E-09	0.659834764	45.6327
ENSMUSG00000037172	*E330009J07Rik*	ENSMUST00000101492.9	1.49E-07	0.825370438	3.77063
ENSMUSG00000057098	*Ebf1*	ENSMUST00000081265.11	0.000176052	-0.644107376	9.3616
ENSMUSG00000031168	*Ebp*	ENSMUST00000033509.14	0.002568091	0.524247648	83.0534
ENSMUSG00000057530	*Ece1*	ENSMUST00000102518.9	0.005509864	-0.231474937	69.2219
ENSMUSG00000034457	*Eda2r*	ENSMUST00000037353.9	7.54E-08	-1.219788832	3.96327
ENSMUSG00000036270	*Edc4*	ENSMUST00000040254.15	0.00054331	0.358244046	38.992
ENSMUSG00000030104	*Edem1*	ENSMUST00000089162.4	6.78E-05	0.348174216	47.4667
ENSMUSG00000034488	*Edil3*	ENSMUST00000081769.12	0.000156219	0.957186265	7.29029
ENSMUSG00000071644	*Eef1g*	ENSMUST00000052248.7	0.000220039	-0.311966728	141.227
ENSMUSG00000026495	*Efcab2*	ENSMUST00000027775.8	0.000988927	0.557853579	5.75269
ENSMUSG00000020467	*Efemp1*	ENSMUST00000020759.11	1.81E-10	1.602466517	109.079
ENSMUSG00000024909	*Efemp2*	ENSMUST00000165485.7	1.99E-07	-0.642724895	217.026
ENSMUSG00000028039	*Efna3*	ENSMUST00000029673.9	0.001460224	-0.719362259	1.98325
ENSMUSG00000048915	*Efna5*	ENSMUST00000076840.11	0.000416347	-1.155714648	10.3902
ENSMUSG00000000402	*Egfl6*	ENSMUST00000000412.2	2.00E-35	-1.977997557	64.7214
ENSMUSG00000031987	*Egln1*	ENSMUST00000034469.6	2.75E-23	0.923022311	57.7821
ENSMUSG00000037868	*Egr2*	ENSMUST00000048289.13	4.43E-11	-0.973978065	5.67282
ENSMUSG00000024937	*Ehbp1l1*	ENSMUST00000049295.14	7.32E-05	-0.36577092	19.2642
ENSMUSG00000024772	*Ehd1*	ENSMUST00000025684.3	0.001785568	-0.295492887	26.1517
ENSMUSG00000062762	*Ei24*	ENSMUST00000115086.12	4.02E-05	-0.392372415	47.772
ENSMUSG00000091337	*Eid1*	ENSMUST00000164756.3	0.009776548	-0.229404531	236.047
ENSMUSG00000024079	*Eif2ak2*	ENSMUST00000024884.4	0.003193299	-0.309226369	50.7723
ENSMUSG00000031668	*Eif2ak3*	ENSMUST00000034093.14	9.52E-05	0.375256055	30.1289
ENSMUSG00000021116	*Eif2s1*	ENSMUST00000071230.7	0.006348093	-0.236867131	51.2653
ENSMUSG00000069049	*Eif2s3y*	ENSMUST00000091197.3	4.57E-05	0.40781907	107.277
ENSMUSG00000028798	*Eif3i*	ENSMUST00000102593.10	0.006771753	-0.237802113	179.572
ENSMUSG00000093661	*Eif4e3*	ENSMUST00000032151.2	0.000104765	-0.538447415	7.4019
ENSMUSG00000020454	*Eif4enif1*	ENSMUST00000110049.7	0.008052695	0.289195201	29.5984
ENSMUSG00000028760	*Eif4g3*	ENSMUST00000203828.2	0.000943998	-0.679750123	2.39196
ENSMUSG00000008398	*Elk3*	ENSMUST00000008542.11	0.000692619	0.409408788	39.7017
ENSMUSG00000070002	*Ell*	ENSMUST00000093454.7	0.000252466	0.552936339	18.0856
ENSMUSG00000017670	*Elmo2*	ENSMUST00000094329.10	0.005769364	-0.253741768	51.9709
ENSMUSG00000035151	*Elmod2*	ENSMUST00000053902.3	2.74E-08	0.609556822	18.0353
ENSMUSG00000021696	*Elovl7*	ENSMUST00000022207.9	8.93E-09	1.734389099	2.62582
ENSMUSG00000021728	*Emb*	ENSMUST00000022242.8	0.002821123	-1.696245016	1.03047
ENSMUSG00000078517	*Emc1*	ENSMUST00000042096.14	1.07E-06	-0.480538464	16.5964
ENSMUSG00000029163	*Emilin1*	ENSMUST00000031055.7	0.00731853	0.22500261	98.3965
ENSMUSG00000071647	*Eml3*	ENSMUST00000096241.5	0.006282115	-0.484281064	18.1564
ENSMUSG00000026814	*Eng*	ENSMUST00000009705.13	0.001854864	-0.366536299	37.356
ENSMUSG00000013155	*Enkd1*	ENSMUST00000013299.10	0.000603959	0.635919424	33.5306
ENSMUSG00000022425	*Enpp2*	ENSMUST00000171545.7	0.001475662	1.824487928	32.5247
ENSMUSG00000015085	*Entpd2*	ENSMUST00000028328.2	0.003746657	-0.708784404	3.02204

Table S3. RNAseq in D5 in mDPCs-CAS9 cells and sgCreb3l1 _A8_4: 1007 downregulated genes and 1027 upregulated genes

ENSMUSG00000033068	*Entpd6*	ENSMUST00000094467.5	0.000227137	-0.58617803	4.71484
ENSMUSG00000035245	*Eogt*	ENSMUST00000054344.10	0.003633507	0.378785659	11.3766
ENSMUSG00000028906	*Epb41*	ENSMUST00000146021.7	0.009511226	-0.458615094	10.8384
ENSMUSG00000024376	*Epb41l4a*	ENSMUST00000025234.5	1.19E-16	0.873351237	25.8735
ENSMUSG00000028434	*Epb41l4b*	ENSMUST00000030142.3	0.006627282	-1.507839184	0.994934
ENSMUSG00000029859	*Epha1*	ENSMUST00000073387.4	3.55E-05	0.555433906	11.8302
ENSMUSG00000028289	*Epha7*	ENSMUST00000029964.11	7.58E-11	-0.628811303	13.1435
ENSMUSG00000010080	*Epn3*	ENSMUST00000142992.1	0.005664773	0.87842027	8.04178
ENSMUSG00000018166	*Erbb3*	ENSMUST00000082059.6	4.97E-05	1.480083465	0.633242
ENSMUSG00000021709	*Erbin*	ENSMUST00000091269.10	0.008012903	-0.269163168	38.1261
ENSMUSG00000005881	*Ergic3*	ENSMUST00000006035.12	0.000471301	-0.326810101	259.682
ENSMUSG00000031527	*Eri1*	ENSMUST00000033927.7	7.27E-13	0.646232428	39.9552
ENSMUSG00000051978	*Erich1*	ENSMUST00000110813.4	0.000605789	0.595234619	10.2404
ENSMUSG00000031483	*Erlin2*	ENSMUST00000033873.8	2.15E-06	0.471138061	21.153
ENSMUSG00000046324	*Ermp1*	ENSMUST00000054083.12	0.007416312	-0.339034906	8.34407
ENSMUSG00000020715	*Ern1*	ENSMUST00000001059.8	0.001430115	0.399060619	6.4007
ENSMUSG00000024955	*Esrra*	ENSMUST00000025906.10	0.002380139	-0.562860281	14.3742
ENSMUSG00000004610	*Etfb*	ENSMUST00000206196.1	0.0007863	-0.476954912	88.3742
ENSMUSG00000027809	*Etfdh*	ENSMUST00000029386.13	0.004633651	-0.268744274	39.6636
ENSMUSG00000022895	*Ets2*	ENSMUST00000023612.14	6.65E-10	0.534728106	68.1067
ENSMUSG00000013089	*Etv5*	ENSMUST00000079601.12	4.10E-05	-0.522551906	7.10374
ENSMUSG00000074030	*Exoc8*	ENSMUST00000098312.3	0.000982678	0.42070474	9.00602
ENSMUSG00000034321	*Exosc1*	ENSMUST00000075280.10	0.004915481	-0.380168343	16.145
ENSMUSG00000017264	*Exosc10*	ENSMUST00000017408.13	0.003111259	-0.268963122	66.5407
ENSMUSG00000021978	*Extl3*	ENSMUST00000022550.7	0.009672671	-0.246484427	14.6528
ENSMUSG00000052397	*Ezr*	ENSMUST00000064234.6	7.33E-05	0.731055605	30.8912
ENSMUSG00000062515	*Fabp4*	ENSMUST00000029041.5	0.007056781	2.754495846	22.4738
ENSMUSG00000027533	*Fabp5*	ENSMUST00000029046.8	7.31E-32	1.063600558	845.934
ENSMUSG00000024664	*Fads3*	ENSMUST00000115995.2	1.98E-09	-0.527087089	41.1434
ENSMUSG00000030630	*Fah*	ENSMUST00000032865.16	0.000739079	-0.440837592	20.607
ENSMUSG00000023011	*Faim2*	ENSMUST00000023750.7	1.88E-05	2.874166938	0.798199
ENSMUSG00000049687	*Fam109b*	ENSMUST00000050349.2	0.00074287	-0.500450502	6.4384
ENSMUSG00000035184	*Fam124a*	ENSMUST00000039064.7	6.28E-06	-1.156745088	0.797196
ENSMUSG00000026153	*Fam135a*	ENSMUST00000027337.14	0.000325742	0.489828815	8.7132
ENSMUSG00000035095	*Fam167a*	ENSMUST00000121288.1	3.74E-05	3.189350032	2.71594
ENSMUSG00000074071	*Fam169b*	ENSMUST00000210558.1	7.39E-10	-5.136603751	4.74E-08
ENSMUSG00000064138	*Fam172a*	ENSMUST00000091459.11	0.005030347	-0.272349972	23.4616
ENSMUSG00000095595	*Fam177a*	ENSMUST00000177768.2	0.003356146	0.276545225	33.8231
ENSMUSG00000031774	*Fam192a*	ENSMUST00000034226.7	1.07E-08	0.632609075	30.5435
ENSMUSG00000038827	*Fam206a*	ENSMUST00000045368.11	0.003411077	-0.397567032	8.53549
ENSMUSG00000033799	*Fam208b*	ENSMUST00000096069.4	0.00320622	-0.270701179	13.1815
ENSMUSG00000027495	*Fam210b*	ENSMUST00000028995.4	0.000331152	-0.672858723	4.20486
ENSMUSG00000048458	*Fam212b*	ENSMUST00000098273.2	0.000265521	-2.3588397	0.885367
ENSMUSG00000029059	*Fam213b*	ENSMUST00000030935.9	0.00088285	-0.661895881	65.4257
ENSMUSG00000003039	*Fam32a*	ENSMUST00000003123.9	1.87E-09	0.554424305	67.5919
ENSMUSG00000031399	*Fam3a*	ENSMUST00000114143.9	0.003340964	0.317403462	41.8105
ENSMUSG00000044468	*Fam46c*	ENSMUST00000061455.8	5.49E-07	0.843337411	5.48283
ENSMUSG00000047992	*Fam69c*	ENSMUST00000052501.7	0.001423026	-0.676683263	4.95102
ENSMUSG00000072568	*Fam84b*	ENSMUST00000100635.4	1.58E-08	1.42552128	2.10866
ENSMUSG00000031879	*Fam96b*	ENSMUST00000164884.8	9.27E-06	0.529313951	86.1624
ENSMUSG00000002017	*Fam98a*	ENSMUST00000112507.3	0.006215811	-0.269170215	26.3162
ENSMUSG00000000392	*Fap*	ENSMUST00000102732.9	1.19E-10	-1.279672542	6.85085
ENSMUSG00000025555	*Farp1*	ENSMUST00000026635.7	8.85E-06	-0.496190809	17.2483
ENSMUSG00000006369	*Fbln1*	ENSMUST00000109432.3	1.89E-08	-0.946643782	4.34467
ENSMUSG00000064080	*Fbln2*	ENSMUST00000113498.8	5.16E-18	0.801542385	471.441
ENSMUSG00000027204	*Fbn1*	ENSMUST00000028633.12	1.08E-06	-0.511171785	47.1608
ENSMUSG00000033313	*Fbxl8*	ENSMUST00000036221.11	0.008714919	0.528812146	6.51561
ENSMUSG00000038365	*Fbxo25*	ENSMUST00000043520.4	4.26E-06	0.512458707	33.6028
ENSMUSG00000027180	*Fbxo3*	ENSMUST00000028603.9	0.009293249	-0.251854353	17.148
ENSMUSG00000052934	*Fbxo31*	ENSMUST00000059018.13	0.000217919	0.72593022	10.1856
ENSMUSG00000021243	*Fcf1*	ENSMUST00000021669.14	0.000883691	-0.374245797	80.556
ENSMUSG00000070524	*Fcrlb*	ENSMUST00000094337.1	9.59E-05	1.727584304	2.62846
ENSMUSG00000043683	*Fem1a*	ENSMUST00000060253.3	0.003857516	-0.354216098	9.29785
ENSMUSG00000032118	*Fez1*	ENSMUST00000034630.14	0.001568707	0.818034843	6.16433
ENSMUSG00000037946	*Fgd3*	ENSMUST00000110087.8	0.00118007	-0.491355484	3.62468
ENSMUSG00000037225	*Fgf2*	ENSMUST00000200585.4	3.14E-05	-0.613642619	2.80727
ENSMUSG00000031565	*Fgfr1*	ENSMUST00000117179.8	2.47E-10	0.604317714	72.5528
ENSMUSG00000054252	*Fgfr3*	ENSMUST00000067150.13	0.00026722	-4.447561429	0.0249045
ENSMUSG00000008136	*Fhl2*	ENSMUST00000008280.13	0.000217829	-0.347806787	73.0862
ENSMUSG00000032643	*Fhl3*	ENSMUST00000038684.5	0.002148774	-0.315909793	61.9487
ENSMUSG00000028259	*Fhl5*	ENSMUST00000029922.13	0.0085036	-1.386776214	2.59515
ENSMUSG00000034295	*Fhod3*	ENSMUST00000037097.7	0.006724836	0.50988853	6.60011

Table S3. RNAseq in D5 in mDPCs-CAS9 cells and sgCreb3l1 _A8_4: 1007 downregulated genes and 1027 upregulated genes

ENSMUSG00000038417	*Fig4*	ENSMUST00000043814.4	0.000742267	0.319435308	38.0235
ENSMUSG00000035455	*Fignl1*	ENSMUST00000047689.10	0.001035616	0.420446451	31.6275
ENSMUSG00000019054	*Fis1*	ENSMUST00000019198.6	0.004377182	-0.281931535	166.57
ENSMUSG00000019428	*Fkbp8*	ENSMUST00000075491.13	0.000132458	0.472501038	157.839
ENSMUSG00000029781	*Fkbp9*	ENSMUST00000031795.7	0.008916724	-0.474168094	521.443
ENSMUSG00000041559	*Fmod*	ENSMUST00000048183.7	0.000389205	-1.945507399	34.3742
ENSMUSG00000074738	*Fndc10*	ENSMUST00000099265.2	0.000279982	-1.249062669	8.25146
ENSMUSG00000033737	*Fndc3c1*	ENSMUST00000039447.13	7.34E-09	-0.947087008	1.83771
ENSMUSG00000015994	*Fnta*	ENSMUST00000016138.10	0.006559007	0.429192047	63.6669
ENSMUSG00000029135	*Fosl2*	ENSMUST00000031017.10	1.20E-08	0.482202147	48.6051
ENSMUSG00000042812	*Foxf1*	ENSMUST00000181504.1	0.000353075	0.464849077	51.0591
ENSMUSG00000033713	*Foxn3*	ENSMUST00000046859.11	0.003783152	-0.311878934	12.2709
ENSMUSG00000042903	*Foxo4*	ENSMUST00000062000.5	0.001475395	0.303197954	38.1719
ENSMUSG00000030067	*Foxp1*	ENSMUST00000113322.8	0.000547118	0.33133347	30.8969
ENSMUSG00000038415	*Foxq1*	ENSMUST00000042118.10	8.82E-09	1.30925456	7.57994
ENSMUSG00000074676	*Foxs1*	ENSMUST00000099200.2	4.51E-09	-1.321955397	3.91955
ENSMUSG00000031590	*Frg1*	ENSMUST00000033999.7	3.16E-08	0.587877095	104.157
ENSMUSG00000019779	*Frk*	ENSMUST00000019913.14	2.83E-14	0.915129912	17.9968
ENSMUSG00000024816	*Frmd8*	ENSMUST00000025728.12	0.002677871	-0.289309938	21.0012
ENSMUSG00000022816	*Fstl1*	ENSMUST00000114763.2	0.001099948	-0.351773483	653.618
ENSMUSG00000055932	*Fto*	ENSMUST00000069718.14	0.000133682	0.527256744	93.3783
ENSMUSG00000028034	*Fubp1*	ENSMUST00000196695.4	6.09E-07	0.587376802	40.8081
ENSMUSG00000033703	*Fuk*	ENSMUST00000041382.6	0.003566993	0.496237244	13.6647
ENSMUSG00000025040	*Fundc1*	ENSMUST00000026016.12	0.005921451	0.275165535	54.6134
ENSMUSG00000031198	*Fundc2*	ENSMUST00000033541.4	7.37E-06	0.404560401	65.5531
ENSMUSG00000025466	*Fuom*	ENSMUST00000026539.13	0.001031509	0.705117469	18.748
ENSMUSG00000046152	*Fut10*	ENSMUST00000066173.11	6.24E-05	0.539811946	14.7321
ENSMUSG00000070583	*Fv1*	ENSMUST00000094481.1	0.000416546	-0.524809965	12.5964
ENSMUSG00000022148	*Fyb*	ENSMUST00000162154.1	0.005401238	-3.292843251	4.92614
ENSMUSG00000078612	*Fyb2*	ENSMUST00000106804.1	0.009607005	0.867787273	2.33863
ENSMUSG00000019843	*Fyn*	ENSMUST00000099967.9	0.002165797	0.406291339	40.8042
ENSMUSG00000049791	*Fzd4*	ENSMUST00000058755.4	0.003207999	-0.558991469	9.21678
ENSMUSG00000074203	*G430095P16Rik*	ENSMUST00000098571.4	1.39E-06	1.179631518	19.9687
ENSMUSG00000031950	*Gabarapl2*	ENSMUST00000034428.7	3.20E-13	0.787616127	65.3353
ENSMUSG00000031340	*Gabre*	ENSMUST00000064780.3	0.001275321	0.503724366	5.52071
ENSMUSG00000036390	*Gadd45a*	ENSMUST00000043098.8	1.85E-20	0.923419441	139.934
ENSMUSG00000021453	*Gadd45g*	ENSMUST00000021903.2	0.003568869	-0.354423708	33.1989
ENSMUSG00000035473	*Galm*	ENSMUST00000039205.10	8.15E-05	-0.398175559	32.6404
ENSMUSG00000015027	*Galns*	ENSMUST00000015171.10	7.20E-09	0.632854653	35.3863
ENSMUSG00000060988	*Galnt13*	ENSMUST00000068595.5	0.006536992	1.771133227	1.14151
ENSMUSG00000031608	*Galnt7*	ENSMUST00000034021.11	0.001819759	0.757280764	4.05743
ENSMUSG00000052557	*Gan*	ENSMUST00000162997.2	0.000305247	0.465517089	2.73557
ENSMUSG00000071650	*Ganab*	ENSMUST00000096246.3	0.001548901	-0.267800753	154.229
ENSMUSG00000047261	*Gap43*	ENSMUST00000102817.4	0.000115467	2.02741054	19.6794
ENSMUSG00000029777	*Gars*	ENSMUST00000003572.9	0.000505139	0.428908578	387.375
ENSMUSG00000040220	*Gas8*	ENSMUST00000093043.6	0.000487054	0.419481337	36.1147
ENSMUSG00000036180	*Gatad2a*	ENSMUST00000116463.3	8.23E-08	0.511954978	54.211
ENSMUSG00000015944	*Gatsl2*	ENSMUST00000016088.8	5.99E-05	0.39482148	13.1951
ENSMUSG00000028048	*Gba*	ENSMUST00000077367.10	1.04E-07	-0.445889702	164.461
ENSMUSG00000022707	*Gbe1*	ENSMUST00000163832.7	0.000905565	0.507369875	12.6065
ENSMUSG00000025224	*Gbf1*	ENSMUST00000026254.13	0.002001664	-0.31390928	19.4102
ENSMUSG00000029298	*Gbp9*	ENSMUST00000031238.12	0.000123164	1.750629663	8.18411
ENSMUSG00000026893	*Gca*	ENSMUST00000028257.2	4.84E-05	0.984871282	2.00026
ENSMUSG00000029708	*Gcc1*	ENSMUST00000090511.3	0.005168727	0.306522471	22.4174
ENSMUSG00000021218	*Gdi2*	ENSMUST00000059515.7	0.001196823	-0.270985812	182.466
ENSMUSG00000061666	*Gdpd1*	ENSMUST00000020804.7	2.47E-09	0.890754334	12.0007
ENSMUSG00000028214	*Gem*	ENSMUST00000108304.8	1.26E-05	0.546122812	25.8144
ENSMUSG00000029992	*Gfpt1*	ENSMUST00000113658.7	0.001434473	0.267000578	79.1762
ENSMUSG00000020363	*Gfpt2*	ENSMUST00000020629.4	1.20E-06	-0.85041334	2.25901
ENSMUSG00000041028	*Ghitm*	ENSMUST00000223921.1	0.003555778	-0.313629581	134.918
ENSMUSG00000047867	*Gimap6*	ENSMUST00000053661.6	0.003734278	1.680828819	1.93667
ENSMUSG00000051124	*Gimap9*	ENSMUST00000054050.4	0.002664224	0.457087771	29.0706
ENSMUSG00000031821	*Gins2*	ENSMUST00000155609.1	0.001446761	0.581723987	68.7253
ENSMUSG00000031669	*Gins3*	ENSMUST00000034094.10	3.13E-05	0.54659257	18.9183
ENSMUSG00000031546	*Gins4*	ENSMUST00000033950.6	1.12E-05	0.441516374	88.4146
ENSMUSG00000042357	*Gjb5*	ENSMUST00000046498.2	0.00552135	-1.359386245	0.57251
ENSMUSG00000021552	*Gkap1*	ENSMUST00000091579.5	8.68E-05	0.489567394	33.367
ENSMUSG00000045594	*Glb1*	ENSMUST00000063042.10	0.00751774	-0.257181903	42.8974
ENSMUSG00000003316	*Glg1*	ENSMUST00000169020.7	1.23E-12	0.599624284	100.679
ENSMUSG00000021318	*Gli3*	ENSMUST00000110510.3	1.51E-10	-0.56344982	15.3213
ENSMUSG00000028480	*Glipr2*	ENSMUST00000030202.13	7.08E-05	-0.344906509	77.7045
ENSMUSG00000024026	*Glo1*	ENSMUST00000024823.12	0.005642756	-0.236766865	117.111

ENSMUSG00000038257	*Glra3*	ENSMUST00000000275.8	2.52E-06	-0.648252506	2.55825
ENSMUSG00000011884	*Gltp*	ENSMUST00000012028.13	0.008412443	0.309684653	109.799
ENSMUSG00000110444	*Gm10033*	ENSMUST00000213012.1	0.004397258	0.543489039	2.07536
ENSMUSG00000061062	*Gm10093*	ENSMUST00000079363.4	0.006137935	-0.309224297	24.6613
ENSMUSG00000066878	*Gm10184*	ENSMUST00000086423.4	1.57E-07	-0.449345794	123.049
ENSMUSG00000068141	*Gm10232*	ENSMUST00000089221.2	0.000988565	-0.60693807	19.4858
ENSMUSG00000069117	*Gm10260*	ENSMUST00000074072.4	0.00278888	-0.249889425	935.004
ENSMUSG00000070713	*Gm10282*	ENSMUST00000075602.7	0.002064941	-0.27782707	148.768
ENSMUSG00000073647	*Gm10557*	ENSMUST00000097690.3	0.006351941	0.561546195	17.7577
ENSMUSG00000068240	*Gm11808*	ENSMUST00000089430.5	9.60E-09	0.479102917	2590.74
ENSMUSG00000082292	*Gm12250*	ENSMUST00000119467.1	0.000152429	-2.01722659	0.248894
ENSMUSG00000082082	*Gm13230*	ENSMUST00000120870.1	1.62E-18	1.745826374	13.5043
ENSMUSG00000095101	*Gm13285*	ENSMUST00000179725.2	0.007290882	-0.434382351	10.7231
ENSMUSG00000079008	*Gm14124*	ENSMUST00000109922.1	5.99E-06	1.173964244	2.2306
ENSMUSG00000079009	*Gm14139*	ENSMUST00000109926.7	0.000130525	2.968426618	1.99411
ENSMUSG00000078877	*Gm14295*	ENSMUST00000118012.8	0.001340829	-0.527764366	17.4796
ENSMUSG00000095362	*Gm14325*	ENSMUST00000150650.1	0.005468523	-0.630799209	48.7858
ENSMUSG00000078862	*Gm14326*	ENSMUST00000108928.1	0.005805799	-0.546203229	12.5075
ENSMUSG00000074521	*Gm14327*	ENSMUST00000108935.7	0.00534526	-0.730875647	1.83047
ENSMUSG00000090093	*Gm14399*	ENSMUST00000109059.7	0.001953535	-1.570819634	1.40533
ENSMUSG00000078870	*Gm14410*	ENSMUST00000108964.1	0.000614904	-0.611464154	10.5116
ENSMUSG00000078881	*Gm14434*	ENSMUST00000120959.7	0.000856395	-0.513229943	17.3501
ENSMUSG00000082630	*Gm14834*	ENSMUST00000119416.1	2.12E-06	1.04162235	22.0698
ENSMUSG00000093954	*Gm16867*	ENSMUST00000179116.2	0.009140449	-1.908858917	0.030151
ENSMUSG00000107810	*Gm18609*	ENSMUST00000203370.1	0.001662697	0.960266149	1.36695
ENSMUSG00000107758	*Gm19253*	ENSMUST00000204279.1	0.002482787	1.764263329	27.2612
ENSMUSG00000097187	*Gm19426*	ENSMUST00000181711.1	4.88E-05	-0.788076238	14.5785
ENSMUSG00000041716	*Gm20604*	ENSMUST00000174651.1	1.06E-08	-0.774292846	8.16028
ENSMUSG00000075359	*Gm20775*	ENSMUST00000100115.2	0.009922679	-0.818445767	1.32596
ENSMUSG00000096850	*Gm21748*	ENSMUST00000179623.1	0.000266553	1.145337193	61.1301
ENSMUSG00000095366	*Gm21860*	ENSMUST00000177893.1	0.000266553	1.145337193	61.1301
ENSMUSG00000094942	*Gm3604*	ENSMUST00000187656.6	0.00199982	-0.78164793	1.21358
ENSMUSG00000067017	*Gm3608*	ENSMUST00000086725.5	6.93E-09	-0.626037157	21.7689
ENSMUSG00000107383	*Gm4366*	ENSMUST00000201488.1	1.07E-08	-0.547795335	75.5679
ENSMUSG00000107770	*Gm44126*	ENSMUST00000204831.1	0.007408951	0.480061496	66.2309
ENSMUSG00000112808	*Gm4739*	ENSMUST00000218296.1	6.17E-13	0.704071527	358.964
ENSMUSG00000095597	*Gm6472*	ENSMUST00000187973.1	0.002609426	0.254770224	907.641
ENSMUSG00000113473	*Gm7045*	ENSMUST00000221466.1	0.007089501	0.823044413	11.1163
ENSMUSG00000090667	*Gm765*	ENSMUST00000164491.2	0.00067854	-3.052483513	0
ENSMUSG00000089901	*Gm8113*	ENSMUST00000160298.1	9.01E-08	-0.62356411	12.5769
ENSMUSG00000073234	*Gm8773*	ENSMUST00000101627.4	0.0002784	0.896469677	5.92622
ENSMUSG00000056133	*Gm9992*	ENSMUST00000070059.3	2.55E-05	2.815129699	1.68238
ENSMUSG00000024639	*Gnaq*	ENSMUST00000025541.5	0.000398553	-0.329497146	17.6344
ENSMUSG00000024429	*Gnl1*	ENSMUST00000087200.3	0.009459506	-0.224067673	72.1079
ENSMUSG00000031985	*Gnpat*	ENSMUST00000034466.9	0.001090542	0.354029597	25.2465
ENSMUSG00000035521	*Gnptg*	ENSMUST00000038973.6	0.001483714	-0.376890972	39.4713
ENSMUSG00000015341	*Golga7*	ENSMUST00000051094.8	1.21E-05	0.4708228	58.6793
ENSMUSG00000021556	*Golm1*	ENSMUST00000022039.6	1.22E-05	-0.623399959	19.7924
ENSMUSG00000031672	*Got2*	ENSMUST00000034097.7	1.55E-08	0.487121296	117.361
ENSMUSG00000080935	*Got2-ps1*	ENSMUST00000121655.1	0.005168166	0.967661222	3.55311
ENSMUSG00000031545	*Gpat4*	ENSMUST00000167004.2	3.76E-08	0.494461245	39.8347
ENSMUSG00000031119	*Gpc4*	ENSMUST00000033450.2	0.00322414	-0.441741878	153.358
ENSMUSG00000058571	*Gpc6*	ENSMUST00000078849.10	4.62E-06	-1.040525396	4.68461
ENSMUSG00000026827	*Gpd2*	ENSMUST00000028167.2	0.00203122	0.337413175	15.0587
ENSMUSG00000028848	*Gpn2*	ENSMUST00000030661.13	0.008331064	-0.407605844	18.0961
ENSMUSG00000029816	*Gpnmb*	ENSMUST00000031840.9	1.47E-10	0.734224612	457.116
ENSMUSG00000053101	*Gpr141*	ENSMUST00000065335.2	0.00662124	1.701775924	1.52849
ENSMUSG00000046733	*Gprc5a*	ENSMUST00000050104.7	4.38E-05	0.588548564	25.2618
ENSMUSG00000045441	*Gprin3*	ENSMUST00000051065.5	2.12E-22	-1.160153158	2.91956
ENSMUSG00000026930	*Gpsm1*	ENSMUST00000066936.8	0.00660517	-0.489379241	25.6976
ENSMUSG00000031700	*Gpt2*	ENSMUST00000034136.11	1.27E-08	1.064312099	50.7192
ENSMUSG00000021760	*Gpx8*	ENSMUST00000022282.4	2.15E-06	-0.415904236	176.702
ENSMUSG00000020176	*Grb10*	ENSMUST00000093321.11	3.75E-05	1.164324421	129.524
ENSMUSG00000042942	*Greb1l*	ENSMUST00000048977.14	0.009692038	0.444589358	1.94174
ENSMUSG00000074934	*Grem1*	ENSMUST00000099575.3	0.00030268	0.45122175	712.201
ENSMUSG00000001986	*Gria3*	ENSMUST00000148212.1	0.00936473	-0.693949205	4.12964
ENSMUSG00000022564	*Grina*	ENSMUST00000023225.6	0.003649	0.242910184	386.972
ENSMUSG00000003228	*Grk5*	ENSMUST00000003313.8	4.48E-07	-0.530937595	15.4483
ENSMUSG00000034708	*Grn*	ENSMUST00000049460.10	3.90E-05	-0.405462023	519.947
ENSMUSG00000039934	*Gsap*	ENSMUST00000036031.12	0.007724675	0.473795665	4.49099
ENSMUSG00000022575	*Gsdmd*	ENSMUST00000023238.4	0.000376214	0.485564284	73.9533
ENSMUSG00000044715	*Gskip*	ENSMUST00000051934.6	0.004099468	-0.349332936	11.4408

ENSMUSG00000026879	*Gsn*	ENSMUST00000201185.3	1.28E-07	-0.448997639	147.773
ENSMUSG00000071723	*Gspt2*	ENSMUST00000096368.3	4.07E-07	1.895374021	2.8595
ENSMUSG00000031584	*Gsr*	ENSMUST00000033992.8	1.15E-05	0.469812676	32.0597
ENSMUSG00000027610	*Gss*	ENSMUST00000079691.12	0.000296987	-0.389553925	32.3437
ENSMUSG00000029864	*Gstk1*	ENSMUST00000031897.7	0.005825682	1.121193903	15.2071
ENSMUSG00000068762	*Gstm6*	ENSMUST00000106685.8	0.003284106	-1.622141667	0.591973
ENSMUSG00000004035	*Gstm7*	ENSMUST00000004137.10	0.003240291	-0.514625565	8.11983
ENSMUSG00000025068	*Gsto1*	ENSMUST00000026050.7	1.10E-06	-0.484074957	72.3705
ENSMUSG00000031585	*Gtf2e2*	ENSMUST00000167264.7	1.03E-07	0.607034633	54.7862
ENSMUSG00000002658	*Gtf2f1*	ENSMUST00000002733.6	0.000118753	-0.347599494	77.1019
ENSMUSG00000026816	*Gtf3c5*	ENSMUST00000028157.8	0.001389742	-0.342604949	25.116
ENSMUSG00000022385	*Gtse1*	ENSMUST00000170629.1	0.000341179	-0.42170811	29.2448
ENSMUSG00000023982	*Guca1a*	ENSMUST00000059348.7	0.009155686	0.92102392	4.46098
ENSMUSG00000030074	*Gxylt2*	ENSMUST00000032157.8	7.85E-10	-0.516351234	31.6874
ENSMUSG00000079547	*H2-DMb1*	ENSMUST00000114232.3	0.006929574	0.949424998	6.09051
ENSMUSG00000060032	*H2afj*	ENSMUST00000203982.1	0.007252446	-0.681778799	31.2672
ENSMUSG00000033629	*Hacd3*	ENSMUST00000036615.6	0.009046096	-0.27718562	44.6643
ENSMUSG00000028497	*Hacd4*	ENSMUST00000151280.7	0.000143084	-0.336374949	31.6778
ENSMUSG00000024158	*Hagh*	ENSMUST00000118788.7	0.008565789	0.375919384	27.8437
ENSMUSG00000007594	*Hapln4*	ENSMUST00000007738.10	7.60E-09	1.10852892	3.65153
ENSMUSG00000022367	*Has2*	ENSMUST00000050544.7	0.006533624	-0.464433696	9.61654
ENSMUSG00000027018	*Hat1*	ENSMUST00000028408.2	0.001620646	-0.274483645	115.817
ENSMUSG00000024486	*Hbegf*	ENSMUST00000025363.5	0.001292503	0.540810562	8.63452
ENSMUSG00000028800	*Hdac1*	ENSMUST00000102597.4	0.000605034	-0.292150903	113.556
ENSMUSG00000002833	*Hdgfl2*	ENSMUST00000226053.1	0.000584058	-0.482540776	44.4029
ENSMUSG00000025104	*Hdgfl3*	ENSMUST00000107305.7	0.009630097	0.358841906	32.1349
ENSMUSG00000050244	*Heatr1*	ENSMUST00000059270.9	0.002857979	-0.305241346	8.61088
ENSMUSG00000031657	*Heatr3*	ENSMUST00000034079.13	1.64E-07	0.506875435	46.999
ENSMUSG00000035181	*Heatr5a*	ENSMUST00000040583.6	0.006431024	0.231302777	36.7652
ENSMUSG00000039414	*Heatr5b*	ENSMUST00000097281.2	6.90E-05	-0.365837273	15.7142
ENSMUSG00000019853	*Hebp2*	ENSMUST00000020000.6	0.009492434	0.421004911	7.63264
ENSMUSG00000041180	*Hectd2*	ENSMUST00000047247.11	0.000311292	-0.723889061	1.69581
ENSMUSG00000046861	*Hectd3*	ENSMUST00000050067.9	0.001122074	-0.332765765	14.8387
ENSMUSG00000031209	*Heph*	ENSMUST00000113838.7	4.42E-07	3.833286505	0.933907
ENSMUSG00000029804	*Herc3*	ENSMUST00000041401.10	0.00373015	0.32498953	13.4305
ENSMUSG00000031770	*Herpud1*	ENSMUST00000161576.7	1.68E-05	0.830236129	64.3794
ENSMUSG00000022528	*Hes1*	ENSMUST00000023171.7	3.53E-10	-1.33119016	2.51461
ENSMUSG00000039307	*Hexdc*	ENSMUST00000124925.7	0.001433748	0.433564677	9.12443
ENSMUSG00000048878	*Hexim1*	ENSMUST00000053063.6	0.003093761	-0.28179637	27.8806
ENSMUSG00000037260	*Hgsnat*	ENSMUST00000037609.7	6.73E-13	0.775445882	36.1356
ENSMUSG00000036450	*Hif1an*	ENSMUST00000040455.4	0.005417234	-0.276369562	11.3286
ENSMUSG00000025868	*Higd2a*	ENSMUST00000026986.6	0.001567945	-0.341079231	127.311
ENSMUSG00000078851	*Hist3h2a*	ENSMUST00000108817.4	0.000357245	-0.507769626	13.9687
ENSMUSG00000002428	*Hltf*	ENSMUST00000002502.11	0.001536421	0.374499785	12.1691
ENSMUSG00000032126	*Hmbs*	ENSMUST00000077353.14	0.007527984	-0.316210152	25.5356
ENSMUSG00000030060	*Hmces*	ENSMUST00000032141.13	0.002986724	0.321076748	55.8508
ENSMUSG00000056758	*Hmga2*	ENSMUST00000072777.13	0.002388525	-0.438690049	24.5914
ENSMUSG00000054717	*Hmgb2*	ENSMUST00000067925.7	1.39E-13	0.619188674	200.766
ENSMUSG00000028672	*Hmgcl*	ENSMUST00000030432.7	6.76E-05	-0.411212117	51.2793
ENSMUSG00000003038	*Hmgn2*	ENSMUST00000102553.10	0.00034542	-0.325850495	189.863
ENSMUSG00000034518	*Hmgxb4*	ENSMUST00000145919.1	1.85E-07	0.683809476	16.1781
ENSMUSG00000005413	*Hmox1*	ENSMUST00000005548.7	0.002907861	0.406353775	168.921
ENSMUSG00000046434	*Hnrnpa1*	ENSMUST00000087351.7	0.009667306	-0.22379676	486.512
ENSMUSG00000052566	*Hook2*	ENSMUST00000064495.7	7.79E-05	0.64064032	10.6038
ENSMUSG00000037234	*Hook3*	ENSMUST00000037182.13	4.04E-05	0.424510503	13.302
ENSMUSG00000059325	*Hopx*	ENSMUST00000113453.8	1.22E-12	1.895014931	17.6462
ENSMUSG00000000938	*Hoxa10*	ENSMUST00000126402.1	0.002155777	1.662580086	1.88476
ENSMUSG00000079560	*Hoxa3*	ENSMUST00000114434.8	7.78E-07	0.940873058	6.723
ENSMUSG00000000942	*Hoxa4*	ENSMUST00000101395.2	0.004242504	0.54359533	8.60075
ENSMUSG00000043219	*Hoxa6*	ENSMUST00000062829.8	0.002033106	1.058757665	6.24226
ENSMUSG00000038236	*Hoxa7*	ENSMUST00000048715.8	1.66E-09	1.332513451	24.2839
ENSMUSG00000038227	*Hoxa9*	ENSMUST00000114425.2	0.007492863	1.763176252	5.95856
ENSMUSG00000075588	*Hoxb2*	ENSMUST00000100523.6	2.43E-06	1.892383344	4.52528
ENSMUSG00000048763	*Hoxb3*	ENSMUST00000093944.9	7.88E-17	1.836818127	5.77094
ENSMUSG00000038692	*Hoxb4*	ENSMUST00000049241.8	1.06E-08	2.521854463	3.03241
ENSMUSG00000038005	*Hpf1*	ENSMUST00000037190.14	8.97E-13	0.79899839	83.0613
ENSMUSG00000025630	*Hprt*	ENSMUST00000026723.8	0.000118119	0.363843293	134.859
ENSMUSG00000074811	*Hps6*	ENSMUST00000099393.2	0.00719281	-0.425939007	7.48074
ENSMUSG00000031839	*Hsbp1*	ENSMUST00000034300.7	0.000777362	0.300245787	206.924
ENSMUSG00000029311	*Hsd17b11*	ENSMUST00000031251.15	0.007271298	0.382732352	115.97
ENSMUSG00000042289	*Hsd3b7*	ENSMUST00000046863.11	0.003934439	0.456002204	15.7935
ENSMUSG00000034189	*Hsdl1*	ENSMUST00000036049.5	1.05E-12	0.809845173	25.2695

Table S3. RNAseq in D5 in mDPCs-CAS9 cells and sgCreb3l1 _A8_4: 1007 downregulated genes and 1027 upregulated genes

ENSMUSG00000028383	*Hsdl2*	ENSMUST00000030078.11	5.62E-07	-0.473395234	36.1818
ENSMUSG00000059970	*Hspa2*	ENSMUST00000219555.1	0.001384341	-0.366575201	32.5335
ENSMUSG00000039745	*Htatip2*	ENSMUST00000207895.1	0.000992471	0.541756198	21.2922
ENSMUSG00000068329	*Htra2*	ENSMUST00000089645.12	0.000339901	0.336507142	94.5402
ENSMUSG00000064267	*Hvcn1*	ENSMUST00000072602.13	0.000324621	0.448878318	16.805
ENSMUSG00000062054	*Iah1*	ENSMUST00000076813.7	0.002570899	-0.434141867	26.2818
ENSMUSG00000037405	*Icam1*	ENSMUST00000086399.5	0.002687372	-1.916758716	0.329365
ENSMUSG00000032174	*Icam5*	ENSMUST00000019616.5	0.006925885	-0.563675287	3.28021
ENSMUSG00000050002	*Idnk*	ENSMUST00000109868.3	0.007776063	-0.484940677	6.14072
ENSMUSG00000079017	*Ifi27l2a*	ENSMUST00000055071.8	1.03E-06	-0.407850898	1744.22
ENSMUSG00000074896	*Ifit3*	ENSMUST00000102825.3	0.00116326	-1.219193665	31.2691
ENSMUSG00000062488	*Ifit3b*	ENSMUST00000076249.5	0.003807104	-0.97191793	12.2697
ENSMUSG00000036256	*Igfbp7*	ENSMUST00000046746.9	0.000725412	-0.548833097	139.728
ENSMUSG00000036334	*Igsf10*	ENSMUST00000194546.5	3.08E-06	0.422892663	137.313
ENSMUSG00000028431	*Ikbkap*	ENSMUST00000030140.2	0.00214598	-0.284811959	16.3018
ENSMUSG00000031537	*Ikbkb*	ENSMUST00000033939.12	5.21E-06	0.551993162	13.9199
ENSMUSG00000026069	*Il1rl1*	ENSMUST00000173514.7	0.002693492	1.729024634	54.1777
ENSMUSG00000068758	*Il3ra*	ENSMUST00000090591.3	0.003070309	0.587020315	14.9523
ENSMUSG00000040612	*Ildr2*	ENSMUST00000111416.6	0.00369439	0.61945027	1.06577
ENSMUSG00000066324	*Impad1*	ENSMUST00000084949.2	0.009782699	-0.216861809	76.6876
ENSMUSG00000003500	*Impdh1*	ENSMUST00000078155.11	0.005564536	0.310925327	50.7239
ENSMUSG00000024660	*Incenp*	ENSMUST00000025562.7	0.007568559	-0.271710215	57.2994
ENSMUSG00000045969	*Ing1*	ENSMUST00000209565.1	8.07E-05	0.527771861	32.5295
ENSMUSG00000037035	*Inhbb*	ENSMUST00000038765.5	5.63E-11	0.866529022	9.57301
ENSMUSG00000005534	*Insr*	ENSMUST00000091291.4	1.30E-18	0.950493235	8.66126
ENSMUSG00000031864	*Ints10*	ENSMUST00000110241.7	0.000610849	0.412060307	23.5706
ENSMUSG00000029034	*Ints11*	ENSMUST00000030901.8	0.003137267	-0.352503871	41.6115
ENSMUSG00000027933	*Ints3*	ENSMUST00000029542.11	1.82E-05	-0.457365999	20.2694
ENSMUSG00000002319	*Ipo4*	ENSMUST00000047131.15	0.009182249	-0.264859654	20.4988
ENSMUSG00000030662	*Ipo5*	ENSMUST00000032898.7	0.000274409	-0.320801847	31.6895
ENSMUSG00000021676	*Iqgap2*	ENSMUST00000068603.6	1.87E-17	-1.309157575	9.5916
ENSMUSG00000031392	*Irak1*	ENSMUST00000114354.9	2.26E-05	0.497437487	30.6542
ENSMUSG00000032251	*Irak1bp1*	ENSMUST00000113245.8	0.002438536	0.427676713	15.3651
ENSMUSG00000032293	*Ireb2*	ENSMUST00000034843.8	0.005762428	-0.234653451	35.5234
ENSMUSG00000031627	*Irf2*	ENSMUST00000034041.8	8.44E-07	0.627469021	18.2787
ENSMUSG00000038894	*Irs2*	ENSMUST00000040514.7	1.18E-11	0.769739749	18.2509
ENSMUSG00000001504	*Irx2*	ENSMUST00000074372.5	0.004689951	-0.771606729	3.48915
ENSMUSG00000044792	*Isca1*	ENSMUST00000057115.6	0.000885614	-0.299525284	69.4035
ENSMUSG00000035692	*Isg15*	ENSMUST00000085425.4	6.00E-45	-1.173700683	412.029
ENSMUSG00000039236	*Isg20*	ENSMUST00000038142.14	0.001038319	0.512532191	23.6718
ENSMUSG00000048039	*Isg20l2*	ENSMUST00000055984.6	0.006191645	-0.283765755	21.6026
ENSMUSG00000037206	*Islr*	ENSMUST00000041477.14	7.92E-06	-0.976933812	14.9197
ENSMUSG00000031729	*Ist1*	ENSMUST00000034164.5	1.82E-08	0.543919148	67.01
ENSMUSG00000031703	*Itfg1*	ENSMUST00000034140.8	2.65E-13	0.645182469	72.5528
ENSMUSG00000042284	*Itga1*	ENSMUST00000224865.1	1.16E-20	-1.642230131	3.08876
ENSMUSG00000015533	*Itga2*	ENSMUST00000056117.9	0.000730023	-1.694791069	0.0483624
ENSMUSG00000027009	*Itga4*	ENSMUST00000099972.4	0.000732853	0.949727708	0.493478
ENSMUSG00000039115	*Itga9*	ENSMUST00000044165.13	0.004990228	-1.677215169	2.1568
ENSMUSG00000027087	*Itgav*	ENSMUST00000028499.10	0.002102552	-0.256960777	57.5167
ENSMUSG00000025809	*Itgb1*	ENSMUST00000090006.11	1.61E-09	0.567366934	585.444
ENSMUSG00000020689	*Itgb3*	ENSMUST00000021028.4	1.26E-11	-1.027969115	4.91946
ENSMUSG00000022108	*Itm2b*	ENSMUST00000022704.7	0.000102959	-0.327802895	345.643
ENSMUSG00000074797	*Itpa*	ENSMUST00000103193.4	0.008452337	-0.257140829	71.4364
ENSMUSG00000042644	*Itpr3*	ENSMUST00000049308.8	0.005426637	0.35884656	11.4026
ENSMUSG00000095115	*Itpripl2*	ENSMUST00000178344.2	0.000199145	0.315693059	43.565
ENSMUSG00000027332	*Ivd*	ENSMUST00000028807.5	4.20E-13	-0.826894399	7.94125
ENSMUSG00000027276	*Jag1*	ENSMUST00000028735.7	0.002313179	-0.539510878	11.918
ENSMUSG00000024502	*Jakmip2*	ENSMUST00000082254.6	1.52E-13	1.208804253	7.86805
ENSMUSG00000098789	*Jmjd7*	ENSMUST00000044675.4	0.005880832	-0.550480754	5.88883
ENSMUSG00000024165	*Jpt2*	ENSMUST00000024981.7	0.00345631	-0.246949747	90.5236
ENSMUSG00000052837	*Junb*	ENSMUST00000064922.6	0.008905432	0.751588852	38.8502
ENSMUSG00000032702	*Kank1*	ENSMUST00000049400.14	0.006066204	0.687959432	2.76615
ENSMUSG00000031948	*Kars*	ENSMUST00000034426.13	0.003192041	0.262772674	112.38
ENSMUSG00000031540	*Kat6a*	ENSMUST00000044331.6	0.000118503	0.447497577	15.9384
ENSMUSG00000031787	*Katnb1*	ENSMUST00000034239.8	0.000977138	0.468765832	14.9149
ENSMUSG00000055675	*Kbtbd11*	ENSMUST00000069399.6	5.87E-09	0.800042063	6.25334
ENSMUSG00000059486	*Kbtbd2*	ENSMUST00000114323.7	0.000240888	0.339543478	50.8357
ENSMUSG00000041695	*Kcnj2*	ENSMUST00000042970.2	0.002544896	-0.410216427	3.96443
ENSMUSG00000054342	*Kcnn4*	ENSMUST00000205428.1	0.007945285	1.004335822	11.0645
ENSMUSG00000028631	*Kcnq4*	ENSMUST00000030376.7	0.000445416	-0.878953772	0.544379
ENSMUSG00000052726	*Kcnt2*	ENSMUST00000120709.7	2.06E-05	0.876503477	6.66499
ENSMUSG00000026608	*Kctd3*	ENSMUST00000085678.7	0.007565285	0.306394937	19.5276

Table S3. RNAseq in D5 in mDPCs-CAS9 cells and sgCreb3l1 _A8_4: 1007 downregulated genes and 1027 upregulated genes

ENSMUSG00000033326	*Kdm4a*	ENSMUST00000106406.8	4.13E-05	-0.396803641	36.5228
ENSMUSG00000024201	*Kdm4b*	ENSMUST00000025036.10	0.000532264	-0.350767243	17.9054
ENSMUSG00000042207	*Kdm5b*	ENSMUST00000112198.2	4.50E-06	-0.625351612	4.08857
ENSMUSG00000056673	*Kdm5d*	ENSMUST00000187296.1	7.07E-10	0.725338774	18.5598
ENSMUSG00000042599	*Kdm7a*	ENSMUST00000002305.8	2.93E-05	0.49827002	5.12457
ENSMUSG00000022332	*Khdrbs3*	ENSMUST00000022954.6	4.39E-07	0.506054672	58.2727
ENSMUSG00000007670	*Khsrp*	ENSMUST00000007814.8	0.001890795	-0.264249704	56.6225
ENSMUSG00000038844	*Kif16b*	ENSMUST00000043589.7	0.000270207	-0.438578294	8.21059
ENSMUSG00000063077	*Kif1b*	ENSMUST00000030806.5	0.001363709	-0.285613831	19.4859
ENSMUSG00000024795	*Kif20b*	ENSMUST00000087341.6	0.001008977	-0.378449983	13.9163
ENSMUSG00000041642	*Kif21b*	ENSMUST00000075164.10	0.001338752	0.340552091	8.2335
ENSMUSG00000021693	*Kif2a*	ENSMUST00000117423.8	0.002018941	-0.374296293	20.7199
ENSMUSG00000028678	*Kif2c*	ENSMUST00000065896.8	0.006314175	-0.243541613	47.9056
ENSMUSG00000021288	*Klc1*	ENSMUST00000084941.11	0.009989718	-0.380707289	47.4315
ENSMUSG00000037465	*Klf10*	ENSMUST00000074043.5	0.002853852	0.29367819	32.5056
ENSMUSG00000052040	*Klf13*	ENSMUST00000063694.9	0.000313857	0.364770499	16.4197
ENSMUSG00000030087	*Klf15*	ENSMUST00000032174.11	8.23E-06	-3.874343616	0.17487
ENSMUSG00000025959	*Klf7*	ENSMUST00000114086.7	7.66E-06	0.474410828	11.3385
ENSMUSG00000029775	*Klhdc10*	ENSMUST00000068259.9	0.006676812	0.24278847	33.7133
ENSMUSG00000040263	*Klhdc4*	ENSMUST00000045884.16	0.000153031	0.545981224	27.8477
ENSMUSG00000031605	*Klhl2*	ENSMUST00000034017.8	1.82E-10	0.832941494	13.8553
ENSMUSG00000026705	*Klhl20*	ENSMUST00000111611.7	0.009925533	0.241690638	32.8385
ENSMUSG00000042155	*Klhl23*	ENSMUST00000053087.3	2.76E-08	0.877231027	5.571
ENSMUSG00000079852	*Klra4*	ENSMUST00000119096.1	0.000361498	2.5637302	1.11091
ENSMUSG00000018362	*Kpna2*	ENSMUST00000018506.12	2.06E-24	1.354599227	63.1469
ENSMUSG00000027782	*Kpna4*	ENSMUST00000194558.5	0.005991161	-0.235179435	54.6726
ENSMUSG00000003731	*Kpna6*	ENSMUST00000102590.10	0.0065708	-0.234993431	34.5428
ENSMUSG00000040213	*Kyat3*	ENSMUST00000106218.7	0.004530694	-0.579973408	3.22831
ENSMUSG00000025937	*Lactb2*	ENSMUST00000027071.6	1.73E-10	0.649420742	20.9515
ENSMUSG00000032796	*Lama1*	ENSMUST00000035471.7	1.24E-05	-0.878556325	0.420031
ENSMUSG00000031447	*Lamp1*	ENSMUST00000033824.7	1.26E-06	0.490803512	1610.63
ENSMUSG00000016534	*Lamp2*	ENSMUST00000016678.13	5.84E-05	-0.346292781	438.369
ENSMUSG00000062190	*Lancl2*	ENSMUST00000050077.14	0.008164543	0.284684367	29.7339
ENSMUSG00000038366	*Lasp1*	ENSMUST00000043843.11	2.72E-05	-0.349898471	95.5009
ENSMUSG00000024063	*Lbh*	ENSMUST00000024857.12	1.70E-05	-0.36625105	66.6133
ENSMUSG00000004880	*Lbr*	ENSMUST00000005003.11	2.71E-05	0.374511299	51.1009
ENSMUSG00000035237	*Lcat*	ENSMUST00000038896.7	0.00350261	0.766415681	4.65951
ENSMUSG00000025223	*Ldb1*	ENSMUST00000026252.13	1.26E-06	-0.562621721	34.8841
ENSMUSG00000021798	*Ldb3*	ENSMUST00000022328.12	0.001580626	-3.977576405	0
ENSMUSG00000057722	*Lepr*	ENSMUST00000106921.8	0.009278133	0.551446495	15.173
ENSMUSG00000031513	*Leprotl1*	ENSMUST00000033910.8	4.67E-18	0.910215706	37.3434
ENSMUSG00000033880	*Lgals3bp*	ENSMUST00000043722.9	1.78E-07	-0.711643225	113.939
ENSMUSG00000042363	*Lgalsl*	ENSMUST00000047028.8	2.31E-05	0.418898338	33.3452
ENSMUSG00000050199	*Lgr4*	ENSMUST00000046548.13	1.43E-13	-0.724822585	24.9026
ENSMUSG00000020140	*Lgr5*	ENSMUST00000020350.14	0.001223739	1.829393573	7.31745
ENSMUSG00000048332	*Lhfp*	ENSMUST00000059562.13	0.000599339	-0.299937532	104.257
ENSMUSG00000041700	*Lhfpl1*	ENSMUST00000040084.9	0.003447663	-2.807610536	0
ENSMUSG00000045312	*Lhfpl2*	ENSMUST00000054274.7	1.28E-05	-0.583172499	39.6573
ENSMUSG00000010721	*Lmbr1*	ENSMUST00000055195.10	0.004635324	0.500043756	3.56865
ENSMUSG00000047866	*Lonp2*	ENSMUST00000034141.15	1.33E-07	0.516171804	49.0369
ENSMUSG00000039633	*Lonrf1*	ENSMUST00000065297.5	0.00406164	0.537130231	6.60008
ENSMUSG00000024529	*Lox*	ENSMUST00000025409.7	0.000178543	0.540892765	137.667
ENSMUSG00000034205	*Loxl2*	ENSMUST00000022660.13	1.65E-58	-1.374495303	28.7917
ENSMUSG00000033306	*Lpp*	ENSMUST00000038053.12	0.009941679	-0.313281792	10.2901
ENSMUSG00000022305	*Lrp12*	ENSMUST00000022916.11	0.007495589	0.264683786	25.9383
ENSMUSG00000024913	*Lrp5*	ENSMUST00000025856.16	0.000433232	-0.390816144	8.79436
ENSMUSG00000029103	*Lrpap1*	ENSMUST00000030986.14	0.007005191	-0.363110061	41.1761
ENSMUSG00000024120	*Lrpprc*	ENSMUST00000112308.8	0.000503015	-0.320173789	26.7724
ENSMUSG00000052316	*Lrrc15*	ENSMUST00000064606.7	0.000285618	1.609148353	46.7396
ENSMUSG00000028617	*Lrrc42*	ENSMUST00000030360.10	0.000248724	-0.324791018	96.1869
ENSMUSG00000025145	*Lrrc45*	ENSMUST00000026139.13	0.007419438	-0.344029846	13.3929
ENSMUSG00000070639	*Lrrc8b*	ENSMUST00000112707.2	0.006550504	0.557599113	14.3822
ENSMUSG00000046589	*Lrrc8e*	ENSMUST00000053035.6	1.05E-07	0.86961426	5.32388
ENSMUSG00000026305	*Lrrfip1*	ENSMUST00000097649.9	1.27E-06	0.464029494	34.2098
ENSMUSG00000036273	*Lrrk2*	ENSMUST00000060642.6	0.002244109	0.301541398	10.7951
ENSMUSG00000071656	*Lrrn4cl*	ENSMUST00000096257.2	7.49E-05	0.632615272	8.51269
ENSMUSG00000037296	*Lsm1*	ENSMUST00000038421.7	0.00020088	0.462334321	19.0561
ENSMUSG00000031848	*Lsm4*	ENSMUST00000034311.14	0.009509193	0.422557699	57.4978
ENSMUSG00000031683	*Lsm6*	ENSMUST00000146824.1	0.005837783	0.785794841	30.7786
ENSMUSG00000044155	*Lsm8*	ENSMUST00000056398.10	0.000899452	0.323416885	183.641
ENSMUSG00000018819	*Lsp1*	ENSMUST00000038946.8	6.64E-05	0.693431753	15.9581
ENSMUSG00000036446	*Lum*	ENSMUST00000038160.5	0.00972519	0.81575567	68.7814

ENSMUSG00000039246	*Lyplal1*	ENSMUST00000045388.7	3.93E-10	0.935542537	22.7893
ENSMUSG00000045854	*Lyrm2*	ENSMUST00000062802.4	0.005598064	-0.473625692	55.2515
ENSMUSG00000069516	*Lyz2*	ENSMUST00000092163.8	0.000344528	-1.347476112	0.796052
ENSMUSG00000028990	*Lzic*	ENSMUST00000030842.7	7.31E-05	-0.510964426	41.639
ENSMUSG00000056947	*Mab21l1*	ENSMUST00000075422.5	1.55E-07	-0.796172422	5.43731
ENSMUSG00000029910	*Mad2l1*	ENSMUST00000101343.1	0.000117874	0.358751203	90.8306
ENSMUSG00000034509	*Mad2l1bp*	ENSMUST00000171172.1	0.003568146	-0.386081517	23.3474
ENSMUSG00000055435	*Maf*	ENSMUST00000109104.1	0.00740701	0.239207328	26.9018
ENSMUSG00000074622	*Mafb*	ENSMUST00000099126.4	4.77E-16	1.7485391	5.44444
ENSMUSG00000018143	*Mafk*	ENSMUST00000018287.9	0.008929198	0.324125111	15.6253
ENSMUSG00000025268	*Maged2*	ENSMUST00000112700.7	6.38E-05	-0.365745105	68.3213
ENSMUSG00000047238	*Mageh1*	ENSMUST00000051484.4	9.69E-14	1.132247287	28.8312
ENSMUSG00000045095	*Magi1*	ENSMUST00000203519.2	0.006771566	-0.457118355	5.03524
ENSMUSG00000031147	*Magix*	ENSMUST00000130287.1	0.003476331	0.921653135	2.05493
ENSMUSG00000031578	*Mak16*	ENSMUST00000033983.5	1.31E-05	0.464146677	40.4465
ENSMUSG00000075520	*Malrd1*	ENSMUST00000146205.2	0.009081195	-2.626245265	0
ENSMUSG00000032688	*Malt1*	ENSMUST00000049248.5	0.009305556	0.531797464	2.53563
ENSMUSG00000031925	*Maml2*	ENSMUST00000159294.8	0.007506644	-0.565315539	7.63624
ENSMUSG00000059401	*Mamld1*	ENSMUST00000082088.9	7.96E-05	-3.294987631	0.110265
ENSMUSG00000036646	*Man1b1*	ENSMUST00000042390.4	0.000187584	-0.313743184	71.6973
ENSMUSG00000040520	*Manea*	ENSMUST00000041374.7	3.61E-06	-0.415060196	24.5221
ENSMUSG00000032718	*Mansc1*	ENSMUST00000047443.4	0.001629995	-2.352236823	0.118112
ENSMUSG00000025037	*Maoa*	ENSMUST00000026013.5	4.49E-05	0.442014702	55.3713
ENSMUSG00000040147	*Maob*	ENSMUST00000040820.12	3.39E-05	0.737097494	19.943
ENSMUSG00000027254	*Map1a*	ENSMUST00000110639.7	2.75E-13	-0.648112371	11.9667
ENSMUSG00000031812	*Map1lc3b*	ENSMUST00000034270.16	1.34E-17	0.722062213	180.997
ENSMUSG00000018932	*Map2k3*	ENSMUST00000019076.9	0.007323258	-0.246945634	51.7128
ENSMUSG00000002948	*Map2k7*	ENSMUST00000062686.10	4.35E-08	0.583573596	35.4623
ENSMUSG00000031303	*Map3k15*	ENSMUST00000033665.8	0.000397699	1.21595273	1.6492
ENSMUSG00000071369	*Map3k5*	ENSMUST00000095806.9	0.009828541	-0.474910717	1.5967
ENSMUSG00000042688	*Mapk6*	ENSMUST00000049355.10	0.001480193	0.287820423	52.4951
ENSMUSG00000032577	*Mapkapk3*	ENSMUST00000035194.7	1.25E-06	-0.79746232	3.09016
ENSMUSG00000033902	*Mapkbp1*	ENSMUST00000066058.7	0.00781249	-0.266645438	10.3723
ENSMUSG00000031833	*Mast3*	ENSMUST00000166004.2	0.005735212	0.519221916	3.93583
ENSMUSG00000034751	*Mast4*	ENSMUST00000167058.7	0.000376423	-0.538432013	3.62489
ENSMUSG00000042032	*Mat2b*	ENSMUST00000040167.10	0.004554002	0.348104277	33.909
ENSMUSG00000024513	*Mbd2*	ENSMUST00000074058.10	8.21E-05	0.362707122	89.0173
ENSMUSG00000035596	*Mboat7*	ENSMUST00000038608.13	0.005014943	-0.429950446	20.3121
ENSMUSG00000041607	*Mbp*	ENSMUST00000091789.10	0.001291358	0.87350931	0.967228
ENSMUSG00000031835	*Mbtps1*	ENSMUST00000081381.5	1.35E-11	0.565700206	148.443
ENSMUSG00000027709	*Mccc1*	ENSMUST00000029259.9	8.84E-06	-0.554090203	10.3779
ENSMUSG00000024150	*Mcfd2*	ENSMUST00000024963.9	8.18E-06	-0.412783585	55.7398
ENSMUSG00000050164	*Mchr1*	ENSMUST00000166855.1	0.000119105	1.308343614	1.98938
ENSMUSG00000002870	*Mcm2*	ENSMUST00000058011.7	0.005243421	0.311352984	376.151
ENSMUSG00000041859	*Mcm3*	ENSMUST00000053266.10	0.008685062	0.219696477	207.951
ENSMUSG00000005410	*Mcm5*	ENSMUST00000212426.1	4.47E-06	0.562297369	87.4085
ENSMUSG00000039842	*Mcph1*	ENSMUST00000039412.14	0.001515917	0.401299258	8.70181
ENSMUSG00000043557	*Mdga1*	ENSMUST00000165528.7	0.003153331	-1.171897905	0.802283
ENSMUSG00000020184	*Mdm2*	ENSMUST00000020408.15	0.001119337	-0.297897011	40.5073
ENSMUSG00000032418	*Me1*	ENSMUST00000034989.14	0.000758679	0.295714958	61.8498
ENSMUSG00000028910	*Mecr*	ENSMUST00000030742.10	0.00138911	-0.464677656	15.0355
ENSMUSG00000056476	*Med12l*	ENSMUST00000040325.13	4.52E-10	1.398027115	2.44991
ENSMUSG00000027080	*Med19*	ENSMUST00000102645.3	0.000106786	-0.386920247	38.0475
ENSMUSG00000015776	*Med22*	ENSMUST00000102899.9	0.000322171	-0.324909218	38.4663
ENSMUSG00000022109	*Med4*	ENSMUST00000022705.5	0.003812434	-0.328026201	39.7853
ENSMUSG00000029659	*Medag*	ENSMUST00000093110.11	0.000736031	-0.320834138	34.4175
ENSMUSG00000001419	*Mef2d*	ENSMUST00000119251.7	0.002624856	-0.381696951	11.0303
ENSMUSG00000043289	*Mei4*	ENSMUST00000057067.9	0.006041808	1.831110282	1.66685
ENSMUSG00000058704	*Memo1*	ENSMUST00000078459.6	0.005479828	-0.286333982	44.6887
ENSMUSG00000021891	*Mettl6*	ENSMUST00000055303.3	0.005949458	-0.351210728	11.0616
ENSMUSG00000031647	*Mfap3l*	ENSMUST00000160719.7	8.34E-05	1.323030053	1.06025
ENSMUSG00000033174	*Mgll*	ENSMUST00000113585.8	8.14E-05	-1.006390578	0.819763
ENSMUSG00000008540	*Mgst1*	ENSMUST00000008684.10	0.00602018	2.413733467	13.313
ENSMUSG00000019823	*Mical1*	ENSMUST00000019967.15	0.003835811	-0.440730971	18.5129
ENSMUSG00000039478	*Micu3*	ENSMUST00000136835.1	0.000108417	0.810355082	3.76289
ENSMUSG00000008035	*Mid1ip1*	ENSMUST00000008179.6	0.007421181	0.297423732	45.429
ENSMUSG00000035621	*Midn*	ENSMUST00000099492.9	0.009991533	-0.442550124	41.0123
ENSMUSG00000054942	*Miga1*	ENSMUST00000199397.1	1.93E-05	-0.531264954	10.0065
ENSMUSG00000026858	*Miga2*	ENSMUST00000077977.13	0.000674331	-0.36098772	18.409
ENSMUSG00000024896	*Minpp1*	ENSMUST00000025827.8	0.000583128	-0.350298772	36.9761
ENSMUSG00000029922	*Mkrn1*	ENSMUST00000031985.12	0.004810246	0.334485854	27.1897
ENSMUSG00000061013	*Mkx*	ENSMUST00000079788.5	0.00974407	0.938720024	16.2829

ENSMUSG00000024806	*Mlana*	ENSMUST00000025719.3	2.00E-08	-2.118104022	1.67635
ENSMUSG00000074064	*Mlycd*	ENSMUST00000098367.4	0.000176073	0.660436942	9.03227
ENSMUSG00000031790	*Mmp15*	ENSMUST00000034243.6	0.001493822	-1.7071995	0.153142
ENSMUSG00000028226	*Mmp16*	ENSMUST00000142434.7	0.009492048	-0.505866769	18.5417
ENSMUSG00000031740	*Mmp2*	ENSMUST00000034187.8	0.000508137	0.349104498	662.135
ENSMUSG00000025159	*Mms19*	ENSMUST00000171755.7	0.005205012	-0.513097842	7.20086
ENSMUSG00000043131	*Mob1a*	ENSMUST00000055261.10	0.000136706	0.320336547	148.499
ENSMUSG00000028709	*Mob3c*	ENSMUST00000030477.3	0.004276599	-0.451630659	4.55099
ENSMUSG00000056458	*Mok*	ENSMUST00000070565.14	0.007470739	-0.852347353	1.24964
ENSMUSG00000078908	*Mon1b*	ENSMUST00000035777.9	6.21E-06	0.629649729	6.96262
ENSMUSG00000031843	*Mphosph6*	ENSMUST00000034303.2	0.0001559	0.530806871	38.7839
ENSMUSG00000038388	*Mpp6*	ENSMUST00000036236.14	5.72E-09	0.7257225	45.2606
ENSMUSG00000057440	*Mpp7*	ENSMUST00000115869.2	7.12E-05	0.503897911	8.66172
ENSMUSG00000031070	*Mrgprf*	ENSMUST00000033386.11	0.000445974	0.448857356	26.3339
ENSMUSG00000022558	*Mroh1*	ENSMUST00000096385.10	0.009074117	0.223421002	50.0315
ENSMUSG00000029486	*Mrpl1*	ENSMUST00000121477.1	0.00104284	-0.685747915	5.72054
ENSMUSG00000024902	*Mrpl11*	ENSMUST00000025836.4	0.000871295	-0.406413587	10.9892
ENSMUSG00000024829	*Mrpl21*	ENSMUST00000025743.6	0.009524501	-0.383079644	37.803
ENSMUSG00000075279	*Mrpl23-ps1*	ENSMUST00000173086.1	0.006964185	0.418651673	84.7399
ENSMUSG00000024181	*Mrpl28*	ENSMUST00000025014.8	0.000385395	-0.342434124	106.845
ENSMUSG00000028622	*Mrpl37*	ENSMUST00000030365.5	0.000251247	-0.440355749	57.5662
ENSMUSG00000036850	*Mrpl41*	ENSMUST00000045604.3	0.000126316	-0.458639673	58.7639
ENSMUSG00000007338	*Mrpl49*	ENSMUST00000007482.6	6.28E-05	-0.375888727	60.2435
ENSMUSG00000010406	*Mrpl52*	ENSMUST00000010550.11	0.003913609	-0.44859699	93.6383
ENSMUSG00000028140	*Mrpl9*	ENSMUST00000029786.13	0.001250095	-0.340544401	45.2381
ENSMUSG00000049960	*Mrps16*	ENSMUST00000224286.1	0.009226731	0.352064172	62.0789
ENSMUSG00000023967	*Mrps18a*	ENSMUST00000024763.9	0.005498859	-0.296406959	81.8127
ENSMUSG00000041632	*Mrps27*	ENSMUST00000052249.6	0.008468317	-0.307914258	20.5736
ENSMUSG00000031533	*Mrps31*	ENSMUST00000033934.4	0.000185642	0.479413879	29.8698
ENSMUSG00000026887	*Mrrf*	ENSMUST00000028250.8	0.003276714	-0.33139237	17.3494
ENSMUSG00000005611	*Mrvi1*	ENSMUST00000127935.7	0.000620533	-1.691933544	0.183035
ENSMUSG00000069769	*Msi2*	ENSMUST00000107909.7	9.17E-11	0.925869522	5.7569
ENSMUSG00000063011	*Msln*	ENSMUST00000075884.6	2.32E-12	3.4454434	5.26397
ENSMUSG00000064357	*mt-Atp6*	ENSMUST00000082408.1	0.001169128	-0.368163933	4773.21
ENSMUSG00000064345	*mt-Nd2*	ENSMUST00000082396.1	0.002702182	-0.265091968	1182.69
ENSMUSG00000031765	*Mt1*	ENSMUST00000034215.7	1.59E-05	0.741413834	1253.07
ENSMUSG00000071646	*Mta2*	ENSMUST00000096240.2	9.68E-06	-0.376326176	67.857
ENSMUSG00000062937	*Mtap*	ENSMUST00000058030.9	0.005797075	-0.234300329	81.3158
ENSMUSG00000031816	*Mthfsd*	ENSMUST00000133037.7	4.25E-09	0.934164419	29.2258
ENSMUSG00000015214	*Mtmr1*	ENSMUST00000015358.7	0.009267166	0.271369891	16.3926
ENSMUSG00000021311	*Mtr*	ENSMUST00000099856.5	0.006724791	-0.27845496	7.38783
ENSMUSG00000024906	*Mus81*	ENSMUST00000124334.7	0.009606319	-0.346177193	17.4457
ENSMUSG00000023341	*Mx2*	ENSMUST00000188251.6	0.000199707	-0.649467994	10.2351
ENSMUSG00000024048	*Myl12a*	ENSMUST00000148960.7	5.21E-05	-0.350941897	389.106
ENSMUSG00000034868	*Myl12b*	ENSMUST00000038446.8	0.001603537	-0.279432486	144.27
ENSMUSG00000090841	*Myl6*	ENSMUST00000164181.1	0.001443226	-0.269144788	1058.81
ENSMUSG00000022836	*Mylk*	ENSMUST00000023538.8	6.96E-08	-0.542811761	7.19099
ENSMUSG00000035441	*Myo1d*	ENSMUST00000041065.13	0.003320417	0.336605402	12.8923
ENSMUSG00000066952	*Myo1h*	ENSMUST00000196676.4	0.001073386	0.879214537	8.93269
ENSMUSG00000031652	*N4bp1*	ENSMUST00000034074.7	2.15E-07	0.482508818	21.2681
ENSMUSG00000041132	*N4bp2l1*	ENSMUST00000016279.10	0.001195831	0.912137116	4.72175
ENSMUSG00000043943	*Naalad2*	ENSMUST00000166825.7	9.56E-15	1.931899469	5.81627
ENSMUSG00000001910	*Nacc1*	ENSMUST00000001975.4	2.69E-06	0.406427977	61.2701
ENSMUSG00000026932	*Nacc2*	ENSMUST00000028300.5	6.25E-05	-0.468688716	8.8769
ENSMUSG00000031878	*Nae1*	ENSMUST00000034349.9	0.003945024	0.465899957	22.556
ENSMUSG00000014907	*Naf1*	ENSMUST00000118009.1	4.32E-14	0.965695809	18.1509
ENSMUSG00000012396	*Nanog*	ENSMUST00000012540.4	0.005067989	0.724385581	3.12191
ENSMUSG00000082229	*Nap1l2*	ENSMUST00000121720.1	9.63E-06	1.149924779	2.94589
ENSMUSG00000028693	*Nasp*	ENSMUST00000030457.11	2.62E-08	-0.546846901	39.382
ENSMUSG00000051147	*Nat2*	ENSMUST00000093470.6	0.004070816	0.973729172	3.35227
ENSMUSG00000031505	*Naxd*	ENSMUST00000033901.10	3.37E-08	0.959240608	22.3573
ENSMUSG00000041120	*Nbl1*	ENSMUST00000042844.6	1.14E-15	-0.794599498	88.3089
ENSMUSG00000028224	*Nbn*	ENSMUST00000029879.14	0.003880483	-0.279647733	40.0691
ENSMUSG00000028330	*Ncbp1*	ENSMUST00000030014.8	0.007798369	-0.230047628	63.3049
ENSMUSG00000023009	*Nckap5l*	ENSMUST00000023747.13	0.002066409	-0.465184444	5.92073
ENSMUSG00000027678	*Ncoa3*	ENSMUST00000109252.7	0.000287948	-0.470392851	6.10966
ENSMUSG00000039697	*Ncoa7*	ENSMUST00000216172.1	0.00016337	-0.968072119	2.63908
ENSMUSG00000049001	*Ndnf*	ENSMUST00000169795.1	8.32E-06	0.832213412	97.1699
ENSMUSG00000005125	*Ndrg1*	ENSMUST00000005256.12	7.35E-25	0.889294868	113.478
ENSMUSG00000002379	*Ndufa11*	ENSMUST00000002452.6	1.20E-05	-0.441131615	27.2431
ENSMUSG00000036199	*Ndufa13*	ENSMUST00000110167.4	1.53E-05	0.411023013	133.076
ENSMUSG00000023089	*Ndufa5*	ENSMUST00000023851.8	0.004645877	0.274190702	195.351

Table S3. RNAseq in D5 in mDPCs-CAS9 cells and sgCreb3l1 _A8_4: 1007 downregulated genes and 1027 upregulated genes

ENSMUSG00000026895	*Ndufa8*	ENSMUST00000070112.5	0.001102932	-0.304991482	171.436
ENSMUSG00000025204	*Ndufb8*	ENSMUST00000026222.10	0.003457783	-0.397614956	257.346
ENSMUSG00000005510	*Ndufs3*	ENSMUST00000005647.3	0.002928551	-0.325427066	60.0578
ENSMUSG00000021764	*Ndufs4*	ENSMUST00000225707.1	8.43E-06	-0.78356199	23.781
ENSMUSG00000037916	*Ndufv1*	ENSMUST00000042497.13	0.004397581	-0.270885263	69.0133
ENSMUSG00000024099	*Ndufv2*	ENSMUST00000143987.7	0.000282907	-0.390681225	44.748
ENSMUSG00000028923	*Necap2*	ENSMUST00000030760.14	0.001849577	-0.288068197	69.8493
ENSMUSG00000032012	*Nectin1*	ENSMUST00000034510.8	0.002190067	-0.415347514	4.179
ENSMUSG00000021365	*Nedd9*	ENSMUST00000163623.2	6.96E-05	-0.368176365	27.5407
ENSMUSG00000039396	*Neil3*	ENSMUST00000047768.10	1.20E-11	0.861289929	24.0895
ENSMUSG00000034290	*Nek9*	ENSMUST00000040992.7	0.001584056	-0.2665946	43.8292
ENSMUSG00000016253	*Nelfcd*	ENSMUST00000109075.7	0.000365966	-0.329961727	54.237
ENSMUSG00000022454	*Nell2*	ENSMUST00000075275.2	0.000380923	0.899653176	8.31557
ENSMUSG00000033016	*Nfatc1*	ENSMUST00000035800.7	0.000315932	0.610953121	8.01608
ENSMUSG00000031902	*Nfatc3*	ENSMUST00000109308.2	1.99E-08	0.50415602	37.2905
ENSMUSG00000022204	*Ngdn*	ENSMUST00000022815.8	0.000463751	-0.404641248	39.9716
ENSMUSG00000039835	*Nhsl1*	ENSMUST00000037341.13	0.004365376	0.459920172	7.48781
ENSMUSG00000079481	*Nhsl2*	ENSMUST00000101339.7	4.95E-05	-0.520730277	1.89457
ENSMUSG00000021910	*Nisch*	ENSMUST00000022469.12	0.006729145	-0.367449551	66.6362
ENSMUSG00000032525	*Nktr*	ENSMUST00000035112.12	0.000674166	0.292864421	36.4945
ENSMUSG00000049871	*Nlrc3*	ENSMUST00000180200.7	0.007243063	1.265713909	0.579882
ENSMUSG00000067786	*Nnat*	ENSMUST00000153739.8	0.003366866	-1.769228001	15.081
ENSMUSG00000036285	*Noa1*	ENSMUST00000047860.8	0.005358569	0.357534444	21.9592
ENSMUSG00000003848	*Nob1*	ENSMUST00000003946.8	0.000589973	0.400604117	38.8873
ENSMUSG00000024999	*Noc3l*	ENSMUST00000025963.7	0.003155284	-0.312099131	12.7709
ENSMUSG00000061411	*Nol4l*	ENSMUST00000109784.1	1.41E-14	-1.127132476	3.30901
ENSMUSG00000021392	*Nol8*	ENSMUST00000222197.1	0.008674373	-0.392640714	5.88196
ENSMUSG00000028948	*Nol9*	ENSMUST00000084116.12	0.007712866	-0.279859969	18.0786
ENSMUSG00000015176	*Nolc1*	ENSMUST00000223683.1	0.002070712	-0.344134651	25.428
ENSMUSG00000026923	*Notch1*	ENSMUST00000028288.4	1.32E-16	-1.275530267	4.41055
ENSMUSG00000027878	*Notch2*	ENSMUST00000079812.7	0.006628035	-0.230530667	20.29
ENSMUSG00000021242	*Npc2*	ENSMUST00000021668.9	2.04E-05	-0.426083808	198.959
ENSMUSG00000042684	*Npl*	ENSMUST00000041874.8	0.000502393	1.11576597	3.28863
ENSMUSG00000056209	*Npm3*	ENSMUST00000070215.7	4.94E-07	-0.662156294	35.7883
ENSMUSG00000036437	*Npy1r*	ENSMUST00000212588.1	0.001780928	3.279895087	3.48617
ENSMUSG00000069171	*Nr2f1*	ENSMUST00000091458.12	0.000401484	-0.675448858	3.27113
ENSMUSG00000031618	*Nr3c2*	ENSMUST00000109912.7	0.0001117	0.721134419	3.73676
ENSMUSG00000029148	*Nrbp1*	ENSMUST00000031034.11	0.004664009	-0.247345183	90.3463
ENSMUSG00000075590	*Nrbp2*	ENSMUST00000019516.4	0.000658741	0.316152646	50.2219
ENSMUSG00000021179	*Nrde2*	ENSMUST00000021596.8	0.008072611	-0.328728571	9.27729
ENSMUSG00000001520	*Nrip2*	ENSMUST00000001561.11	4.27E-05	0.665005545	18.1523
ENSMUSG00000025969	*Nrp2*	ENSMUST00000102822.8	0.005578636	0.494484219	43.0625
ENSMUSG00000060739	*Nsa2*	ENSMUST00000225410.1	0.00282351	-0.359392582	49.7196
ENSMUSG00000054823	*Nsd3*	ENSMUST00000146919.7	1.47E-06	0.632086494	16.793
ENSMUSG00000029126	*Nsg1*	ENSMUST00000031009.7	3.59E-10	0.727709488	499.61
ENSMUSG00000040331	*Nsmce4a*	ENSMUST00000160289.8	0.008080385	0.271438384	76.6871
ENSMUSG00000025041	*Nt5c2*	ENSMUST00000168536.7	0.008651887	0.355996867	16.7832
ENSMUSG00000071547	*Nt5dc2*	ENSMUST00000090212.4	1.35E-12	-0.668837038	60.304
ENSMUSG00000049107	*Ntf3*	ENSMUST00000050484.8	7.47E-05	1.80640778	7.81315
ENSMUSG00000031851	*Ntpcr*	ENSMUST00000034313.12	0.000525055	0.677441031	19.7118
ENSMUSG00000030659	*Nucb2*	ENSMUST00000032895.14	0.002840275	0.358786092	27.6657
ENSMUSG00000032565	*Nudt16*	ENSMUST00000035179.8	0.002113565	0.581427338	10.8571
ENSMUSG00000031754	*Nudt21*	ENSMUST00000212981.1	0.007264948	0.331762139	52.6456
ENSMUSG00000037349	*Nudt22*	ENSMUST00000041686.9	2.08E-06	-0.699489896	18.8852
ENSMUSG00000029310	*Nudt9*	ENSMUST00000031250.13	0.008591332	0.247460702	106.016
ENSMUSG00000039509	*Nup133*	ENSMUST00000044795.7	4.37E-06	0.451290479	22.9931
ENSMUSG00000016619	*Nup50*	ENSMUST00000165443.2	0.007904704	-0.228447299	39.3581
ENSMUSG00000109511	*Nup62*	ENSMUST00000057195.16	0.002214519	0.270607396	74.757
ENSMUSG00000032939	*Nup93*	ENSMUST00000079961.13	4.28E-09	0.51723635	87.691
ENSMUSG00000030717	*Nupr1*	ENSMUST00000032961.3	0.009204073	0.363968435	209.559
ENSMUSG00000008450	*Nutf2*	ENSMUST00000008594.8	3.61E-06	0.501344853	180.712
ENSMUSG00000010097	*Nxf1*	ENSMUST00000010248.3	0.000149537	-0.765235466	43.0606
ENSMUSG00000075592	*Nynrin*	ENSMUST00000168479.1	0.003468367	-0.417943118	11.5441
ENSMUSG00000032014	*Oaf*	ENSMUST00000034512.6	0.000778896	-0.453787885	55.8147
ENSMUSG00000011179	*Odc1*	ENSMUST00000171737.2	0.000499654	0.29732171	133.466
ENSMUSG00000021913	*Ogdhl*	ENSMUST00000022480.7	5.70E-10	-2.19772412	1.01576
ENSMUSG00000033009	*Ogfod1*	ENSMUST00000109556.8	0.000136993	0.437046393	11.6074
ENSMUSG00000049401	*Ogfr*	ENSMUST00000029087.3	9.93E-07	-0.427300376	71.4451
ENSMUSG00000046618	*Olfml2a*	ENSMUST00000057279.5	6.55E-07	-1.053815105	0.965875
ENSMUSG00000038463	*Olfml2b*	ENSMUST00000046792.8	0.005534087	0.482664686	148.061
ENSMUSG00000108218	*Olfr1372-ps1*	ENSMUST00000203403.2	1.08E-07	0.704766456	3.77081
ENSMUSG00000040328	*Olfr56*	ENSMUST00000203412.1	0.009064266	-1.777582418	0.0995879

ENSMUSG00000028587	*Orc1*	ENSMUST00000102744.3	0.009884721	-0.285808225	13.6767
ENSMUSG00000026761	*Orc4*	ENSMUST00000154784.7	0.001224487	-0.716495144	9.38382
ENSMUSG00000031697	*Orc6*	ENSMUST00000034132.12	4.50E-07	0.521040246	88.9017
ENSMUSG00000027416	*Otor*	ENSMUST00000028902.2	4.47E-08	-3.81043051	0.369583
ENSMUSG00000024767	*Otub1*	ENSMUST00000025679.10	1.22E-05	-0.396542757	80.6536
ENSMUSG00000036990	*Otud4*	ENSMUST00000173078.7	0.001227207	0.667531296	12.3656
ENSMUSG00000022186	*Oxct1*	ENSMUST00000110690.8	0.00464956	-0.237676615	117.082
ENSMUSG00000027071	*P2rx3*	ENSMUST00000145285.1	0.007885796	1.794318079	11.461
ENSMUSG00000022758	*P2rx6*	ENSMUST00000023441.10	0.000134547	-1.02636925	3.64407
ENSMUSG00000029468	*P2rx7*	ENSMUST00000100737.9	0.000129853	0.752786975	7.63257
ENSMUSG00000021143	*Pacs2*	ENSMUST00000084891.4	0.003522466	-0.447246642	18.6002
ENSMUSG00000089945	*Pakap*	ENSMUST00000150412.3	0.000434715	-0.545059366	16.4778
ENSMUSG00000026335	*Pam*	ENSMUST00000058762.14	1.41E-11	0.743115371	42.3135
ENSMUSG00000024899	*Papss2*	ENSMUST00000025833.6	1.97E-10	-1.127682165	5.24201
ENSMUSG00000063268	*Parp10*	ENSMUST00000075689.6	2.08E-05	-0.411853285	38.092
ENSMUSG00000021725	*Parp8*	ENSMUST00000223949.1	0.000824545	-0.39894625	16.0872
ENSMUSG00000030770	*Parva*	ENSMUST00000106643.7	0.001059018	-0.330103413	47.1652
ENSMUSG00000046139	*Patl1*	ENSMUST00000061618.7	0.006534169	-0.251862445	23.2907
ENSMUSG00000042613	*Pbxip1*	ENSMUST00000038942.9	1.90E-12	-0.628728079	26.1047
ENSMUSG00000020098	*Pcbd1*	ENSMUST00000020298.7	0.008775893	0.903112759	5.50143
ENSMUSG00000041650	*Pcca*	ENSMUST00000038374.12	0.000426339	-0.594805868	4.56964
ENSMUSG00000051375	*Pcdh1*	ENSMUST00000159405.2	0.002095641	-1.820046423	0.818767
ENSMUSG00000037892	*Pcdh18*	ENSMUST00000035931.12	4.88E-06	-0.395500201	36.2733
ENSMUSG00000044043	*Pcdhb14*	ENSMUST00000052387.4	2.67E-07	0.929970865	2.03158
ENSMUSG00000047910	*Pcdhb16*	ENSMUST00000051442.6	0.000285155	0.616397829	3.30529
ENSMUSG00000046387	*Pcdhb17*	ENSMUST00000053856.5	0.002176375	0.384059149	8.95193
ENSMUSG00000048347	*Pcdhb18*	ENSMUST00000055949.3	2.43E-08	0.929769049	3.36365
ENSMUSG00000043313	*Pcdhb19*	ENSMUST00000059571.6	0.001348862	0.664041766	1.1797
ENSMUSG00000046191	*Pcdhb20*	ENSMUST00000052179.7	0.000132543	0.875526417	1.93823
ENSMUSG00000044022	*Pcdhb21*	ENSMUST00000061405.5	0.000366683	0.636792461	2.85052
ENSMUSG00000073591	*Pcdhb22*	ENSMUST00000192409.1	5.82E-05	0.530866519	5.04241
ENSMUSG00000104346	*Pcdhga3*	ENSMUST00000073447.7	4.82E-05	-0.611524324	3.59988
ENSMUSG00000103793	*Pcdhga6*	ENSMUST00000195823.1	0.001267385	-0.996408563	0.654146
ENSMUSG00000038542	*Pcid2*	ENSMUST00000164416.7	8.05E-07	0.531019613	30.385
ENSMUSG00000040204	*Pclaf*	ENSMUST00000130308.1	3.39E-05	-0.491963381	97.3173
ENSMUSG00000031592	*Pcm1*	ENSMUST00000211247.1	0.000141119	0.453598879	20.7965
ENSMUSG00000051285	*Pcmtd1*	ENSMUST00000061280.16	1.63E-09	0.688792048	17.6967
ENSMUSG00000021140	*Pcnx*	ENSMUST00000222005.1	0.00060608	-0.626793897	7.63062
ENSMUSG00000015354	*Pcolce2*	ENSMUST00000015498.8	8.15E-37	-3.509931544	0.258077
ENSMUSG00000038370	*Pcp4l1*	ENSMUST00000111332.1	0.001547474	-2.687666845	0.0636012
ENSMUSG00000044254	*Pcsk9*	ENSMUST00000049507.5	0.005738213	-0.95210122	0.509337
ENSMUSG00000020553	*Pctp*	ENSMUST00000020864.8	0.008694379	0.553926815	3.86523
ENSMUSG00000024892	*Pcx*	ENSMUST00000224726.1	9.49E-11	-0.626881986	27.7073
ENSMUSG00000024620	*Pdgfrb*	ENSMUST00000025522.10	1.49E-05	-0.365144365	83.5942
ENSMUSG00000010914	*Pdhx*	ENSMUST00000011058.8	2.40E-05	-0.472340969	16.0701
ENSMUSG00000022090	*Pdlim2*	ENSMUST00000153735.7	0.004522378	-0.448720771	17.1676
ENSMUSG00000028273	*Pdlim5*	ENSMUST00000029941.15	0.000297939	-0.308135262	41.5207
ENSMUSG00000048371	*Pdp2*	ENSMUST00000059588.7	0.000844392	0.609324059	4.3789
ENSMUSG00000033624	*Pdpr*	ENSMUST00000039333.9	0.004977799	0.482404848	6.98711
ENSMUSG00000027472	*Pdrg1*	ENSMUST00000028972.8	1.01E-06	-0.467831207	79.5922
ENSMUSG00000026784	*Pdss1*	ENSMUST00000053729.13	0.002370875	-0.533831672	5.7249
ENSMUSG00000035357	*Pdzrn3*	ENSMUST00000075994.10	2.96E-05	0.358728238	65.2851
ENSMUSG00000074305	*Peak1*	ENSMUST00000188142.6	6.09E-05	-0.403586696	8.0275
ENSMUSG00000028073	*Pear1*	ENSMUST00000174776.7	2.25E-05	-1.209011856	3.85275
ENSMUSG00000092035	*Peg10*	ENSMUST00000176204.7	0.001702151	-0.719674336	80.3175
ENSMUSG00000020134	*Peli1*	ENSMUST00000093290.11	0.008273596	0.28660169	20.7964
ENSMUSG00000021846	*Peli2*	ENSMUST00000073150.4	0.002845847	-0.348234254	5.7835
ENSMUSG00000042275	*Pelo*	ENSMUST00000109226.4	0.006522033	-0.386292272	37.317
ENSMUSG00000078486	*Perm1*	ENSMUST00000105572.2	0.002715069	-0.526108464	2.59865
ENSMUSG00000019851	*Perp*	ENSMUST00000019998.8	0.0081028	0.869891588	81.5709
ENSMUSG00000069633	*Pex11g*	ENSMUST00000004686.12	0.000828013	0.875707937	2.39368
ENSMUSG00000040374	*Pex2*	ENSMUST00000059021.9	0.009857222	0.322422161	31.5491
ENSMUSG00000073678	*Pgap1*	ENSMUST00000097739.4	6.20E-05	-0.475683567	2.72682
ENSMUSG00000062031	*Pgghg*	ENSMUST00000079403.10	2.19E-07	0.640736493	22.7781
ENSMUSG00000030729	*Pgm2l1*	ENSMUST00000084935.10	0.004876858	0.389617097	3.71408
ENSMUSG00000041731	*Pgm5*	ENSMUST00000047666.4	0.001378802	1.430275473	36.3106
ENSMUSG00000056204	*Pgpep1*	ENSMUST00000070173.8	2.94E-05	0.455987761	15.5124
ENSMUSG00000062866	*Phactr2*	ENSMUST00000105543.8	1.68E-05	0.674000712	12.1429
ENSMUSG00000066043	*Phactr4*	ENSMUST00000084170.11	0.00331838	-0.413674185	12.4506
ENSMUSG00000047777	*Phf13*	ENSMUST00000055688.9	0.007814502	-0.282005333	41.7329
ENSMUSG00000036879	*Phkb*	ENSMUST00000053771.13	8.79E-10	0.681413689	13.9997
ENSMUSG00000029186	*Pi4k2b*	ENSMUST00000031081.10	0.000320597	0.524638682	14.4522

Table S3. RNAseq in D5 in mDPCs-CAS9 cells and sgCreb3l1 _A8_4: 1007 downregulated genes and 1027 upregulated genes

ENSMUSG00000038861	*Pi4kb*	ENSMUST00000072287.11	0.000355509	-0.375626997	22.407
ENSMUSG00000029263	*Pigg*	ENSMUST00000119014.7	0.000801405	0.458539894	9.50516
ENSMUSG00000017721	*Pigt*	ENSMUST00000103101.10	0.000183873	-0.524938904	38.1946
ENSMUSG00000041417	*Pik3r1*	ENSMUST00000055518.12	0.004241597	-0.463601707	29.4251
ENSMUSG00000028698	*Pik3r3*	ENSMUST00000030464.13	1.12E-05	-0.393785162	24.9884
ENSMUSG00000024014	*Pim1*	ENSMUST00000024811.7	3.58E-06	0.55730423	21.874
ENSMUSG00000018547	*Pip4k2b*	ENSMUST00000018691.8	0.001968673	-0.27164645	33.2579
ENSMUSG00000021193	*Pitrm1*	ENSMUST00000021611.9	0.000136255	-0.381642079	31.3705
ENSMUSG00000027499	*Pkia*	ENSMUST00000028999.11	4.11E-16	1.112209869	16.5122
ENSMUSG00000031903	*Pla2g15*	ENSMUST00000034377.7	1.25E-08	0.681343865	24.6714
ENSMUSG00000023913	*Pla2g7*	ENSMUST00000024706.11	1.14E-05	0.996039782	14.5517
ENSMUSG00000072674	*Plac9b*	ENSMUST00000184142.7	0.003215409	-2.426523483	0.216935
ENSMUSG00000031538	*Plat*	ENSMUST00000033941.6	8.60E-05	0.394826593	139.958
ENSMUSG00000024960	*Plcb3*	ENSMUST00000025912.8	0.003016659	-0.263619404	33.7458
ENSMUSG00000039943	*Plcb4*	ENSMUST00000147744.1	0.007610036	-0.321927671	17.8522
ENSMUSG00000038910	*Plcl2*	ENSMUST00000043938.6	2.46E-07	0.580189523	16.2458
ENSMUSG00000040268	*Plekha1*	ENSMUST00000120441.7	0.000517513	0.410707767	20.0306
ENSMUSG00000031557	*Plekha2*	ENSMUST00000128715.7	0.000221667	0.791342808	27.0021
ENSMUSG00000030231	*Plekha5*	ENSMUST00000203483.1	0.00287032	0.507782538	14.6685
ENSMUSG00000041757	*Plekha6*	ENSMUST00000105082.8	0.005993336	0.326911311	10.5004
ENSMUSG00000040852	*Plekhh2*	ENSMUST00000047206.5	1.93E-08	-0.559699017	15.7149
ENSMUSG00000051344	*Plekhm3*	ENSMUST00000097713.1	8.21E-05	0.52276513	4.38136
ENSMUSG00000002831	*Plin4*	ENSMUST00000190703.6	5.87E-07	-1.414543713	0.506077
ENSMUSG00000025758	*Plk4*	ENSMUST00000026858.10	0.000590115	-0.347048313	26.1276
ENSMUSG00000032374	*Plod2*	ENSMUST00000160359.1	0.002342764	-0.334336593	38.4655
ENSMUSG00000031146	*Plp2*	ENSMUST00000033486.5	5.19E-08	0.469929993	275.863
ENSMUSG00000021759	*Plpp1*	ENSMUST00000070951.7	0.003683135	-0.331769069	39.9964
ENSMUSG00000040563	*Plppr2*	ENSMUST00000046371.12	0.000305559	-0.491431152	22.09
ENSMUSG00000027998	*Plrg1*	ENSMUST00000150268.7	3.16E-05	-0.393928364	52.0458
ENSMUSG00000034845	*Plvap*	ENSMUST00000048452.5	1.38E-05	1.580001506	3.14185
ENSMUSG00000017417	*Plxdc1*	ENSMUST00000017561.14	0.005897242	1.015618345	12.3748
ENSMUSG00000030123	*Plxnd1*	ENSMUST00000015511.14	3.28E-08	-0.497328993	16.2182
ENSMUSG00000028066	*Pmf1*	ENSMUST00000056370.12	0.0035814	-0.286262683	102.198
ENSMUSG00000026179	*Pnkd*	ENSMUST00000113805.7	0.001289393	-0.597027003	62.6576
ENSMUSG00000012889	*Podnl1*	ENSMUST00000093380.4	1.98E-05	0.654592745	15.7397
ENSMUSG00000006678	*Pola1*	ENSMUST00000006856.2	0.004843468	0.235953529	62.0947
ENSMUSG00000031536	*Polb*	ENSMUST00000033938.6	8.58E-07	0.63382251	49.14
ENSMUSG00000030726	*Pold3*	ENSMUST00000032969.13	0.00318183	0.33606959	22.9116
ENSMUSG00000020974	*Pole2*	ENSMUST00000021359.6	0.008286638	-0.283136832	31.5506
ENSMUSG00000030042	*Pole4*	ENSMUST00000095786.5	0.000540197	0.307986507	130.719
ENSMUSG00000021668	*Polk*	ENSMUST00000022172.11	1.36E-07	-0.59651046	9.57994
ENSMUSG00000031783	*Polr2c*	ENSMUST00000109521.3	8.43E-07	0.498263109	74.0231
ENSMUSG00000071662	*Polr2g*	ENSMUST00000096261.3	1.98E-05	-0.404049248	138.714
ENSMUSG00000027427	*Polr3f*	ENSMUST00000028914.8	0.001728427	-0.333428397	12.3603
ENSMUSG00000038628	*Polr3k*	ENSMUST00000039551.8	0.000453847	-0.354379611	23.4082
ENSMUSG00000037251	*Pomk*	ENSMUST00000061850.4	1.29E-06	0.592805159	13.9174
ENSMUSG00000029759	*Pon3*	ENSMUST00000031773.8	4.19E-05	0.471076172	86.4268
ENSMUSG00000005514	*Por*	ENSMUST00000005651.12	0.001946187	0.299196296	120.834
ENSMUSG00000027750	*Postn*	ENSMUST00000117373.7	0.004081962	-1.2420218	1055
ENSMUSG00000029676	*Pot1a*	ENSMUST00000115330.7	7.55E-09	0.690037079	25.753
ENSMUSG00000032383	*Ppib*	ENSMUST00000034947.6	0.004243896	-0.246613484	732.815
ENSMUSG00000061130	*Ppm1b*	ENSMUST00000080217.12	0.000259733	-0.433287948	16.2861
ENSMUSG00000040385	*Ppp1ca*	ENSMUST00000046094.4	0.000193013	-0.310318483	252.405
ENSMUSG00000056612	*Ppp1r14b*	ENSMUST00000070850.6	0.000126979	-0.489679404	80.6401
ENSMUSG00000047714	*Ppp1r2*	ENSMUST00000060188.13	0.002507304	0.299896534	62.1488
ENSMUSG00000009630	*Ppp2cb*	ENSMUST00000009774.10	3.95E-07	0.491552084	97.9537
ENSMUSG00000007564	*Ppp2r1a*	ENSMUST00000007708.13	0.00033438	-0.300668521	124.897
ENSMUSG00000024500	*Ppp2r2b*	ENSMUST00000117687.7	0.001318606	-3.297659105	0.180894
ENSMUSG00000026626	*Ppp2r5a*	ENSMUST00000067976.8	2.68E-06	0.422749892	67.0625
ENSMUSG00000017843	*Ppp2r5c*	ENSMUST00000220509.1	0.000367504	-0.563130628	16.4007
ENSMUSG00000059409	*Ppp2r5d*	ENSMUST00000002839.8	0.001533329	-0.399478403	76.1368
ENSMUSG00000028161	*Ppp3ca*	ENSMUST00000070198.13	0.001243597	-0.353005238	41.4188
ENSMUSG00000052144	*Ppp4r2*	ENSMUST00000063854.6	0.007657629	0.223185077	92.269
ENSMUSG00000028657	*Ppt1*	ENSMUST00000030412.10	0.000164086	-0.320093984	92.8046
ENSMUSG00000038582	*Pptc7*	ENSMUST00000053426.14	0.001014	0.352933248	28.5926
ENSMUSG00000050271	*Prag1*	ENSMUST00000110492.1	7.08E-11	0.814593643	11.701
ENSMUSG00000029913	*Prdm5*	ENSMUST00000031976.13	4.97E-05	0.517562669	26.1573
ENSMUSG00000005161	*Prdx2*	ENSMUST00000005292.14	8.97E-05	0.333907801	232.465
ENSMUSG00000024953	*Prdx5*	ENSMUST00000025904.11	2.01E-05	-0.375571797	137.825
ENSMUSG00000021486	*Prelid1*	ENSMUST00000224220.1	0.000320692	-0.320822481	191.293
ENSMUSG00000024127	*Prepl*	ENSMUST00000171795.7	7.06E-05	-0.511998737	9.31543
ENSMUSG00000005469	*Prkaca*	ENSMUST00000005606.7	1.35E-06	0.409640208	158.753

ENSMUSG00000050965	*Prkca*	ENSMUST00000100302.3	5.15E-05	-0.615866559	7.30084
ENSMUSG00000045038	*Prkce*	ENSMUST00000097275.8	0.000104926	-0.449977968	6.48703
ENSMUSG00000003402	*Prkcsh*	ENSMUST00000003493.8	3.59E-05	-0.393100888	85.2711
ENSMUSG00000002688	*Prkd1*	ENSMUST00000002765.8	4.36E-06	0.439083653	35.9473
ENSMUSG00000052920	*Prkg1*	ENSMUST00000065067.12	7.59E-06	-0.66021531	4.78663
ENSMUSG00000002731	*Prkra*	ENSMUST00000002808.6	0.000396107	-0.406232877	26.9617
ENSMUSG00000023110	*Prmt5*	ENSMUST00000023873.11	0.003060017	-0.315996801	20.0096
ENSMUSG00000060098	*Prmt7*	ENSMUST00000071592.11	0.000203374	0.478081267	14.286
ENSMUSG00000037134	*Prmt9*	ENSMUST00000056237.14	0.000184966	0.513480839	12.6052
ENSMUSG00000008373	*Prpf31*	ENSMUST00000143231.1	0.000465099	-0.554144537	26.2253
ENSMUSG00000063800	*Prpf38a*	ENSMUST00000079213.5	0.000771204	-0.324677117	58.8857
ENSMUSG00000035597	*Prpf39*	ENSMUST00000120580.7	0.008220467	0.295396736	15.9302
ENSMUSG00000073565	*Prr16*	ENSMUST00000116639.2	0.000876199	0.332119933	42.1923
ENSMUSG00000045114	*Prrt2*	ENSMUST00000159916.4	0.006821822	-0.72225932	1.08868
ENSMUSG00000045009	*Prrt3*	ENSMUST00000204134.2	0.008901807	-3.349764511	0
ENSMUSG00000022598	*Psca*	ENSMUST00000023265.4	0.00954828	1.539100078	2.98292
ENSMUSG00000030465	*Psd3*	ENSMUST00000120071.7	0.000403067	0.764023062	4.46051
ENSMUSG00000019969	*Psen1*	ENSMUST00000041806.12	0.006390191	-0.254449792	41.7038
ENSMUSG00000048310	*Pskh1*	ENSMUST00000049699.8	6.22E-05	0.480913722	32.6257
ENSMUSG00000015671	*Psma2*	ENSMUST00000170836.3	0.004440519	-0.255306356	53.6045
ENSMUSG00000027566	*Psma7*	ENSMUST00000029082.8	0.000134086	-0.329907734	254.346
ENSMUSG00000014769	*Psmb1*	ENSMUST00000014913.9	0.002021678	-0.260734048	280.125
ENSMUSG00000031897	*Psmb10*	ENSMUST00000034369.9	0.005116924	-0.502900599	16.1464
ENSMUSG00000028837	*Psmb2*	ENSMUST00000030642.2	0.007265538	-0.236236321	261.772
ENSMUSG00000018286	*Psmb6*	ENSMUST00000018430.6	0.002502638	-0.283287213	295.223
ENSMUSG00000026750	*Psmb7*	ENSMUST00000028083.5	0.000253125	-0.310593316	228.521
ENSMUSG00000021178	*Psmc1*	ENSMUST00000021595.9	4.92E-06	-0.392113876	127.832
ENSMUSG00000002102	*Psmc3*	ENSMUST00000067663.13	1.56E-05	-0.419064675	259.519
ENSMUSG00000017428	*Psmd11*	ENSMUST00000173938.7	0.005174204	-0.478058207	28.2412
ENSMUSG00000026914	*Psmd14*	ENSMUST00000028278.13	0.009436862	-0.230605333	101.494
ENSMUSG00000005625	*Psmd4*	ENSMUST00000107237.7	0.003619613	-0.263384149	134.771
ENSMUSG00000039067	*Psmd7*	ENSMUST00000044106.5	9.17E-11	0.557395159	200.679
ENSMUSG00000079197	*Psme2*	ENSMUST00000161807.7	0.000461059	-0.507291737	150.609
ENSMUSG00000021938	*Pspc1*	ENSMUST00000022507.12	1.85E-07	-0.608879325	9.80116
ENSMUSG00000068744	*Psrc1*	ENSMUST00000090561.9	7.75E-08	-1.473808208	9.00836
ENSMUSG00000021518	*Ptdss1*	ENSMUST00000021990.3	0.00777117	-0.246710492	32.9505
ENSMUSG00000037759	*Ptger2*	ENSMUST00000046891.5	0.00434985	0.588786911	2.2308
ENSMUSG00000017969	*Ptgis*	ENSMUST00000088041.10	0.002514397	-0.819366525	3.83026
ENSMUSG00000047250	*Ptgs1*	ENSMUST00000062069.5	3.83E-07	0.445208742	74.1302
ENSMUSG00000026384	*Ptpn4*	ENSMUST00000064091.11	0.002478222	0.402694818	3.46013
ENSMUSG00000032290	*Ptpn9*	ENSMUST00000034832.7	0.002202615	-0.278578053	27.3987
ENSMUSG00000033278	*Ptprm*	ENSMUST00000223982.1	0.004238914	-0.596721684	5.30101
ENSMUSG00000035916	*Ptprq*	ENSMUST00000050702.8	0.000134772	1.717273629	0.712628
ENSMUSG00000009291	*Pttg1ip*	ENSMUST00000009435.11	0.005281024	0.233279118	261.004
ENSMUSG00000027832	*Ptx3*	ENSMUST00000029421.5	6.65E-07	-0.800594289	87.2073
ENSMUSG00000030793	*Pycard*	ENSMUST00000033056.4	0.007657694	-2.258019845	0.0962732
ENSMUSG00000025140	*Pycr1*	ENSMUST00000026133.14	0.001505064	0.878298924	48.7463
ENSMUSG00000021069	*Pygl*	ENSMUST00000071250.12	0.009189999	0.905135051	8.30158
ENSMUSG00000031488	*Rab11fip1*	ENSMUST00000033878.13	6.71E-05	0.855575977	4.96314
ENSMUSG00000040022	*Rab11fip2*	ENSMUST00000171986.7	0.009273262	0.374538	5.88408
ENSMUSG00000051343	*Rab11fip5*	ENSMUST00000060837.9	1.67E-05	0.453431091	23.9882
ENSMUSG00000027935	*Rab13*	ENSMUST00000065418.6	0.00051414	-0.458015953	50.2202
ENSMUSG00000073639	*Rab18*	ENSMUST00000097680.5	0.006029268	0.229961047	157.94
ENSMUSG00000024870	*Rab1b*	ENSMUST00000025804.5	1.77E-05	-0.3901486	108.979
ENSMUSG00000020132	*Rab21*	ENSMUST00000020343.8	0.004230084	0.254769621	38.5409
ENSMUSG00000031202	*Rab39b*	ENSMUST00000033545.5	0.000213938	0.75521461	6.9552
ENSMUSG00000019478	*Rab4a*	ENSMUST00000118535.7	1.45E-05	0.647398724	25.3926
ENSMUSG00000079477	*Rab7*	ENSMUST00000113600.9	0.006152871	0.242134908	602.361
ENSMUSG00000052688	*Rab7b*	ENSMUST00000064664.9	3.16E-05	0.983820254	11.777
ENSMUSG00000035437	*Rabgap1*	ENSMUST00000061179.11	9.55E-05	-0.44893378	19.1865
ENSMUSG00000003813	*Rad23a*	ENSMUST00000128035.1	0.002936618	0.621985679	56.3392
ENSMUSG00000024824	*Rad9a*	ENSMUST00000025740.6	0.00218759	-0.345408013	21.2355
ENSMUSG00000078452	*Raet1d*	ENSMUST00000095795.4	1.07E-05	1.342345148	5.14984
ENSMUSG00000053219	*Raet1e*	ENSMUST00000065527.10	1.50E-25	1.799680932	32.2842
ENSMUSG00000041046	*Ramp3*	ENSMUST00000045374.7	0.000108148	1.150735647	5.41492
ENSMUSG00000037415	*Ranbp10*	ENSMUST00000041400.5	6.17E-08	0.544745432	19.5045
ENSMUSG00000002372	*Ranbp3*	ENSMUST00000002445.8	0.003922605	-0.281444793	76.8274
ENSMUSG00000032892	*Rangrf*	ENSMUST00000038644.4	0.002195101	-0.554410356	13.9771
ENSMUSG00000051615	*Rap2a*	ENSMUST00000062117.12	0.00043393	-0.679581089	5.12501
ENSMUSG00000036894	*Rap2b*	ENSMUST00000049064.3	1.47E-05	-0.474527653	6.93522
ENSMUSG00000026014	*Raph1*	ENSMUST00000140485.7	1.13E-07	0.668774373	7.78665
ENSMUSG00000031453	*Rasa3*	ENSMUST00000117551.3	1.40E-07	0.797617157	46.7632

Table S3. RNAseq in D5 in mDPCs-CAS9 cells and sgCreb3l1 _A8_4: 1007 downregulated genes and 1027 upregulated genes

ENSMUSG00000041696	*Rasl12*	ENSMUST00000085453.5	0.000308006	-1.336212435	0.360016
ENSMUSG00000027339	*Rassf2*	ENSMUST00000103182.7	0.001709113	-0.316396124	18.9994
ENSMUSG00000042129	*Rassf4*	ENSMUST00000035842.6	0.006864621	-1.432500593	0.0801485
ENSMUSG00000030259	*Rassf8*	ENSMUST00000111704.7	5.75E-05	0.36154787	34.9131
ENSMUSG00000031666	*Rbl2*	ENSMUST00000034091.7	5.41E-12	0.701447761	22.1578
ENSMUSG00000010608	*Rbm25*	ENSMUST00000048155.15	0.002536571	-0.264253833	38.9873
ENSMUSG00000029701	*Rbm28*	ENSMUST00000007993.15	0.000383596	0.31200173	53.0809
ENSMUSG00000033931	*Rbm34*	ENSMUST00000212618.1	0.001750031	0.566350553	14.1518
ENSMUSG00000039607	*Rbms3*	ENSMUST00000111773.9	0.002403482	0.335626435	7.51345
ENSMUSG00000031134	*Rbmx*	ENSMUST00000114726.7	5.79E-10	-0.817502257	16.7867
ENSMUSG00000037070	*Rbmxl1*	ENSMUST00000211719.1	1.79E-05	0.478557393	60.3987
ENSMUSG00000024990	*Rbp4*	ENSMUST00000112335.2	0.007048769	-2.267770563	0.126789
ENSMUSG00000039191	*Rbpj*	ENSMUST00000113865.4	0.000123545	0.422792838	89.0209
ENSMUSG00000079575	*Rbpsuh-rs3*	ENSMUST00000182751.1	0.000236063	0.353222619	92.8506
ENSMUSG00000005873	*Reep5*	ENSMUST00000006027.5	1.32E-05	0.376595217	91.5286
ENSMUSG00000022270	*Retreg1*	ENSMUST00000022881.13	2.03E-18	0.911809511	34.0923
ENSMUSG00000058833	*Rex1bd*	ENSMUST00000133683.7	0.002341731	0.334943856	176.885
ENSMUSG00000037962	*Rflna*	ENSMUST00000036109.7	0.004620789	-3.160701837	0.117768
ENSMUSG00000033596	*Rfwd3*	ENSMUST00000038739.4	4.80E-07	0.448738991	43.5132
ENSMUSG00000031706	*Rfx1*	ENSMUST00000005600.5	0.007856487	0.38003996	9.36362
ENSMUSG00000036120	*Rfxank*	ENSMUST00000212320.1	0.008242893	0.608650831	11.9045
ENSMUSG00000022018	*Rgcc*	ENSMUST00000022595.7	0.002008371	-0.937301471	1.80836
ENSMUSG00000040146	*Rgl3*	ENSMUST00000045726.7	0.000416933	-0.671790666	6.58996
ENSMUSG00000022075	*Rhobtb2*	ENSMUST00000022665.3	0.002475827	-0.457900862	2.96457
ENSMUSG00000046768	*Rhoj*	ENSMUST00000055390.5	0.004284115	-0.408468974	28.6346
ENSMUSG00000039960	*Rhou*	ENSMUST00000045487.3	2.52E-15	0.911016888	21.6537
ENSMUSG00000021408	*Ripk1*	ENSMUST00000021844.14	0.007792665	-0.25508885	28.7986
ENSMUSG00000022221	*Ripk3*	ENSMUST00000022830.12	1.72E-05	-0.4089313	51.7365
ENSMUSG00000038604	*Ripor1*	ENSMUST00000043531.9	0.000273922	0.388316444	31.3956
ENSMUSG00000049878	*Rlf*	ENSMUST00000056635.12	0.000754406	-0.319739208	13.971
ENSMUSG00000070730	*Rmdn3*	ENSMUST00000094695.11	0.009193477	-0.281337148	28
ENSMUSG00000002222	*Rmnd5a*	ENSMUST00000002292.14	6.33E-05	0.35933266	35.6263
ENSMUSG00000095687	*Rnaset2a*	ENSMUST00000097420.5	0.006181238	-0.487721778	305.328
ENSMUSG00000054855	*Rnd1*	ENSMUST00000003451.10	7.93E-08	0.973139309	7.448
ENSMUSG00000017144	*Rnd3*	ENSMUST00000017288.8	4.28E-15	-0.655576252	120.736
ENSMUSG00000006418	*Rnf114*	ENSMUST00000078050.6	0.001945789	-0.333573779	29.9562
ENSMUSG00000031438	*Rnf128*	ENSMUST00000113026.1	0.005368901	-0.746666194	1.60379
ENSMUSG00000020376	*Rnf130*	ENSMUST00000102776.4	4.52E-09	1.039628614	25.6636
ENSMUSG00000020707	*Rnf135*	ENSMUST00000017839.2	5.88E-06	-0.645902115	8.65904
ENSMUSG00000058761	*Rnf169*	ENSMUST00000080817.5	0.000775599	-0.386210463	5.32572
ENSMUSG00000013878	*Rnf170*	ENSMUST00000014022.14	4.87E-09	0.756405345	10.892
ENSMUSG00000063760	*Rnf217*	ENSMUST00000081989.7	0.00909208	0.278847891	6.58449
ENSMUSG00000048911	*Rnf24*	ENSMUST00000059372.10	0.000569562	-0.669807208	18.3327
ENSMUSG00000090083	*Rnf8*	ENSMUST00000024817.13	0.008899261	-0.332677492	19.2456
ENSMUSG00000028274	*Rngtt*	ENSMUST00000108153.8	0.001933913	0.353736103	24.2496
ENSMUSG00000038650	*Rnh1*	ENSMUST00000106033.4	2.39E-08	0.518475224	100.283
ENSMUSG00000026269	*Rnpepl1*	ENSMUST00000027487.14	0.003672921	0.446216559	16.1517
ENSMUSG00000022883	*Robo1*	ENSMUST00000023600.7	0.001187842	-0.567041145	5.84905
ENSMUSG00000071648	*Rom1*	ENSMUST00000096242.3	0.000210182	-0.719626384	4.76654
ENSMUSG00000067847	*Romo1*	ENSMUST00000088610.10	0.003897519	-0.398447966	138.797
ENSMUSG00000036192	*Rorb*	ENSMUST00000112832.7	0.001919594	2.630762032	0.282766
ENSMUSG00000033282	*Rpgrip1l*	ENSMUST00000047783.13	4.12E-13	0.765597676	15.9419
ENSMUSG00000059291	*Rpl11*	ENSMUST00000102536.10	0.000982706	-0.283953736	460.531
ENSMUSG00000045128	*Rpl18a*	ENSMUST00000212494.1	6.50E-07	0.452498854	866.937
ENSMUSG00000098274	*Rpl24*	ENSMUST00000023269.4	0.004393043	-0.237655388	832.825
ENSMUSG00000062997	*Rpl35*	ENSMUST00000080861.5	0.001117045	-0.272771846	481.342
ENSMUSG00000057863	*Rpl36*	ENSMUST00000080492.5	0.000185256	-0.436526728	1131.74
ENSMUSG00000079435	*Rpl36a*	ENSMUST00000113211.7	0.000187053	-0.330533687	688.148
ENSMUSG00000049751	*Rpl36al*	ENSMUST00000054544.6	2.82E-05	-0.374738437	454.976
ENSMUSG00000093674	*Rpl41*	ENSMUST00000176010.7	6.93E-05	-0.337910153	1857.78
ENSMUSG00000062647	*Rpl7a*	ENSMUST00000102898.4	7.97E-06	-0.379531611	843.171
ENSMUSG00000063888	*Rpl7l1*	ENSMUST00000078286.5	1.80E-05	-0.375742057	57.9285
ENSMUSG00000024800	*Rpp30*	ENSMUST00000025714.7	0.00044293	-0.405455761	40.5197
ENSMUSG00000091957	*Rps2-ps10*	ENSMUST00000170335.3	0.000219868	-0.340045436	204.35
ENSMUSG00000095427	*Rps2-ps6*	ENSMUST00000095471.4	0.001562192	-0.400269073	80.2395
ENSMUSG00000034892	*Rps29*	ENSMUST00000037023.8	1.36E-06	-0.414492678	1385.03
ENSMUSG00000063171	*Rps4l*	ENSMUST00000071745.3	5.72E-15	-5.079393276	0
ENSMUSG00000031880	*Rrad*	ENSMUST00000034351.7	1.10E-09	1.093241035	14.1195
ENSMUSG00000055723	*Rras2*	ENSMUST00000069449.6	0.00122608	0.460106256	15.0973
ENSMUSG00000022292	*Rrm2b*	ENSMUST00000022901.15	3.02E-05	0.477958776	12.4137
ENSMUSG00000035049	*Rrp12*	ENSMUST00000038677.3	0.003211799	-0.372979925	6.80545
ENSMUSG00000023971	*Rrp36*	ENSMUST00000024766.6	0.003877304	-0.344457542	39.4717

ENSMUSG00000028871	*Rspo1*	ENSMUST00000030687.7	2.01E-07	1.804769214	8.81497
ENSMUSG00000051920	*Rspo2*	ENSMUST00000063492.6	0.009864907	0.407333262	73.4527
ENSMUSG00000050079	*Rspry1*	ENSMUST00000060389.9	0.004824413	0.319436935	21.4241
ENSMUSG00000027502	*Rtfdc1*	ENSMUST00000029005.3	0.009725781	-0.252350302	42.2901
ENSMUSG00000019864	*Rtn4ip1*	ENSMUST00000054418.11	0.004940647	0.461302341	6.32085
ENSMUSG00000045287	*Rtn4rl1*	ENSMUST00000102514.3	3.21E-07	-2.266156761	0.127177
ENSMUSG00000050896	*Rtn4rl2*	ENSMUST00000151799.7	2.55E-05	0.873716925	10.6569
ENSMUSG00000020070	*Rufy2*	ENSMUST00000119567.7	0.000687561	0.380104282	14.6353
ENSMUSG00000031568	*Rwdd4a*	ENSMUST00000033973.13	9.81E-06	0.485898087	32.075
ENSMUSG00000015846	*Rxra*	ENSMUST00000077257.11	0.002596369	-0.261961429	42.678
ENSMUSG00000041959	*S100a10*	ENSMUST00000045756.13	0.000315611	-0.483834993	780.888
ENSMUSG00000074457	*S100a16*	ENSMUST00000098911.9	0.003657949	0.780612147	35.4286
ENSMUSG00000043895	*S1pr2*	ENSMUST00000054197.6	0.00038927	-0.334755575	14.3024
ENSMUSG00000067586	*S1pr3*	ENSMUST00000087978.4	3.20E-15	-1.645084866	2.50615
ENSMUSG00000025240	*Sacm1l*	ENSMUST00000026270.8	0.004432172	0.254790216	43.2067
ENSMUSG00000049532	*Sall2*	ENSMUST00000135523.4	0.005832748	-0.300813471	10.1415
ENSMUSG00000079003	*Samd1*	ENSMUST00000210523.1	1.55E-07	0.546176283	85.3582
ENSMUSG00000031532	*Saraf*	ENSMUST00000033933.6	8.31E-05	0.49656198	116.418
ENSMUSG00000009614	*Sardh*	ENSMUST00000102886.9	4.79E-05	-0.37717872	34.6705
ENSMUSG00000038331	*Satb2*	ENSMUST00000114415.9	3.66E-05	-0.463417314	10.034
ENSMUSG00000045107	*Saysd1*	ENSMUST00000059666.5	0.009885315	0.450402935	6.61121
ENSMUSG00000046056	*Sbsn*	ENSMUST00000182229.7	0.002387969	2.045088025	7.52482
ENSMUSG00000034463	*Scara3*	ENSMUST00000042046.4	6.68E-31	-3.137554367	0.298603
ENSMUSG00000002565	*Scin*	ENSMUST00000002640.5	0.005913399	2.404596404	12.9897
ENSMUSG00000026307	*Scly*	ENSMUST00000027532.12	1.41E-05	0.52442327	24.1806
ENSMUSG00000008226	*Scrn3*	ENSMUST00000090811.10	0.000566633	-0.471269893	6.63886
ENSMUSG00000016763	*Scube1*	ENSMUST00000016907.7	0.00124917	-0.738340308	1.86662
ENSMUSG00000038677	*Scube3*	ENSMUST00000043503.9	0.001148608	-1.343086923	0.407402
ENSMUSG00000021577	*Sdha*	ENSMUST00000022062.7	5.47E-06	-0.385903758	73.5218
ENSMUSG00000009863	*Sdhb*	ENSMUST00000010007.8	6.57E-05	-0.367793758	124.478
ENSMUSG00000039683	*Sdk1*	ENSMUST00000074546.6	6.43E-09	-0.650314966	6.76405
ENSMUSG00000079614	*Seh1l*	ENSMUST00000025421.7	0.003365141	0.2515055	67.7189
ENSMUSG00000020964	*Sel1l*	ENSMUST00000021347.11	0.008979275	-0.330528087	19.1539
ENSMUSG00000075703	*Selenoi*	ENSMUST00000132404.7	1.90E-05	-0.699636392	4.22409
ENSMUSG00000050989	*Selenon*	ENSMUST00000060435.6	0.002492576	-0.269475196	70.1347
ENSMUSG00000057969	*Sema3b*	ENSMUST00000102529.9	0.001465949	-0.611889591	8.83139
ENSMUSG00000028780	*Sema3c*	ENSMUST00000030568.13	0.000228803	0.466776486	27.1972
ENSMUSG00000063531	*Sema3e*	ENSMUST00000073957.7	0.001639002	0.675992846	2.41481
ENSMUSG00000034684	*Sema3f*	ENSMUST00000192727.5	0.000256645	0.575582433	21.0222
ENSMUSG00000021904	*Sema3g*	ENSMUST00000090180.2	0.000235276	0.913887155	1.43456
ENSMUSG00000000627	*Sema4f*	ENSMUST00000000641.14	0.001619659	1.168714411	10.8396
ENSMUSG00000038264	*Sema7a*	ENSMUST00000043059.8	0.008481371	-0.840876062	1.3952
ENSMUSG00000058013	*Sept11*	ENSMUST00000074733.10	5.59E-05	-0.342410448	119.11
ENSMUSG00000022456	*Sept3*	ENSMUST00000023095.12	5.22E-05	0.709997833	4.38702
ENSMUSG00000050379	*Sept6*	ENSMUST00000115239.9	0.000933766	-0.429763893	18.1802
ENSMUSG00000001833	*Sept7*	ENSMUST00000165594.3	0.002656862	-0.254398905	239.613
ENSMUSG00000036371	*Serbp1*	ENSMUST00000204293.2	0.00083089	0.556334972	595.587
ENSMUSG00000021643	*Serf1*	ENSMUST00000022145.14	0.002052008	-0.468739626	70.8771
ENSMUSG00000058586	*Serhl*	ENSMUST00000078218.10	0.006520165	0.304038366	53.3077
ENSMUSG00000021403	*Serpinb9b*	ENSMUST00000006392.2	0.009550592	1.441405505	2.05436
ENSMUSG00000032009	*Sesn3*	ENSMUST00000208222.1	4.30E-05	-0.372105771	35.5069
ENSMUSG00000024548	*Setbp1*	ENSMUST00000025430.10	0.001156928	-0.299258953	9.7811
ENSMUSG00000028902	*Sf3a3*	ENSMUST00000030734.4	0.006613009	-0.240035756	95.645
ENSMUSG00000024853	*Sf3b2*	ENSMUST00000025774.9	0.002484468	-0.252218675	125.187
ENSMUSG00000033732	*Sf3b3*	ENSMUST00000042012.6	1.27E-06	0.404729293	98.9382
ENSMUSG00000027996	*Sfrp2*	ENSMUST00000029625.7	0.009121273	-0.483877619	222.538
ENSMUSG00000040848	*Sft2d2*	ENSMUST00000043338.9	2.68E-09	0.562139008	28.1911
ENSMUSG00000021474	*Sfxn1*	ENSMUST00000021930.9	4.27E-05	-0.409764033	23.476
ENSMUSG00000020354	*Sgcd*	ENSMUST00000077221.5	0.002971292	0.325291895	45.0827
ENSMUSG00000004631	*Sgce*	ENSMUST00000115579.7	7.76E-07	0.436825827	164.824
ENSMUSG00000021054	*Sgpp1*	ENSMUST00000021450.5	8.47E-05	0.371058578	36.0973
ENSMUSG00000042216	*Sgsm1*	ENSMUST00000057209.11	4.78E-06	0.690333352	6.16154
ENSMUSG00000042743	*Sgtb*	ENSMUST00000044385.13	0.001859751	-0.404637085	8.64003
ENSMUSG00000059013	*Sh2d3c*	ENSMUST00000074248.10	0.007267824	-2.012912545	0.234026
ENSMUSG00000032261	*Sh3bgrl2*	ENSMUST00000113215.9	2.00E-08	0.882094437	6.00096
ENSMUSG00000028843	*Sh3bgrl3*	ENSMUST00000030651.8	0.000822124	-0.341126689	196.896
ENSMUSG00000037062	*Sh3glb1*	ENSMUST00000199854.4	7.69E-05	-0.616529821	33.9696
ENSMUSG00000031642	*Sh3rf1*	ENSMUST00000034060.6	0.000155339	0.468722549	9.10772
ENSMUSG00000042626	*Shc1*	ENSMUST00000094378.9	3.69E-11	-0.617887265	41.6303
ENSMUSG00000020312	*Shc2*	ENSMUST00000020564.6	0.000193978	0.718243359	18.5159
ENSMUSG00000022322	*Shcbp1*	ENSMUST00000022945.8	0.000127642	0.415944948	31.0111
ENSMUSG00000027833	*Shox2*	ENSMUST00000162098.8	0.008138468	-0.228196804	106.749

ENSMUSG00000036840	*Siah1a*	ENSMUST00000045296.5	7.04E-07	0.629969779	23.1201
ENSMUSG00000034908	*Sidt2*	ENSMUST00000038488.16	0.001346258	-0.312276128	30.0438
ENSMUSG00000030468	*Siglecg*	ENSMUST00000005592.6	0.002670966	1.348002851	2.88613
ENSMUSG00000036078	*Sigmar1*	ENSMUST00000059354.14	0.006000594	-0.262640865	77.0481
ENSMUSG00000034135	*Sik3*	ENSMUST00000122865.1	0.000887248	-0.417720044	21.32
ENSMUSG00000062713	*Sim2*	ENSMUST00000072182.7	4.13E-29	2.903761653	6.59828
ENSMUSG00000043183	*Simc1*	ENSMUST00000121401.7	0.007723992	-0.339815063	5.87911
ENSMUSG00000042557	*Sin3a*	ENSMUST00000167715.7	0.006655589	-0.552835664	14.1076
ENSMUSG00000031622	*Sin3b*	ENSMUST00000109950.4	0.002420651	0.318094516	108.785
ENSMUSG00000042700	*Sipa1l1*	ENSMUST00000222714.1	0.001176835	-0.393872737	11.825
ENSMUSG00000001995	*Sipa1l2*	ENSMUST00000212168.1	5.62E-06	0.486231504	11.4265
ENSMUSG00000020063	*Sirt1*	ENSMUST00000020257.12	0.001596007	0.407995316	13.3576
ENSMUSG00000051367	*Six1*	ENSMUST00000050029.7	1.92E-07	-0.434599072	121.574
ENSMUSG00000024134	*Six2*	ENSMUST00000163568.3	0.008436897	-0.738602665	58.0945
ENSMUSG00000034460	*Six4*	ENSMUST00000043208.7	0.003563894	-0.366528971	4.72875
ENSMUSG00000016018	*Skiv2l2*	ENSMUST00000022281.4	2.37E-05	-0.374162197	35.999
ENSMUSG00000031684	*Slc10a7*	ENSMUST00000034111.9	8.06E-07	0.69565146	12.406
ENSMUSG00000017765	*Slc12a4*	ENSMUST00000116429.8	2.20E-09	0.702057458	41.2842
ENSMUSG00000029416	*Slc15a4*	ENSMUST00000031367.14	0.001903078	0.373300092	24.6141
ENSMUSG00000032902	*Slc16a1*	ENSMUST00000046212.1	0.005278217	0.700433067	37.4299
ENSMUSG00000019838	*Slc16a10*	ENSMUST00000092566.7	0.000537149	0.468301042	6.29809
ENSMUSG00000049624	*Slc17a5*	ENSMUST00000052441.11	0.008681009	0.314807432	15.3439
ENSMUSG00000023393	*Slc17a9*	ENSMUST00000094218.3	7.36E-05	1.08329996	2.49671
ENSMUSG00000037656	*Slc20a2*	ENSMUST00000067786.8	1.83E-12	0.704625889	31.4045
ENSMUSG00000063873	*Slc24a3*	ENSMUST00000137908.1	0.001406507	-0.38547236	15.2962
ENSMUSG00000031482	*Slc25a15*	ENSMUST00000033871.7	0.005382001	0.386478285	9.59003
ENSMUSG00000032519	*Slc25a38*	ENSMUST00000035106.11	0.001085125	-0.601033713	4.87139
ENSMUSG00000031633	*Slc25a4*	ENSMUST00000034049.4	7.96E-14	0.634491695	314.979
ENSMUSG00000040569	*Slc26a7*	ENSMUST00000042221.13	1.54E-11	-2.300403106	4.20923
ENSMUSG00000027932	*Slc27a3*	ENSMUST00000029541.11	3.84E-05	-0.687966098	32.6926
ENSMUSG00000036298	*Slc2a13*	ENSMUST00000109283.1	0.000219354	-1.064159378	0.237525
ENSMUSG00000028293	*Slc35a1*	ENSMUST00000029970.13	0.00052507	-0.398895691	19.1165
ENSMUSG00000027957	*Slc35a3*	ENSMUST00000029569.8	0.000128903	0.382824269	17.2079
ENSMUSG00000019731	*Slc35e1*	ENSMUST00000152080.7	6.51E-11	0.600669595	39.9265
ENSMUSG00000043885	*Slc36a4*	ENSMUST00000061568.8	0.003297281	-0.280122417	14.0698
ENSMUSG00000022464	*Slc38a4*	ENSMUST00000023101.9	2.80E-07	0.465831734	124.14
ENSMUSG00000036534	*Slc38a7*	ENSMUST00000212270.1	4.43E-08	0.591690056	27.7859
ENSMUSG00000013275	*Slc41a1*	ENSMUST00000086559.6	6.72E-05	0.3821378	24.5068
ENSMUSG00000030089	*Slc41a3*	ENSMUST00000044019.15	2.13E-05	0.445757582	39.3246
ENSMUSG00000057193	*Slc44a2*	ENSMUST00000217461.1	0.004351522	-0.251769925	51.8391
ENSMUSG00000060961	*Slc4a4*	ENSMUST00000148750.7	1.24E-05	0.810821697	2.12925
ENSMUSG00000021733	*Slc4a7*	ENSMUST00000057015.7	0.002221718	0.328406271	13.2032
ENSMUSG00000023032	*Slc4a8*	ENSMUST00000162049.1	0.000838971	0.798734692	2.11311
ENSMUSG00000027737	*Slc7a11*	ENSMUST00000029297.5	9.17E-05	0.854619349	3.04372
ENSMUSG00000031297	*Slc7a3*	ENSMUST00000113710.7	0.001070922	0.728620798	4.69795
ENSMUSG00000031904	*Slc7a6*	ENSMUST00000034378.4	4.56E-07	0.650160649	15.8609
ENSMUSG00000033106	*Slc7a6os*	ENSMUST00000035925.6	0.001758064	0.375177398	47.5037
ENSMUSG00000014786	*Slc9a5*	ENSMUST00000212772.1	1.35E-05	0.661243822	7.04108
ENSMUSG00000032548	*Slco2a1*	ENSMUST00000035148.12	0.000146335	-0.86282403	3.21716
ENSMUSG00000031681	*Smad1*	ENSMUST00000066091.13	1.06E-05	0.48627157	25.5174
ENSMUSG00000032402	*Smad3*	ENSMUST00000034973.9	7.97E-08	-0.637279424	25.2622
ENSMUSG00000032870	*Smap2*	ENSMUST00000043200.7	0.007566727	-0.271002983	34.4899
ENSMUSG00000024921	*Smarca2*	ENSMUST00000208674.1	0.00828841	-0.840606683	11.6776
ENSMUSG00000032187	*Smarca4*	ENSMUST00000174008.7	0.003251019	-0.354384411	29.9588
ENSMUSG00000031715	*Smarca5*	ENSMUST00000043359.8	5.85E-13	0.61954545	59.6065
ENSMUSG00000024943	*Smc5*	ENSMUST00000223934.1	0.00901676	-0.265785207	21.2751
ENSMUSG00000020608	*Smc6*	ENSMUST00000020931.5	4.24E-05	0.359696224	70.2507
ENSMUSG00000001415	*Smg5*	ENSMUST00000001451.10	0.005013189	-0.240306721	48.6188
ENSMUSG00000031534	*Smim19*	ENSMUST00000033935.15	1.10E-05	0.684934108	23.5369
ENSMUSG00000058351	*Smim4*	ENSMUST00000090205.3	0.000329339	-1.04369244	15.027
ENSMUSG00000021645	*Smn1*	ENSMUST00000022147.14	0.000100208	-0.45275546	38.8989
ENSMUSG00000028885	*Smpdl3b*	ENSMUST00000030709.8	9.00E-12	1.117186462	26.388
ENSMUSG00000042821	*Snai1*	ENSMUST00000052631.7	0.000444342	-0.45161326	33.3418
ENSMUSG00000022676	*Snai2*	ENSMUST00000023356.6	2.16E-05	-0.488430372	76.1122
ENSMUSG00000036281	*Snapc4*	ENSMUST00000035427.10	0.009677491	-0.329885161	6.97623
ENSMUSG00000001424	*Snd1*	ENSMUST00000001460.13	0.001294446	0.268956345	182.804
ENSMUSG00000047793	*Sned1*	ENSMUST00000062202.13	2.19E-05	-1.226246913	13.4024
ENSMUSG00000037972	*Snn*	ENSMUST00000089011.4	5.72E-09	-0.675280635	20.5854
ENSMUSG00000001158	*Snrnp27*	ENSMUST00000001186.10	0.004185997	0.258090151	249.58
ENSMUSG00000074088	*Snrnp40*	ENSMUST00000105994.3	0.002214397	-0.326857161	36.2131
ENSMUSG00000008333	*Snrpb2*	ENSMUST00000127760.7	0.00886979	-0.437940846	45.1582
ENSMUSG00000024217	*Snrpc*	ENSMUST00000071006.7	0.000196821	-0.350476421	204.729

ENSMUSG00000060429	*Sntb1*	ENSMUST00000039769.11	0.0095998	0.406816297	20.0898
ENSMUSG00000041308	*Sntb2*	ENSMUST00000212524.1	2.52E-08	0.483693565	24.9392
ENSMUSG00000038301	*Snx10*	ENSMUST00000049152.14	2.19E-07	0.698098019	31.7591
ENSMUSG00000020590	*Snx13*	ENSMUST00000048519.16	0.003668547	0.268879355	27.8245
ENSMUSG00000024535	*Snx24*	ENSMUST00000165032.7	0.002655124	0.427202677	16.7074
ENSMUSG00000038291	*Snx25*	ENSMUST00000170416.7	6.74E-12	0.864164782	24.5452
ENSMUSG00000037104	*Socs5*	ENSMUST00000041369.6	0.007253462	-0.37714428	25.3937
ENSMUSG00000055485	*Soga1*	ENSMUST00000069098.6	0.00347306	-0.684830844	13.5525
ENSMUSG00000031626	*Sorbs2*	ENSMUST00000139869.7	2.97E-09	0.796591903	14.782
ENSMUSG00000022091	*Sorbs3*	ENSMUST00000022682.5	4.28E-10	-0.665617619	16.1678
ENSMUSG00000027227	*Sord*	ENSMUST00000110551.3	1.01E-10	-0.544957563	101.95
ENSMUSG00000068747	*Sort1*	ENSMUST00000102632.6	0.005598763	-0.905698818	1.4539
ENSMUSG00000000567	*Sox9*	ENSMUST00000000579.2	0.000422693	-0.922311059	3.72568
ENSMUSG00000070031	*Sp140*	ENSMUST00000080204.10	0.001306789	2.015315293	1.61844
ENSMUSG00000025323	*Sp4*	ENSMUST00000222314.1	0.003148788	-0.847182183	1.10689
ENSMUSG00000060284	*Sp7*	ENSMUST00000078508.5	0.001682408	-0.927180451	0.628565
ENSMUSG00000029309	*Sparcl1*	ENSMUST00000031249.7	7.12E-05	-1.044511414	15.9464
ENSMUSG00000020191	*Spata48*	ENSMUST00000020410.10	0.00876235	1.799092647	1.50617
ENSMUSG00000034401	*Spata6*	ENSMUST00000038868.13	0.009414921	-0.283492382	25.1613
ENSMUSG00000054408	*Spcs3*	ENSMUST00000067476.8	2.80E-06	0.600592545	56.2944
ENSMUSG00000027329	*Spef1*	ENSMUST00000110218.8	0.002361362	0.465610959	8.92477
ENSMUSG00000021395	*Spin1*	ENSMUST00000095797.5	0.000945629	-0.2857939	41.1334
ENSMUSG00000051457	*Spn*	ENSMUST00000049931.5	0.001877894	0.577884605	11.9796
ENSMUSG00000040447	*Spns2*	ENSMUST00000045303.9	0.003460642	-1.881539652	0.146076
ENSMUSG00000037379	*Spon2*	ENSMUST00000046186.7	0.000125089	-0.996579841	20.3821
ENSMUSG00000039660	*Spout1*	ENSMUST00000100220.4	0.000324436	-0.457783417	20.2042
ENSMUSG00000027366	*Sppl2a*	ENSMUST00000028844.10	0.005910872	-0.240220704	34.4324
ENSMUSG00000031986	*Sprtn*	ENSMUST00000034467.6	2.77E-06	0.553342597	13.3154
ENSMUSG00000061654	*Spry3*	ENSMUST00000076951.3	0.00032229	1.043552282	3.00235
ENSMUSG00000024427	*Spry4*	ENSMUST00000025295.6	2.52E-07	-1.123953619	0.785786
ENSMUSG00000057738	*Sptan1*	ENSMUST00000046257.13	0.002294032	-0.267116643	66.196
ENSMUSG00000022463	*Srebf2*	ENSMUST00000023100.6	0.009792982	0.222292613	178.716
ENSMUSG00000090084	*Srpx*	ENSMUST00000115544.8	1.47E-13	-0.922038266	28.5726
ENSMUSG00000037013	*Ss18*	ENSMUST00000092041.9	0.009906497	0.257585078	49.1419
ENSMUSG00000039086	*Ss18l1*	ENSMUST00000041126.8	0.001055625	-0.584716495	3.2709
ENSMUSG00000061887	*Ssbp3*	ENSMUST00000072753.12	0.005593946	-0.248033106	94.2302
ENSMUSG00000070003	*Ssbp4*	ENSMUST00000049908.10	1.70E-08	0.700128388	52.2311
ENSMUSG00000030255	*Sspn*	ENSMUST00000032383.13	8.86E-05	0.369958017	31.4788
ENSMUSG00000021427	*Ssr1*	ENSMUST00000225246.1	0.003950667	-0.27875014	54.1582
ENSMUSG00000041355	*Ssr2*	ENSMUST00000195014.5	6.88E-06	-0.452275172	485.118
ENSMUSG00000079478	*Sssca1*	ENSMUST00000025885.4	0.000374265	-0.602459861	23.1642
ENSMUSG00000031749	*St3gal2*	ENSMUST00000034197.4	4.59E-12	0.596738544	78.2571
ENSMUSG00000025862	*Stag2*	ENSMUST00000069619.13	0.001779779	0.266691701	61.3924
ENSMUSG00000055371	*Stam2*	ENSMUST00000102759.7	0.005073143	-0.271938108	16.4832
ENSMUSG00000031574	*Star*	ENSMUST00000033979.5	0.00268936	0.963567399	0.789041
ENSMUSG00000027367	*Stard7*	ENSMUST00000110375.8	0.001992248	-0.281834337	43.951
ENSMUSG00000025920	*Stau2*	ENSMUST00000162007.7	0.000723378	0.453070812	12.5432
ENSMUSG00000020272	*Stk10*	ENSMUST00000102821.3	0.000388016	0.364458487	17.4844
ENSMUSG00000022329	*Stk3*	ENSMUST00000018476.13	2.00E-05	0.367543813	101.743
ENSMUSG00000037885	*Stk35*	ENSMUST00000166282.2	0.002476182	-0.308233442	11.8467
ENSMUSG00000042608	*Stk40*	ENSMUST00000094761.10	3.26E-09	-0.623896109	14.9495
ENSMUSG00000026880	*Stom*	ENSMUST00000028241.6	0.001608422	-0.455446031	50.273
ENSMUSG00000028455	*Stoml2*	ENSMUST00000030169.14	0.001362568	-0.335082903	54.7345
ENSMUSG00000033855	*Ston1*	ENSMUST00000163588.7	1.99E-06	-0.451983681	33.1876
ENSMUSG00000014601	*Strip1*	ENSMUST00000064759.6	0.005621051	-0.333360904	32.7661
ENSMUSG00000030806	*Stx1b*	ENSMUST00000106267.4	0.000240927	1.141197593	1.15838
ENSMUSG00000041488	*Stx3*	ENSMUST00000069285.5	0.008817283	0.392887186	8.26364
ENSMUSG00000022110	*Sucla2*	ENSMUST00000022706.6	0.003617158	-0.325318212	41.1316
ENSMUSG00000011306	*Sugp1*	ENSMUST00000011450.7	0.002229423	0.344495808	25.3561
ENSMUSG00000016918	*Sulf1*	ENSMUST00000177608.7	0.002485241	-0.875070882	2.6151
ENSMUSG00000030711	*Sult1a1*	ENSMUST00000106373.7	0.008660119	-2.183424192	0.653862
ENSMUSG00000027751	*Supt20*	ENSMUST00000197502.4	1.54E-05	-0.592978137	9.89309
ENSMUSG00000036160	*Surf6*	ENSMUST00000047632.13	0.004500325	-0.319513505	16.0748
ENSMUSG00000038486	*Sv2a*	ENSMUST00000035371.8	0.003789837	0.459810966	4.20517
ENSMUSG00000028643	*Svbp*	ENSMUST00000030395.8	0.005840891	-0.433123487	137.739
ENSMUSG00000074093	*Svip*	ENSMUST00000098414.4	0.000335372	3.324831444	3.06559
ENSMUSG00000051238	*Swsap1*	ENSMUST00000053583.6	0.001043317	-0.512064049	8.41985
ENSMUSG00000022340	*Sybu*	ENSMUST00000110267.7	2.62E-06	0.78610641	9.14138
ENSMUSG00000054150	*Syne3*	ENSMUST00000067005.10	5.55E-06	-0.46068922	12.2931
ENSMUSG00000022415	*Syngr1*	ENSMUST00000009728.12	0.001669451	1.089361196	8.10179
ENSMUSG00000048277	*Syngr2*	ENSMUST00000026649.13	0.005122911	0.335841774	31.5441
ENSMUSG00000020570	*Sypl*	ENSMUST00000020885.12	0.001935087	0.285783724	82.7789

Table S3. RNAseq in D5 in mDPCs-CAS9 cells and sgCreb3l1 _A8_4: 1007 downregulated genes and 1027 upregulated genes

ENSMUSG00000028860	*Sytl1*	ENSMUST00000030674.7	0.00363344	-1.954403858	0.361325
ENSMUSG00000015755	*Tab2*	ENSMUST00000146444.7	0.003122227	0.274123625	113.262
ENSMUSG00000061762	*Tac1*	ENSMUST00000184986.1	2.17E-06	2.431566428	12.293
ENSMUSG00000031314	*Taf1*	ENSMUST00000118878.7	0.003104087	0.281929058	34.4979
ENSMUSG00000054321	*Taf4b*	ENSMUST00000169862.1	0.005450544	0.501387445	2.50918
ENSMUSG00000038697	*Taf5l*	ENSMUST00000165628.8	1.68E-05	0.632339764	23.0327
ENSMUSG00000041949	*Tango6*	ENSMUST00000048359.4	0.000622081	0.48112149	7.08343
ENSMUSG00000024308	*Tapbp*	ENSMUST00000025161.8	1.03E-07	-0.487282778	184.039
ENSMUSG00000090290	*Tarbp1*	ENSMUST00000170518.2	6.60E-05	0.575538071	4.20677
ENSMUSG00000039678	*Tbc1d13*	ENSMUST00000044556.11	0.009039519	-0.253227012	23.2615
ENSMUSG00000037410	*Tbc1d2b*	ENSMUST00000041767.13	0.002286302	0.358413006	25.5911
ENSMUSG00000022364	*Tbc1d31*	ENSMUST00000022992.12	0.00612252	0.258386828	43.8986
ENSMUSG00000031709	*Tbc1d9*	ENSMUST00000093393.4	2.35E-06	1.285195628	8.69541
ENSMUSG00000027868	*Tbx15*	ENSMUST00000029462.9	1.02E-08	-0.598028436	20.5718
ENSMUSG00000051579	*Tceal8*	ENSMUST00000060101.9	0.000427707	-0.303286779	172.913
ENSMUSG00000041852	*Tcf20*	ENSMUST00000048966.5	0.002779152	0.25881378	37.0674
ENSMUSG00000001472	*Tcf25*	ENSMUST00000212571.1	3.73E-05	0.623101709	83.2593
ENSMUSG00000053477	*Tcf4*	ENSMUST00000078486.12	2.59E-05	0.361588756	44.1685
ENSMUSG00000038932	*Tcfl5*	ENSMUST00000037877.10	4.90E-05	-2.1504351	0.529918
ENSMUSG00000001750	*Tcirg1*	ENSMUST00000001801.10	0.007000782	-0.259477055	40.0959
ENSMUSG00000068039	*Tcp1*	ENSMUST00000089024.12	0.0024525	-0.276932468	101.841
ENSMUSG00000055320	*Tead1*	ENSMUST00000165036.7	0.002706628	-0.560332213	6.04942
ENSMUSG00000031708	*Tecr*	ENSMUST00000019382.16	5.11E-12	0.637992548	183.653
ENSMUSG00000031561	*Tenm3*	ENSMUST00000190840.7	4.05E-07	-3.726879621	0
ENSMUSG00000033430	*Terf2ip*	ENSMUST00000052138.10	0.000471052	0.447428098	11.8067
ENSMUSG00000021611	*Tert*	ENSMUST00000022104.8	0.000531655	-1.25230276	0.331765
ENSMUSG00000009628	*Tex15*	ENSMUST00000009772.7	0.00806966	0.426227506	2.64851
ENSMUSG00000040548	*Tex2*	ENSMUST00000042780.13	0.002255773	0.334622245	15.8639
ENSMUSG00000038482	*Tfdp1*	ENSMUST00000209885.1	5.36E-09	0.832091692	110.098
ENSMUSG00000021253	*Tgfb3*	ENSMUST00000003687.7	0.000732924	-0.299343093	45.564
ENSMUSG00000089736	*Tgfbr3l*	ENSMUST00000124750.1	0.00710829	0.84648749	5.98338
ENSMUSG00000078921	*Tgtp2*	ENSMUST00000128411.1	0.003998218	-0.874822261	14.44
ENSMUSG00000036442	*Thap11*	ENSMUST00000040445.8	0.008020904	0.467562101	34.8454
ENSMUSG00000074743	*Thbd*	ENSMUST00000099270.4	1.19E-22	-0.982577406	15.4627
ENSMUSG00000040152	*Thbs1*	ENSMUST00000039559.8	1.52E-07	-0.969665864	86.6602
ENSMUSG00000028047	*Thbs3*	ENSMUST00000029682.10	6.76E-05	-0.492448712	35.5619
ENSMUSG00000056665	*Them6*	ENSMUST00000070923.2	0.005612429	0.423478995	15.3593
ENSMUSG00000048550	*Thnsl1*	ENSMUST00000054591.9	0.000280605	0.570543506	5.2275
ENSMUSG00000041319	*Thoc6*	ENSMUST00000047436.10	0.000300344	-0.761374193	7.82659
ENSMUSG00000044390	*Tigd3*	ENSMUST00000055911.4	0.003186864	-0.714598056	2.16714
ENSMUSG00000048429	*Timm29*	ENSMUST00000062125.10	0.003052211	-0.298086832	23.2752
ENSMUSG00000002949	*Timm44*	ENSMUST00000003029.13	0.000669754	0.377350028	43.1085
ENSMUSG00000039016	*Timm8b*	ENSMUST00000044051.5	0.004682993	-0.300684387	200.026
ENSMUSG00000030516	*Tjp1*	ENSMUST00000032729.7	0.000954541	0.339444453	19.9962
ENSMUSG00000034105	*Tldc1*	ENSMUST00000049156.6	0.002515563	0.497100923	10.1026
ENSMUSG00000024642	*Tle4*	ENSMUST00000052011.13	0.002278388	-0.517029996	3.69235
ENSMUSG00000028465	*Tln1*	ENSMUST00000030187.13	0.008252646	-0.321282415	104.823
ENSMUSG00000079164	*Tlr5*	ENSMUST00000110997.6	0.000118074	0.669415	6.8379
ENSMUSG00000031556	*Tm2d2*	ENSMUST00000033961.6	0.006569668	0.426968015	74.2197
ENSMUSG00000025544	*Tm9sf2*	ENSMUST00000026624.10	0.002476497	-0.276905066	38.537
ENSMUSG00000068040	*Tm9sf4*	ENSMUST00000089027.2	0.005361871	-0.251467383	41.8975
ENSMUSG00000025591	*Tma16*	ENSMUST00000026681.6	0.007299688	0.661747026	2.1
ENSMUSG00000052428	*Tmco1*	ENSMUST00000195015.5	0.002715591	0.276537399	133.724
ENSMUSG00000038497	*Tmco3*	ENSMUST00000045229.6	3.03E-09	0.597023456	26.408
ENSMUSG00000006850	*Tmco6*	ENSMUST00000007046.7	0.000902108	0.578464701	9.44733
ENSMUSG00000032180	*Tmed1*	ENSMUST00000034698.8	0.000155302	-0.373768154	65.0062
ENSMUSG00000026109	*Tmeff2*	ENSMUST00000081851.3	0.002544476	-1.043376043	2.506
ENSMUSG00000034947	*Tmem106a*	ENSMUST00000039581.13	0.004174515	0.542831424	8.62534
ENSMUSG00000029571	*Tmem106b*	ENSMUST00000031556.13	2.88E-05	0.426243754	64.6459
ENSMUSG00000052369	*Tmem106c*	ENSMUST00000064200.7	3.30E-08	0.535779447	85.2947
ENSMUSG00000020895	*Tmem107*	ENSMUST00000075980.11	0.000343874	0.541315181	47.7877
ENSMUSG00000034659	*Tmem109*	ENSMUST00000038128.14	0.00524799	-0.291187479	60.2441
ENSMUSG00000054675	*Tmem119*	ENSMUST00000067853.5	0.006771362	0.502826553	182.994
ENSMUSG00000034850	*Tmem127*	ENSMUST00000035871.14	0.00614677	-0.241793492	30.9162
ENSMUSG00000039428	*Tmem135*	ENSMUST00000041968.10	0.001843979	0.390632799	10.7056
ENSMUSG00000025933	*Tmem14a*	ENSMUST00000027065.11	0.001579128	0.709119885	8.56398
ENSMUSG00000056498	*Tmem154*	ENSMUST00000107682.1	0.0024486	2.286501237	1.32079
ENSMUSG00000054871	*Tmem158*	ENSMUST00000068140.5	0.00747368	-0.496943959	8.75679
ENSMUSG00000026188	*Tmem169*	ENSMUST00000027380.11	0.000178483	0.601995786	8.64266
ENSMUSG00000031953	*Tmem170*	ENSMUST00000034431.2	3.34E-05	0.691954414	4.79464
ENSMUSG00000024245	*Tmem178*	ENSMUST00000025092.4	0.001638792	-1.758081028	2.44696
ENSMUSG00000031617	*Tmem184c*	ENSMUST00000034030.14	0.000664877	0.367047153	27.0323

Table S3. RNAseq in D5 in mDPCs-CAS9 cells and sgCreb3l1 _A8_4: 1007 downregulated genes and 1027 upregulated genes

ENSMUSG00000090213	*Tmem189*	ENSMUST00000006587.6	0.000145065	-0.432091035	47.8094
ENSMUSG00000025521	*Tmem192*	ENSMUST00000026595.12	3.15E-06	0.619901235	42.9778
ENSMUSG00000014856	*Tmem208*	ENSMUST00000015000.11	4.22E-05	0.632607921	49.5519
ENSMUSG00000029782	*Tmem209*	ENSMUST00000115160.9	2.88E-12	0.656319478	61.201
ENSMUSG00000032121	*Tmem218*	ENSMUST00000034632.9	0.005936274	-0.419699628	6.18104
ENSMUSG00000031951	*Tmem231*	ENSMUST00000034429.8	0.001720387	0.621723631	14.2042
ENSMUSG00000004945	*Tmem242*	ENSMUST00000005053.13	0.001904389	-0.308128572	105.213
ENSMUSG00000079659	*Tmem243*	ENSMUST00000115365.2	0.000259595	0.675630849	16.7645
ENSMUSG00000055296	*Tmem245*	ENSMUST00000068792.12	0.007320581	-0.262306573	4.25242
ENSMUSG00000048572	*Tmem252*	ENSMUST00000057243.4	0.000249756	1.842691033	2.08009
ENSMUSG00000060044	*Tmem26*	ENSMUST00000080995.7	0.000729582	-0.62417785	9.89332
ENSMUSG00000041353	*Tmem29*	ENSMUST00000151067.7	0.002587706	-0.639696229	10.1699
ENSMUSG00000032328	*Tmem30a*	ENSMUST00000034878.11	0.002671749	0.251605587	103.629
ENSMUSG00000034435	*Tmem30b*	ENSMUST00000042975.6	2.54E-08	-3.68504892	0.0608866
ENSMUSG00000030095	*Tmem43*	ENSMUST00000032183.5	0.000367333	-0.329211791	112.319
ENSMUSG00000025666	*Tmem47*	ENSMUST00000026760.2	3.58E-06	-0.98146979	5.76992
ENSMUSG00000028221	*Tmem55a*	ENSMUST00000029875.3	0.003878915	-0.257810315	59.1947
ENSMUSG00000028826	*Tmem57*	ENSMUST00000030628.14	0.000887632	-0.306887047	30.6055
ENSMUSG00000036026	*Tmem63b*	ENSMUST00000113523.8	0.000245126	-0.348970142	28.5005
ENSMUSG00000062373	*Tmem65*	ENSMUST00000072113.5	0.007934491	0.243403781	40.8296
ENSMUSG00000054409	*Tmem74*	ENSMUST00000067469.4	1.02E-06	1.135966235	7.05818
ENSMUSG00000045377	*Tmem88*	ENSMUST00000050140.5	7.03E-05	-1.077756773	4.05739
ENSMUSG00000030059	*Tmf1*	ENSMUST00000124173.7	0.009271306	0.395112253	36.2081
ENSMUSG00000058587	*Tmod3*	ENSMUST00000072232.8	0.000917923	-0.277141435	83.0847
ENSMUSG00000030306	*Tmtc1*	ENSMUST00000060095.14	8.84E-07	-0.519062806	8.05693
ENSMUSG00000036019	*Tmtc2*	ENSMUST00000061506.8	1.34E-20	1.863254569	7.82674
ENSMUSG00000041594	*Tmtc4*	ENSMUST00000037726.13	0.006890812	-0.418257194	4.19786
ENSMUSG00000022074	*Tnfrsf10b*	ENSMUST00000022663.6	0.00078071	-0.333463618	22.4222
ENSMUSG00000037613	*Tnfrsf23*	ENSMUST00000152703.1	0.000686394	1.023385233	13.1258
ENSMUSG00000031529	*Tnks*	ENSMUST00000033929.5	1.68E-08	0.481858672	32.0512
ENSMUSG00000026725	*Tnn*	ENSMUST00000039178.11	8.94E-06	0.609268831	23.4668
ENSMUSG00000091898	*Tnnc1*	ENSMUST00000169169.7	5.77E-06	-1.669170369	2.13945
ENSMUSG00000031691	*Tnpo2*	ENSMUST00000093360.11	1.64E-06	0.457102872	34.8149
ENSMUSG00000042870	*Tom1*	ENSMUST00000165630.2	9.39E-07	0.528630871	39.3353
ENSMUSG00000033475	*Tomm6*	ENSMUST00000113302.9	0.000515882	-0.322854172	209.766
ENSMUSG00000036822	*Topors*	ENSMUST00000042575.6	7.67E-06	-0.385188197	47.9412
ENSMUSG00000009563	*Tor2a*	ENSMUST00000009707.13	0.003477006	-0.344680496	28.8946
ENSMUSG00000041272	*Tox*	ENSMUST00000039987.3	0.008422312	-0.255270505	21.4145
ENSMUSG00000027506	*Tpd52*	ENSMUST00000091354.11	1.08E-10	1.453566619	8.78205
ENSMUSG00000000296	*Tpd52l1*	ENSMUST00000214644.1	0.000622197	0.529288921	27.6815
ENSMUSG00000031799	*Tpm4*	ENSMUST00000003575.9	1.64E-08	0.516477683	898.689
ENSMUSG00000014846	*Tppp3*	ENSMUST00000014990.12	9.97E-07	0.976511472	13.402
ENSMUSG00000006005	*Tpr*	ENSMUST00000124973.8	0.007547693	0.373509136	55.1223
ENSMUSG00000027469	*Tpx2*	ENSMUST00000109816.7	2.46E-08	-0.513126932	42.6215
ENSMUSG00000031887	*Tradd*	ENSMUST00000034359.9	0.000267079	0.577156158	15.0269
ENSMUSG00000038102	*Trappc11*	ENSMUST00000039061.14	2.44E-08	0.546991944	30.2729
ENSMUSG00000028847	*Trappc3*	ENSMUST00000030660.8	0.005184866	-0.269913666	147.931
ENSMUSG00000040236	*Trappc5*	ENSMUST00000044857.3	0.000998996	0.387523376	22.9079
ENSMUSG00000020993	*Trappc6b*	ENSMUST00000021380.9	0.003246617	0.299805587	97.8631
ENSMUSG00000030966	*Trim21*	ENSMUST00000033264.11	0.005549748	-0.405480179	11.4654
ENSMUSG00000029833	*Trim24*	ENSMUST00000120428.7	0.002335076	0.334222334	28.2961
ENSMUSG00000030921	*Trim30a*	ENSMUST00000076922.5	0.000558828	0.877002856	42.9951
ENSMUSG00000057596	*Trim30d*	ENSMUST00000071069.12	9.50E-31	2.33067175	11.4994
ENSMUSG00000051675	*Trim32*	ENSMUST00000050850.13	0.006796868	-0.29165883	27.3394
ENSMUSG00000027189	*Trim44*	ENSMUST00000102573.7	8.65E-05	-0.335070878	33.2555
ENSMUSG00000037376	*Trmt6*	ENSMUST00000039554.6	0.009595947	-0.2837497	18.571
ENSMUSG00000018199	*Trove2*	ENSMUST00000159879.1	0.001978626	0.329304547	9.65654
ENSMUSG00000028211	*Trp53inp1*	ENSMUST00000029865.3	1.81E-20	-1.147835941	10.3513
ENSMUSG00000038375	*Trp53inp2*	ENSMUST00000043237.13	0.000229898	-0.442334774	35.6431
ENSMUSG00000002496	*Tsc2*	ENSMUST00000097373.1	3.75E-06	-0.408796124	22.5303
ENSMUSG00000056820	*Tsnax*	ENSMUST00000075896.6	2.92E-08	0.501923959	85.1842
ENSMUSG00000020577	*Tspan13*	ENSMUST00000020896.16	6.84E-07	1.549214714	4.75551
ENSMUSG00000037300	*Ttc13*	ENSMUST00000214828.1	2.93E-05	0.487090102	21.2251
ENSMUSG00000024078	*Ttc27*	ENSMUST00000024882.6	0.001468324	-0.373148737	14.7838
ENSMUSG00000038172	*Ttc39b*	ENSMUST00000048274.10	0.004177831	-0.880183744	3.90619
ENSMUSG00000062380	*Tubb3*	ENSMUST00000071134.3	0.000233153	1.108611608	3.63688
ENSMUSG00000036752	*Tubb4b*	ENSMUST00000043584.4	0.002578294	-0.326224352	336.983
ENSMUSG00000000759	*Tubgcp3*	ENSMUST00000000776.14	1.05E-05	0.414314132	43.002
ENSMUSG00000039530	*Tusc3*	ENSMUST00000209440.1	3.74E-06	0.566589071	211.338
ENSMUSG00000046275	*Tusc5*	ENSMUST00000062024.2	0.002021127	-1.916832712	0.136558
ENSMUSG00000014177	*Tvp23b*	ENSMUST00000014321.4	0.001744825	0.324867779	56.4871
ENSMUSG00000020561	*Twistnb*	ENSMUST00000020877.8	0.004335478	0.293549419	17.791

ENSMUSG00000031723	*Txnl4b*	ENSMUST00000034159.7	1.06E-05	0.717689191	12.1453
ENSMUSG00000090137	*Uba52*	ENSMUST00000081940.10	3.39E-05	0.477106221	254.91
ENSMUSG00000041765	*Ubac2*	ENSMUST00000039803.5	0.002242047	-0.396621377	13.2165
ENSMUSG00000021774	*Ube2e1*	ENSMUST00000022296.6	0.000508944	0.425659225	38.0737
ENSMUSG00000058317	*Ube2e2*	ENSMUST00000150727.7	0.000967878	0.464771933	30.0978
ENSMUSG00000078923	*Ube2v1*	ENSMUST00000109207.9	3.05E-05	-0.360484546	111.642
ENSMUSG00000025939	*Ube2w*	ENSMUST00000117146.7	0.008841623	0.251469852	54.4137
ENSMUSG00000025171	*Ubtd1*	ENSMUST00000026170.1	0.007551077	-0.435982597	26.0627
ENSMUSG00000020634	*Ubxn2a*	ENSMUST00000020962.11	0.002467229	0.37390763	19.2629
ENSMUSG00000052906	*Ubxn8*	ENSMUST00000095349.5	1.11E-06	0.643584738	14.9169
ENSMUSG00000033685	*Ucp2*	ENSMUST00000207748.1	0.006367202	0.683457527	75.322
ENSMUSG00000062963	*Ufc1*	ENSMUST00000080001.8	0.0031611	0.307273821	111.846
ENSMUSG00000031634	*Ufsp2*	ENSMUST00000034051.6	2.40E-07	0.64757389	31.4155
ENSMUSG00000042104	*Uggt2*	ENSMUST00000156203.7	0.000623557	-0.460439132	2.84638
ENSMUSG00000001228	*Uhrf1*	ENSMUST00000001258.14	0.006427441	-0.259897175	91.2518
ENSMUSG00000004798	*Ulk2*	ENSMUST00000004920.3	0.001452347	0.361758492	12.3599
ENSMUSG00000034799	*Unc13a*	ENSMUST00000030170.14	0.000232335	0.873395389	0.799735
ENSMUSG00000059921	*Unc5c*	ENSMUST00000106236.8	2.40E-33	-1.613735907	1.3204
ENSMUSG00000058301	*Upf1*	ENSMUST00000215817.1	0.003819637	0.351916053	19.8157
ENSMUSG00000043241	*Upf2*	ENSMUST00000060092.12	0.005147077	-0.255352686	22.2111
ENSMUSG00000038398	*Upf3a*	ENSMUST00000043767.8	0.000146716	0.57371594	48.1607
ENSMUSG00000031976	*Urb2*	ENSMUST00000034457.8	0.000402941	0.612775094	5.21227
ENSMUSG00000069020	*Urm1*	ENSMUST00000091142.3	1.25E-05	-0.439878405	44.4735
ENSMUSG00000028684	*Urod*	ENSMUST00000030446.14	0.006976968	-0.287586091	44.5569
ENSMUSG00000002395	*Use1*	ENSMUST00000019169.7	0.003123273	0.449459951	48.585
ENSMUSG00000029407	*Uso1*	ENSMUST00000031355.9	0.004850191	0.23772096	75.8964
ENSMUSG00000031826	*Usp10*	ENSMUST00000108988.8	9.74E-06	0.666236124	16.3181
ENSMUSG00000054568	*Usp17la*	ENSMUST00000067695.7	0.00017983	1.749671203	1.63988
ENSMUSG00000030107	*Usp18*	ENSMUST00000032198.10	0.007053371	-0.385324076	84.8909
ENSMUSG00000028514	*Usp24*	ENSMUST00000094933.4	3.73E-07	-0.439767168	16.082
ENSMUSG00000022867	*Usp25*	ENSMUST00000023580.6	0.000716728	0.307618876	35.0995
ENSMUSG00000038250	*Usp38*	ENSMUST00000042724.7	7.02E-07	0.549063305	14.8782
ENSMUSG00000056305	*Usp39*	ENSMUST00000070345.4	0.001967304	0.295805979	55.9914
ENSMUSG00000020020	*Usp44*	ENSMUST00000216224.1	0.00528799	1.207673382	0.969084
ENSMUSG00000041264	*Uspl1*	ENSMUST00000050472.15	0.006890685	-0.602558707	3.736
ENSMUSG00000047712	*Ust*	ENSMUST00000061601.8	0.000713696	0.413639569	10.872
ENSMUSG00000028907	*Utp11*	ENSMUST00000030738.7	0.000722083	-0.325517305	62.1233
ENSMUSG00000041438	*Utp4*	ENSMUST00000047629.6	0.000485197	0.352298261	47.1066
ENSMUSG00000068457	*Uty*	ENSMUST00000154004.7	2.35E-06	0.599903909	17.8057
ENSMUSG00000010936	*Vac14*	ENSMUST00000034190.10	0.001114376	0.568592595	46.3314
ENSMUSG00000028955	*Vamp3*	ENSMUST00000030797.3	0.000106847	-0.409577102	49.5183
ENSMUSG00000026696	*Vamp4*	ENSMUST00000135241.7	0.003444548	0.347508275	40.0842
ENSMUSG00000039646	*Vasn*	ENSMUST00000038770.3	0.00396462	-0.252908696	83.2063
ENSMUSG00000046844	*Vat1l*	ENSMUST00000049509.6	2.22E-19	1.554640706	7.12942
ENSMUSG00000009621	*Vav2*	ENSMUST00000056176.7	0.000377354	-0.413536138	7.03071
ENSMUSG00000033721	*Vav3*	ENSMUST00000046864.13	0.000187248	-0.782183094	1.21938
ENSMUSG00000021614	*Vcan*	ENSMUST00000109546.8	1.28E-08	-0.981874951	33.4889
ENSMUSG00000028452	*Vcp*	ENSMUST00000030164.7	0.009712117	-0.230469282	309.614
ENSMUSG00000021771	*Vdac2*	ENSMUST00000022293.13	0.006990087	-0.245400092	75.8186
ENSMUSG00000008892	*Vdac3*	ENSMUST00000009036.10	3.56E-12	0.600185502	237.419
ENSMUSG00000023951	*Vegfa*	ENSMUST00000071648.11	0.001175251	0.339256746	33.9706
ENSMUSG00000031520	*Vegfc*	ENSMUST00000033919.5	1.59E-13	0.955887432	22.7897
ENSMUSG00000021038	*Vipas39*	ENSMUST00000072744.14	0.001058218	-0.355179108	19.9959
ENSMUSG00000024076	*Vit*	ENSMUST00000024880.9	1.00E-05	-2.125767646	0.405874
ENSMUSG00000073131	*Vma21*	ENSMUST00000114576.8	0.008054953	0.226883944	61.1285
ENSMUSG00000046230	*Vps13a*	ENSMUST00000068156.7	0.000411207	-0.432792247	4.12976
ENSMUSG00000037646	*Vps13b*	ENSMUST00000048646.7	0.003521417	0.28259215	7.10154
ENSMUSG00000027411	*Vps16*	ENSMUST00000028900.10	0.007139164	-0.256440345	29.7814
ENSMUSG00000034216	*Vps18*	ENSMUST00000037280.4	0.001995667	-0.292033115	24.377
ENSMUSG00000031696	*Vps35*	ENSMUST00000034131.9	1.92E-09	0.500855803	157.932
ENSMUSG00000031600	*Vps37a*	ENSMUST00000098817.2	1.66E-14	0.81515377	14.3875
ENSMUSG00000031913	*Vps4a*	ENSMUST00000034388.9	8.07E-08	0.544794131	49.5011
ENSMUSG00000020128	*Vps54*	ENSMUST00000109578.7	0.004163147	0.557068009	8.81757
ENSMUSG00000008958	*Vps72*	ENSMUST00000009102.8	0.004916128	-0.26599341	83.2561
ENSMUSG00000021115	*Vrk1*	ENSMUST00000072040.6	0.001589475	-0.478493014	11.2963
ENSMUSG00000054459	*Vsnl1*	ENSMUST00000072299.6	0.001960537	1.301972819	2.64346
ENSMUSG00000017344	*Vtn*	ENSMUST00000017488.4	1.22E-06	-4.155834012	0
ENSMUSG00000021266	*Wars*	ENSMUST00000109848.9	0.000541572	-0.33654557	37.3837
ENSMUSG00000028868	*Wasf2*	ENSMUST00000084241.11	0.000748956	-0.303358496	68.96
ENSMUSG00000029684	*Wasl*	ENSMUST00000031695.14	2.99E-08	0.473292875	69.4115
ENSMUSG00000047731	*Wbp1l*	ENSMUST00000099376.10	0.007527522	-0.34402381	16.6242
ENSMUSG00000014547	*Wdfy2*	ENSMUST00000014691.9	7.90E-05	-0.439125736	5.8953

Table S3. RNAseq in D5 in mDPCs-CAS9 cells and sgCreb3l1 _A8_4: 1007 downregulated genes and 1027 upregulated genes

ENSMUSG00000025737	*Wdr24*	ENSMUST00000026833.5	0.005652037	-0.409992903	8.1253
ENSMUSG00000039382	*Wdr45*	ENSMUST00000043045.9	0.005044377	0.367340982	34.5175
ENSMUSG00000031959	*Wdr59*	ENSMUST00000038193.14	0.000981198	0.417657181	10.1409
ENSMUSG00000042050	*Wdr60*	ENSMUST00000039349.7	0.006211994	-0.333577767	8.09267
ENSMUSG00000061559	*Wdr61*	ENSMUST00000051822.12	0.00022167	-0.453302751	38.1409
ENSMUSG00000042729	*Wdr74*	ENSMUST00000049424.10	0.005250135	-0.36317647	31.5873
ENSMUSG00000059355	*Wdr83os*	ENSMUST00000079764.13	1.19E-05	0.606004085	53.3141
ENSMUSG00000055235	*Wdr86*	ENSMUST00000068693.11	2.69E-05	-3.562514791	0.27718
ENSMUSG00000041245	*Wnk3*	ENSMUST00000184730.7	0.000598167	-0.653228742	1.19205
ENSMUSG00000026167	*Wnt10a*	ENSMUST00000006718.14	0.00060087	1.100631716	53.67
ENSMUSG00000029671	*Wnt16*	ENSMUST00000031681.9	0.003602931	0.453842531	14.9814
ENSMUSG00000033227	*Wnt6*	ENSMUST00000006716.7	0.004190338	0.876056919	18.4589
ENSMUSG00000031583	*Wrn*	ENSMUST00000033991.12	1.51E-06	0.693778793	5.32431
ENSMUSG00000031563	*Wwc2*	ENSMUST00000057561.8	3.51E-15	0.706326002	34.2692
ENSMUSG00000031930	*Wwp2*	ENSMUST00000166615.2	4.38E-10	0.673945993	25.1014
ENSMUSG00000019470	*Xab2*	ENSMUST00000019614.12	0.000875327	0.352101633	32.9359
ENSMUSG00000040483	*Xaf1*	ENSMUST00000094041.3	0.003326204	-0.505949592	58.4156
ENSMUSG00000025860	*Xiap*	ENSMUST00000115094.7	0.000127952	0.440056935	33.8538
ENSMUSG00000015342	*Xk*	ENSMUST00000015486.6	0.000573763	0.634506094	2.61408
ENSMUSG00000025027	*Xpnpep1*	ENSMUST00000182877.7	0.000740151	-0.335479512	36.0454
ENSMUSG00000037005	*Xpnpep2*	ENSMUST00000077775.10	7.17E-09	0.974671673	25.8261
ENSMUSG00000026469	*Xpr1*	ENSMUST00000027741.11	0.008115143	0.269469494	12.3578
ENSMUSG00000026187	*Xrcc5*	ENSMUST00000027379.8	0.000186501	0.355996119	50.5667
ENSMUSG00000028639	*Ybx1*	ENSMUST00000079644.12	0.002453396	-0.295486692	891.007
ENSMUSG00000014932	*Yes1*	ENSMUST00000072311.12	0.004313174	0.287837889	18.8031
ENSMUSG00000024875	*Yif1a*	ENSMUST00000025811.4	0.004643309	-0.313988112	35.5646
ENSMUSG00000048967	*Yjefn3*	ENSMUST00000152938.8	0.0011967	1.87668038	7.93477
ENSMUSG00000042675	*Ypel3*	ENSMUST00000038614.11	0.000227535	0.581799283	81.9859
ENSMUSG00000047213	*Ythdf3*	ENSMUST00000108345.8	0.0030295	-0.296729423	22.0197
ENSMUSG00000018326	*Ywhab*	ENSMUST00000018470.9	0.002753927	-0.250047634	144.885
ENSMUSG00000069114	*Zbtb10*	ENSMUST00000155203.1	0.008172324	0.382700352	6.49194
ENSMUSG00000049657	*Zbtb5*	ENSMUST00000055028.8	0.003012916	-0.416090808	11.3072
ENSMUSG00000028807	*Zbtb8a*	ENSMUST00000030610.2	0.001550468	-0.495289585	6.60582
ENSMUSG00000043542	*Zc2hc1a*	ENSMUST00000051064.8	0.002187341	0.480151022	46.8648
ENSMUSG00000017478	*Zc3h18*	ENSMUST00000093073.11	1.62E-06	0.579372751	59.0993
ENSMUSG00000047749	*Zc3hav1l*	ENSMUST00000058524.2	0.004683244	0.28031667	15.0428
ENSMUSG00000039199	*Zdhhc1*	ENSMUST00000212303.1	1.84E-05	0.827693039	11.3837
ENSMUSG00000030471	*Zdhhc13*	ENSMUST00000118927.7	0.008482362	0.341182955	15.0011
ENSMUSG00000033906	*Zdhhc15*	ENSMUST00000042070.5	4.75E-06	0.452991376	18.2339
ENSMUSG00000025157	*Zdhhc16*	ENSMUST00000026154.8	0.002477719	-0.335357466	30.2109
ENSMUSG00000028403	*Zdhhc21*	ENSMUST00000030110.14	0.004741599	0.298279382	9.55194
ENSMUSG00000034075	*Zdhhc5*	ENSMUST00000035840.5	0.005409795	-0.234258806	48.3539
ENSMUSG00000024238	*Zeb1*	ENSMUST00000025081.11	1.02E-07	0.545214342	17.8136
ENSMUSG00000052763	*Zfp212*	ENSMUST00000009411.8	0.004780143	0.280801866	37.719
ENSMUSG00000063108	*Zfp26*	ENSMUST00000098970.9	0.000562244	-0.551969989	4.22753
ENSMUSG00000001065	*Zfp276*	ENSMUST00000001092.14	0.000614419	0.514886057	8.94562
ENSMUSG00000046351	*Zfp322a*	ENSMUST00000050101.8	0.006310267	-0.345380722	5.32426
ENSMUSG00000031711	*Zfp330*	ENSMUST00000034147.3	2.98E-06	0.541818246	41.4101
ENSMUSG00000017667	*Zfp334*	ENSMUST00000103084.3	0.000869827	-0.350542643	6.42183
ENSMUSG00000039834	*Zfp335*	ENSMUST00000041361.13	0.000327998	-0.386430065	12.1592
ENSMUSG00000074731	*Zfp345*	ENSMUST00000109914.1	0.000124903	1.638645562	1.27969
ENSMUSG00000021127	*Zfp36l1*	ENSMUST00000021552.2	9.57E-06	-0.411775656	92.5514
ENSMUSG00000045817	*Zfp36l2*	ENSMUST00000060366.6	3.43E-06	-0.549435618	76.9001
ENSMUSG00000028389	*Zfp37*	ENSMUST00000068822.3	0.003385251	-0.664945814	1.45292
ENSMUSG00000042063	*Zfp386*	ENSMUST00000073551.5	0.000135552	-0.515638883	11.5232
ENSMUSG00000075040	*Zfp408*	ENSMUST00000111333.1	0.000188109	-0.940677127	3.59355
ENSMUSG00000042472	*Zfp410*	ENSMUST00000045931.11	0.000874317	-0.380638221	20.1725
ENSMUSG00000051351	*Zfp46*	ENSMUST00000069195.4	0.002351906	-0.436558408	13.1585
ENSMUSG00000024420	*Zfp521*	ENSMUST00000025288.7	0.003381029	0.601407936	43.2302
ENSMUSG00000062794	*Zfp599*	ENSMUST00000086281.4	0.00185436	-0.543010341	2.33032
ENSMUSG00000052713	*Zfp608*	ENSMUST00000064763.5	0.001321418	-0.302582958	14.792
ENSMUSG00000066880	*Zfp617*	ENSMUST00000119003.1	0.001478352	0.400207271	13.8993
ENSMUSG00000028358	*Zfp618*	ENSMUST00000107415.7	7.28E-07	-0.545916406	3.98904
ENSMUSG00000040209	*Zfp704*	ENSMUST00000041124.12	9.31E-08	0.604255605	4.82433
ENSMUSG00000062397	*Zfp706*	ENSMUST00000078976.7	0.00013259	0.321253468	86.5651
ENSMUSG00000056019	*Zfp709*	ENSMUST00000188685.6	3.41E-06	0.830621062	2.82996
ENSMUSG00000074194	*Zfp791*	ENSMUST00000211109.1	0.004181376	0.66943477	3.25064
ENSMUSG00000069743	*Zfp820*	ENSMUST00000084141.4	0.00120909	-0.441739524	5.37416
ENSMUSG00000071064	*Zfp827*	ENSMUST00000098614.8	3.40E-05	0.506532589	7.89176
ENSMUSG00000043090	*Zfp866*	ENSMUST00000137573.1	0.001923818	0.404161467	10.7163
ENSMUSG00000060427	*Zfp868*	ENSMUST00000121886.7	0.002381197	0.400968104	16.4445
ENSMUSG00000054648	*Zfp869*	ENSMUST00000080987.6	0.001026227	0.4393726	22.895

Table S3. RNAseq in D5 in mDPCs-CAS9 cells and sgCreb3l1 _A8_4: 1007 downregulated genes and 1027 upregulated genes

ENSMUSG00000089857	*Zfp882*	ENSMUST00000110002.7	0.006188095	0.806544311	1.27436
ENSMUSG00000059897	*Zfp930*	ENSMUST00000212681.1	0.003380359	0.445645775	8.68815
ENSMUSG00000078861	*Zfp931*	ENSMUST00000108923.1	0.008848713	-0.498719341	5.25626
ENSMUSG00000060314	*Zfp941*	ENSMUST00000106052.1	0.000366346	0.822889777	1.77158
ENSMUSG00000067931	*Zfp948*	ENSMUST00000088787.5	0.009746282	-0.278194794	19.2159
ENSMUSG00000092260	*Zfp963*	ENSMUST00000130458.7	1.95E-07	1.029415222	6.26908
ENSMUSG00000091764	*Zfp964*	ENSMUST00000204285.2	0.00917904	0.500281644	2.55838
ENSMUSG00000078896	*Zfp965*	ENSMUST00000109042.9	0.005784152	-0.688317322	2.98699
ENSMUSG00000078898	*Zfp968*	ENSMUST00000131676.7	0.002744673	-1.018360507	1.64527
ENSMUSG00000095990	*Zfp97*	ENSMUST00000147630.1	0.000826276	-0.440361486	9.08853
ENSMUSG00000074519	*Zfp971*	ENSMUST00000108925.9	2.42E-05	-1.063180155	4.55565
ENSMUSG00000078879	*Zfp973*	ENSMUST00000121956.7	0.005784152	-0.688317322	2.98699
ENSMUSG00000022306	*Zfpm2*	ENSMUST00000053467.4	1.97E-06	1.79245658	1.56514
ENSMUSG00000022201	*Zfr*	ENSMUST00000122941.7	0.009975834	-0.215115663	80.3317
ENSMUSG00000027582	*Zgpat*	ENSMUST00000108807.8	0.000275127	-0.721043707	7.22001
ENSMUSG00000035877	*Zhx3*	ENSMUST00000109460.7	0.003479682	-0.533537262	3.74802
ENSMUSG00000007817	*Zmiz1*	ENSMUST00000162645.7	0.003637425	-0.315794304	7.72692
ENSMUSG00000042408	*Zmym6*	ENSMUST00000046751.12	0.000415188	-0.512375524	3.98745
ENSMUSG00000044068	*Zrsr1*	ENSMUST00000049506.6	0.001453477	-0.37570953	9.87017
ENSMUSG00000035671	*Zswim4*	ENSMUST00000039480.6	8.93E-06	0.507461289	14.4954
ENSMUSG00000032264	*Zw10*	ENSMUST00000034803.9	0.005006893	-0.28427182	24.3854
ENSMUSG00000029860	*Zyx*	ENSMUST00000203652.2	0.00020284	0.318600215	262.686

Table S3. RNAseq in D5 in mDPCs-CAS9 cells and sgCreb3l1 _A8_4: 1007 downregulated genes and 1027 upregulated genes

D5_A8_4_2_tpm	D5_A8_4_3_tpm	D5_Ctrl_1_tpm	D5_Ctrl_2_tpm	D5_Ctrl_3_tpm
0.877447	0.489675	2.66576	3.03801	4.11376
0.143093	0	7.68964	5.87125	1.40896
1.48178	0.743881	7.93603	7.91227	7.91569
10.8447	11.3683	5.1612	5.37921	5.4454
58.3279	66.6092	83.4146	97.6791	111.024
440.953	523.63	631.196	639.973	762.278
1.27453	1.99919	4.06578	4.58208	4.13357
118.875	83.559	62.0634	61.7028	45.0298
15.6767	19.4281	12.3848	11.3914	10.8555
54.7636	59.1949	38.2574	42.1737	33.6059
19.9784	19.3351	31.2613	33.1504	25.7517
5.7988	5.26341	14.3547	14.0171	14.2989
47.9167	39.3179	75.773	69.2683	71.1751
28.9124	29.5863	15.319	15.0658	17.3471
13.3385	10.6173	17.4467	16.8584	17.5693
1.54178	1.03339	5.81775	9.39248	6.95035
28.5902	28.0607	17.6089	16.071	17.2571
10.569	14.0919	16.173	15.5786	16.5292
5.24193	4.3487	2.78378	2.44434	3.40359
91.5183	113.276	56	55.5676	54.5707
11.1229	12.0432	16.783	18.6913	14.9951
60.3953	75.6048	29.3308	28.1635	37.6451
186.657	130.655	107.23	106.328	67.7365
6.16846	4.90103	10.648	11.3904	7.55603
0.24123	0.17377	0.505271	0.99475	0.699949
14.402	14.6083	8.71393	8.88694	7.36739
12.1267	10.6454	14.1279	14.6075	13.147
2.35841	1.71452	0.809928	0.768306	0.846564
6.56275	3.88176	13.0723	12.8418	19.2395
13.2618	14.4945	5.48638	7.14391	5.90759
16.6796	18.1472	11.607	11.9707	10.8607
0.798943	0.400407	0.239256	0.236301	0.204129
6.66048	6.38966	10.7297	11.5622	9.80378
73.0075	79.9146	44.0619	43.6174	46.0052
14.4763	13.8266	7.4574	6.05094	5.6332
20.8342	25.726	11.3284	11.1386	12.4382
24.4796	32.0812	16.5217	15.5085	15.0077
9.10423	10.0962	32.8555	37.3	25.9937
47.5356	32.9661	30.4881	29.3806	27.418
0	0	2.11259	1.94693	1.28706
55.9256	69.5228	45.8441	44.8557	49.3893
67.6883	67.0605	98.524	95.5774	90.7905
57.8519	66.8945	43.5294	45.6164	37.5495
20.7414	17.6677	13.289	7.41463	6.86374
41.0695	41.3966	58.4201	56.314	47.5261
0.699194	1.47448	0.24727	0.242759	0.322705
18.7282	32.5332	11.0203	13.1467	14.4592
5.45834	8.50278	15.0501	14.4782	12.0365
84.0313	74.9253	117.419	114.467	111.472
76.3756	145.093	41.6312	42.802	51.586
199.893	162.513	236.785	230.795	226.811
22.1928	15.1114	39.743	39.7833	42.9554
1.28255	1.56717	0.0512302	0.0306187	0.153643
3.34853	5.61054	2.3303	2.13911	2.29795
4.66298	5.68381	10.0297	8.18225	9.59722
9.79799	7.2705	0.972541	1.42825	1.82316
23.6758	20.0386	33.937	34.7921	31.056
98.1062	72.853	67.563	67.6038	51.6701
3.05411	2.22769	1.01577	1.24466	0.895352
1.5305	2.8257	4.73011	5.1424	3.77978
83.1774	75.0407	102.604	101.048	103.036
81.758	73.0965	59.2616	62.6916	58.4618
56.0305	38.4812	24.8177	25.2751	13.6505
96.7825	73.2475	123.919	118.952	109.035
63.7377	70.0412	54.2416	50.4445	47.659
19.9533	15.9735	37.2497	37.9925	25.9751
20.4228	19.78	10.4715	8.36341	9.01482
18.869	22.8469	13.1827	13.8675	13.8061

Table S3. RNAseq in D5 in mDPCs-CAS9 cells and sgCreb3l1 _A8_4: 1007 downregulated genes and 1027 upregulated genes

175.526	130.27	101.452	99.7457	96.8417
23.5889	19.6394	38.3888	37.3736	33.9794
56.8659	38.6954	106.755	112.244	68.5705
0	0	1.17776	0.724564	1.30538
1.51602	1.04315	8.32259	7.65248	9.14189
84.6008	99.7658	159.643	151.039	137.701
10.3925	12.0997	8.28473	9.00614	7.36476
20.792	24.036	28.8566	30.3125	32.1014
50.1778	53.3799	22.7568	23.7608	36.7038
9.14369	8.13573	2.88492	3.59548	5.9466
4.59568	6.61802	2.61067	2.35426	2.32683
42.5256	38.275	61.2604	57.666	53.9608
36.7105	26.1568	46.9324	49.795	45.7234
30.729	22.3743	77.6984	74.2431	64.7305
104.796	94.4447	125.436	136.974	130.629
33.951	36.3109	55.4135	54.6446	48.4257
104.673	107.934	160.757	151.113	176.765
38.5592	42.7337	57.309	63.7004	48.3877
9.64882	6.67545	6.04527	4.28302	4.53938
18.9376	17.5022	28.1172	24.7305	26.876
5.3027	6.658	4.01455	3.96679	3.36036
38.5439	47.2687	56.6969	61.7706	60.1471
602.999	505.277	724.11	718.838	706.001
0	0.0920433	0.955162	0.889489	1.33659
10.4197	17.1163	1.80354	2.31591	2.54977
166.213	116.549	222.973	219.906	179.861
19.4396	17.2379	11.5389	10.842	9.42451
111.525	87.4656	64.6784	62.0778	26.0949
7.31986	6.19431	18.9335	17.9093	15.3311
11.8587	11.2194	16.2524	16.2873	15.2457
12.3191	16.9375	8.21102	7.83241	9.72888
22.4046	20.8655	31.1123	29.9197	26.1681
5.54065	4.27508	2.03533	1.49337	1.90107
71.5284	75.4219	54.1393	53.5245	49.7127
8.91694	5.78977	18.7221	19.7503	16.9386
83.9275	74.4089	38.7531	37.0377	34.2978
3.09765	3.24765	5.37126	5.47855	4.28427
1.58462	3.59255	9.14092	8.2889	6.62795
14.5936	20.2665	22.5655	23.413	23.6879
8.27164	10.7379	5.32435	4.81749	5.24092
30.1594	22.9722	40.2713	41.3431	32.9817
10.0056	6.25793	22.2569	20.653	15.7869
22.5649	27.5345	18.7928	17.7637	14.9258
1.76097	2.15171	0.283728	0.120125	0.220281
9.39609	8.62705	4.27723	5.38655	5.47912
33.1409	22.6303	14.3995	15.79	12.532
8.02377	9.77828	11.9848	12.1805	10.6791
700.483	669.029	874.677	868.686	865.822
221.364	257.517	135.701	134.14	149.27
97.8135	89.3916	150.23	142.625	133.233
39.3772	56.4939	28.6081	26.0452	32.1932
1.53977	1.31253	4.62382	4.78573	4.82464
21.5317	21.2341	11.3334	11.62	10.1322
49.4709	55.7762	30.4636	29.9256	30.2066
5.19234	2.77027	8.77453	8.00562	6.34473
4.44034	4.34148	1.36203	1.02271	1.38259
39.6146	39.5931	54.8242	53.6871	47.0204
23.2796	19.3049	16.206	13.6083	16.745
4.86929	3.2899	6.30257	6.33753	5.15877
53.7942	60.1587	85.8074	74.7096	85.85
52.908	54.0934	72.2766	75.1734	77.5251
11.9392	12.0603	7.89379	6.8399	4.86613
78.3799	114.189	170.805	160.013	176.699
143.644	119.043	85.827	77.1918	87.9046
24.4888	44.6183	12.7644	11.8864	19.1687
15.8251	28.2224	2.38042	1.36435	4.03596
29.4649	27.8074	39.5693	42.4914	34.5111
3.77629	2.12678	0.210752	0.21085	0.142614
5.89573	7.88958	3.62688	3.31427	2.37966
27.9942	23.4193	48.7282	48.3838	41.7341
116.812	106.449	97.4517	92.5428	80.7729
28.8037	42.4323	14.5392	15.9581	17.1271

42.6802	26.9778	21.38	22.1484	15.8224
22.8591	20.8687	14.5058	13.675	16.7686
9.25455	7.23967	17.7239	14.7026	11.3973
1.57825	2.22034	7.92879	6.52661	6.3856
23.5688	22.2664	45.3939	45.9005	44.3907
19.2462	25.7009	28.1547	28.9109	27.6258
6.87161	5.74971	11.4294	11.6753	9.62869
2.39694	2.33867	4.4286	5.06201	3.73011
27.2734	14.5545	11.6768	10.7302	8.8578
3.21146	4.84756	1.98743	1.89651	1.80685
19.4656	15.296	12.9042	11.9454	9.82628
26.9965	25.9094	18.2371	17.4122	15.8187
24.0937	16.188	8.73064	9.89254	6.29071
27.7996	20.4635	15.9439	15.2876	12.3982
14.0268	11.8293	20.5061	21.2363	15.1175
236.991	297.491	187.609	191.82	215.194
104.68	83.2631	55.1497	57.8075	45.7662
109.471	78.1261	140.379	141.984	130.769
28.9193	14.2142	11.5211	11.656	6.62854
270.005	344.481	153.337	154.655	200.716
103.494	98.9787	77.4882	75.4628	78.0226
12.2848	8.11925	17.2591	20.3207	19.1612
10.9959	6.87461	16.3728	15.5295	12.8082
70.5488	51.2491	89.2582	91.6199	78.6035
2.36479	4.0768	7.76736	6.03665	9.55318
161.315	177.701	113.136	113.262	109.04
9.07541	5.83062	12.7165	13.3762	11.3734
27.1245	25.992	18.0324	17.3292	13.1672
42.2564	40.6266	23.1745	22.7594	20.7981
5.67277	4.28446	8.1215	11.3628	7.79481
31.8877	42.2896	18.1738	17.3401	18.4974
7.80549	5.91024	3.76225	3.4807	4.70326
0	0	1.28219	0.874147	0.974556
172.994	139.935	104.073	106.207	113.245
31.4928	25.1952	44.4802	45.4726	33.2261
2.28386	0.471749	7.05504	6.75666	3.29097
22.6946	22.8282	28.2908	29.4331	26.5736
141.998	138.096	109.776	110.142	98.1421
71.5946	77.5087	61.4207	63.7242	52.4745
7.43628	13.8631	2.42667	2.69828	3.86079
403.466	359.02	325.72	326.261	243.49
19.3073	21.3278	13.7964	13.2374	11.7883
36.6369	31.8674	50.3201	49.5845	41.86
2.8205	3.19064	5.19339	5.07273	4.44666
16.0072	14.692	18.0031	18.9414	22.3594
12.4285	5.43896	19.4582	22.384	14.754
29.0188	23.2583	13.6577	13.7471	11.6924
36.8412	28.02	22.6076	21.2749	19.5595
38.6137	55.0142	30.5233	31.3956	29.0417
445.242	365.816	590.063	583.195	687.104
149.272	130.469	94.0435	100.261	98.8547
292.894	264.414	421.636	416.056	449.108
255.392	261.638	207.957	198.542	190.082
165.133	118.31	103.354	103.902	94.3431
20.5072	17.9893	8.87255	8.35386	7.29567
64.1762	62.1663	34.4538	37.3099	30.2787
32.8219	42.411	62.7633	63.8238	59.3955
0.144971	0.232467	1.09382	1.29893	1.7248
754.056	627.242	874.284	879.942	1002.83
4.46174	4.08813	6.17148	6.20309	5.72761
69.5567	64.9671	81.5612	86.3671	100.572
25.0769	26.7859	16.8497	16.3309	16.3513
4.55121	3.76749	17.7382	16.6852	12.6895
141.867	169.185	111.629	108.383	120.446
8.1694	8.598	20.4739	17.1847	9.54745
2.56808	1.68704	3.79176	4.33472	5.44025
4.26787	1.86215	1.49382	0.714789	0.968535
4.94352	7.90054	1.46317	1.45127	1.33001
16.9285	13.7191	26.3042	26.1528	20.3317
86.9841	71.1463	55.1535	57.9538	62.7887
12.0913	10.4028	18.3354	19.099	19.7315
48.6223	40.3069	66.9703	68.1631	73.2041

64.3485	51.9897	45.6718	48.5139	44.2266
12.3333	11.4284	8.11607	8.0332	6.90816
1.67307	0.319964	15.5721	16.3016	10.6181
3.68202	4.28587	6.03698	6.25587	4.82274
8.96268	8.89813	4.61498	4.92088	4.90112
5.39277	6.39884	8.7012	8.86077	8.10398
16.6222	18.9082	13.2935	12.062	12.5829
2.20823	3.67356	4.51473	5.26822	5.6287
5.89671	6.09211	4.02054	4.14012	2.77193
76.2516	59.955	97.0426	99.6465	87.065
48.3001	44.8249	26.636	27.106	25.268
20.7012	18.3176	33.6779	33.3144	29.9643
53.2239	47.3091	33.957	33.9836	40.6379
14.9147	14.792	28.1342	23.1607	21.5523
11.3643	9.73789	15.2656	15.2695	11.9368
24.0857	27.2536	32.8359	39.3191	39.6379
8.89109	5.67976	2.90797	3.90301	3.58627
76.7015	116.257	45.4747	44.1221	51.9226
38.1646	23.9939	51.3038	54.3866	43.8323
47.3229	42.9639	61.7964	62.1369	74.9085
15.9427	18.329	10.5347	11.1452	8.67162
0.565872	0.081046	3.95539	3.85624	1.52718
25.3344	18.2142	6.87797	6.1584	10.6507
14.5349	12.2954	21.6311	20.7054	27.1524
6.22842	6.54538	10.4063	9.67067	10.8935
2.63538	3.02226	4.69254	4.52788	5.02202
14.0514	18.0604	10.052	10.4848	8.97531
5.07213	5.49629	2.65165	2.96021	1.99987
8.44816	9.95417	5.87177	5.30992	5.14139
38.2086	35.4173	31.6949	29.1976	24.1729
29.9776	29.8437	16.7741	16.6488	15.526
12.3868	22.7498	0.188741	0.0319562	0
17.6114	16.0047	39.2531	41.6704	43.1138
0.877712	0.609453	0.314082	0.146821	0.179963
10.3571	11.6593	14.191	13.6953	13.4068
18.4952	17.7588	27.2345	24.1893	20.2233
501.056	572.167	640.643	647.947	679.6
65.5154	78.4517	46.6028	54.7257	55.236
13.0815	11.0108	22.6533	22.7491	21.9809
60.8785	52.395	75.8087	72.0722	70.6256
10.8833	10.0572	15.807	16.0686	13.3509
3.44599	3.09711	1.40558	1.16826	1.76557
11.2654	10.647	8.36151	7.00747	5.3811
54.1742	58.1319	46.3615	42.8093	40.5409
2.78295	0.510516	11.9614	18.7903	5.90344
3.55273	5.87663	0.85767	1.09365	1.99953
0.616661	0.504686	1.97006	2.07592	1.23895
0.769015	6.04092	0.177837	0.0398916	0.333515
1092.49	897.969	769.383	772.135	688.634
8.74064	6.86986	12.0866	11.6543	9.96656
7.41904	7.60887	4.65267	5.23263	4.01695
62.2883	91.2734	34.8142	34.7999	41.2291
178.289	195.386	259.338	252.786	262.85
234.917	251.309	346.127	351.417	354.748
26.9299	18.371	37.8206	34.654	30.0652
49.8725	44.855	39.1945	38.4216	31.2592
68.1678	74.3503	39.6979	35.4631	46.3566
36.0967	33.3001	46.781	49.4506	47.724
184.798	251.684	279.966	261.255	338.938
75.0435	64.2161	83.8944	81.702	97.4655
11.7685	9.98989	3.74763	2.50235	5.65535
116.923	103.41	58.6593	56.4748	46.4815
8.19172	11.6194	6.52764	5.16279	5.40814
37.9408	26.9336	26.3699	23.1539	19.8949
16.6547	16.8532	21.4386	23.0149	19.7554
14.1836	18.4067	8.33548	9.53854	10.0168
6.27155	5.11701	13.8287	12.1206	11.9277
0.0746853	0	2.35023	1.16061	0.583665
51.7855	43.6235	55.1827	61.8583	58.5011
34.4802	46.5442	26.3248	27.1253	22.4434
16.8195	17.516	13.8501	12.8654	12.2323
130.051	178.751	58.6683	52.9826	68.0057

7.89716	6.16957	11.5107	10.575	9.36357
49.5793	39.8434	35.0639	32.6635	29.8138
5.86273	4.64765	3.32995	2.74173	1.98813
90.1591	90.0621	118.84	115.543	138.263
112.265	97.7368	85.584	79.8066	80.2788
20.1616	11.4867	46.6565	44.4339	34.2484
42.8384	33.5084	58.4487	59.0852	42.5718
55.1318	45.4433	119.696	116.849	99.9781
22.1977	20.8986	39.3962	35.4682	32.4665
5.46711	3.76515	2.88419	2.96755	2.39713
2.59945	1.90411	1.04664	0.895327	0.209368
112.149	136.527	165.155	159.055	176.385
12.7248	14.9722	3.17044	3.76793	5.00002
37.2151	25.1824	11.8262	7.19983	8.5506
83.7337	64.3027	38.3995	43.5591	42.6379
0.564892	0.566901	5.14094	5.24494	5.95878
89.7294	72.0377	104.23	111.477	115.655
506.034	595.116	294.166	300.151	366.857
116.545	98.7206	70.8609	64.5086	57.6878
2.64851	1.94974	5.21547	5.6395	4.11463
14.7995	17.1479	26.3	27.7658	30.219
53.6765	39.9353	61.6053	67.0996	56.4743
1.76965	0.379412	5.6238	6.60972	4.8291
46.9517	49.3307	67.7492	68.1043	56.6545
8.6651	6.86713	4.43222	3.40386	3.18142
34.5699	38.1861	54.6911	50.1325	56.7566
87.7141	35.1632	170.783	171.936	119.098
8.24542	10.5045	4.24817	5.32784	3.99026
96.8032	60.8659	167.783	164.816	156.634
26.0376	19.3356	14.8871	14.5804	11.5742
27.414	28.7069	21.915	20.0851	12.8
15.4789	25.3425	5.01101	5.88136	9.46098
13.4577	14.2923	8.28381	8.07592	9.51707
5.0732	7.35715	1.86908	2.17869	2.64218
20.1945	21.3812	28.0314	28.8168	25.9725
12.9051	10.1092	17.1162	17.3021	19.8491
29.6424	27.1968	17.7658	18.5475	15.3924
19.8671	17.692	28.0032	27.7585	23.0307
12.6472	9.31579	18.5883	15.8011	13.8851
2.97873	4.10812	2.62552	1.98397	1.99636
16.7706	15.7378	22.2821	23.5077	21.3587
85.0849	63.3062	45.7209	46.903	43.3149
18.9715	19.6483	9.80025	11.0441	9.58724
241.419	219.695	135.145	133.938	142.567
579.602	628.693	881.57	842.19	977.075
21.5499	27.927	11.9179	13.1843	18.5944
16.2645	14.2506	7.98128	8.87255	7.21674
60.6903	49.6032	80.8823	85.0422	66.2977
10.0938	9.25278	12.7945	13.0329	11.2371
45.0696	35.1116	31.7322	32.41	23.7094
75.8273	68.1175	44.4259	43.4296	49.7409
105.463	88.0128	80.6052	76.3289	72.706
103.31	100.323	79.2634	75.5771	76.3383
1.67778	1.84817	0.756137	0.7107	0.663353
0.952551	0.831879	2.16437	2.23966	1.53089
94.6856	98.0181	127.381	123.095	129.008
51.7834	35.5941	64.9865	65.8215	59.3358
87.913	85.6006	63.7174	59.6724	64.8841
9.03217	5.71784	2.85347	2.32723	1.76726
8.55459	8.16594	28.1938	26.3253	38.4453
2.5221	1.75467	0.599255	0.676413	0.407999
11.3003	22.0211	7.17011	8.12337	6.2913
1.62149	0.697078	10.8897	11.112	7.65354
0.336694	0.101286	2.3659	2.15235	0.657634
91.9372	79.169	58.6593	56.7883	51.8062
17.7426	16.9987	21.7511	23.5129	24.1457
57.0272	38.0232	28.8711	26.3491	22.7865
59.6375	58.0839	39.2376	40.5552	38.2291
55.1763	60.274	71.2627	74.1412	71.1123
53.6684	53.6087	75.1296	71.2046	64.8299
185.92	135.577	219.379	226.991	220.334
187.523	130.371	218.902	226.057	215.701

38.356	30.8997	54.2849	55.4564	51.4993
9.65313	17.537	44.4322	49.1606	61.5362
42.7529	31.6346	25.8562	24.6728	23.1986
153.654	123.307	108.777	111.977	105.365
33.4155	35.9127	20.0238	19.1936	16.4833
7.59372	7.0176	4.29914	4.6331	4.03955
270.089	311.415	224.12	223.659	240.419
51.5905	59.6025	34.7103	34.8385	32.2849
30.8411	38.1787	19.6102	20.8559	18.2394
53.2299	54.8693	22.3749	24.5474	29.2048
72.114	76.232	99.5979	100.599	98.9239
79.8003	56.7257	51.1747	50.1784	42.9589
25.0831	25.8024	16.6855	13.9719	13.9265
43.8798	42.4273	24.3373	24.8374	24.6243
13.5103	10.0254	6.47875	7.13127	7.62969
71.3771	26.9625	210.225	209.118	147.305
399.321	382.021	244.297	236.82	157.922
7.61608	2.42815	0.651005	0.872765	0.85213
14.2791	22.008	10.038	8.58668	12.0651
5.2761	4.50459	1.79576	0.98388	1.73846
87.7602	43.5052	168.005	170.896	132.476
0.140237	0.0140546	13.2094	14.0205	7.5998
0	0.0862094	0.289191	0.176061	0.23546
6.76464	0.795172	55.41	56.5845	30.8422
101.344	78.0428	67.0131	70.5841	52.3677
144.857	130.516	81.1582	79.6776	80.9222
236.982	247.739	193.147	187.757	165.71
58.9574	41.91	32.5923	34.2827	29.9014
3.6954	3.76993	6.81144	6.31136	6.80314
38.2994	32.8118	25.7873	22.6099	24.2844
7.77644	7.41225	6.49021	5.18577	4.44397
969.239	782.425	517.267	536.03	584.018
11.9268	15.1281	2.09979	4.266	8.84481
8.76529	16.0903	26.1935	43.908	42.5318
240.229	287.578	190.35	193.649	198.598
467.828	335.933	712.472	732.337	623.469
365.634	151.096	124.014	128.418	57.1262
14.0812	15.5738	6.88293	7.65305	5.78783
39.3	41.0917	27.2462	25.8821	29.7896
33.7677	23.0668	17.7314	20.0042	16.3859
28.6753	29.2284	18.4251	17.7245	15.6048
27.6361	31.6699	39.8814	37.9087	38.9597
86.8749	74.1434	53.5761	55.2956	59.6938
19.065	20.1425	25.1643	24.0202	25.4483
18.6553	13.2408	31.7278	29.7481	28.425
4.84387	6.72029	2.27107	2.78192	2.18815
27.7634	33.4476	87.4894	87.9557	163.084
0.363279	0.0910989	3.47838	2.98501	3.81723
123.857	57.7961	425.275	435.927	444.276
21.1687	16.1052	94.1432	89.3875	51.8708
3.68801	2.24334	1.73312	1.22778	1.98309
11.4303	12.332	7.45396	8.82736	7.68458
32.117	28.695	25.721	23.9166	16.6479
32.7422	38.7399	21.4487	18.641	26.4555
9.64492	4.75173	33.8076	33.6043	25.3253
6.49355	4.75853	10.3094	9.01194	8.35999
2.20956	1.05029	3.14682	3.98831	3.74047
72.8975	84.2526	172.58	180.89	202.867
47.7306	52.2443	37.3738	33.1136	33.2626
31.4812	22.1743	43.1239	34.4166	37.8417
7.3061	7.60784	11.1122	10.9921	10.6941
303.794	225.536	364.888	366.091	324.202
35.2215	32.8602	46.0715	42.8384	43.0908
44.0845	41.6638	33.1774	33.7259	28.702
10.0335	8.69354	17.6817	18.1001	15.8946
47.4723	50.7808	62.5652	72.0415	84.1959
288.665	249.226	408.265	402.663	471.505
24.0534	13.4127	51.8757	48.9675	42.8271
284.444	298.211	439.429	433.422	521.86
79.1916	71.4054	42.7879	43.011	37.4464
12.6373	14.287	19.4873	19.4744	16.1226
40.0784	27.8927	52.0827	52.395	46.5144

13.2749	19.8488	9.80953	10.7931	9.10711
67.4016	82.9399	49.4497	49.1933	49.4084
7.85947	8.36872	10.9401	12.5811	10.0263
8.0052	8.46901	3.2244	3.56364	3.34237
12.4798	6.59583	1.08965	1.22996	0.902613
0.359881	0.483612	0.824903	0.842704	2.66727
81.3646	97.1073	42.4605	41.3438	49.2373
74.5629	54.6688	18.5628	17.5512	22.9346
0.460057	0.107043	2.10992	2.36986	2.87653
3.3924	3.24558	1.10377	0.590157	1.51571
8.75994	7.14731	2.71802	3.41684	3.7737
4.7056	10.9847	2.17548	2.31332	2.2228
0.615025	3.41694	0.248232	0.0840581	0.323749
0.189692	0.189912	0.541874	0.538824	0.647531
12.615	13.1873	8.16915	7.5979	6.65999
394.903	266.845	222.968	221.938	219.566
2.13095	3.22793	7.82722	6.39074	8.86334
175.288	239.398	140.05	136.306	143.891
5.70838	7.34351	9.6942	9.42039	9.07402
38.9604	33.0325	46.6833	49.3438	45.2268
35.5778	24.1131	18.7365	20.3488	16.9064
13.692	9.60825	20.6205	20.5315	18.3494
20.2555	21.2287	28.8688	29.582	32.4561
18.8302	19.9217	32.1173	29.9614	31.5716
0.229291	0	2.67949	3.2862	1.53539
14.2706	9.6864	5.15705	4.86742	4.26506
69.8919	54.7143	50.0164	47.5194	41.9504
18.5599	13.4438	23.2376	23.2325	21.0839
17.7344	18.9935	8.80388	9.98804	9.18167
7.47327	8.56587	13.7974	13.4589	12.7485
4.32976	3.90384	16.3117	18.0787	18.2386
71.189	50.4611	37.23	36.061	33.6946
3.51434	4.03097	1.4402	1.49012	1.35482
17.6101	18.5535	27.3371	25.1392	24.9614
61.6054	64.0831	86.3827	87.3797	80.2054
7.42843	6.68349	4.0154	4.27912	3.25636
68.3412	75.1201	35.7379	36.2735	35.3338
1.15761	1.25409	0.340125	0.329073	0.452636
35.1761	25.3901	23.6333	22.1716	22.6011
51.3	62.3569	68.5069	73.3734	67.8473
23.826	22.5404	15.4178	15.7334	15.9664
21.3078	16.6936	14.6801	14.0634	13.9951
47.9841	32.0458	29.6725	29.4028	23.2383
5.20272	5.91115	2.92594	3.1524	3.00682
75.3393	71.2714	56.771	54.527	46.3481
21.4991	19.0762	7.6201	7.88755	10.8341
36.1941	44.7511	26.5581	25.3554	28.3603
14.6977	20.7048	7.70753	7.49143	8.40799
6.2602	3.15565	11.0741	10.9449	8.62665
46.396	44.6047	32.6622	35.3819	34.1482
7.33791	12.3932	1.33338	0.970698	3.8358
17.1372	15.3505	28.3714	25.9537	18.8668
19.1643	14.7279	13.3623	12.819	9.26435
37.247	30.8963	22.1508	23.1652	22.4509
4.65326	5.32962	8.28318	7.36283	7.18468
0.225867	9.78276	0	0	0.047206
1.07633	2.00253	5.31405	4.22594	15.1286
13.3887	9.6351	19.7824	20.5369	19.302
0	0	2.11259	1.94693	1.28706
1.69616	1.84973	0.397284	0.261654	0.0635716
8.60376	5.71329	18.3036	13.9053	15.0781
1.10159	3.22291	0.81879	0.448515	0.493536
113.055	129.628	65.2148	64.157	70.4221
44.1574	30.3176	27.9255	28.4289	24.9367
5.51639	5.21574	6.971	7.07668	6.87473
25.4965	23.1899	16.7573	19.5021	16.9718
34.5559	29.3429	44.4677	42.3497	40.8077
3.9737	5.82615	12.8816	13.9782	9.63168
59.416	43.3638	41.2397	40.7941	37.6812
9.07336	5.91976	5.05201	5.31107	4.79439
12.3301	11.5384	19.3084	19.1694	16.995
14.917	11.6922	26.4525	26.9442	21.5094

Table S3. RNAseq in D5 in mDPCs-CAS9 cells and sgCreb3l1 _A8_4: 1007 downregulated genes and 1027 upregulated genes

83.2783	66.7335	117.36	109.938	89.1956
24.7727	25.5901	16.7183	16.2395	11.0436
4.13613	3.24823	7.93029	9.4425	7.36374
19.6152	13.5926	24.2303	27.5095	25.5389
6.77482	3.31464	11.2507	12.2351	9.86558
49.1329	34.491	74.2998	70.0825	67.3496
70.3061	53.9103	116.107	112.538	125.278
7.99394	8.08822	11.531	10.6571	9.0565
0.406061	0.135606	3.6638	3.24309	3.54695
2.84362	3.99544	1.30592	1.45725	0.82457
106.899	110.326	88.8916	80.8559	81.2869
318.056	298.043	378.081	367.901	435.117
7.85806	6.54952	5.15273	5.13429	4.95557
1.57567	0.87939	0.243961	0.270073	0.227057
7.34769	4.84803	0.618992	0.444667	0
6.50004	6.47474	2.68043	1.46292	1.4754
16.6292	11.813	24.7608	25.7808	20.2191
8.54333	11.836	4.9555	5.95023	5.02506
46.2982	61.0685	32.8408	29.962	28.3171
18.773	12.8825	11.0355	10.3268	5.9902
13.486	13.4337	8.69921	9.33112	8.72066
14.5187	12.4872	9.71142	9.7244	8.93872
4.42107	2.09705	1.79968	1.20858	0.673931
27.7422	27.1354	36.7029	38.5631	33.4564
77.1289	80.1747	100.104	98.2282	101.475
45.4535	43.9655	34.5261	32.1407	27.1353
47.9753	57.5088	71.2179	74.2436	90.402
116.499	153.168	88.8986	88.5529	99.2537
2.43586	2.31169	1.71444	1.30603	0.908747
43.6116	42.6045	23.6168	21.8607	21.7567
4.01811	3.01305	1.79161	1.55696	1.30591
8.48059	14.6362	20.1005	19.7174	19.3844
70.9332	124.892	55.9875	51.3848	50.9918
73.2816	86.2452	92.9723	93.8555	96.0772
3.91081	2.18003	12.255	12.4423	8.29049
34.8713	28.343	24.8142	24.6953	20.5359
45.5151	47.777	34.2781	34.3644	29.483
7.7092	5.30673	3.85682	1.74999	2.37701
140.348	156.852	201.128	201.377	188.112
5.49163	3.79705	3.03496	3.23574	2.18108
109.664	180.406	21.5505	21.6746	36.1626
201.134	270.728	394.631	404.915	489.704
1.90021	1.71286	4.68609	4.36853	2.66308
10.6827	4.27021	28.2983	28.5542	18.0575
67.5624	70.0075	421.026	411.629	640.06
57.486	47.9918	19.7427	20.5629	23.5601
5.109	4.33195	11.9534	11.4791	16.3902
20.0966	15.8275	25.3673	27.8131	24.8914
26.5914	25.6393	34.1616	32.877	36.9164
47.4678	45.6192	69.7973	70.432	65.5766
191.19	186.265	264.483	271.005	224.528
52.0437	69.3423	76.7831	75.0268	76.9902
28.3328	25.6627	20.0835	20.6988	16.352
51.7006	53.2579	63.621	62.6803	69.0178
105.511	134.753	71.7353	72.9893	82.3503
189.701	163.768	215.815	213.569	238.88
6.96983	8.55764	13.3265	11.3962	14.0693
32.7173	27.002	24.0166	23.9333	18.2876
2.24617	1.59244	5.13644	4.36679	2.82203
38.4364	51.8414	27.1579	26.029	31.354
18.3439	27.4375	10.7624	12.3046	12.5186
54.2743	40.6873	64.6998	64.6238	56.2958
17.189	18.3027	9.85074	9.33581	9.47093
2.57114	2.36937	0.619226	0.285989	0.443459
0.771523	3.14843	4.80995	5.54354	15.1126
17.554	14.9201	26.9918	24.4189	26.6965
100.381	83.5684	77.3967	76.0655	68.3418
18.4467	11.2111	26.7361	27.6774	20.8052
30.71	26.9647	50.2614	46.6941	37.929
34.9115	27.056	22.7632	16.344	12.1442
36.3418	39.4009	4.71168	2.4155	16.3122
2.49146	4.26259	4.86607	6.11326	8.80341

5.90219	5.50345	9.70384	10.2895	8.57977
11.5129	16.5093	9.29322	8.23103	8.67466
7.56008	12.0315	15.4972	18.8586	12.7884
27.7353	25.7758	11.3167	11.1547	10.1935
0.700017	0.1669	1.72788	2.07941	3.15592
10.9173	10.9289	5.90284	6.09137	7.08505
12.2943	12.5392	23.8653	23.6237	22.6829
8.25763	8.46931	2.86355	3.26462	4.02047
0.653866	0.795764	0.163151	0.220986	0.0911012
35.1737	48.3631	50.6404	53.3103	51.5216
234.295	192.753	302.787	305.544	325.781
40.8942	36.9889	21.0919	21.7674	18.1103
14.1829	11.2473	5.69045	6.46178	7.12021
21.3095	23.5137	13.7372	13.2652	13.5304
6.95312	6.94162	9.92233	9.65957	11.1311
6.5869	6.10875	4.89088	4.11246	3.69221
10.9259	8.83575	23.4835	20.632	14.9162
69.813	75.28	116.256	135.283	122.803
41.1728	41.1787	52.922	51.9625	52.5637
67.1899	75.2734	40.2901	37.4256	43.9567
5.83457	6.10402	9.85366	9.43531	12.5211
9.71332	6.90222	5.88684	6.05513	4.57966
17.3962	13.6322	24.6955	22.5331	20.7536
64.8389	52.6083	76.2182	80.816	79.1791
14.2089	15.7895	19.0319	18.9681	18.2498
31.4837	24.6101	16.591	16.2129	9.49451
23.5494	304.435	1.82699	2.06211	9.95853
794.712	1013.41	285.949	294.487	322.463
40.7656	45.4848	76.5696	72.2969	64.2059
19.7944	27.1529	32.7293	31.3148	39.0974
0.842596	0.654468	0.0441473	0.037384	0.0205462
7.98659	5.38038	11.8989	10.3078	9.73746
0.86155	0.464165	2.19253	2.86664	1.62789
9.33694	9.21553	6.43641	5.54569	4.61368
3.16366	0.430403	0.142133	0.0401079	0.0220507
1.81E-07	5.88E-07	2.81517	2.45534	3.43351
22.0064	21.6791	33.2947	30.5027	23.897
33.8098	29.104	25.6194	24.6462	22.005
27.9159	24.5209	14.4097	15.3556	13.5544
8.82352	5.73274	11.579	11.1993	10.5194
14.2008	16.3981	19.2144	19.0609	18.051
4.22691	5.55195	7.8258	7.59856	12.0121
0.977708	0.11596	6.3353	6.02483	3.85916
67.4318	36.9929	102.124	100.202	113.114
67.3307	68.2192	37.7164	36.4299	41.0448
39.1434	36.909	29.2294	28.3624	26.9983
5.53108	4.23555	1.90314	1.83847	2.77867
4.01368	3.58402	9.43541	10.341	5.37957
1.97685	1.06689	0.40728	0.419737	0.329606
79.3163	65.6895	42.0086	43.179	49.0019
27.1296	30.806	38.6334	34.6291	35.354
6.34195	4.8672	16.8708	19.6231	28.6488
17.9745	13.1357	27.6164	28.0048	22.3391
5.51084	7.45189	12.9495	14.9467	15.2539
478.452	415.181	221.554	216.087	169.015
48.5951	59.9473	92.9633	91.3285	71.6752
7.81756	4.9915	3.98826	3.60278	3.44953
38.8633	40.2776	20.5929	22.5688	23.3346
19.232	20.2181	24.4065	23.9131	23.2801
9.6972	5.95775	4.37239	4.58248	3.17165
74.2975	89.1829	105.5	115.336	131.831
2.04182	7.39984	0.653274	0.632059	0.435279
9.07248	6.91514	13.5715	12.1207	9.81968
7.01923	4.21993	3.30859	1.86725	2.39367
3.49274	2.73128	5.7638	5.42097	4.64111
2.94265	2.10542	4.51546	4.80085	4.85606
76.9328	58.3586	38.9497	37.2104	35.0926
7.74E-09	0	2.40128	1.95024	0.549221
74.3512	56.7886	98.265	97.0991	88.4244
55.253	43.7639	70.6517	75.7967	69.4589
2.61613	0.975337	11.0068	10.9817	3.63913
7.01754	4.92182	4.48953	4.06327	2.58176

37.7049	41.233	26.8781	28.9835	27.8703
30.8942	25.7043	19.3733	18.7656	18.7398
168.958	150.699	193.079	199.937	249.686
168.787	117.218	93.1976	81.464	95.8309
522.769	331.904	808.809	792.904	560.777
31.8649	6.80723	163.584	158.037	94.9447
8.58665	3.22411	25.7746	23.1837	15.7108
2.4157	1.50503	5.23818	5.47347	3.85764
64.6184	39.7803	34.4977	37.1471	33.8342
49.7832	57.3227	31.3545	31.465	31.6343
52.4982	35.2566	28.8986	30.2559	25.6915
12.2835	9.21957	16.1033	16.108	13.0594
41.559	37.8399	29.5732	29.1615	26.8456
29.4468	37.5523	23.8856	23.604	21.4373
5.92919	4.41325	1.25277	2.17042	1.35542
3.54466	4.68826	11.7232	13.079	22.124
96.925	110.391	51.3795	54.7905	65.6161
16.8924	15.451	5.36582	7.5525	7.10398
19.7501	16.6031	26.6345	25.4851	23.1369
661.796	787.086	909.469	912.906	1128.04
89.6388	62.0918	49.396	50.6957	40.8551
36.494	47.3022	23.7787	23.8938	20.3991
12.4596	7.87258	6.78666	6.336	6.72116
53.9411	60.1529	39.88	40.0247	46.6925
71.5447	80.7053	44.5897	46.1048	52.4424
22.3668	29.8151	11.0974	8.71374	15.056
14.0627	13.0396	8.15827	7.92866	7.90498
12.9669	11.9618	18.9075	21.6308	22.3193
0	0	10.3182	17.1114	12.1509
2.20912	2.82505	0.63794	0.852718	1.84454
34.2927	27.5234	24.6114	23.5691	18.7833
9.20767	5.30884	14.6039	13.7776	11.4672
22.9651	17.4591	5.53343	7.38722	5.3787
61.1359	66.0624	26.7167	28.2535	31.6054
4.89145	4.37946	2.56383	2.82365	3.43487
132.102	171.169	56.3065	54.124	62.7114
35.5173	43.1623	54.7408	57.096	45.6911
29.0637	25.2533	40.9618	39.9019	46.4791
36.1744	30.981	17.5262	16.9525	19.1464
1.57634	2.00665	0.115369	0.206825	0.490453
4.45764	7.61419	2.28673	1.95236	3.02284
2.82892	2.85236	1.8172	1.73938	1.64746
157.046	147.545	205.435	198.046	187.814
17.2052	3.92586	1.31483	1.8088	1.14946
385.479	334.046	275.132	262.31	183.13
32.7902	26.503	20.9227	23.3636	17.5188
53.901	40.586	29.8884	29.0333	28.1658
14.6202	14.1293	10.3124	9.17739	8.41765
164.011	163.063	237.843	234.125	289.404
13.5108	20.1628	9.72504	8.61638	8.55477
20.2707	16.2443	24.7659	27.2001	23.2736
8.63815	27.0764	1.48583	2.01864	3.15881
2.61833	2.90008	0.828635	0.830597	1.0694
25.0399	29.4328	20.582	19.749	15.5006
170.014	203.709	235.516	232.538	249.849
12.8471	17.4133	4.87262	5.62849	6.44748
29.2356	20.0688	15.1365	13.503	13.7996
83.1572	103.003	67.8878	66.9283	64.0669
2.61047	3.25526	6.77429	5.49713	6.40333
147.111	157.832	199.682	207.986	190.873
1.84966	2.31786	0.209626	0.251761	0.750951
29.1738	33.2032	16.9717	16.3774	24.725
76.9334	61.0325	33.7906	31.4389	52.6312
17.2298	14.0346	10.2862	9.89759	8.34099
87.5579	80.1324	48.3786	56.6861	57.9543
0.557064	0.186344	1.55985	1.81573	1.45381
36.3423	33.9108	20.9509	23.9615	18.1227
41.6001	33.6608	52.522	50.2284	47.2732
103.308	103.514	59.6587	57.7641	49.4238
15.663	15.6743	27.3235	27.0616	26.3743
80.4394	77.8894	109.941	108.891	109.855
112.732	109.281	141.057	136.848	146.128

Table S3. RNAseq in D5 in mDPCs-CAS9 cells and sgCreb3l1 _A8_4: 1007 downregulated genes and 1027 upregulated genes

2.93946	2.57233	4.83828	4.5793	5.85897
105.326	81.7177	79.0555	76.2884	59.4838
2.00687	2.52064	1.49005	0.889615	1.47355
26.5741	27.5595	33.1135	32.1834	41.1993
131.521	125.97	195.687	185.45	207.837
29.5256	21.9865	41.5058	45.562	40.6546
966.39	1080.66	1219.67	1237.64	1355.39
145.663	119.854	181.587	183.645	173.268
19.2717	21.7816	10.6236	12.2585	10.2609
2457.84	2258.81	1494.73	1464.47	1561.04
0.62766	1.62093	3.7706	5.18723	6.6831
14.943	13.2707	2.24222	3.12286	1.88798
9.14668	9.26469	13.7507	14.2142	16.5511
2.01282	3.74439	0.842071	0.607809	0.891387
2.96234	3.11881	0	0.219023	0.301409
20.9049	22.2482	28.1517	31.0185	43.9139
42.5761	64.0517	75.7218	87.5368	136.856
13.401	11.2103	18.3849	17.0767	30.3325
2.24916	2.90912	4.40546	4.61945	5.21327
0.755167	2.20148	6.01032	5.84065	8.84879
16.3553	18.0256	23.7394	27.5747	28.9765
21.2993	18.8782	31.3226	28.4892	35.2015
16.5749	19.9909	6.01242	6.6319	7.66797
0.0972849	0.0967202	0.442566	0.528033	0.669135
0.806443	2.04822	0.394585	0.620359	0.472307
30.3606	33.3866	3.99332	2.30853	10.2965
11.9533	9.63535	24.4124	21.9124	33.0921
7.33548	6.6287	15.1167	17.0652	15.1058
2.39299	2.83064	5.96084	3.02883	5.70359
44.1739	64.5392	14.4201	13.6679	26.6741
44.1739	64.5392	14.4201	13.6679	26.6741
1.55243	2.48385	3.25167	3.16431	4.65548
21.4031	25.9262	40.8598	38.0797	48.7289
69.3724	74.3995	133.299	126.151	115.774
55.7864	80.5749	41.5222	40.9319	41.1951
382.269	379.151	166.865	177.997	208.272
904.29	920.2	659.666	685.843	767.955
10.905	15.4166	5.77623	5.62573	4.60585
0.0625968	0	0.689261	0.389	0.564314
12.9397	10.89	20.4798	22.662	23.8207
7.03631	7.2479	2.84515	2.24809	3.00804
1.42631	7.34163	0.214011	0.0603922	0.133001
18.206	17.1141	25.6468	26.8588	20.5095
72.0016	59.3354	85.6767	85.5411	78.9283
24.933	22.4986	16.922	18.4643	15.0056
34.2095	28.9526	50.0963	47.4462	49.2525
60.3604	60.7991	37.1915	34.6403	39.1023
22.9249	16.2149	31.812	40.4764	35.5305
115.647	120.127	72.1558	71.6941	70.0864
3.30169	4.13404	1.60517	1.90426	0.786998
36.9622	33.0491	23.7492	20.7436	21.4256
141.344	107.472	241.054	216.502	161.098
5.28599	2.87084	13.0583	13.7842	8.59396
15.1242	17.8733	12.1745	12.1544	9.55626
15.3572	10.7354	22.7036	21.755	19.8397
451.135	322.095	188.704	189.912	196.113
1.58551	0.513815	0.335735	0.301583	0.0552786
23.8516	16.0345	12.516	11.1233	11.3703
3.02538	2.36548	8.7202	10.0754	7.41191
24.3518	16.1898	38.2487	40.4451	27.2547
51.1544	37.6596	18.8735	18.3737	11.0582
180.887	179.033	260.031	252.62	293.938
136.826	62.2229	36.4151	36.9984	23.7991
2.02324	1.63489	1.39567	1.33967	0.860731
729.166	841.666	433.613	430.109	579.437
3.57165	3.27823	8.98511	8.41067	4.92616
383.773	379.314	305.167	301.385	284.248
14.8206	18.3348	28.0336	27.6051	25.5885
490.376	384.11	738.602	695.193	617.728
4.83208	5.4302	2.84867	2.63546	3.61051
77.6303	86.2161	42.6945	42.9806	60.384
12.0089	12.9065	15.7683	16.501	18.6597

Table S3. RNAseq in D5 in mDPCs-CAS9 cells and sgCreb3l1 _A8_4: 1007 downregulated genes and 1027 upregulated genes

146.427	135.425	220.88	222.988	219.458
3.40717	3.02186	0.741884	0.573549	0.197191
28.7906	39.6445	20.4996	19.3667	21.5685
30.2771	24.3391	40.4965	40.5901	44.8626
13.9271	26.7356	3.91617	4.19846	11.2594
2.04122	1.70124	5.90243	4.37967	11.1392
7.12747	8.9755	12.3248	10.8089	17.7043
69.7858	62.134	114.263	107.501	105.532
51.3305	56.5251	27.4741	30.3638	29.6397
77.6471	70.3226	109.82	107.868	97.0093
25.5029	25.0041	35.4906	36.2289	33.3992
28.4827	21.3033	43.836	40.7786	33.7512
5.57108	6.36189	2.38059	2.14956	1.78009
31.9911	25.8574	51.6809	51.0474	44.7189
4.52104	2.62796	1.183	1.2701	2.56495
23.8077	13.7408	49.8406	43.8894	36.1322
44.2394	48.7298	58.0958	51.9937	70.2206
34.928	34.7947	43.7434	43.4619	53.1689
29.1304	26.6891	19.0305	20.2041	17.5653
4.029	4.86307	1.52852	1.15282	1.37125
11.7104	15.7424	17.6699	17.5005	22.1274
122.039	137.545	156.075	152.756	178.857
11.3838	6.54283	4.96535	4.50644	5.41661
117.249	113.363	148.496	152.298	154.014
35.6094	30.329	63.4932	64.9665	47.4872
31.9546	21.1023	20.216	19.7158	17.7132
9.34254	10.2131	12.9303	12.4542	12.1959
47.0602	37.3626	27.7251	27.5002	22.6812
38.3898	33.634	31.3932	29.6284	24.373
16.7813	15.5684	24.5621	23.4437	20.4389
8.7857	7.44365	6.26643	4.61249	4.66599
1.79201	2.19991	4.40678	3.78618	3.39101
15.0208	12.1783	21.5601	20.5102	15.9505
0.659711	0.702616	0.0348193	0	0
14.6648	10.426	9.55642	9.72079	7.97156
66.5019	47.5934	28.1213	32.1082	17.9309
2.40166	3.16447	10.2022	11.5042	8.77178
8.37602	11.2505	6.29612	5.11339	6.94923
27.4339	22.8557	35.4904	33.3474	32.9604
33.8292	31.2072	15.5899	16.448	13.9037
11.8357	11.0777	15.6531	15.1239	13.7543
121.258	121.88	169.36	170.178	178.592
14.1364	9.11242	20.3588	20.7864	18.7726
12.3155	15.2614	9.27657	8.34131	9.18642
24.9476	25.5383	32.3633	33.4148	37.4241
59.4794	56.8587	42.0802	41.2915	39.9364
25.504	36.4059	40.5944	41.7352	48.1444
192.149	161.155	99.0332	98.029	95.8063
49.7958	40.8864	73.6786	74.6096	62.8953
176.23	141.536	247.111	224.591	218.928
16.9826	17.2906	9.96889	8.47645	6.90424
170.778	208.144	110.332	105.463	146.743
502.516	476.825	634.999	623.863	553.741
11.3706	12.746	5.9518	4.92271	7.32731
13.8477	16.7417	8.87082	8.81897	10.5352
16.215	15.3622	2.81292	1.42884	3.3835
3.75047	2.31415	0.508158	0.280751	0.634894
6.09703	4.41515	2.05174	2.49437	1.98487
9.36576	6.40317	4.13439	5.00873	4.65147
5.99249	6.32975	2.36854	2.13868	1.77104
24.2715	15.0882	5.76444	6.92953	3.88543
8.77865	1.53812	0.788599	0.532956	0.72871
7.25485	2.62477	0.952927	0.355789	0.728872
5.7508	4.67661	0.900688	0.626328	1.01833
2.91155	2.71712	0.286676	0.10401	0.304626
84.7621	77.2716	35.7237	35.3092	37.8249
140.454	165.893	96.8547	93.7094	112.09
6.98188	4.59371	10.2297	9.84097	8.22756
209.328	169.025	138.289	133.418	155.647
107.245	135.534	73.8043	70.0161	99.4307
15.818	23.4678	11.0357	10.0877	12.8546
24.9783	20.9657	11.4842	10.6642	9.09696

Table S3. RNAseq in D5 in mDPCs-CAS9 cells and sgCreb3l1 _A8_4: 1007 downregulated genes and 1027 upregulated genes

34.5689	32.7883	52.7324	53.3463	57.8859
33.6141	23.5666	42.4708	40.3448	43.355
18.9928	25.226	10.6502	12.1814	14.8653
93.5474	111.491	65.356	73.2361	72.1665
17.7937	15.387	9.91926	9.5321	12.1127
26.1285	25.7673	34.3921	35.4781	51.66
0.237456	0.0396912	1.03418	0.694551	2.09833
2.97861	1.67797	4.84379	4.27885	4.15088
6.86215	10.1165	13.6275	10.55	12.3084
1803.06	2125.13	2688.38	2762.63	3068.22
31.8407	96.1616	158.313	161.175	140.106
10.6458	30.7588	41.9511	43.3432	39.323
121.361	157.911	204.721	214.007	306.801
149.974	149.064	95.5457	97.3284	88.7219
17.9918	16.8947	23.8193	22.2553	21.0147
16.2058	16.0706	9.52588	8.26868	8.39865
57.3762	211.896	10.0691	11.4916	30.4053
13.6189	10.9337	6.85958	8.83109	6.04168
1.20441	0.855951	0.699181	0.632113	0.365142
79.3926	70.4081	94.4184	95.9819	86.6979
47.9827	35.839	32.8145	34.538	29.0373
58.8718	43.1208	70.8505	71.7429	61.9614
26.1561	32.7695	15.9884	16.0987	21.3249
9.97279	12.699	4.52079	4.9234	3.85747
9.13073	11.6652	3.99287	3.92382	3.24269
23.8026	23.0151	15.2606	14.623	16.1073
38.8541	28.5873	52.2331	47.327	51.6801
16.2573	16.4438	27.7049	27.0611	27.2987
20.9515	19.2423	29.3348	26.1773	22.8179
30.7981	33.0308	44.9396	43.0796	41.7285
9.56239	6.24268	32.2118	33.4009	25.9019
31.1295	34.5965	18.6667	22.5553	16.8684
13.0653	19.1954	9.71508	11.3861	9.24912
37.9472	37.1834	45.5683	46.6236	45.6269
22.8035	24.9861	12.346	11.4485	10.7337
19.2257	14.0996	8.45541	8.39026	6.61493
3.39189	2.26634	8.24135	8.2363	3.87392
66.1239	76.184	94.1385	89.0791	98.4135
398.855	483.556	1453.37	1465.61	1232.56
21.1521	31.8696	14.5635	13.9732	16.2331
21.4155	21.4526	27.7352	29.9128	26.8775
15.6539	7.93148	30.4235	33.2897	33.0271
64.5729	53.1926	35.8712	36.5989	32.8817
68.735	77.3455	41.915	38.2333	33.2642
2.78661	2.35523	15.173	14.1679	12.6202
0.052317	0.131204	0.277754	0.510499	0.459133
0.452906	0.743553	0.206388	0.172009	0.248726
1.80841	0.323149	5.88508	5.94738	5.39687
60.4365	54.9049	75.649	78.5021	66.4267
605.167	512.353	342.039	330.377	280.377
5.24041	3.44732	13.9255	13.1993	10.137
335.639	330.93	441.604	435.822	512.808
80.3994	65.3037	89.875	89.0698	97.7121
13.1455	9.63838	8.59476	8.78564	6.22787
42.6652	40.5297	31.9893	33.8779	25.7748
9.94185	10.0613	21.6358	20.9779	20.0744
12.1532	8.15775	21.2604	19.7467	13.5967
7.23897	11.5236	2.98392	2.47701	2.23861
5.97442	4.594	10.6144	9.561	8.08156
86.2755	83.5731	113.926	116.758	98.6692
41.2457	66.7934	16.4149	19.5482	33.5626
3.26874	3.80758	1.53346	1.12323	2.40746
115.066	105.316	83.1988	86.6372	82.4842
18.2363	18.77	10.4136	12.4133	10.5307
13.1949	9.32488	8.18499	7.41759	7.07973
5.65645	6.93157	2.71186	2.31301	3.37664
49.7684	57.5832	40.2759	34.0457	36.7226
3.40651	3.85012	5.09081	5.03216	6.66168
9.26061	6.20034	3.23982	3.7937	2.40186
0.686948	1.04034	1.56896	1.80724	1.89154
8.79408	13.2716	3.78217	4.12349	3.42806
18.1936	14.0965	13.3199	13.4567	10.6628

35.3162	30.9982	53.0958	53.3733	44.4099
15.9725	13.8454	22.8299	22.2328	21.3956
4.72997	4.1057	8.23089	8.69098	6.94996
18.1481	21.6079	9.32156	9.52098	8.89279
5.37436	6.43988	3.04866	3.40788	3.65333
56.5107	64.1637	37.7032	40.2063	29.3227
57.4904	46.367	73.6414	71.397	60.7318
8.70807	8.80908	15.4554	14.432	10.0737
20.3509	18.5994	28.3135	25.8645	22.6904
12.2266	13.5877	17.8398	19.5793	19.7161
8.92845	8.62177	6.33367	5.9792	5.75433
20.0187	22.4152	29.2445	31.1575	30.158
48.729	52.4747	61.7914	60.2481	65.5073
43.7831	30.7192	66.065	58.1706	49.9171
32.5406	36.5456	22.7846	23.7595	28.34
17.0529	15.5182	12.1052	12.8504	8.93674
0	0	1.73356	4.02961	1.26843
11.2943	13.5715	8.65194	7.48866	6.17267
34.3151	31.5554	26.6411	26.9863	23.4368
24.9813	17.6379	13.6445	14.355	11.6673
15.2917	12.1586	6.29895	5.97231	5.36909
33.8521	29.8808	24.3978	26.1487	24.1313
6.10938	5.28296	2.39315	2.71811	1.88891
1.47766	7.32792	0.0971023	0.0822055	0.27177
61.3823	53.7339	12.5724	16.3865	16.6235
53.8381	55.3369	68.3692	69.9947	66.0071
35.6606	32.1374	44.8061	42.9013	39.8707
2.74775	5.02034	6.90091	5.35961	6.66299
23.4839	27.6657	12.6654	12.5431	11.6731
0.425392	0.697547	1.22565	1.44917	0.951921
1602.74	1182.2	895.473	883.624	849.116
417.215	407.245	562.32	563.207	639.083
32.8125	31.9764	23.9939	23.6931	22.3938
100.064	80.2559	134.992	133.776	116.213
71.2039	69.1837	92.5334	92.1391	110.684
51.1973	57.2974	36.2184	38.3688	33.5788
4.78441	6.1528	2.1173	2.31527	2.63302
36.6749	38.9833	63.7344	69.3184	58.7766
0.388701	0	2.34092	2.00425	1.09858
15.1981	18.5319	8.25613	7.13685	13.2493
36.0939	39.6375	15.8123	15.7813	13.3492
109	165.378	258.307	252.919	255.182
34.103	28.2837	22.0907	21.7031	18.276
23.7988	26.588	55.5295	57.7207	41.4021
7.41238	1.33946	0.600449	0.660041	0.700806
102.694	103.132	144.039	143.274	126.365
0.1151	0	0.845864	0.664935	0.788253
37.4361	26.9809	60.2314	55.3429	66.1814
3.49501	5.61567	2.71938	2.54975	2.14699
45.2544	39.4295	27.2454	27.1438	24.0889
5.73442	5.44343	4.38513	3.83616	2.32961
144.207	124.246	70.0097	66.9927	99.3837
30.8414	30.7974	124.41	122.876	105.784
8.79046	7.75526	13.502	11.851	10.7219
25.3095	30.8782	21.8547	20.5291	19.5426
8.43886	7.50614	13.1282	13.3237	9.85116
38.3041	26.7295	51.5649	51.6429	45.1172
24.7395	21.1528	35.2236	35.2732	28.2113
49.2805	13.434	7.36789	7.0406	4.55424
91.9975	72.696	121.379	117.763	114.689
15.0293	10.2703	19.0053	19.4932	15.0451
15.1287	10.2746	8.87031	8.8242	5.12029
6.18684	5.47791	2.04323	2.67512	2.30584
36.0191	37.1843	22.6066	23.2581	20.7291
11.4466	14.0863	9.13835	9.14037	8.05727
8.29932	8.64605	4.19275	3.81034	5.46297
17.4811	15.8969	11.5143	10.2182	10.7622
47.5356	32.9661	30.4881	29.3806	27.418
21.55	27.1314	16.9984	7.74956	12.5249
183.39	201.297	133.908	137.999	135.375
14.2227	18.3613	7.32225	8.59537	7.90757
67.0124	38.8071	18.2238	20.4726	39.7393

23.0961	23.1796	9.22474	8.8813	8.60003
45.5264	48.0029	80.1852	78.9009	79.6304
1.03247	1.81441	2.89402	4.05786	7.592
39.6233	28.5538	59.9248	66.5563	52.6405
6.27188	4.28934	11.4425	11.1223	12.0901
97.1088	95.3647	63.4363	63.4742	68.4535
22.4394	21.0882	29.9386	34.008	33.4084
28.257	26.4819	21.9522	24.3044	17.6002
4.5749	4.38366	0.837746	0.796787	0.791496
15.6344	13.8505	11.707	11.2577	9.29489
69.2661	64.1392	94.4756	95.0964	97.2187
29.3494	24.7142	9.69988	9.19434	7.49921
4.84104	3.31476	7.48139	7.73358	5.28539
1.68457	1.8332	0.786214	0.954833	0.487546
44.2549	51.8751	28.3184	27.904	28.0269
0.0276567	0	0.144907	0.110404	0.215758
3.05297	1.74702	1.57024	1.42313	1.16517
7.8599	4.13266	12.0345	11.0277	9.75959
0	0.0617225	1.44743	1.79975	0.58595
67.9505	63.5327	95.0191	89.4214	89.4193
25.5337	23.4825	39.0639	35.8394	34.9185
0.127725	0	1.16139	0.60504	0.624097
56.829	75.8181	37.7407	39.9074	40.2488
19.975	26.1557	9.79742	8.33134	13.3749
12.3044	12.1041	21.9422	22.9722	23.627
170.981	165.189	77.1166	77.7231	93.7272
53.0526	56.1855	69.1064	70.2308	63.9723
35.923	32.9673	22.3554	19.8191	15.7497
1.26228	0.613656	0.305889	0.441249	0.219539
1.51992	2.31974	2.80578	3.0581	2.62355
54.8538	43.0276	38.6135	38.3101	33.804
3.78036	4.7032	8.24429	7.72738	9.11366
10.5877	8.72256	12.5485	13.1379	12.3688
4.66376	4.58167	3.13329	2.44886	2.19936
4.22273	2.89999	5.39705	7.15438	5.53336
35.1834	48.5424	28.8104	27.9305	24.3141
90.4328	80.6945	62.3874	59.5641	56.5496
20.5016	13.1169	28.2112	28.8206	23.4149
1.08521	1.47103	0.482201	0.428779	0.50205
151.103	119.352	81.0234	80.0937	72.333
12.2108	11.8749	19.0293	20.6932	19.3678
58.2632	66.1613	84.6866	85.2142	98.7855
2.385	5.25395	0.588883	0.747793	1.00574
375.841	283.004	269.056	264.665	211.478
205.816	196.764	164.507	164.23	154.523
76.7094	79.3312	47.2215	41.5997	48.0397
10.0157	8.87233	6.43211	6.21096	5.54812
1.0181	0.751252	3.39042	2.92535	2.18899
41.7259	34.7501	53.8695	54.0605	47.25
61.8565	69.0268	45.6036	43.0615	53.0435
16.1912	12.8991	24.8856	23.4852	20.953
2.12532	3.38511	0.703661	0.45956	0.773159
35.6176	34.6148	52.6828	47.2715	57.7851
37.2389	31.1629	47.4616	49.2621	48.4816
40.0406	33.3044	50.1417	49.5757	55.135
34.7955	26.382	44.8733	42.7459	41.351
10.2367	8.71811	15.6609	14.8044	12.797
2.22856	0.734447	0.13239	0.596886	0.103743
45.271	48.7834	61.5366	62.1287	58.7183
10.1823	13.3675	16.0238	14.2269	18.2008
1.02638	1.31023	0.355474	0.318089	0.215656
0.990364	1.21638	2.76759	2.75327	2.60526
16.0622	101.205	1.07899	1.69539	7.6182
20.6324	13.5722	30.4611	28.4289	21.5273
4.49908	4.88087	1.75426	1.75534	2.22499
44.02	57.737	35.1161	41.0275	31.522
35.9111	23.7434	50.6981	56.156	44.3924
8.92885	10.5404	17.1737	16.8414	15.3972
17.1961	15.6555	24.0895	24.6618	23.6181
33.1195	28.2784	49.9514	49.0299	38.8043
26.4154	20.7332	20.2953	17.767	14.4973
15.8375	40.2451	7.19671	6.8966	11.9492

Table S3. RNAseq in D5 in mDPCs-CAS9 cells and sgCreb3l1 _A8_4: 1007 downregulated genes and 1027 upregulated genes

2.32609	4.95768	21.0739	17.1503	34.6416
8.41134	6.57449	3.90145	4.66812	3.60071
0.112115	0.0374051	0.586483	0.690363	0.341999
21.8402	13.2066	37.4679	27.6355	23.0376
658.018	838.514	483.693	469.751	540.609
5.28925	5.57567	11.8912	10.444	7.66765
151.822	170.553	117.439	113.828	105.294
4.69006	6.71356	6.99798	8.23893	9.26787
1.76271	0.773619	2.85013	2.71371	2.86893
7.96888	7.40738	3.77218	4.36635	3.59861
34.289	35.3569	24.0794	19.587	19.5623
39.733	37.6468	19.3863	18.7458	20.2382
8.1154	7.49463	4.8864	4.27206	5.31287
26.192	36.4792	18.4309	16.9611	20.0776
53.0772	48.6046	42.3629	40.6451	36.6804
4.4773	8.32036	10.1043	11.988	13.3725
10.8054	10.5253	15.3623	15.594	16.952
43.2977	42.3611	65.2478	53.5481	61.1444
87.6894	111.721	61.2703	55.0834	69.0606
111.646	82.66	141.549	137.821	136.755
57.5663	41.7726	87.6409	83.0392	68.6984
62.4977	59.7378	90.4138	92.5544	101.408
63.8324	53.0091	81.7054	82.3657	90.0132
97.3627	69.5435	135.27	147.421	123.631
40.6238	36.6292	60.5487	56.8793	52.2839
62.5862	49.1654	34.1185	40.3863	46.8944
85.3085	75.485	111.073	101.997	110.019
24.7315	22.3628	31.3584	29.7601	29.5456
27.9442	32.6917	17.9055	19.0596	18.2345
18.2405	14.2445	23.7367	22.7839	21.6228
0.107995	0.180408	0.998713	1.06999	0.589192
5.43082	5.05226	2.61235	2.00668	1.78093
3.89194	5.02673	0.204221	0.0432217	0.190257
3605.11	3500.07	6050.33	5797.66	5079.64
1157.12	1084.94	1549.85	1428.64	1438.19
1151.55	1641.03	551.805	569.15	808.501
72.24	59.243	98.546	96.5471	90.4656
83.8626	81.6089	101.498	94.9937	111.768
35.4859	37.0048	14.4719	12.1523	12.7131
16.7262	16.0567	12.2316	13.0585	11.6787
7.96215	8.20104	10.3682	11.9818	8.56235
15.8263	11.6289	21.4013	19.8873	20.4978
11.7618	18.1102	25.5725	24.946	23.0638
375.319	293.651	495.018	488.701	488.126
139.041	128.002	176.317	183.753	176.927
1057.74	1082.46	1264.16	1364.34	1546.78
7.87364	7.0704	13.5769	12.4599	11.5392
13.7103	11.0678	9.36427	9.75936	7.4534
9.74408	12.2887	3.43717	3.13481	6.71554
22.1857	24.6984	15.1229	14.0113	12.36
5.21848	8.86927	2.44195	1.67613	3.25606
5.65876	8.18803	0.818927	1.05661	0.865193
62.4533	48.6912	37.786	37.0728	37.6001
8.48533	9.67997	14.6501	13.1636	14.7639
27.0035	37.4471	17.9612	15.9369	18.8998
19.1693	17.0418	6.6322	7.54456	6.23995
4.11543	3.79676	1.30919	1.47869	2.69387
2.65476	4.26014	0.942658	1.2333	0.838076
40.2849	45.831	76.5843	67.3311	69.8856
3.67466	3.76968	2.09866	1.25718	0.861135
25.9159	28.6005	8.87684	10.0303	10.0187
74.736	74.9043	189.574	190.409	142.892
38.2049	37.1443	48.2764	53.0372	49.3058
62.71	55.472	79.1745	76.1989	69.7641
5.77378	3.76938	7.79552	7.55284	8.47286
6.72027	6.08006	11.503	9.59868	8.88049
3.23602	4.34723	6.96503	7.32548	12.9354
104.885	63.8599	31.8096	46.0718	36.1208
112.911	115.693	44.225	42.7196	52.1605
28.1502	20.7501	37.9426	37.9125	39.8761
136.666	105.921	83.5641	78.7541	82.2848
173.603	182.094	129.412	147.127	138.426

156.674	144.22	222.148	218.392	193.924
243.145	177.21	318.001	303.466	371.529
56.9999	44.4277	69.4539	72.9586	77.2018
28.7473	41.5514	73.9016	59.3608	66.9848
68.6612	54.3882	85.9839	85.885	75.7002
38.992	37.9595	57.9702	63.705	55.7938
72.7164	54.6108	89.2665	86.4075	82.0305
4.02194	3.0483	5.99169	6.36984	4.474
29.1531	24.0742	42.8763	40.709	31.9912
25.2059	22.0499	11.4844	9.65376	8.71522
43.5632	44.6976	61.603	60.3428	48.8727
51.8092	40.783	70.1409	67.612	62.4317
8.726	7.06203	2.60417	2.46491	5.09379
7.70447	5.57332	4.59504	4.00214	2.85429
36.5883	31.8856	22.5208	22.0642	18.4437
40.6725	31.3002	54.8179	56.8653	53.1496
6.66147	5.05197	4.90655	3.44805	3.57714
1.73056	1.87677	3.11866	3.1212	2.88699
60.4053	44.1646	86.2659	86.0141	69.0163
37.9258	38.3492	28.4641	29.118	25.4283
0.760041	2.11116	0.394399	0.155772	0.29355
14.7172	2.37825	49.0417	47.7259	45.0948
21.1452	15.5582	13.8509	15.0879	11.3336
40.1621	31.3034	27.0604	22.305	23.3983
13.2841	15.1792	19.3086	18.5604	17.5578
2.62208	2.01123	8.49695	8.06968	7.20706
6.27073	8.48343	9.07163	10.3734	10.3517
16.2889	15.9072	23.5142	23.0198	19.1979
24.538	24.0394	36.2234	35.4966	31.4485
4.69367	2.87137	14.5562	14.3238	12.5415
21.3786	19.0316	25.5135	25.9657	23.7818
194.116	228.846	299.945	283.398	357.65
3.43288	2.76826	0.57931	1.58027	1.07984
31.3583	29.692	58.5542	68.9607	58.6343
3.61964	3.77881	0.535524	0.248164	0
2.56171	4.70994	5.93252	7.19737	6.76461
4.25118	3.8465	2.12614	1.94474	1.61393
88.6106	71.0001	102.722	108.534	102.803
57.5165	66.4309	44.888	40.4465	39.2547
8.06058	7.4179	11.7944	10.7977	11.1969
17.7043	11.0854	7.6628	8.48174	7.12918
43.193	57.3047	29.234	22.1682	35.665
60.1793	57.8399	77.4729	81.4655	78.2204
15.7996	14.0277	9.69794	8.47879	6.49277
454.271	500.783	208.709	210.455	279.614
75.7429	72.2048	62.1392	53.251	54.0672
15.5984	14.7648	10.6759	10.9097	10.9106
60.9115	71.7985	121.115	116.96	133.563
7.78724	9.98501	1.32454	0.615777	2.92455
17.7709	14.6743	8.99318	9.05342	7.92793
33.4563	32.6814	23.461	22.8637	18.4647
9.2586	8.62408	4.70217	4.78513	6.42613
57.2133	44.9645	40.247	35.7715	33.5515
18.1429	20.9176	35.3731	37.2671	43.1574
103.618	94.697	80.7154	82.1831	71.8689
21.8707	20.1984	13.4394	13.756	13.5616
40.8916	37.1164	49.1345	50.1023	46.0846
79.7942	62.5942	55.4968	57.0111	50.2326
89.8254	84.8719	55.116	51.0123	48.286
213.154	221.822	160.707	182.294	110.418
176.186	170.87	120.36	113.235	85.9909
44.5695	23.7627	78.2111	75.5551	75.214
9.94968	9.1194	19.3071	15.2023	11.961
47.6356	35.0361	72.0952	70.7053	68.4189
131.459	133.869	107.002	106.981	80.7086
1.2113	0.395402	8.35902	7.72653	5.55491
11.7548	10.442	7.54317	6.53165	7.44595
73.5249	63.1533	109.333	103.921	100.964
1.65145	0.962732	3.02003	2.87059	4.05763
148.242	93.763	85.9938	85.6076	62.948
3.41093	4.94878	1.9368	1.80856	2.089
0.155797	1.01437	1.55714	1.35315	1.91093

16.168	16.4335	19.6581	18.5781	22.4933
10.7489	6.00056	15.4253	17.5975	18.557
90.1359	89.412	49.6255	50.7501	57.4413
0.5323	0	10.5121	10.5608	13.3552
80.8461	66.8482	113.492	113.575	106.91
10.3107	9.56532	6.52924	6.73355	3.57339
114.023	96.5771	150.274	141.371	118.593
13.7774	11.0223	2.49219	1.12226	1.86651
3.83941	1.83157	9.75371	9.14406	6.08357
7.89902	7.93836	2.837	3.24142	5.18228
15.932	14.2579	28.2635	29.4369	18.5944
16.8659	11.3914	29.3996	25.912	20.4128
41.844	33.6822	21.2069	18.3729	15.7499
5.27056	3.20185	14.4516	15.1638	11.4589
35.7384	32.5808	58.1324	55.2422	45.5392
16.5384	17.8981	22.9259	22.7352	29.0066
48.8514	43.4556	61.2184	68.0921	62.0935
23.8783	29.6142	32.7973	33.0724	30.9495
25.6356	23.1756	49.0934	48.9038	40.791
6.63788	7.3871	2.94928	2.49686	2.24153
3.65293	4.03479	7.10708	8.51533	6.39685
0.275584	1.96411	3.96614	3.99053	6.6698
36.6667	29.7251	51.6338	51.5488	46.5587
2.16616	1.47541	0.70061	0.665266	0.80745
3.34061	3.261	1.64654	2.29109	1.42677
9.07152	8.5414	5.29923	6.87384	5.74787
3.80724	5.07565	1.84779	1.3812	1.48196
1.35429	1.34676	0.682267	0.651854	0.622195
2.5592	2.62037	1.02765	0.869966	0.983305
2.90486	4.1988	1.53585	1.67696	1.89676
5.9877	7.13854	3.45576	3.41651	3.5419
2.71801	3.77167	7.36894	5.89801	5.10925
0.845199	1.79033	2.98895	2.81871	2.28145
27.666	28.1938	16.7965	18.4028	14.9238
83.0165	80.9651	132.388	139.3	152.336
22.1923	28.9158	16.7939	14.8815	13.0883
16.7537	19.0721	8.73111	10.8453	7.22396
7.7277	4.61419	12.571	13.5172	10.0483
0.343572	0.1722	9.05018	9.84131	6.95847
0.137512	0	1.44436	1.773	0.605912
0.55089	1.07701	2.13717	2.39602	1.02174
2.99573	3.07748	1.80105	1.95473	1.82
28.8692	27.7271	51.0035	47.7767	57.1711
89.4438	100.666	135.818	132.619	119.549
18.5046	17.8584	25.4099	27.2418	30.3925
12.6031	15.8901	26.0523	20.9312	23.3768
43.6989	45.1941	60.6302	58.0531	56.1872
3.81147	6.84647	2.68028	2.48858	2.6232
8.06892	7.4636	5.15775	5.75102	3.18467
79.7642	65.0615	111.357	119.084	122.397
6.76831	6.01353	11.2602	9.31557	10.6705
67.0616	83.7104	48.7075	48.601	50.5114
8.68342	8.64932	12.2015	11.4377	13.8704
4.17093	7.63761	20.5175	16.295	14.1884
84.1018	41.0859	138.346	135.951	122.245
19.6593	15.9775	14.552	14.5081	12.5335
5.40376	7.04479	8.00097	9.01923	8.30442
32.2157	23.5045	47.3297	46.5704	39.3552
1.87383	2.12569	3.87729	3.47929	3.58535
81.5819	30.9416	26.603	26.6937	20.4565
2.45351	2.28287	0.70092	1.24685	1.02993
29.4777	31.7398	22.8265	22.3947	21.0831
3.02945	3.10194	4.66013	4.44569	4.93232
25.5792	21.8877	11.4258	13.872	11.259
3.76947	3.41866	2.32208	3.03049	2.0157
37.6536	10.0261	6.52371	6.32019	4.33742
15.3163	13.4648	9.24303	9.07033	9.28623
12.7237	8.58875	4.96035	5.75319	5.87484
10.738	8.6375	15.9419	15.6584	15.3305
41.3212	31.2417	53.316	52.2332	43.2056
14.6189	16.6336	6.92881	8.98959	6.75793
13.6755	21.3558	9.50175	9.17391	9.59649

20.6121	18.0467	31.566	31.0267	25.1644
9.66632	9.59012	5.55152	6.3583	6.025
38.4481	25.0187	54.8753	58.2949	53.2412
29.8935	39.4832	47.3301	44.0201	64.0512
25.2682	21.7029	36.613	34.4208	33.9106
17.7512	21.9014	10.1638	13.0409	11.5362
30.8267	28.8983	42.837	43.0836	34.7031
34.3433	30.1906	47.6961	46.4449	44.2165
15.5433	13.7509	5.43545	5.88888	3.7342
26.2863	21.6172	13.2552	10.9713	11.944
12.8154	9.68593	3.21876	4.4404	6.04931
0.268646	0	2.7136	1.66952	1.44548
146.246	172.069	93.358	96.7596	114.376
34.8842	31.7283	45.7591	46.4164	37.129
16.3266	16.3723	24.4534	24.1237	20.4138
16.7668	17.2118	9.18585	9.37364	9.1287
19.9369	17.337	13.5344	12.5465	11.3755
25.7958	44.4493	13.4256	11.9544	17.1757
16.6493	14.218	9.50426	10.3074	7.39865
11.2992	9.23516	7.74747	7.67147	6.60567
17.5202	12.356	26.0371	25.9872	25.7851
3.95324	5.58319	2.79552	2.64404	2.6
0.553197	0.849892	3.28126	2.94209	1.69165
27.6294	21.9188	32.9307	38.4015	33.9612
41.4299	52.307	57.5536	57.9051	65.3223
269.619	278.458	177.769	177.696	154.67
45.3418	43.5166	60.1621	61.3778	55.8468
19.6468	13.9365	29.8894	29.4342	28.8332
52.8631	53.0354	74.9288	76.7045	79.595
3.763	1.94008	0.603427	0.510843	0.562233
10.0046	3.7508	2.76059	3.10781	2.43891
16.6179	16.8908	27.6785	27.1299	25.7821
105.579	83.773	135.838	126.113	120.714
66.2623	37.2288	104.061	99.7552	87.465
14.1177	11.5257	7.24812	7.21168	6.5704
64.3477	71.5606	54.5889	53.9197	45.78
48.0282	35.1593	23.9645	21.4653	23.2014
20.7965	18.566	15.7398	16.054	12.2296
32.4508	38.751	43.7743	42.8435	47.6085
133.502	126.071	96.144	95.1401	91.7758
9.92572	8.33812	17.5612	16.2455	15.951
70.0929	69.7679	39.601	40.5993	48.5619
136.353	139.306	202.588	200.527	212.025
12.8724	11.6121	16.7676	16.3619	17.5378
24.6574	22.3334	31.7385	32.46	34.7444
14.4904	13.1036	7.18929	7.25107	8.18075
90.3227	115.566	57.0909	55.7307	66.289
114.687	101.86	78.5316	74.7078	94.0152
1072.21	365.213	3308.04	3311	1500.49
21.0063	23.9115	11.2042	11.5764	12.0335
721.163	570.876	841.309	821.654	888.522
13.4561	15.3441	24.2047	24.1733	20.24
237.735	209.094	315.85	303.777	319.835
71.6206	55.0564	119.9	118.021	94.8885
61.2692	45.6699	42.9765	40.7219	38.678
98.7604	92.671	57.3562	61.4294	56.0508
119.648	115.338	164.867	156.822	157.648
0	0	2.28398	1.58953	0.479264
62.9458	56.8909	38.9232	42.9098	38.7466
20.8912	18.3426	33.0068	30.6007	32.8361
72.2559	53.7152	108.754	103.688	83.2823
45.9899	53.7272	69.7612	73.814	54.8481
94.1919	105.071	78.9559	74.0293	76.5203
95.1242	99.1097	129.365	126.734	133.791
26.7695	22.2729	20.3209	18.6983	14.8853
12.2999	8.86889	4.65814	5.1411	4.41361
26.6268	20.6974	17.0614	14.8199	11.5634
228.856	228.682	165.33	150.349	173.068
132.07	141.435	185.27	182.977	226.212
185.253	146.05	251.516	241.328	215.653
9.20753	9.98588	16.8597	16.5186	13.7021
162.232	124.616	100.572	95.449	93.8699

9.13724	7.38801	14.104	14.6876	14.5436
6.78654	5.54318	10.7471	10.6269	7.89169
78.661	77.6804	126.152	118.655	108.767
38.0864	31.9213	22.5052	21.824	22.8116
5.61879	5.24074	11.1726	12.0156	7.30921
30.596	21.9106	41.5595	38.0185	37.286
20.8813	20.7654	28.9477	29.0757	25.5247
12.5964	10.6133	8.09488	8.28314	6.48806
13.7821	13.4749	7.89511	7.01809	8.65612
22.3681	24.8859	39.0012	34.9073	55.2547
61.0774	63.628	80.8361	80.9945	89.1853
16.0407	20.0828	12.6713	12.5908	12.6846
45.2572	51.7967	35.4969	34.2095	28.8494
2.22429	1.25111	3.27071	3.33914	2.37575
0	0	0.504615	0.655301	0.158075
3.68194	2.9326	0.323447	1.50592	0.453527
6.06016	7.88139	2.22085	2.6925	3.4313
38.2003	44.1176	52.3528	49.925	55.3341
31.1156	23.5865	19.4764	18.6454	14.9114
51.7715	59.6713	65.322	65.9929	79.2154
243.326	267.616	336.351	330.601	389.134
292.752	303.714	359.103	361.964	405.688
14.7326	25.8441	28.9714	30.032	30.9035
260.698	233.73	317.89	298.955	331.622
266.384	214.214	342.69	337.89	331.787
227.741	229.59	291.964	288.406	348.218
127.293	142.058	190.862	185.793	203.48
251.361	191.044	361.135	337.757	345.652
21.9869	35.2754	43.8387	38.8116	52.0514
100.737	109.627	126.911	120.594	140.859
128.861	119.951	160.581	161.674	170.567
185.962	213.691	109.937	107.028	122.486
140.803	203.988	248.432	249.882	309.698
11.0315	9.82319	20.3193	19.4019	16.0473
7.55961	4.37035	32.2674	37.0267	20.1668
33.2701	31.7983	42.093	42.9236	38.7497
2.62264	3.89138	1.37486	1.39667	1.9199
3.91724	2.44605	9.53462	5.95555	7.37773
77.3652	92.7	52.8589	48.7132	52.0315
3.18077	3.56266	2.16314	2.53249	2.03969
27.5017	30.8186	40.025	38.85	32.9834
5.1377	2.92214	8.11816	8.86761	6.35747
0.85689	0.404772	0.0705576	0.12251	0.137074
257.928	248.062	201.65	193.085	204.421
83.4486	96.2387	224.27	237.292	139.721
0.122183	0	0.88431	0.248084	0.635507
51.5561	28.3026	19.2034	22.8608	11.1364
8.04164	8.05352	2.13344	2.5223	6.2684
4.098	3.54883	1.40774	1.93835	1.91748
6.06125	5.33306	4.30871	4.0731	3.36358
23.9347	28.5912	16.9906	16.3717	14.4845
48.1905	63.2827	76.0646	77.6541	99.1873
155.759	190.011	130.712	127.736	134.776
113.468	84.6714	155.68	150.607	138.397
38.8009	38.2772	29.9166	29.0027	29.3908
6.2354	8.45982	4.34879	3.68151	2.26177
23.6141	18.297	11.7995	13.387	9.55173
601.559	576.604	469.989	468.21	440.853
13.916	18.9177	4.30303	4.69883	7.62264
24.3129	18.9598	32.9479	33.5148	29.4804
50.7129	29.169	22.2901	26.1419	21.3505
21.281	16.9666	28.2807	27.5702	26.6496
5.53911	7.31262	1.00329	1.53297	2.17931
34.9582	42.7144	6.13581	5.31612	6.32992
5.3303	9.95622	3.04813	1.99782	1.37604
20.9739	18.0852	11.2982	11.1875	10.9137
75.6424	57.1685	95.8459	92.934	83.2488
14.6491	10.5988	20.6549	23.2168	23.4387
4.77483	2.77751	7.58154	8.48495	7.80242
7.77458	6.47235	10.4641	11.8893	11.148
7.94975	6.03762	4.2915	3.75342	2.97652
48.8101	70.8467	22.7008	23.0738	26.9777

0.522803	0.671182	1.44313	1.7682	2.86894
19.3942	19.2925	24.7817	23.0378	30.7987
0.086701	0.0724109	0.351363	0.229017	0.533231
35.7571	35.8981	24.9962	26.1092	22.1962
22.4518	22.497	11.0715	10.7187	10.9878
40.6775	49.4104	55.3617	55.0355	54.4013
50.595	55.1454	36.8778	40.9819	36.7731
12.7636	13.8852	8.7612	6.36552	7.8707
8.17624	8.27803	5.55846	5.51016	5.79388
15.7866	19.7364	41.3218	36.1291	39.1715
74.4343	51.6512	39.3736	38.7213	34.8415
0.136951	0.275442	2.74108	2.44251	1.61743
86.1212	119.434	66.6122	61.1364	60.4899
92.2446	117.265	66.7273	74.0065	67.8806
93.9685	79.6076	60.3791	59.1486	59.3593
33.252	35.2071	13.405	13.0792	14.1203
161.152	180.899	119.915	130.137	118.934
0	0	1.00277	1.50652	1.12159
43.0646	45.0593	27.4135	28.2349	27.1567
9.41423	7.30175	5.53788	6.19395	5.73368
11.7801	9.23865	4.13456	7.27272	6.6244
4.46457	2.24499	5.29154	7.71452	7.55361
4.29766	3.46667	8.8795	9.58091	8.3603
2.72462	3.22019	4.39659	4.34495	5.1615
29.6897	39.625	45.8019	42.6869	56.3422
21.89	19.8344	9.08487	7.99851	8.03417
26.7077	30.6044	37.7933	36.4277	35.2227
54.2574	54.0397	77.2649	75.5797	85.1764
30.4135	22.7962	20.4649	18.5459	17.1156
15.0815	15.1058	19.32	20.4494	20.1134
26.9751	19.9182	34.4117	31.6207	30.8835
35.4333	32.5987	25.0102	24.8594	21.5323
293.053	221.197	505.472	517.72	315.363
8.87073	11.8102	3.01225	3.58377	3.69901
119.796	115.984	224.304	215.687	237.625
25.7377	29.7742	37.1732	35.5324	45.3749
1.69831	1.08708	3.75221	3.94653	1.83537
22.669	21.5042	7.10689	8.76317	8.47923
8.53285	9.15967	15.7935	17.7935	16.1287
5.17599	4.69199	7.33157	8.23694	6.53082
11.1841	13.5159	5.49726	5.78744	5.10524
5.8834	5.77036	4.6468	4.99652	3.98281
19.1765	10.4292	31.1535	30.7593	27.3159
18.7833	13.0922	23.7278	23.3415	22.2916
22.5891	17.7687	14.8812	16.8828	12.824
91.4486	98.0189	55.0223	53.6941	61.6461
16.0306	12.7276	11.049	10.7152	6.97957
6.5964	3.69725	9.34115	9.61549	8.33364
6.87022	5.60327	9.39718	10.5648	15.5597
119.772	87.1412	166.012	169.086	168.883
0.338671	0.446994	0	0.0440211	0.0625839
16.5655	15.7292	8.6005	6.84084	6.77996
481.103	437.441	580.481	597.995	637.034
833.174	955.988	554.801	547.162	584.933
797.5	911.483	1026.37	1050.71	1123.07
460.16	464.02	583.026	584.959	662.75
1116.07	795.141	1553.15	1598.99	1510.34
624.013	808.564	975.727	968.072	993.681
468.311	475.73	659.499	681.58	691.756
1800.02	1661.43	2396.33	2326.74	2713.45
861.38	884.598	1158.32	1233.23	1363.46
61.7129	61.1724	81.8686	81.9169	96.5665
37.3709	37.5445	57.4093	56.763	56.9739
210.493	208.092	270.652	301.847	294.753
76.7414	57.7344	100.508	112.257	102.912
1422.87	1239.06	2083.44	2123	1932.08
0	0	9.75882	9.69873	11.8913
14.1243	12.4848	4.62195	4.08004	4.71265
14.7392	10.8123	8.58854	8.50242	7.93048
13.0813	12.8257	7.66584	7.97034	7.76491
6.65377	5.20741	10.0105	9.45531	7.31216
38.176	29.5441	50.0819	52.8902	45.6795

7.12296	7.37248	0.903688	0.859474	2.39469
75.0446	47.5444	45.6648	44.8536	36.103
23.0915	19.9052	17.2709	16.3185	12.8224
43.323	42.1217	51.9944	52.2645	58.1457
6.56541	5.60013	4.02031	3.8142	3.62313
0.137553	0.165489	2.19223	2.14887	0.671366
8.66143	11.4755	4.25279	5.37571	3.19199
16.3268	15.2613	11.3091	9.82734	10.0317
32.0226	23.1445	18.2111	18.2622	15.9306
42.79	50.4981	55.4488	60.6796	57.5542
752.008	511.039	1078.88	1097.06	1061.17
31.2287	26.8137	10.2826	11.7583	22.6595
15.835	12.3297	20.0341	18.9901	19.1688
2.28391	4.1081	14.2302	12.9369	17.161
45.9414	55.1008	36.3088	34.9599	38.415
10.8109	8.72498	13.7688	14.1248	11.6135
88.2261	76.5379	46.901	48.8378	47.0887
120.746	82.1269	64.1399	68.2589	57.1056
32.9612	26.9877	50.0664	46.2491	39.5318
10.5001	7.9287	15.9699	14.6201	13.7552
7.02934	5.7202	4.14096	3.877	4.09096
6.33577	25.0001	0.426459	1.60362	2.83202
0.296052	0.269828	6.26455	6.01996	8.43298
15.3595	1.18678	0.689581	0.291886	0.765633
23.2631	29.1825	13.964	13.8763	16.8323
6.49839	7.14953	11.1135	9.91092	11.0565
1.89047	2.25854	4.24639	4.45833	3.72675
0.774853	0.214802	2.06307	1.81906	1.01388
74.0178	79.9011	121.666	114.904	94.9017
119.162	103.64	177.235	167.248	151.164
6.01529	5.9209	11.9159	11.3075	12.0445
73.6297	79.0748	55.7681	54.6304	58.2753
19.9258	18.4237	29.987	30.4113	19.8811
6.84956	6.83081	11.6961	12.1378	10.9272
69.3458	56.6422	90.7997	89.9445	72.524
14.318	7.87074	20.9612	18.2957	15.6335
29.4739	29.1726	15.3936	16.2964	21.9696
2.02526	2.67854	1.02157	1.70093	0.940486
22.0982	16.425	11.1059	8.79673	13.2947
1.84529	2.08093	0.461586	0.744301	0.961586
9.05913	3.70387	3.04937	2.09458	1.61879
1.2427	0.593319	2.45106	1.68098	3.06202
120.636	108.338	170.652	166.486	146.441
4.50241	2.9917	2.20505	1.82897	1.65328
17.4992	15.2352	29.2746	24.3878	23.7293
250.464	255.534	299.45	301.611	349.388
570.231	393.806	322.377	329.866	228.505
64.3453	44.2487	102.999	95.3655	82.9584
53.8722	52.8754	39.005	39.889	37.7621
1.62563	6.62028	0.535253	0.399814	1.2394
36.0303	45.173	59.077	57.6959	49.4878
10.803	11.3065	14.1623	14.3804	13.7624
89.5811	90.5264	118.687	113.415	113.827
127.863	123.502	170.719	165.549	142.827
95.8444	101.616	67.0682	64.7297	63.0672
199.453	122.379	299.546	293.495	253.469
28.2232	31.839	16.1127	15.3951	18.0778
24.7825	25.4596	36.3539	35.4618	37.6853
44.8992	45.6452	29.6935	32.1599	35.0581
171.496	172.896	107.717	104.925	112.135
38.2269	47.1985	26.8985	26.3124	28.9452
6.94151	6.85965	3.32246	3.0258	3.50407
8.7068	7.04898	11.0046	12.2239	12.7427
1.24368	0.276965	4.35653	4.70318	1.77484
5.28911	6.37628	2.50452	2.23823	2.43202
183.332	143.314	237.814	233.403	253.502
21.3657	33.3286	53.1317	52.9677	52.7582
8.51476	6.89161	5.13283	4.80453	5.07124
47.7463	41.1171	76.445	77.7293	84.2447
16.8789	20.9528	7.34715	7.88945	12.5148
35.6102	37.105	22.7824	23.0501	21.4869
111.828	100.893	134.944	137.023	123.873

Table S3. RNAseq in D5 in mDPCs-CAS9 cells and sgCreb3l1 _A8_4: 1007 downregulated genes and 1027 upregulated genes

25.1044	25.1337	11.4279	13.3376	13.8526
30.6737	25.4195	40.0973	42.1498	33.8656
3.39543	7.46081	0.672258	0.870786	2.00913
73.9446	59.6988	93.7478	87.9625	87.288
17.0063	14.1396	28.5669	24.712	24.4323
5.63562	5.72917	0.441772	0.186993	0.359863
5.51231	5.59382	7.99644	8.56447	7.03026
18.0164	13.3044	23.8632	22.1463	32.1749
111.622	82.7134	76.5766	67.8246	72.0749
11.6086	10.689	19.0844	17.6712	13.5723
13.0106	12.7877	8.65163	7.88884	6.18695
13.15	12.8686	8.61507	8.07561	9.13426
128.802	108.023	192.213	197.044	157.605
40.1573	25.3064	99.045	89.5218	58.6794
5.52421	7.20397	8.00727	8.32066	8.12059
36.9947	38.7913	56.4459	52.5161	51.292
12.2372	15.3171	7.46147	5.57782	6.61839
35.6593	35.9589	16.1866	20.9363	18.185
23.6577	16.8758	14.353	15.5325	13.7755
37.0193	71.1086	21.1973	19.4544	27.9467
6.6703	5.90879	3.83002	3.86874	3.9229
15.5868	14.5329	11.086	11.6888	9.98303
2.89044	4.52935	1.00524	0.81702	1.42088
31.6409	35.9533	15.3991	15.5778	17.2038
17.6368	19.0459	28.3597	22.6916	24.3207
9.9184	6.57514	5.82813	5.50383	5.84476
4.1012	4.54008	8.22102	8.46892	7.66756
293.292	270.713	145.655	151.304	162.477
4.62706	1.61618	34.5588	33.9258	25.7402
32.0199	19.6779	51.6602	53.7169	56.1878
0.271219	0.357656	1.07703	0.962412	0.542903
18.8089	23.7236	29.7847	30.7183	29.667
16.7557	22.1212	13.3424	12.9178	11.2179
40.2795	36.1063	21.6485	21.5903	19.6017
14.5168	13.8754	18.5013	19.758	17.1846
123.322	94.2732	74.0987	70.476	65.3992
27.4095	22.636	14.7915	14.6692	12.8816
24.3155	27.3677	17.6503	16.9643	16.5528
38.6529	29.8487	23.3183	22.4186	21.8415
50.9215	55.4935	71.0563	73.4833	57.1982
1.75621	1.70406	0.782769	0.921327	0.733194
13.8845	13.6644	10.4766	10.2194	8.34591
2.40683	2.20519	0.970046	1.03492	0.95304
3.80903	5.23786	2.0083	1.86671	1.18602
4.54642	5.74653	2.30883	3.04366	1.85982
18.6866	22.7151	10.4978	8.46822	10.2935
47.3987	42.2986	31.9902	31.292	29.7591
8.06154	6.27957	3.98098	4.27499	2.74484
3.73789	6.46824	9.90879	8.98178	11.1522
22.5181	19.7045	13.205	14.9972	12.7214
24.3273	17.5139	44.3986	41.7189	37.6347
32.8364	28.7773	47.1605	44.1075	33.8721
10.724	5.46037	20.1178	23.9578	17.5254
34.1556	23.8701	43.9632	44.3803	34.6834
57.3002	67.1943	32.8909	32.0725	32.423
23.407	21.8698	27.7589	27.3369	30.5262
63.1041	81.8465	51.4626	50.795	45.121
48.6117	41.3752	59.9501	57.3705	55.9622
21.4642	17.2138	11.1886	11.0423	8.64299
21.7228	28.5006	49.2203	48.441	95.166
35.7246	30.1012	59.6801	54.2279	48.6113
23.8805	30.4921	7.37836	7.76193	11.2217
32.5868	22.5605	47.9142	46.6534	40.9525
72.6789	55.1599	118.267	114.798	93.4819
6.80708	6.45678	10.1393	9.02971	8.63482
177.516	160.637	143.947	137.984	112.165
13.5521	31.5862	58.2557	60.0539	62.0985
21.0036	15.1561	40.0584	37.4179	31.8164
264.922	252.079	186.213	186.027	215.476
37.0373	28.0159	48.0807	46.1076	43.5302
40.6985	44.7741	71.7047	53.8849	78.5113
200.871	152.979	253.438	266.663	259.067

22.982	29.8756	18.0473	17.3468	12.4571
24.9926	25.7217	15.858	15.78	14.3413
30.2727	39.3932	18.5931	17.1132	14.0369
26.8428	33.0132	22.5923	22.2126	20.9682
18.7333	13.8175	10.4182	11.8422	9.32626
21.3101	23.0477	8.52411	9.44125	10.7114
23.8412	36.2183	39.4221	38.7681	42.5848
14.5196	7.77692	25.4057	26.6777	16.855
14.5514	16.0961	6.30333	8.00155	6.00576
14.7067	15.0531	30.7421	29.9363	27.5013
102.285	94.8607	170.715	163.189	174.793
2.64982	0.905262	3.9172	4.50704	2.88496
4.1454	2.69316	11.3383	10.6262	5.28851
2.19979	4.48349	0.400805	0.0988217	0.72149
1.29997	1.0714	3.27409	1.95437	3.01257
1.05368	0.477167	1.67547	1.9616	1.56018
18.249	10.2176	32.5521	34.5266	58.365
1.39548	1.71236	0.244384	0.137927	0.303847
26.5567	21.3989	34.4055	33.4488	27.9166
58.477	82.8441	34.9028	34.4718	35.8046
11.4251	7.76113	5.60909	5.58153	5.97551
38.904	40.5884	53.8035	52.4523	51.9267
10.9666	14.9875	5.98396	6.00059	9.25197
0.290771	0.0763447	1.47086	0.936683	0.8431
17.8479	8.82377	39.248	37.8442	41.409
16.2053	17.7573	29.3262	29.6612	25.4164
36.0236	44.1202	46.8118	46.1611	49.8066
13.1192	11.1973	7.0399	7.50707	6.72238
2.8306	6.15883	1.10408	1.16833	1.67003
0.684116	0.955895	2.15128	1.98674	3.43847
68.438	69.5525	92.4173	89.5759	81.0916
174.98	197.551	152.219	146.157	136.367
28.9247	19.7752	66.111	60.3311	62.7285
43.6068	36.5772	33.0176	33.9262	30.9544
2.94465	2.09301	5.64372	5.38534	3.68807
91.6651	80.2758	124.81	112.749	99.6586
52.8659	63.0348	33.7694	27.0831	22.3933
28.5	27.2535	21.5325	21.0343	17.0206
55.3369	57.1456	76.3221	75.1622	66.2503
436.319	351.532	673.8	616.524	673.4
31.1175	19.9951	39.6211	51.7625	42.2081
80.9213	62.5973	41.404	37.8879	40.3563
61.2594	74.0002	51.6023	51.4181	45.0684
17.2908	19.2563	22.8743	23.5097	22.0891
1.16299	1.35107	0.472207	0.336631	0.393298
39.3739	36.1428	56.329	54.4027	45.5646
14.0534	12.6215	8.03537	7.98436	8.51873
19.0963	15.2562	11.1737	12.4141	11.7646
105.106	87.8287	64.2257	69.5304	66.912
12.0226	11.8487	16.6506	17.061	14.3346
13.6555	13.709	27.1299	27.9359	23.0165
52.132	71.3339	84.7983	80.4577	103.038
53.3581	39.5283	70.731	68.6944	62.4268
30.7741	26.4062	47.3804	43.4496	48.7487
32.8631	23.8709	44.2896	43.3782	34.8621
1.03319	1.14645	0.266613	0.478499	0.315296
8.34749	9.45863	6.01395	5.59466	5.70358
37.1191	34.1654	51.3113	52.6944	49.3261
23.6264	20.852	18.088	15.8931	14.809
3.10271	6.39228	8.34774	8.07839	10.2015
0.268008	0	1.83235	2.17752	1.47258
11.6559	9.2571	20.3954	18.9822	15.6952
15.5019	13.6136	22.5223	21.0779	17.8766
3.87183	5.94131	2.77041	2.75049	3.04626
134.086	86.1444	188.032	190.596	159.28
3.13637	2.13025	0.236303	0.228622	0
8.18743	6.6067	11.9616	11.5511	14.7877
9.44729	8.65442	4.08138	3.81736	4.32104
14.0634	11.2431	21.1139	20.1744	17.403
9.28854	4.82999	3.41234	2.85218	1.28402
33.6759	43.2472	26.2616	25.4592	24.1293
78.0167	99.8678	62.1874	61.4988	68.706

0.250416	0.107129	1.37381	2.44437	1.36986
115.269	89.0041	85.8517	81.2275	70.0667
12.2861	11.2211	1.04787	0.452995	1.69013
35.0438	45.038	30.513	27.9752	26.1711
2.27405	2.75725	1.3647	1.54582	1.57428
18.6469	19.6191	11.1982	12.1341	8.89667
7.42938	5.43716	4.23878	4.29836	3.53867
182.26	236.197	339.764	323.347	298.224
4.1556	4.7221	2.20807	2.59617	2.44014
24.8188	21.758	30.6857	28.6787	29.2316
28.1173	24.1383	20.7328	19.6384	13.9154
42.4927	39.1608	34.0706	35.6171	26.555
11.0552	20.8787	3.40595	3.95594	2.99953
20.4236	27.4386	43.4783	40.4095	37.8231
182.791	185.055	232.184	238.079	253.742
40.0589	33.3195	29.4305	29.8199	24.7913
89.8285	56.7889	43.2988	42.3478	34.2362
45.0077	41.3101	31.1578	31.3112	27.195
0.266161	0.0935263	2.30669	2.58642	1.82783
39.0285	36.4061	53.1758	49.8815	44.8706
91.178	102.842	123.681	133.746	127.348
9.7899	8.65828	13.2264	13.9511	14.5162
184.973	223.729	102.221	96.9569	109.224
0.0419022	0.0106763	0.879578	0.76023	0.388471
12.2878	9.81658	7.88347	6.58822	6.84693
0.585044	0.413365	1.60652	1.24441	1.73753
2.06565	2.17407	1.62329	1.5536	1.25033
13.9588	12.0176	9.99907	10.0941	9.23723
122.105	137.196	56.5974	41.7653	61.6242
42.3761	44.3905	57.0132	52.6319	67.18
6.10402	9.92937	3.17283	2.82562	2.96974
14.1163	33.551	49.4785	49.0476	41.1737
35.0661	23.1472	20.02	22.615	14.7439
16.3356	21.0562	48.1184	47.294	42.7205
87.0939	148.913	287.11	273.609	250.98
39.4943	40.4861	70.3573	71.1143	47.7279
15.9537	10.0576	8.5308	9.17238	8.4725
5.95518	7.23945	3.24398	3.50731	3.40896
12.8801	6.56193	21.134	16.2655	18.3193
1.80832	1.19133	3.75738	3.32367	3.134
23.5978	20.3781	28.3738	32.109	28.8012
44.4233	34.2886	28.277	28.4188	25.3001
216.639	196.33	241.394	271.347	319.301
20.2264	25.672	14.2492	16.4154	15.3272
9.54484	6.14486	5.22317	4.5665	5.33945
3.92959	3.1762	5.77905	6.43643	5.61177
105.208	76.5521	139.474	138.746	109.333
6.43472	9.66582	3.62371	3.85735	3.89093
67.1422	45.3997	39.689	36.328	42.0113
37.4794	38.0552	52.7811	51.3819	44.5823
39.5244	38.4671	54.4265	53.2918	44.7194
1.94063	2.68013	1.39693	1.39579	0.725537
122.838	116.088	95.3923	90.9823	92.2307
30.3743	25.0838	14.963	15.23	14.0801
9.16966	9.55048	4.87443	4.74554	6.01882
62.0358	57.4794	90.255	85.4565	88.9612
2.70361	0.945189	6.17309	5.45835	4.23943
7.93855	8.87666	3.43001	5.42031	6.11958
70.2063	89.8648	48.027	47.6956	47.3974
89.3805	87.2618	52.5337	47.5167	51.3527
48.3138	47.2441	29.9887	27.285	25.5267
58.9347	44.8188	76.745	75.504	63.1701
177.003	103.464	87.902	86.5766	93.4876
32.8115	29.5888	40.2576	39.2441	37.5557
10.7351	13.2878	7.48247	7.34614	8.22821
12.1909	7.71356	5.27274	3.72988	4.63719
1.00266	4.4277	0.0639108	0.123395	0.549647
7.72326	5.50045	10.2682	10.6307	14.6298
7.93486	5.74603	4.90062	3.6103	3.48799
4.24306	5.27357	2.39328	2.42245	2.20573
2.14156	12.0734	22.4223	21.5579	25.2142
30.3196	26.4523	19.1805	17.8364	20.1866

40.4736	34.9239	68.9567	65.4726	52.5376
47.0034	36.8043	23.6324	23.4961	20.0953
51.6692	43.3958	22.149	22.9921	31.7342
58.1051	62.2984	34.4681	32.9605	26.057
6.79898	4.729	8.68546	9.77587	7.93274
13.105	9.76005	6.92973	8.41123	4.60977
100.089	97.9632	141.319	130.35	136.992
16.1969	25.4856	10.0404	8.54765	10.1969
5.08845	4.91374	6.37181	6.2732	5.58372
2.08583	0.574269	0.257442	0.181618	0.159853
10.3491	9.06643	15.4276	14.9492	25.0803
6.63226	12.5832	18.8093	15.0917	20.0498
102.066	106.973	79.1483	76.863	83.7166
0.0329248	0	1.3812	1.43235	1.80056
113.363	94.0596	160.609	156.636	122.529
6.32482	4.47477	18.2328	16.9605	9.64356
58.031	64.0745	77.4844	82.3024	71.793
28.5276	25.5178	38.6846	39.9433	34.6772
28.0829	26.1495	39.4249	39.8771	36.3496
39.7325	38.2214	32.4507	30.6482	28.7552
7.92886	6.98352	1.96469	2.3951	2.56464
5.20541	2.0624	10.4313	10.8485	9.59633
34.5716	50.3916	25.0907	23.9436	30.7309
87.0757	81.6268	109.415	108.87	109.04
8.80565	6.42578	13.1296	13.5939	11.5959
7.04891	6.18457	0.953356	0.836885	1.49803
5.01201	4.61033	7.5932	7.60971	5.65665
24.1538	22.5916	31.4658	33.052	30.6693
12.7991	26.2137	4.27036	5.82291	7.8214
32.6864	30.8492	20.3623	19.2266	18.5837
24.659	16.7136	11.4593	10.3162	12.568
1.49628	2.60922	9.15144	9.94937	15.118
34.1313	33.5315	21.4846	21.93	20.5245
38.8143	32.2134	23.2806	21.8047	19.0169
196.64	195.477	265.53	256.56	304.666
46.7359	43.9749	66.2988	67.8029	66.6643
28.296	25.1925	37.9695	40.1363	36.6382
20.2038	20.6429	27.1676	28.235	23.9833
9.32638	11.0382	2.35708	2.40402	1.91057
25.931	28.3368	16.1057	15.601	15.935
910.09	857.777	509.909	511.749	548.181
18.4917	12.2571	7.08185	4.85438	4.48193
59.7689	57.9193	49.9655	40.184	29.6253
51.2518	52.4083	83.9666	81.5049	74.7153
16.6436	11.5559	8.36982	9.2523	6.32233
29.2858	32.1054	16.8924	18.2343	17.1266
148.099	110.271	174.876	172.099	174.008
21.8955	18.3031	14.5984	13.8472	13.6022
87.2892	88.6423	71.2154	71.2122	58.5948
10.8843	16.9713	18.5277	18.9987	19.4961
27.8726	29.3854	20.8896	20.2923	19.1952
41.3337	86.714	21.0372	20.4932	24.6217
12.1768	19.4379	1.66258	1.12917	1.2396
28.3484	24.1405	36.632	34.7727	33.7725
36.3389	36.9338	52.3699	50.6556	43.9826
18.1356	18.6589	23.3187	23.8278	25.4154
10.4547	13.4939	8.30408	7.58144	7.69685
10.3501	8.93186	30.0692	32.9531	28.9638
36.8228	45.1415	56.7708	53.9742	69.8044
23.1154	21.1721	36.1067	34.4872	28.8784
83.8085	69.9121	49.1032	49.8142	43.4102
4.86266	2.43812	0.68022	0.703259	1.00355
21.2593	20.3769	12.9664	14.2693	11.0276
12.7907	11.9807	17.9857	19.4069	19.0825
3.84996	2.24078	9.15201	6.41544	7.86337
3.3143	2.9873	1.02817	0.870448	1.31887
319.101	243.138	389.865	398.187	431.338
46.8334	36.2003	28.6412	29.2917	23.9971
167.847	159.129	98.5062	100.239	100.527
0.0886172	0.0592338	0.650468	0.786656	0.634461
53.4633	71.6121	44.0419	39.7847	44.7531
18.418	21.2192	14.7271	13.5566	13.8089

13.3534	11.9527	6.97283	5.12105	6.01618
236.882	236.349	134.694	143.092	175.479
13.8149	10.7521	20.4964	18.7905	16.1138
43.1205	36.8149	25.7242	23.707	26.595
27.6738	22.5937	17.6897	16.0382	15.8028
114.642	94.1801	150.765	152.418	148.6
52.763	55.9733	40.7932	42.1541	42.1761
29.1751	17.227	37.1079	39.173	32.1449
18.9665	23.0077	14.2922	13.3661	13.7259
14.6578	15.4402	7.58115	8.0624	7.65573
68.7828	34.5349	30.0756	25.3224	29.1583
110.839	123.344	88.0875	78.4246	85.2546
33.7616	41.9581	17.5534	18.3481	19.0061
3.57078	3.81381	5.50531	5.02826	5.34202
91.2695	73.7921	118.256	117.496	92.3887
11.8218	11.9468	9.34872	9.35027	6.44644
0.60712	0.873878	0.240745	0.36891	0.3241
1.33773	1.56347	7.16136	7.04389	6.67815
23.4605	29.1429	15.4729	16.8508	17.2927
21.7108	24.8349	28.5504	30.7179	28.1113
49.0398	31.0257	23.8859	24.9297	21.1097
4.82477	5.46492	2.31909	2.78116	3.22927
40.0129	34.6539	61.5465	54.1026	66.5674
41.4204	46.3036	56.8818	51.5785	66.5168
45.4386	43.5763	28.062	32.3622	26.6775
77.0725	85.8183	60.8305	62.0018	62.4455
14.8397	12.8225	8.19572	7.05898	6.9478
1.73494	1.38925	0.122468	0.324778	0.408782
78.5373	120.293	140.605	124.181	140.358
16.767	16.5906	27.5235	25.6689	22.641
37.1446	36.3029	25.3915	25.4454	27.8517
15.8429	13.7422	8.74559	8.22676	8.28497
55.0647	42.9978	39.7484	37.9592	34.7057
1.79659	1.31847	0.262252	0.367773	0.543182
4.72293	5.53162	8.55008	7.63321	8.76319
10.7254	10.3602	7.2478	6.56262	7.01747
55.4639	61.9733	82.4374	83.6671	79.149
41.8124	45.4261	30.6016	28.9341	33.6869
17.6875	23.0948	10.4463	10.3781	10.6031
42.0417	29.635	25.1125	23.822	16.7788
48.8969	59.7498	73.8134	72.1356	89.1821
40.9139	48.0224	31.6601	30.1686	27.9744
85.0756	74.1466	113.605	111.098	85.1455
6.64071	9.7039	1.7812	1.36627	1.67277
7.43044	6.12993	10.3163	10.4258	9.86181
1.46967	0.802458	2.33543	2.72508	2.29092
36.2255	30.1481	102.768	106.15	60.9471
299.644	324.573	399.726	385.485	373.771
78.781	83.8349	103.448	103.129	94.1765
223.761	271.921	132.542	129.206	134.314
33.2341	28.602	23.5706	21.5526	21.9917
23.7408	17.7503	7.6401	7.92949	8.60352
20.9303	19.1132	28.2479	26.8554	29.3805
0.329204	1.43059	4.06694	4.35237	6.71728
64.4141	71.4801	51.3723	49.343	53.8021
4.97544	6.19146	7.76951	8.16931	7.02252
7.38303	8.97648	5.83294	6.1961	5.32709
30.0697	30.8974	38.5719	37.9305	39.1225
27.2746	20.8799	32.1843	33.2697	29.8175
157.569	167.31	93.6992	91.3689	103.651
13.7488	16.1361	6.67458	6.3648	6.21684
50.2965	47.8602	26.5354	28.9836	28.9851
9.78526	10.7951	3.95467	5.92866	7.13004
83.3783	63.9861	99.9201	103.375	92.0411
14.787	13.7721	20.6561	24.4464	18.4897
3.35798	2.15079	0.634687	0.830384	0.645175
0	0	1.4514	1.32329	3.27916
35.0609	28.5564	50.447	48.5982	40.1854
65.5761	55.3973	90.8743	90.9307	72.0522
72.9992	66.717	46.9606	44.3578	37.5381
15.147	12.9592	21.5393	24.682	16.5066
5.62434	6.5821	9.14086	8.73289	9.77241

9.17555	7.37735	13.1192	10.9551	12.5697
34.0283	38.7266	22.7605	22.3935	28.4947
10.763	10.4397	6.86858	6.64409	6.81451
8.06589	9.44793	11.7835	12.3335	11.0678
36.7245	32.0511	52.8519	56.6004	56.5613
29.4241	21.1486	39.8564	38.0276	37.2971
51.5235	50.8399	30.0179	29.0049	25.1924
0.214099	0	3.76848	3.87451	4.13966
1.24319	1.04383	2.11141	2.04201	2.46264
57.0196	21.6874	14.5912	14.7218	10.88
15.3659	12.9225	9.11507	8.25601	9.70866
11.6423	6.54673	4.06427	4.88591	4.87091
6.73847	8.61792	3.41534	3.44334	3.12686
33.8681	27.8685	15.9757	17.3357	13.4059
27.1647	22.7	12.9562	12.617	11.9852
32.0758	26.4051	23.0425	21.1348	19.085
47.3132	82.9401	96.7092	111.014	98.767
30.8928	34.0873	20.5561	23.9095	18.5185
2.38457	2.78326	1.67819	1.48753	0.981339
38.1855	33.6983	56.2315	43.2576	50.0664
24.9915	15.8763	8.93796	8.57668	6.85626
10.9922	15.242	9.68514	9.71916	9.3652
50.1339	50.2401	34.5676	35.4299	34.264
846.104	651.942	1078.06	1095.84	971.407
21.9569	24.0484	15.661	15.9201	16.0619
33.5932	34.3868	53.9032	46.1563	40.8253
8.7578	2.65915	1.48635	0.314586	0.996381
88.7377	56.7601	37.4879	39.4637	47.1498
23.6456	21.0059	31.1081	32.0406	25.5768
138.906	145.566	183.655	183.888	176.029
6.25921	4.62929	4.53859	4.06424	3.0882
10.7487	7.31755	13.7916	15.7098	13.7058
6.06876	5.04996	10.0993	9.31385	9.16597
48.0319	65.7055	29.771	28.8504	39.0474
60.1347	45.0221	32.5682	33.2863	24.8401
15.0612	13.0488	11.2796	11.6376	9.28397
10.4783	10.7071	3.56383	5.91949	4.80983
16.1973	16.6081	12.5273	9.64093	11.5
19.0751	19.0503	12.4567	11.9543	10.9269
29.6	23.1472	39.359	40.9398	33.8008
9.95016	8.04353	7.07583	7.3199	5.76193
53.2458	54.0644	63.3612	61.1952	69.3773
17.5818	19.3913	10.5084	11.1438	9.65489
36.5103	31.0489	29.2987	26.3479	22.8575
2.71999	3.92226	6.21974	7.04224	5.17756
8.77135	5.79878	4.3065	4.76621	4.51484
5.40095	6.32053	8.52229	7.8826	7.30686
37.828	39.0718	23.7448	21.0153	23.1828
6.38091	5.84851	8.96214	8.429	8.68678
10.8775	9.28373	16.153	16.12	14.3983
0.989335	2.34639	0.305279	0.161767	0.35718
95.3196	105.884	133.569	138.463	166.517
74.8235	61.0265	131.158	137.389	97.0372
1.13525	2.11983	2.99441	3.54985	2.34803
12.2898	17.0556	21.9634	20.7533	23.9697
2.61106	3.2605	7.8193	8.6548	7.33172
19.3654	21.6818	29.5052	27.277	31.6462
15.7958	10.1965	21.862	20.943	16.4252
40.985	23.7163	20.5111	20.7367	15.5435
2.73726	2.22804	4.23395	4.69085	3.51847
15.7867	15.0548	21.8072	21.5853	17.711
15.5465	16.8014	10.0225	9.47456	11.0128
4.78273	5.17591	8.18378	7.72872	7.68613
4.49615	4.3469	2.46147	2.59519	2.29726
83.4943	72.523	62.5268	58.1878	52.5894
2.46338	2.24753	1.01821	1.37775	0.87483
3.15562	2.28481	1.44002	1.34044	1.53668
5.09769	7.24614	7.86625	9.32756	9.77267
7.80012	6.16134	4.46995	4.8406	3.66044
11.2313	11.923	8.05017	7.66679	6.61303
14.699	18.5256	9.84061	11.495	11.3879
25.8003	25.7306	13.9426	15.0116	18.6629

1.8578	1.6453	0.61168	0.83143	0.629488
8.58341	9.13072	5.55927	4.58784	6.69855
5.49009	7.92936	9.04116	9.94285	11.0428
2.32864	3.02308	0.787312	1.35776	0.926611
19.097	24.3333	27.058	26.4068	27.6298
6.96159	6.77617	2.56059	1.93213	2.56838
3.17965	2.47326	1.53957	1.62717	1.70056
3.24253	4.40549	6.04623	5.26303	10.3923
1.22225	2.4666	4.85142	4.42639	4.98376
9.3567	10.5002	14.2478	13.7432	16.419
3.63887	3.40847	8.14067	11.0845	15.2691
3.24253	4.40549	6.04623	5.26303	10.3923
1.44571	4.06127	0.239256	0.388208	0.408258
83.7698	69.9659	94.9403	91.7138	98.653
12.1852	6.47324	18.5795	17.4758	14.722
2.77166	2.21732	4.65836	5.36431	4.39291
6.82169	6.83954	10.9789	9.735	8.3221
3.97018	3.77537	6.65224	6.73958	5.95542
10.2291	11.8674	15.5588	14.4918	15.7346
13.9737	10.8966	7.62517	8.00508	7.5385
21.806	25.1838	31.9989	30.6981	30.6764
263.91	224.043	179.027	169.002	189.277

Table S4. The down-regulated targets in sg*Creb3l1* group

Chroms	txStart	txEnd	refseqID	rank product	Strands	GeneSymbol
chr6	59352460	59426290	NM_183183	0.00002275	-	*Gprin3*
chr12	73543113	73546395	NM_178715	0.0000768	-	*Tmem30b*
chr14	69609475	69695834	NM_033325	0.0001157	+	*Loxl2*
chr4	14505196	14621778	NM_145947	0.0001479	-	*Slc26a7*
chr13	51408617	51422797	NM_010101	0.0002086	+	*S1pr3*
chr18	64528978	64661000	NM_001001488	0.0003783	-	*Atp8b1*
chr4	139082291	139092970	NM_008675	0.0003792	-	*Nbl1*
chr3	107741047	107760469	NM_007778	0.0005442	-	*Csf1*
chr9	111439080	111489611	NM_172928	0.000657	+	*Dclk3*
chr2	148404470	148408188	NM_009378	0.0007613	-	*Thbd*
chr3	137623671	137628332	NM_030143	0.001033	+	*Ddit4l*
chr13	114958080	115101964	NM_001033228	0.00127	-	*Itga1*
chr3	55782509	55785287	NM_010750	0.001436	+	*Mab21l1*
chr2	51130438	51149111	NM_028810	0.001461	-	*Rnd3*
chr19	5474689	5481854	NM_021474	0.001608	+	*Efemp2*
chr2	26457901	26503822	NM_008714	0.001621	-	*Notch1*
chr3	108383803	108388231	NM_001190161	0.001792	+	*Psrc1*
chr3	55242525	55539068	NM_001195538	0.001798	+	*Dclk1*
chr4	59581562	59618694	NM_024255	0.001997	+	*Hsdl2*
chr2	152931897	152933208	NM_010226	0.002184	-	*Foxs1*
chr2	62500935	62574021	NM_007986	0.002503	-	*Fap*
chr9	51081312	51102078	NM_177702	0.002594	-	*4833427G06Rik*
chr14	31134852	31139124	NM_027289	0.002998	+	*Nt5dc2*
chr11	78499119	78502325	NM_011707	0.003152	+	*Vtn*
chr13	38345715	38499728	NM_007556	0.003212	+	*Bmp6*
chr2	109917646	110014257	NM_172671	0.003437	+	*Lgr4*
chr6	98238013	98342754	NM_001128092	0.003474	-	*Gm765*
chr4	126435012	126468421	NM_153403	0.003641	-	*Ago1*
chr9	54764747	54773110	NM_013496	0.003669	+	*Crabp1*
chr2	118861999	118881357	NM_019826	0.003762	+	*Ivd*
chr9	107254926	107289877	NM_178907	0.003938	-	*Mapkapk3*
chr3	89215186	89226837	NM_013691	0.005653	+	*Thbs3*
chr7	68273838	68363092	NM_001013811	0.005829	+	*Fam169b*
chr2	152847963	152895321	NM_001141976	0.005992	+	*Tpx2*
chr2	130390774	130397629	NM_019696	0.006186	-	*Cpxm1*
chr19	5490454	5494031	NM_007687	0.006193	+	*Cfl1*
chr4	139192898	139291820	NM_001037761	0.006508	+	*Capzb*
chr2	143078491	143081699	NM_020595	0.007092	+	*Otor*
chr14	31208311	31211711	NM_009393	0.007353	+	*Tnnc1*
chr4	62965573	63134030	NM_028326	0.007916	+	*Zfp618*
chr5	102768809	102897937	NM_001286468	0.008476	+	*Arhgap24*
chr14	73362231	73385271	NM_008410	0.00895	-	*Itm2b*
chr3	66219886	66225806	NM_008987	0.009102	+	*Ptx3*
chr11	75193992	75267762	NM_177708	0.009156	+	*Rtn4rl1*
chr17	56326049	56402873	NM_172132	0.009551	+	*Kdm4b*
chr2	92403646	92434069	NM_009963	0.009836	-	*Cry2*
chr4	88412929	88438926	NM_025760	0.01015	-	*Hacd4*
chr4	106316212	106441327	NM_183225	0.01041	+	*Usp24*
chr9	8994952	9239013	NM_027823	0.01099	-	*Arhgap42*
chr19	6306456	6325652	NM_001033342	0.01117	+	*Cdc42bpg*

Table S4. The down-regulated targets in sgCreb3l1 group

chr1	93235896	93296448	NM_172463	0.01158	+	*Sned1*
chr2	167538226	167542811	NM_011427	0.01166	+	*Snai1*
chr2	91054015	91059438	NM_008948	0.01209	+	*Psmc3*
chr4	134127405	134128753	NM_080559	0.01229	-	*Sh3bgrl3*
chr1	134560177	134632878	NM_152895	0.0128	+	*Kdm5b*
chr9	14541966	14615000	NM_001081395	0.01302	-	*Amotl1*
chr13	94057795	94195409	NM_172589	0.0136	+	*Lhfpl2*
chr19	29020831	29047902	NM_021299	0.01365	-	*Ak3*
chr13	102732488	103334492	NM_175171	0.01502	-	*Mast4*
chr2	125300593	125506438	NM_007993	0.01553	-	*Fbn1*
chr5	4803384	5380251	NM_011074	0.0156	-	*Cdk14*
chr2	170731806	170879775	NM_029761	0.01567	+	*Dok5*
chr19	53140442	53247326	NM_001164101	0.01602	+	*Add3*
chr13	59634995	59675784	NM_027307	0.01606	-	*Golm1*
chr3	114030539	114220326	NM_007729	0.01609	+	*Col11a1*
chr19	8989283	9076926	NM_001039959	0.01649	+	*Ahnak*
chr17	72918304	72941946	NM_029999	0.01812	+	*Lbh*
chr9	45838528	45862484	NM_001145947	0.01907	+	*Bace1*
chr3	61364506	61368703	NM_028712	0.01924	+	*Rap2b*
chr4	120697472	120747176	NM_001081142	0.02095	-	*Kcnq4*
chr19	8941919	8952300	NM_011842	0.02145	+	*Mta2*
chr12	108860029	108894174	NM_001164314	0.02206	-	*Wars*
chr13	54071844	54108345	NM_027324	0.02237	+	*Sfxn1*
chr1	56793980	56971334	NM_139146	0.02559	-	*Satb2*
chr2	167607638	167632005	NM_023230	0.02706	-	*Ube2v1*
chrX	71050255	71154717	NM_001256048	0.02785	+	*Mamld1*
chr4	121145372	121188534	NM_001081013	0.02825	-	*Rlf*
chr4	135946452	135962617	NM_008254	0.02826	+	*Hmgcl*
chr4	9269316	9451691	NM_028940	0.02853	+	*Clvs1*
chr5	149411805	149431703	NM_027519	0.02929	+	*Medag*
chr19	3584824	3686564	NM_008513	0.02979	-	*Lrp5*
chr4	10874497	10899423	NM_026005	0.0301	+	*2610301B20Rik*
chr9	54717152	54734549	NM_001025375	0.03011	-	*Wdr61*
chr2	37443284	37566437	NM_146121	0.03167	+	*Rabgap1*
chr4	57845247	57896984	NM_009649	0.03228	+	*Akap2*
chr14	31124504	31128930	NR_024069	0.03232	-	*Smim4*
chr4	151047304	151057953	NM_009498	0.0326	-	*Vamp3*
chr19	8967040	8978180	NM_026007	0.03299	+	*Eef1g*
chr17	84705246	84790786	NM_028233	0.03522	-	*Lrpprc*
chr9	46012819	46224194	NM_027498	0.03538	+	*Sik3*
chr3	108074061	108086666	NM_001289719	0.03555	-	*Ampd2*
chr2	142618344	142901464	NM_001081133	0.03614	-	*Kif16b*
chr17	56613394	56614246	NM_018730	0.04073	+	*Rpl36*
chr19	8927381	8929356	NM_009073	0.0413	-	*Rom1*
chr2	71118053	71142926	NM_028593	0.04326	+	*Cybrd1*
chr13	59755414	59769789	NM_026921	0.04367	-	*Isca1*
chr4	133130632	133198330	NM_153423	0.04654	+	*Wasf2*
chr17	62602956	62881317	NM_010109	0.04665	-	*Efna5*
chr19	52991179	53038654	NM_133216	0.04946	-	*Xpnpep1*
chr14	69767471	69784411	NM_020275	0.05257	+	*Tnfrsf10b*
chr13	116854823	117025516	NM_001081009	0.05347	-	*Parp8*
chr4	128993223	129011529	NM_016895	0.05855	+	*Ak2*

Table S4. The down-regulated targets in sgCreb3l1 group

Table S5. The associated peaks of down-regulated targets in sg*Creb3l1* group

chrom	pStart	pEnd	Refseq	Symbol	Distance	Score
chr6	59431701	59432201	NM_183183	*Gprin3*	5661	0.483627643
chr6	59434521	59435021	NM_183183	*Gprin3*	8481	0.432038748
chr12	73507147	73507647	NM_178715	*Tmem30b*	-38998	0.127464167
chr12	73536085	73536585	NM_178715	*Tmem30b*	-10060	0.405595063
chr12	73536986	73537486	NM_178715	*Tmem30b*	-9159	0.420479312
chr14	69692418	69692918	NM_033325	*Loxl2*	83193	0.02175917
chr4	14558126	14558626	NM_145947	*Slc26a7*	-63402	0.04802277
chr4	14621662	14622162	NM_145947	*Slc26a7*	134	0.603288353
chr13	51408581	51409081	NM_010101	*S1pr3*	214	0.601360915
chr18	64660750	64661250	NM_001001488	*Atp8b1*	0	0.60653066
chr4	139087360	139087860	NM_008675	*Nbl1*	-5360	0.489485715
chr4	139175302	139175802	NM_008675	*Nbl1*	82582	0.022297516
chr3	107801389	107801889	NM_007778	*Csf1*	41170	0.116857504
chr9	111429316	111429816	NM_172928	*Dclk3*	-9514	0.414550698
chr9	111460400	111460900	NM_172928	*Dclk3*	21570	0.255943132
chr2	148346017	148346517	NM_009378	*Thbd*	-61921	0.050953593
chr2	148364501	148365001	NM_009378	*Thbd*	-43437	0.106727118
chr3	137525661	137526161	NM_030143	*Ddit4l*	-97760	0.012150317
chr3	137547944	137548444	NM_030143	*Ddit4l*	-75477	0.029626679
chr13	115037265	115037765	NM_001033228	*Itga1*	-64449	0.046053109
chr13	115199134	115199634	NM_001033228	*Itga1*	97420	0.01231669
chr3	55779977	55780477	NM_010750	*Mab21l1*	-2282	0.5536182
chr2	51117934	51118434	NM_028810	*Rnd3*	-30927	0.176033669
chr19	5471783	5472283	NM_021474	*Efemp2*	-2656	0.545397714
chr2	26556176	26556676	NM_008714	*Notch1*	52604	0.073965242
chr3	108379234	108379734	NM_001190161	*Psrc1*	-4319	0.510298209
chr3	55248012	55248512	NM_001195538	*Dclk1*	5737	0.482159648
chr4	59585044	59585544	NM_024255	*Hsdl2*	3732	0.522421785
chr2	152898950	152899450	NM_010226	*Foxs1*	-34008	0.155622823
chr2	152907539	152908039	NM_010226	*Foxs1*	-25419	0.219421663
chr2	62505258	62505758	NM_007986	*Fap*	-68513	0.039143535
chr2	62602675	62603175	NM_007986	*Fap*	28904	0.190870517
chr9	51105672	51106172	NM_177702	*4833427G06Rik*	3844	0.52008657
chr14	31079285	31079785	NM_027289	*Nt5dc2*	-55317	0.066358727
chr14	31226870	31227370	NM_027289	*Nt5dc2*	92268	0.015135383
chr11	78505379	78505879	NM_011707	*Vtn*	6510	0.467479398
chr13	38279073	38279573	NM_007556	*Bmp6*	-66392	0.042609417
chr13	38347657	38348157	NM_007556	*Bmp6*	2192	0.555614817
chr2	110005109	110005609	NM_172671	*Lgr4*	87713	0.018160253
chr6	98342463	98342963	NM_001128092	*Gm765*	-41	0.605536765
chr4	126496169	126496669	NM_153403	*Ago1*	27998	0.197914532
chr9	54782364	54782864	NM_013496	*Crabp1*	17867	0.296804977
chr2	118775321	118775821	NM_019826	*Ivd*	-86428	0.019118095
chr9	107279766	107280266	NM_178907	*Mapkapk3*	-9861	0.408836483
chr3	89210562	89211062	NM_013691	*Thbs3*	-4374	0.509176787
chr7	68337233	68337733	NM_001013811	*Fam169b*	63645	0.04755825
chr2	152898950	152899450	NM_001141976	*Tpx2*	51237	0.078122279
chr2	152907539	152908039	NM_001141976	*Tpx2*	59826	0.055407517
chr2	130353843	130354343	NM_019696	*Cpxm1*	-43536	0.106305314
chr19	5471783	5472283	NM_007687	*Cfl1*	-18421	0.290300118

chr4	139175302	139175802	NM_001037761	*Capzb*	-17346	0.303055295
chr2	143012989	143013489	NM_020595	*Otor*	-65252	0.044597387
chr2	143018553	143019053	NM_020595	*Otor*	-59688	0.055714213
chr14	31226870	31227370	NM_009393	*Tnnc1*	18809	0.285829443
chr4	62998431	62998931	NM_028326	*Zfp618*	33108	0.161327309
chr4	63064817	63065317	NM_028326	*Zfp618*	99494	0.011336134
chr5	102794531	102795031	NM_001286468	*Arhgap24*	25972	0.214621343
chr14	73399803	73400303	NM_008410	*Itm2b*	14782	0.335786412
chr14	73439811	73440311	NM_008410	*Itm2b*	54790	0.067772417
chr3	66180798	66181298	NM_008987	*Ptx3*	-38838	0.128282553
chr11	75240479	75240979	NM_177708	*Rtn4rl1*	46737	0.093529349
chr11	75289913	75290413	NM_177708	*Rtn4rl1*	96171	0.012947663
chr17	56331249	56331749	NM_172132	*Kdm4b*	5450	0.487726735
chr2	92426348	92426848	NM_009963	*Cry2*	-7471	0.449850488
chr4	88427572	88428072	NM_025760	*Hacd4*	-11104	0.389006199
chr4	106261198	106261698	NM_183225	*Usp24*	-54764	0.067842937
chr9	9196004	9196504	NM_027823	*Arhgap42*	-42759	0.109661163
chr19	6384816	6385316	NM_001033342	*Cdc42bpg*	78610	0.026137087
chr1	93211397	93211897	NM_172463	*Sned1*	-24249	0.229934682
chr2	167546840	167547340	NM_011427	*Snai1*	8864	0.425470357
chr2	91027374	91027874	NM_008948	*Psmc3*	-26391	0.211054265
chr4	134136260	134136760	NM_080559	*Sh3bgrl3*	7757	0.444733523
chr1	134516514	134517014	NM_152895	*Kdm5b*	-43413	0.106829625
chr9	14599699	14600199	NM_001081395	*Amotl1*	-15051	0.332192719
chr13	93996528	93997028	NM_172589	*Lhfpl2*	-61017	0.052829792
chr13	94100421	94100921	NM_172589	*Lhfpl2*	42876	0.109149148
chr19	29014449	29014949	NM_021299	*Ak3*	-33203	0.160715429
chr13	103315294	103315794	NM_175171	*Mast4*	-18948	0.284244641
chr13	103400145	103400645	NM_175171	*Mast4*	65903	0.043451061
chr2	125415443	125415943	NM_007993	*Fbn1*	-90745	0.016086096
chr5	5359854	5360354	NM_011074	*Cdk14*	-20147	0.270934008
chr2	170772012	170772512	NM_029761	*Dok5*	40456	0.12024307
chr19	53107236	53107736	NM_001164101	*Add3*	-32956	0.162311167
chr13	59718888	59719388	NM_027307	*Golm1*	43354	0.107082041
chr3	114065409	114065909	NM_007729	*Col11a1*	35120	0.14885241
chr3	114082032	114082532	NM_007729	*Col11a1*	51743	0.076556978
chr19	9022947	9023447	NM_001039959	*Ahnak*	33914	0.156209066
chr17	72965924	72966424	NM_029999	*Lbh*	47870	0.089385214
chr9	45917435	45917935	NM_001145947	*Bace1*	79157	0.025571419
chr3	61419725	61420225	NM_028712	*Rap2b*	55469	0.06595649
chr4	120725878	120726378	NM_001081142	*Kcnq4*	-21048	0.261343407
chr19	9022947	9023447	NM_011842	*Mta2*	81278	0.023491421
chr12	108915880	108916380	NM_001164314	*Wars*	21956	0.252021721
chr13	54122187	54122687	NM_027324	*Sfxn1*	50593	0.080160853
chr1	57040026	57040526	NM_139146	*Satb2*	68942	0.038477562
chr2	167546840	167547340	NM_023230	*Ube2v1*	-84915	0.020310851
chrX	71108031	71108531	NM_001256048	*Mamld1*	58026	0.059543985
chr4	121216137	121216637	NM_001081013	*Rlf*	27853	0.199065771
chr4	135879034	135879534	NM_008254	*Hmgcl*	-67168	0.041307136
chr4	9323881	9324381	NM_028940	*Clvs1*	54815	0.067704678
chr4	9336672	9337172	NM_028940	*Clvs1*	67606	0.040589738
chr5	149381729	149382229	NM_027519	*Medag*	-29826	0.183959436
chr19	3720613	3721113	NM_008513	*Lrp5*	34299	0.153821875

Table S5. The associated peaks of down-regulated targets in sgCreb3l1 group

chr4	10909600	10910100	NM_026005	*2610301B20Rik*	35353	0.14747155
chr9	54782364	54782864	NM_001025375	*Wdr61*	48065	0.088690722
chr2	37370586	37371086	NM_146121	*Rabgap1*	-72448	0.033442759
chr4	57775510	57776010	NM_009649	*Akap2*	-69487	0.037647829
chr14	31079285	31079785	NR_024069	*Smim4*	-49395	0.084095687
chr14	31226870	31227370	NR_024069	*Smim4*	98190	0.011943119
chr4	151132443	151132943	NM_009498	*Vamp3*	74740	0.030513075
chr19	9022947	9023447	NM_026007	*Eef1g*	56157	0.064166116
chr17	84747210	84747710	NM_028233	*Lrpprc*	-43326	0.10720204
chr9	45917435	45917935	NM_027498	*Sik3*	-95134	0.013496026
chr9	45975615	45976115	NM_027498	*Sik3*	-36954	0.138323519
chr3	108174646	108175146	NM_001289719	*Ampd2*	88230	0.017788555
chr2	142838135	142838635	NM_001081133	*Kif16b*	-63079	0.04864725
chr17	56515988	56516488	NM_018730	*Rpl36*	-97156	0.012447444
chr19	9022947	9023447	NM_009073	*Rom1*	93841	0.014212406
chr2	71031519	71032019	NM_028593	*Cybrd1*	-86284	0.019228533
chr13	59718888	59719388	NM_026921	*Isca1*	-50651	0.079975095
chr4	133070416	133070916	NM_153423	*Wasf2*	-59966	0.055098103
chr17	62961958	62962458	NM_010109	*Efna5*	80891	0.023857897
chr19	53107236	53107736	NM_133216	*Xpnpep1*	68832	0.038647237
chr14	69692418	69692918	NM_020275	*Tnfrsf10b*	-74803	0.030436279
chr13	116949484	116949984	NM_001081009	*Parp8*	-75782	0.02926743
chr4	128906637	128907137	NM_016895	*Ak2*	-86336	0.019188579

Table S5. The associated peaks of down-regulated targets in sgCreb3l1 group

**Table S6. The sequences for the *Tmem30b* gRNAs and for the primers used in identification.**	
**gRNAs**	
Name	Sequence
sgRNA_a	GAGCTGGAGTACGACTACAC
sgRNA_b	GCGATGGCGGTGCGATCGAG
**Primers**	
Name	Sequence
P_a	CTGGGCCTCTTCTACTCCT
P_b	AGTCCTGGTTGATGAAGCCG

Table S6. The sequences for the Tmem30b gRNAs and for the primers used in identification.

Table S7. The sequences for the primers used in qRT-PCR.	
Name	Sequence
Creb3l1 -F	GCCCTGGGAAACAAGCTGT
Creb3l1 -R	AGCTGAGTCATTTCTCCTGGG
Dmp1 -F	ACCACAATACTGAATCTGAAAGCTC
Dmp1 -R	TGCTGTCCGTGTGGTCACTA
Dspp -F	GTGGGATCATCAGCCAGTCAG
Dspp -R	TGCCTTTGTTGGGACCTTCA
Tmem30 b-F	TGTACCTCTACTACGAGCTGTC
Tmem30b -R	CACGGTGCGATAGGCAGAC
Gapdh -F	TGTGTCCGTCGTGGATCTGA
Gapdh -R	TTGCTGTTGAAGTCGCAGGAG

Table S7. The sequences for the primers used in qRT-PCR.

Supplementary materials and methods

Hematoxylin and eosin staining (HE)

The mandibles collected from PN 0.5, PN 3W, and PN 8W mice were fixed individually in 4% paraformaldehyde (PFA; Biosharp, Hefei, China) overnight at 4 °C and decalcified in 10% ethylenediaminetetraacetic acid (EDTA, pH 7.4) for 3 days to 6 weeks depending on the age of mice. Hematoxylin and eosin staining was performed on serial deparaffinized sections following a standard procedure.

Immunohistochemistry (IHC)

The mandibles of PN 0.5 mice were fixed in 4% paraformaldehyde (PFA; Biosharp, China) overnight. Mandibles were decalcified in 10% EDTA (pH 7.4) for 3 days. All samples were dehydrated and embedded in paraffin. Sagittal sections of a 6 μm thickness were de-waxed, rehydrated, and then digested in pepsin for antigen retrieval. The slides were incubated with anti-CREB3L1 (11235-2-AP, 1:200, Proteintech, Wuhan, Chinese) antibodies at 4 °C overnight. Specimens were visualized using a diaminobenzidine reagent kit (Maixin, China) after incubation with horseradish peroxidase (HRP) secondary antibody. Then, the immunostained sections were counterstained with hematoxylin.

Scanning electron microscope (SEM)

The mandibles of PN 8W WT and cKO mice were first fixed in a solution containing 2.5% glutaraldehyde (pH 7.4) at room temperature for 2 hours, then

sanded, and etched. After spraying gold, scanning electron microscopy (SEM) (TESCAN, Brno, Kohoutovice Czech Republic) was performed.

Cell culture and differentiation induction

The mouse dental papilla cell line mDPC6T-Cas9 was infected with lentivirus encoding CAS9 (EF1A-spCAS9) as described previously.[1] mDPC6T-Cas9 cells were cultured in DMEM (HyClone, Logan, Utah) containing 10% fetal bovine serum (Gibco, Australia), in a humidified 37 °C incubator with a 5% CO2 atmosphere. For obtaining the cell extracellular supernatant proteins, mDPC6T-Cas9 cells were cultured in DMEM containing 0.5% fetal bovine serum. For differentiation induction, as previously reported,[2] mDPC6T cells were supplemented with 50 mg/mL ascorbic acid (Sigma, St Louis, MO, USA), 10 mM sodium β-glycerophosphate (Sigma), and 10 nM dexamethasone (Sigma) for 0, 12 and 24 hours or for 0, 1, 3, 5, 7 and 9 days. All media were refreshed every 2 days.

For the downregulation of *Creb3l1*, one pair of single guide RNAs (sgRNAs) was designed for transfection of mDPC6T-Cas9 cells. mDPC6T-Cas9 cells were transfected with sgRNAs at a final concentration of 144 nM. The monoclonal cell line was obtained by limiting dilution, and the genomic DNAs from all single cell derived expanded clones were analyzed by PCR amplification and Sanger sequencing.

For the knockout of *Tmem30b,* one pair of sgRNAs was designed, the following steps just as described in the downregulation of *Creb3l1* by sgRNAs. The sequences for the sgRNAs were listed in Table S6, and the sgRNAs were synthesized by GenScript (Nanjing, China). The primers used in genotyping were also listed in Table S6 and synthesized by Sangon Biotech Co., Ltd. (Shanghai, China).

Western blot analysis

Cells were harvested at different time points and lysed in lysis buffer (71009-M, Merck Millipore, St Louis, MO, United States) with phosphatase and protease inhibitor (4693132001 and 4906837001, Roche, St Louis, MO, United States). Nuclear and cytoplasmic protein was separated with Nuclear and Cytoplasmic Protein Extraction Kit (P0028, Beyotime, Shanghai, China). Supernatant proteins were harvested by centrifugation at 13000 rpm for 10 minutes at 4°C. Then, protein concentrations in the lysates were measured and standardized using a BCA Protein Assay Kit (23225, Thermo Fisher Scientific, Rockford, Illinois). Equal amounts of proteins were loaded and separated by 10% polyacrylamide gel electrophoresis, then transferred onto polyvinylidene fluoride (PVDF) membranes (ISEQ00010, Merck Millipore, St Louis, MO, United States). The membranes were blocked with 5% nonfat milk in TBST for 1 hour at room temperature, and subsequently incubated at 4°C overnight with the following primary antibodies: anti-CREB3L1 (11235-2-AP, 1:1000, Proteintech, Wuhan, Chinese) or anti-TMEM30B (ab185944, 1:1000, Abcam, Cambridge, UK), anti-DMP1 (3844-100,1:1000, BioVision, CA, USA), anti-DSPP (A8413, 1:1000, ABclonal, Wuhan, China), and anti-FLAG (F1804, 1:1000, Sigma-Aldrich, St Louis, MO, United States). LAMIN B1 (ab229025, 1:1000, Abcam, Cambridge, UK) was used as the nucleus normalization control. GAPDH (GTX100118, 1:8000, GeneTex, Alton, USA) was used as the cytoplasm loading control. anti-β-ACTIN (PMK058, 1:5000, BioPM, Wuhan, China) was used as an internal reference for protein normalization. Next, the membranes were incubated with goat anti-rabbit (AP132P, 1:4000, Sigma-Aldrich, St Louis, MO, United States) or goat anti-mouse (AP124P, 1:4000, Sigma-Aldrich, St Louis, MO, United States) peroxidase-conjugated secondary antibody for 1 hour at room temperature. WesternBright ECL solution (Advansta, San Jose, California) was used to detect the signals. Protein bands were

detected with an enhanced chemiluminescence system and measured by densitometric analysis with ImageJ software.

Total RNA isolation and quantitative reverse transcription PCR assay

Total RNA was extracted from cultured cells using the HP Total RNA Kit (Omega Bio-Tek, Norcross, Georgia). cDNA was synthesized using the Revert Aid First Strand cDNA Synthesis Kit (K1621, Thermo Fisher Scientific, Rockford, Illinois) according to the manufacturer's instructions. qRT-PCR was performed using the CFX Connect Real-Time System (1855201, Bio-Rad, Hercules, California) and the HiScript II One Step qRT-PCR SYBR Green Kit (Vazyme, Nanjing, China). The expression levels of *Creb3l1*, *Dmp1*, *Dspp*, *Tmem30b,* and *Gapdh* were quantified. *Gapdh* was used as the internal normalization control. The sequences for the primers used in qRT-PCR are shown in Table S7, and they were synthesized by Sangon Biotech Co., Ltd. The $2^{-\Delta\Delta Ct}$ method was applied to calculate gene expression.

Alizarin Red S Staining

Cells were treated with differentiation induction medium and collected at day 0 (D0) and day 14 (D14). Cells were washed with PBS and then fixed in 95% ethyl alcohol for 10 minutes, followed by staining with 1% alizarin red S (Sigma) solution. Nodules were recorded in bright field using an inverted microscope (Axiovert 40; Zeiss, Jena, Germany).

Assay for transposase-accessible chromatin with high-throughput sequencing (ATAC-seq) library preparation

mDPC6T-Cas9 cells and sg*Creb3l1* were cultured in differentiation medium for 5 days. ATAC-seq libraries were generated and indexed with a TruePrep DNA Library Prep Kit (TD501, Vazyme, Nanjing, China) as previously described.[3] Approximately 50,000 cells in each biological replicate were harvested and dissociated using a cell strainer via centrifugation (750×g) for 5 minutes at room temperature. The cell pellets were resuspended in lysis buffer (10 mM Tris-HCl, pH 7.5, 10 mM NaCl, 3 mM MgCl2, 0.1% NP-40) and then subjected to centrifugation (500×g) for 15 minutes at 4 °C. The supernatant was discarded, and the pelleted nuclei were immediately submitted to a tagmentation reaction using Tn5 transposase (TTE Mix V50) for 30 minutes at 37 °C. Then, DNA was purified using a Qiagen PCR purification MinElute Kit (Qiagen, Valencia, CA, USA) and eluted in 10 μL of elution buffer (EB). The purified DNA was then indexed and amplified to generate the final ATAC-seq libraries. Finally, all libraries were purified with VAHTS DNA Clean Beads and sequenced on the Illumina Novaseq 6000. Three independent biological replicates were performed for both Ctrl and sg*Creb3l1* groups.

Analysis of ATAC-seq library

Raw reads of ATAC-seq were first subjected to trimmomatic (v.0.38)[4] for adaptor trimming. Quality inspections were performed before alignment using FastQC (https://www.bioinformatics.babraham.ac.uk/projects/fastqc/) to ensure the proportionate quality of all libraries. Then, the paired-end sequencing reads were aligned to the mouse genome (mm10) using Bowtie 2.[5] SAMtools[6] was used to remove the PCR duplicates. DeepTools2[7] was applied to generate bigwig files. MACS2 (v.2.1.1)[8] was used to accomplish peak calling. The comparison of differentially accessible nucleosome-free regions (NFRs) between different groups was conducted using DiffBind (DESeq2 v.1.26.0)[9]. The Genomic Regions Enrichment of Annotations Tool (GREAT)[10] was utilized

to annotate differentially accessible NFRs and perform the functional enrichment assay using the default parameter. Finally, coverage plots for ATAC-seq results were generated using DeepTools2, uploaded to Cyverse, and visualized with the UCSC genome browser. All correlative graphs were plotted using custom R scripts in RStudio (v. 2022.02.0+443).

RNA-Seq Library Generation and Data Analysis

For RNA-seq, poly(A) mRNA was purified from total RNA and sent to Novogene Bioinformatics Technology Co. Ltd. for analysis. Gene levels were quantified with Kallisto (v 0.44.0)[11] to quantify the abundance of transcripts and the Sleuth R package[12] with a cutoff p-value <0.01 for differential analysis. Metascape[13] and GSEA (v 3.0)[14] were utilized for gene ontology analysis and gene set enrichment assay, respectively.

Binding and Expression Target Analysis (BETA)

To integrate ATAC-seq and RNA-seq data, we utilized the BETA[15] to facilitate the identification of candidate target genes and associated regulatory elements affected by *Creb3l1* downregulation. This tool can identify target genes and associated peaks that display a consistent trend in their changes in *Creb3l1* deficient group, and subsequently rank them based on their score by comparing the lost NFRs in ATAC-seq with all genes in RNA-seq. (Table S4, 5).

siRNA transfection

mDPC6T-Cas9 cells were transfected with si*Tmem30b* and a negative control scrambled siRNA (both were purchased from Genecreate Biological

Engineering Co., Ltd, Wuhan, China) using Lipofectamine 2000, following the manufacturer's instructions, with a final concentration of 100 nM for both siRNAs. The sequences of si*Tmem30b* were 5’ - GCUGUCCAACUUCUACCAGAA-3’ (sense) and 5’ - UUCUGGUAGAAGUUGGACAGC-3’ (antisense). For differentiation induction, mDPC6T-Cas9 cells were transfected every three days with siRNA.

Overexpression of *Flag-Tmem30b* and *Flag-Creb3l1*

The mDPCs-Cas9, sg*Creb3l1*_A8-4, and sg*Tmem30b*_60 cells were seeded so that the cells were 30% confluent at the time of transfection. The mDPCs-Cas9 cells were infected with the lentivirus vector CON522 (GeneChem, China) according to the manufacturer’s instructions. The sg*Creb3l1*_A8-4 Cells were infected with *Flag-Tmem30b* cloned into the lentivirus vector CON522 (GeneChem, China) (oe*Tmem30b*) or vector only. The sg*Tmem30b*_60 cells were infected with the lentivirus vector CON522. *Flag-Creb3l1* cloned into the lentivirus vector pCDH were constructed (oe*Creb3l1*). The mDPCs-Cas9 cells were infected with the lentivirus vector pCDH. The sg*Tmem30b*_60 cells were infected with the lentivirus oe*Creb3l1* or the vector pCDH.

Dual luciferase activity assay

Dual luciferase activity assays were carried out in mDPC6T-Cas9 cells. The sequence was designed approximately 300bp upstream and downstream of the predicted *Creb* family motif of the potential enhancer region of *Tmem30b,* and inserted into the pGL3-promoter vector (T1). The remaining sequence after deletion of the motif region was also inserted into the pGL3-promoter vector as mutant (T1_mut). T1, T1_mut, and overexpression of *Creb3l1* plasmid

(oe*Creb3l1*) were all synthesized by Genecreate Biological Engineering Co., Ltd. The pGL3-promoter vector plasmid (Ctrl), T1, or T1_mut plasmids were cotransfected with oe*Creb3l1.* Luciferase activity was detected 48 hours after transfection using the Dual-Luciferase Reporter Assay System (Promega) following the manufacturer's instructions. Three independent biological replicates were performed for each experiment.

Statistical analysis

All data in this study were presented as mean ± standard deviation (SD). The GraphPad Prism 8.0 software (GraphPad Software, CA, USA) was utilized for statistical analysis and visualization of the data. The statistical difference was analyzed by two-tailed Student's t test between the two groups. $p < 0.05$ was considered statistically significant.

REFERENCES

1. Zuo, H. *et al.* Phosphorylation of ATF2 promotes odontoblastic differentiation via intrinsic HAT activity. *J Genet Genomics* **50**, 497-510 (2023).
2. Lin, H. *et al.* KLF4 promotes the odontoblastic differentiation of human dental pulp cells. *J. Endod.* **37**, 948-954 (2011).
3. Buenrostro, J. D., Giresi, P. G., Zaba, L. C., Chang, H. Y. & Greenleaf, W. J. Transposition of native chromatin for fast and sensitive epigenomic profiling of open chromatin, DNA-binding proteins and nucleosome position. *Nat Methods* **10**, 1213-1218 (2013).
4. Bolger, A. M., Lohse, M. & Usadel, B. Trimmomatic: a flexible trimmer for Illumina sequence data. *Bioinformatics* **30**, 2114-2120 (2014).
5. Langmead, B. & Salzberg, S. L. Fast gapped-read alignment with Bowtie 2. *Nat Methods* **9**, 357-359 (2012).
6. Li, H. *et al.* The Sequence Alignment/Map format and SAMtools. *Bioinformatics* **25**, 2078-2079 (2009).
7. Ramírez, F. *et al.* deepTools2: a next generation web server for deep-sequencing data analysis. *Nucleic Acids Res.* **44**, W160-165 (2016).
8. Zhang, Y. *et al.* Model-based analysis of ChIP-Seq (MACS). *Genome Biol.* **9**, R137 (2008).
9. Love, M. I., Huber, W. & Anders, S. Moderated estimation of fold change and dispersion for RNA-

seq data with DESeq2. *Genome Biol.* **15**, 550 (2014).

10. McLean, C. Y. *et al.* GREAT improves functional interpretation of cis-regulatory regions. *Nat. Biotechnol.* **28**, 495-501 (2010).
11. Bray, N. L., Pimentel, H., Melsted, P. & Pachter, L. Near-optimal probabilistic RNA-seq quantification. *Nat. Biotechnol.* **34**, 525-527 (2016).
12. Pimentel, H., Bray, N. L., Puente, S., Melsted, P. & Pachter, L. Differential analysis of RNA-seq incorporating quantification uncertainty. *Nat Methods* **14**, 687-690 (2017).
13. Tripathi, S. *et al.* Meta- and Orthogonal Integration of Influenza "OMICs" Data Defines a Role for UBR4 in Virus Budding. *Cell Host Microbe* **18**, 723-735 (2015).
14. Subramanian, A. *et al.* Gene set enrichment analysis: a knowledge-based approach for interpreting genome-wide expression profiles. *Proc. Natl. Acad. Sci. U. S. A.* **102**, 15545-15550 (2005).
15. Wang, S. *et al.* Target analysis by integration of transcriptome and ChIP-seq data with BETA. *Nat. Protoc.* **8**, 2502-2515 (2013).
